# Predicting the points of interaction of small molecules in the NF-κB pathway

**DOI:** 10.1186/1752-0509-5-32

**Published:** 2011-02-22

**Authors:** Yogendra Patel, Catherine A Heyward, Michael RH White, Douglas B Kell

**Affiliations:** 1 Manchester Interdisciplinary Biocentre, University of Manchester, Manchester, 131 Princess Street, M1 7DN, UK; 2 Institute of Integrative Biology, University of Liverpool, Liverpool, L69 7ZB, UK; 3 Faculty of Life Sciences, Michael Smith Building, Oxford Road, University of Manchester, Manchester, M13 9PT, UK

## Abstract

**Background:**

The similarity property principle has been used extensively in drug discovery to identify small compounds that interact with specific drug targets. Here we show it can be applied to identify the interactions of small molecules within the NF-κB signalling pathway.

**Results:**

Clusters that contain compounds with a predominant interaction within the pathway were created, which were then used to predict the interaction of compounds not included in the clustering analysis.

**Conclusions:**

The technique successfully predicted the points of interactions of compounds that are known to interact with the NF-κB pathway. The method was also shown to be successful when compounds for which the interaction points were unknown were included in the clustering analysis.

## Background

**Figure 1:**
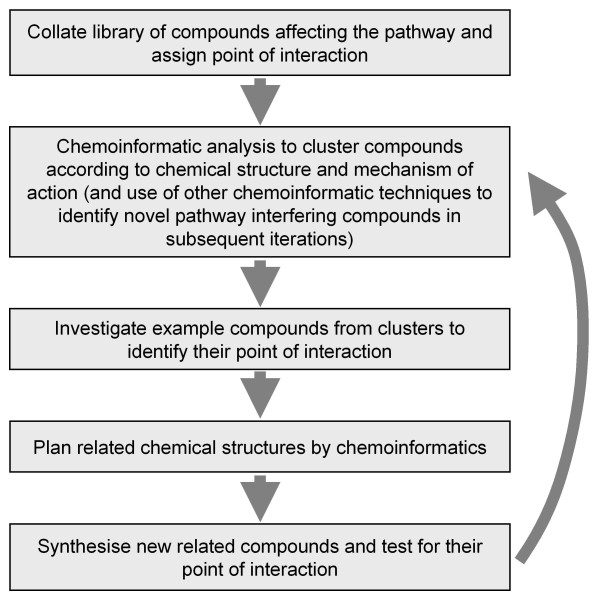
**Methodology of using Chemoinformatics in Systems Biology**. Outline of the methodology involved in applying chemoinformatics to a systems biology problem.

A major challenge of systems biology is to use computational modelling to predict new targets for chemical intervention. Systems biology involves the quantitative analysis and integration of individual components of a biological system leading to a better understanding of the dynamic and regulatory properties of the system[1-3]. Chemical biology, on the other hand, involves the screening of a set of chemical entities to determine their effects on the function of a system. The combination of these approaches can allow a better understanding of the system network through the identification of new cellular reactions at which new chemical entities perturb the system[4-6]. Figure 1 outlines the methodology involved.

One of the most studied cellular signalling systems is the Nuclear Factor κB (NF-κB) network. The NF-κB family of transcription factors controls the transcription of at least 300 genes, but has different transcriptional and cell fate outcomes in different cells and in response to different stimuli. As well as being a critical component of the innate immune response, NF-κB controls cell division and apoptosis in most cell types. While the NF-κB signalling pathway has been studied in many papers (nearly 30,000 are returned by a PubMed search for "Nuclear Factor kappa B"), there is still a great deal about the system which is not understood. Recently, NF-κB proteins have been shown to oscillate between the cytoplasm and nucleus of stimulated cells[7] and the frequency of these oscillations has been suggested to alter the pattern of gene expression[8]. The discovery of the importance of these dynamic processes requires a re-interpretation of the previous literature.

NF-κB has been a much studied drug target in the pharmaceutical industry. Numerous traditional medicines have been shown to contain compounds that affect NF-κB activity. Many of these are now being investigated for pharmaceutical development, for example gambogic acid[9], caffeic acid phenyl ester[10], green tea polyphenols (reviewed by Khan and Mukhtar[11]). In addition, NF-κB antisense oligonucleotides have recently been shown to affect outcome in a murine endotoxic shock model[12] and NF-κB decoy oligonucleotides are of interest as potential therapy for inflammatory diseases (for review see [13]). The effects of NF-κB modulating drugs have been measured mostly using assays for NF-κB function that have been limited to easily available endpoints such as IκB degradation or DNA binding. As a result the interpretation of the site of action of these compounds may require re-analysis. The combination of the limited characterisation of the site of action, as well as the limited understanding of the NF-κB network, has meant that it has been difficult to interpret and compare the action of different NF-κB inhibitors. Here we use chemoinformatic approaches to cluster a set of known NF-κB modulatory compounds.

The methodology is based on the similar property principle[14] (structurally similar compounds have similar properties), although it must be noted that there are flaws with the principle. The main flaw is that small structural changes can lead to a dramatic change in property (e.g. changing a hydrogen bond donor for an acceptor activity can greatly increase activity against a target), which has a major impact in studying quantitative structure activity/property relationships[15]. In this study we use it as a general rule rather than a specific rule. In addition to identifying relationships between clusters of compounds and their biological functions, clusters were also used to identify the points of interaction of compounds (which are known to interact with the NF-κB pathway) not used in the clustering analysis.

The compounds were obtained from a literature search, which in many instances involved manually searching for chemical structures using chemical names present in the literature. Structures for the compounds can be found in the Additional File 1 (chiral information has been included where known; however only 2D information was used in the work presented here). Since the creation of this list, advances in text mining mean it is now possible to automatically extract names of compounds from the literature and associate the names with structures, for example using Pipeline Pilots' ChemMining Collection[16] or OSCAR3[17]. The resulting list of compounds from the literature search could look like the diverse set collated here. Such lists obtained for a cellular pathway could be used (as here) to identify compounds which interact in a similar manner in a given pathway. A point to note is that here, this technique has not been used to identify novel compounds that interact within the pathway, but rather to identify the point of interaction of compounds which are known to affect the pathway. As an additional aim of this work, we have used all the compounds obtained from the literature search in this analysis, including those for which no specific point of interaction in the NF-κB pathway has been suggested, in order to investigate if this step (or a similar first step in the drug discovery process) could also be automated.

**Figure 2:**
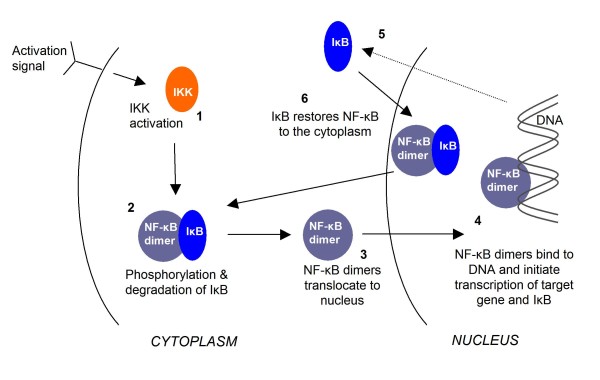
**Simplified NF-κB Pathway**. In unstimulated cells inactive dimers of NF-κB are located in the cytoplasm bound to IκB proteins preventing NF-κB from translocating into the nucleus. Activation of the inhibitor κB kinases (IKK) by NF-κB-activating stimuli (**1**) allows phosphorylation of IκB and NF-κB protein. Phosphorylation of IκB leads to its ubiquitination and degradation and therefore dissociation from the NF-κB dimers (**2**). The free dimers can then translocate into the nucleus (**3**) and regulate target gene transcription (**4**). IκB is a transcriptional target for NF-κB (**5 **and **6**), creating a negative feedback loop.

A major issue in analysing such a diverse system is pooling together all the information available. The available literature for the NF-κB signalling system is an extremely underused resource. This is primarily due to the complexity of comparing data generated using different cell types and stimuli, and the changes in data quality and methodologies over time. Another issue is that the reported effect of a compound is not necessarily indicative of the actual interaction of a compound. For example, if a compound is found to inhibit DNA binding in an electrophoretic mobility shift assay using nuclear extracts it is possible that the actual interaction of this compound could be anywhere upstream of this process[18], as indicated in the schematic diagram of the NF-κB pathway (Figure 2.). In this paper we have assumed that the interactions stated in the literature are correct, but if for example, a compound is reported as interacting at point **3 **in Figure 2, it is possible that its actual effect occurs at any point from **1 **to **3**. In addition, it is entirely possible that molecular interactions may occur at multiple points in the pathway. (For instance, this might be the case with molecules with a reactive oxygen species (ROS) interaction[19]).

## Results

### Method

**Figure 3:**
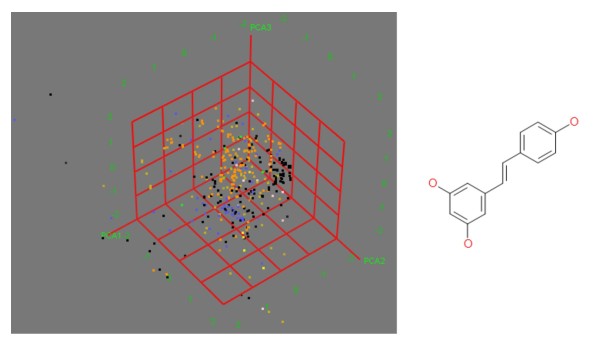
**Distribution of Compounds in a Representation of Chemical Space**. The three principle components were calculated from 184 2D descriptors in MOE[21] and describe 51.1% of the variance. Type of interaction: orange = IKK inhibitors; pink = ROS interactions; blue = DNA interaction; green = inhibits translocation; yellow = increases IκB degradation & phosphorylation; yellow = decreases IκB degradation & phosphorylation; black = unknown. The compounds with unknown interactions in the area A all come from series of compounds based on Resveratrol[25] (bottom).

A set of 460 small molecules that interact with the NF-κB pathway were obtained from the literature. This involved an extensive literature search with additional searches for chemical structures as many of the biological references only refer to compounds that interact with the NF-κB pathway by name (not necessarily the IUPAC name). Structures, SMILES and references for each compound can be found in Additional Files 1. Chiral information has been included where known, but this data was not available for all the compounds (and also the reason why only topological descriptors have been used). The compounds in the set vary greatly in terms of size and functional groups present. Figure 3 shows the distribution of the compounds in a representation of chemical space.

For 297 compounds the type of interaction within the pathway was also taken from the literature. The interactions were defined as: interacting via a ROS mechanism (this can be at any point in the pathway); inhibiting IKK (at point **1 **in Figure 2); inhibiting degradation/phosphorylation of IκB (point **2 **in Figure 2); increasing degradation/phosphorylation of IκB (point **2**); inhibiting translocation (point **3**); and interfering with DNA binding (point **4**). Four compounds had more than one interaction (see Additional File 1 for structures and references).

From this initial compound set of 460 inhibitors, five different training and test set combinations were randomly created. Previous work has shown that different combinations of training and test sets taken from the same data can produce different results[20]. Although this was with respect to a quantitative structure-activity relationship model, the same methodology was applied here. The reason for following this procedure was to identify whether randomly created clusters were able to classify compounds correctly. If only one of the training sets created clusters that classified compounds correctly, we could assume that this was more likely to be due to chance than if all five training sets created clusters that could correctly classify the compounds. A test set was chosen by randomly selecting 60 compounds with known interactions, ensuring that there was at least one compound with each type of interaction. The remainder of compounds were used to form the training set. From this point onward, each of these will be referred to as *dataset 1, dataset 2 *etc., and collectively as *the datasets*. Table 1 shows the number of compounds with a specific interaction in each training and test set of the datasets. Compounds with more than one interaction are included in the training sets of datasets one and four and test sets of datasets two, three and five.

**Table 1:** Compounds in the Various Training and Test Sets

Dataset	ROS interaction	Inhibits translocation	Interfering with DNA binding	Inhibits IKK activation	Inhibits IκB degradation or phosphorylation	Activates IκB degradation or phosphorylation
Training set 1	12	5	48	104	69	3
Test set 1	2	2	12	34	7	3

Training set 2	11	5	52	111	57	4
Test set 2	3	2	8	27	19	2

Training set 3	11	5	53	107	60	4
Test set 3	3	2	7	31	16	2

Training set 4	11	6	48	112	61	3
Test set 4	3	1	12	26	15	3

Training set 5	11	6	48	108	62	5
Test set 5	3	1	12	30	14	1

Table showing the various number of compounds with a specific function for each training and test set of each dataset (training and test set 1 form dataset 1 and so on).

Each training set was clustered using Pipeline Pilot[16] with the following descriptors: Extended Connectivity Fingerprints with a path length of 4 atoms (ECFP4), Property descriptors (AlogP, molecular weight, number of hydrogen bond acceptors, number of hydrogen bond donors, number of atoms, number of rotatable bonds, number of rings, and number of aromatic rings), ECFP4 with Property descriptors, BCUT (descriptors obtained from the eigenvalues of the adjacency matrix, weighting the diagonal elements with atom weights), GCUT (obtained from the eigenvalues of a modified graph distance adjacency matrix), BCUT with GCUT, and GCUT with Property descriptors. The BCUT and GCUT descriptors were calculated using MOE[21]. Clustering was based on maximal dissimilarity partitioning with the clusters derived by imposing a distance threshold between a molecule and its cluster representative. As the clustering algorithm in Pipeline Pilot is dependent upon a *seed *compound, five different seeds were chosen for each descriptor and dataset combination (i.e. there were five different sets of clusters for each descriptor of each dataset giving a total of 25 different sets of clusters for each descriptor).

The number of clusters used in the clustering was chosen by using the following method[22]: first the training set compounds were clustered into a set number of clusters (*n*), which varied from one to 200 (which would give an average of 2 compounds per cluster). For each *n*, the average self-similarity (*avg-s*) of the clusters was calculated. The value of *n *was chosen so that the biggest decrease in *avg-s *was seen between (*n-1*) and *n *clusters.

The clusters were analysed to see if the compounds they contained had predominantly one type of interaction. The interactions used to define the predominance of a cluster are as given above. Validation of the accuracy of the clustering procedure was performed by finding the most similar cluster to the compounds in a test set (i.e. the compounds in the test sets were used as query compounds) in turn and assigning the predominant interaction of the nearest cluster to the test compound. The nearest cluster was found in one of the following ways:

1. The cluster which had the most similar compound;

2. The cluster which had the most similar cluster centre;

3. The cluster with the highest average similarity;

4. Repeat considering only clusters with a minimum of 1 (i.e. singletons), 2, 3, 4 or 5 compounds.

The compounds with unidentified points of interaction in the pathway were included in the training sets used in the clustering analysis in order to investigate how their inclusion affected the ability of using this technique to predict the interactions of the query compounds in the test sets.

### Clustering

**Figure 4:**
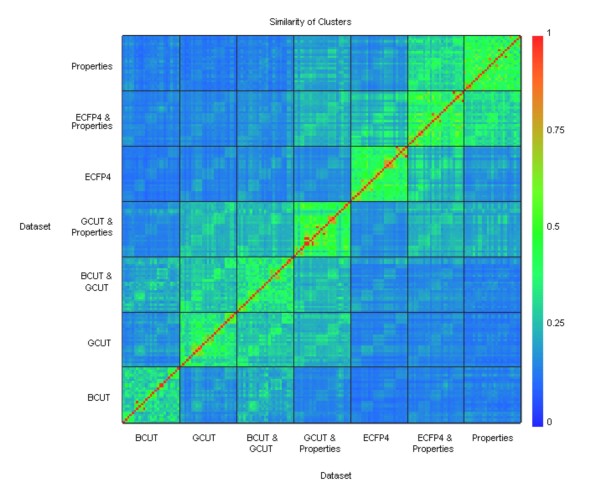
**Similarity of Cluster Sets Using Different Descriptors**. Heat map showing the similarity of the clusters between the datasets as measured using the Tanimoto coefficient (c/(a+b-c) where a = number of pairs of compounds clustered together in the first set of clusters; b = number of pairs of compounds clustered together in the second set of clusters; c = number of pairs of compounds clustered together in both sets of clusters).

Surprisingly, the number of clusters chosen by all the combinations of descriptors and datasets was 135, as this gave the largest decrease in the *avg-s*. Figure 4 shows a heat map representation of the similarity between the clusters for all the datasets. The similarity was measured by comparing which pairs of compounds were clustered together in each of the sets of clusters. The figure shows that the compounds in the clusters vary between the datasets. There is less variance within the datasets of a single descriptor than with those of other descriptors. This is to be expected as datasets of the same descriptor will be partitioned in a similar way. Ignoring singletons, the number of clusters varies from 61 to 85 for the datasets.

Each dataset was then analysed to see how many of the clusters contain compounds with a predominant interaction. The *levels *of predominance used in this analysis were 50%, 66%, and 75%, and considered clusters with a minimum size of 1 (singletons), 2, 3, 4 or 5 compounds. For example, the datasets were analysed to see how many clusters have at least 50% of their members with the same interaction.

**Figure 5:**
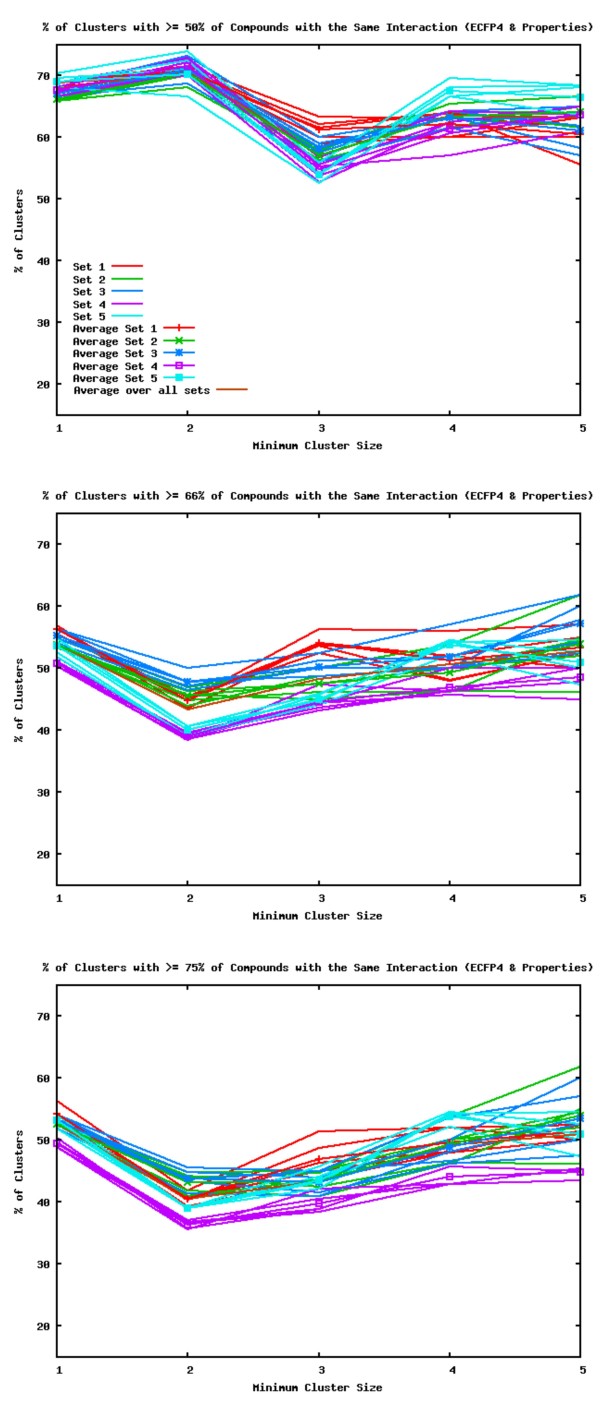
**Proportion of Compounds in Clusters with the Same Interaction (Ecfp4 & Property Descriptors)**. Percentage of clusters with at least 50% (top), 66% (middle), or 75% (bottom) of compounds that have the same interaction using ECFP4 with Property descriptors.

Figure 5 shows the success of the clustering at producing clusters with a predominant interaction using ECFP4 with Property descriptors. The clusters for dataset 5 are shown in Additional File 2. The figure shows that no matter what the minimum size of the clusters taken into consideration, more than half contain over 50% of compounds with the same interaction. Generally 50-70% of clusters contained over 50% of compounds with the same interaction. Of clusters containing 66% or 75% of compounds with the same interaction, dataset 4 performed the worst at 35%, while the other datasets gave 40-60% of clusters having the same interaction. Combining Property descriptors with ECFP4 descriptors may improve the clustering by making the descriptor more specific, e.g. with the Property component compounds of a similar size and a similar number of rings have a higher similarity score to a query. Below we look at the clusters created for a dataset using these descriptors in more detail.

**Figure 6:**
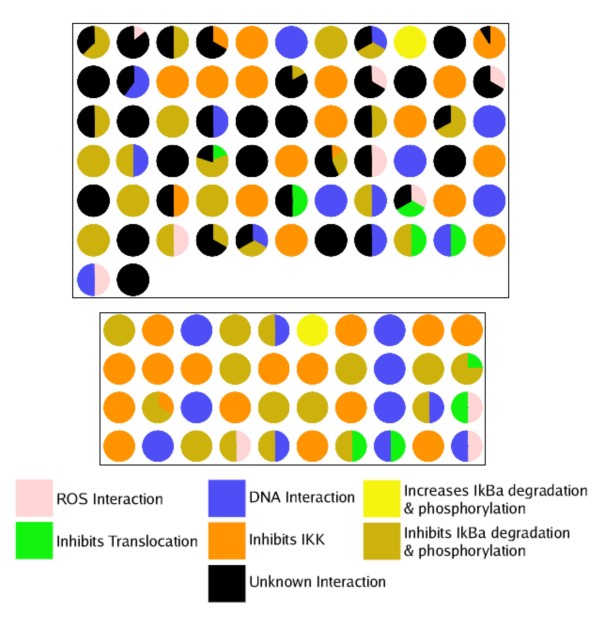
**Pie Chart Representation of Clusters and their Compounds' Interactions**. Pie charts showing the interaction of the compounds within each cluster with at least two compounds. Top: including compounds for which the interaction in the pathway is unknown; Bottom: omitting compounds for which the interaction in the pathway is unknown.

Figure 6 shows pie chart representations of the clusters (minimum size of two compounds) according to the interaction of its member compounds, either including or omitting compounds with unknown interactions. The clusters are those created using ECFP4 with Property descriptors as this gives the best predictions for the compounds not involved in the clustering procedure. The compounds in both sets of clusters can be found in Additional File 3. Taking into account the compounds with unknown interactions, there are 68 clusters with at least two compounds. In 45 clusters 50% or more of its members have the same interaction. 28 clusters have 50% or more members with an unknown interaction (6 have 50% of its members with the same interaction, and 50% with unknown interactions). Omitting the compounds with unknown interactions gives 40 clusters with at least two compounds. All of these have at least 50% of its members having the same interaction. Thirty clusters have 100% of their members with the same interaction, one has 75%, one 66.6%, and the rest (8) have 50% of their members interacting at the same point in the pathway. The results show that clustering can be used to create clusters of compounds with the same interaction. The results shown here are very promising - two thirds of the clusters have the majority of compounds (>50%) with the same interaction; this rises to all the clusters having a majority of compounds with the same interaction when the compounds with unknown interactions are removed from the analysis.

**Figure 7:**
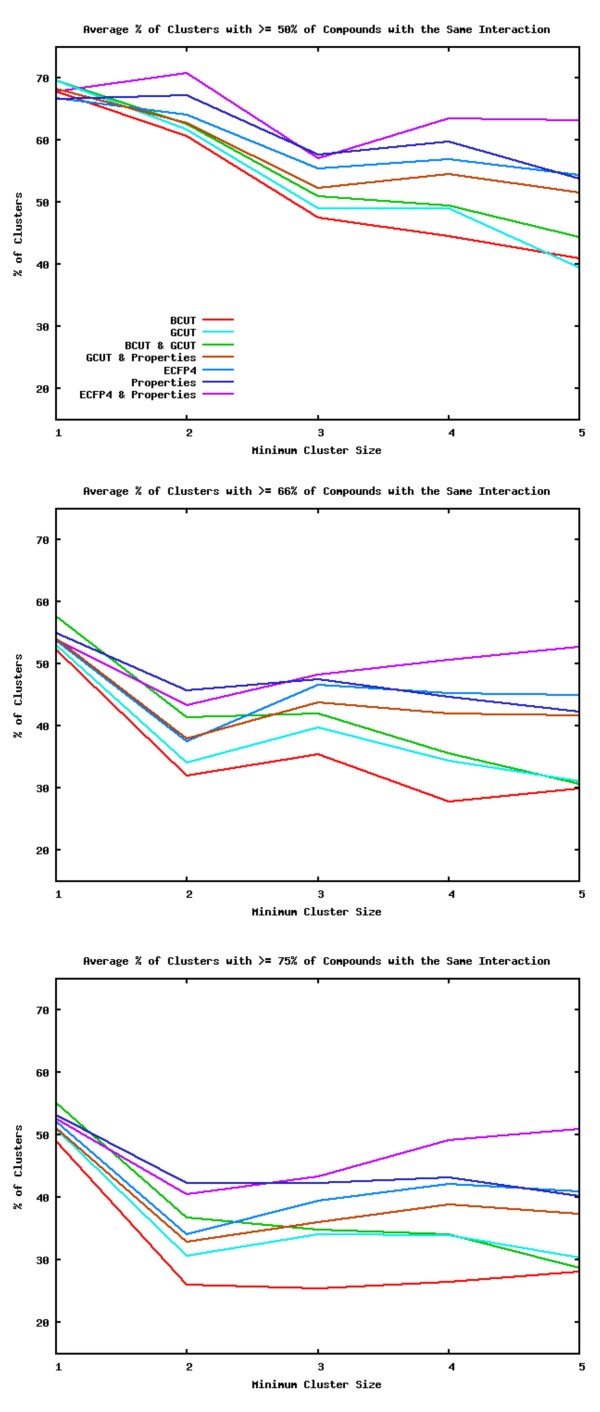
**Average Proportion of Compounds with the Same Interactions in a Cluster for all Descriptors & Datasets**. Percentage of clusters with at least 50% (top), 66% (middle), or 75% (bottom) of compounds that have the same interaction averaged over all the datasets for each descriptor set.

ECFP4 with Property descriptors were the most successful for creating clusters which have a predominant interaction. Figure 7 shows the average classifications for the datasets using each of the descriptors. The order of performance of the descriptors is ECFP4 with Property descriptors > Property descriptors > ECFP4 > GCUT with Property Descriptors > BCUT with GCUT > GCUT > BCUT.

These results show that some of the descriptors and clustering levels used are able to classify compounds into clusters that have predominantly one type of interaction. Next, we look at whether the clusters can be used to identify the interactions of compounds in the test sets.

### Predicting Interactions

**Figure 8:**
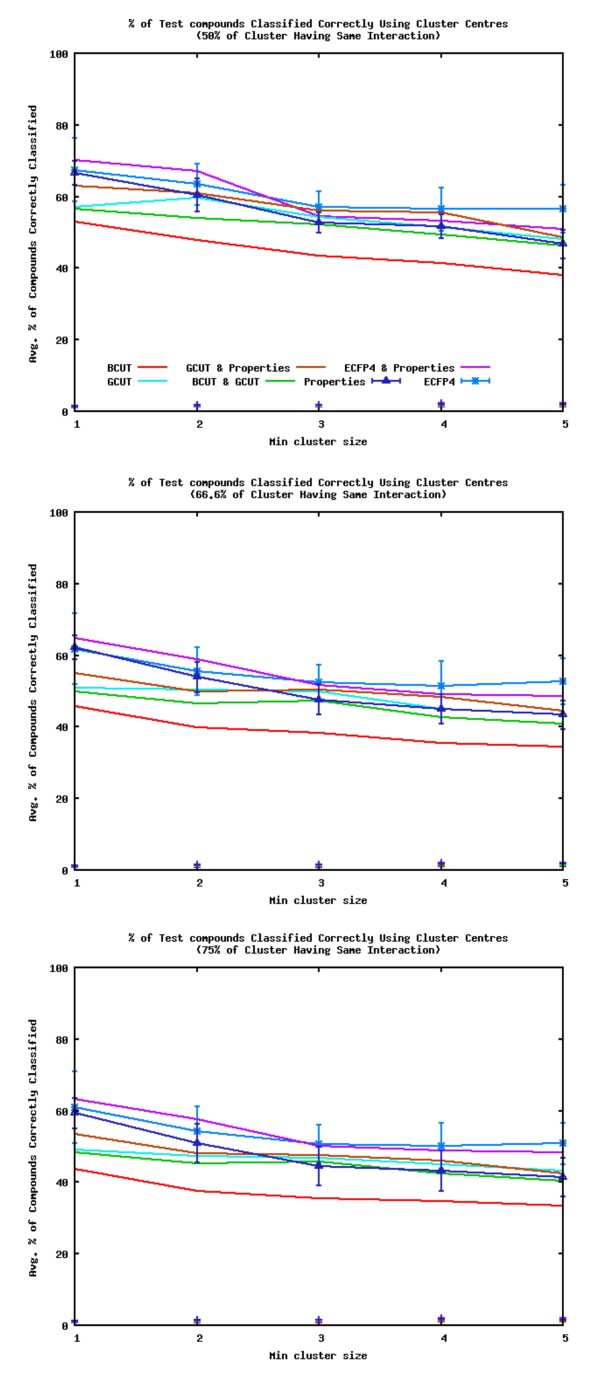
**Percentage of Correctly Identified Interactions (Averaged Over all the Datasets)**. Average percentage of query compounds with interactions correctly identified by the most similar clusters (identified by using the most similar cluster centre; points show interactions assigned at random).

Figure 8 shows the average percentage of query compound interactions correctly identified for the datasets using each of the descriptors (the clusters include compounds for which the pathway interaction is unknown). A query compound is deemed to be "correctly identified" if its point of interaction matches that of 50%, 66.6% or 75% of the other members of the most similar cluster. The most similar cluster is identified using the methods described earlier, i.e. the similarity to the cluster centre, the most similar compounds' cluster or the average similarity to compounds in a cluster. The figure shows the predictions when the similarity to the cluster centre is used to determine the most similar cluster. The average standard deviations over all the datasets are shown for two of the datasets (the other descriptors show similar standard deviations). The percentage of compounds in the test sets classified correctly by assigning clusters at random are also shown by points. The random assignments have < 2% of the interactions of the query compounds correctly identified. In all cases, the interactions of the compounds predicted by finding the nearest cluster using similarity searching techniques are far superior to those by assigning the nearest cluster at random.

**Figure 9:**
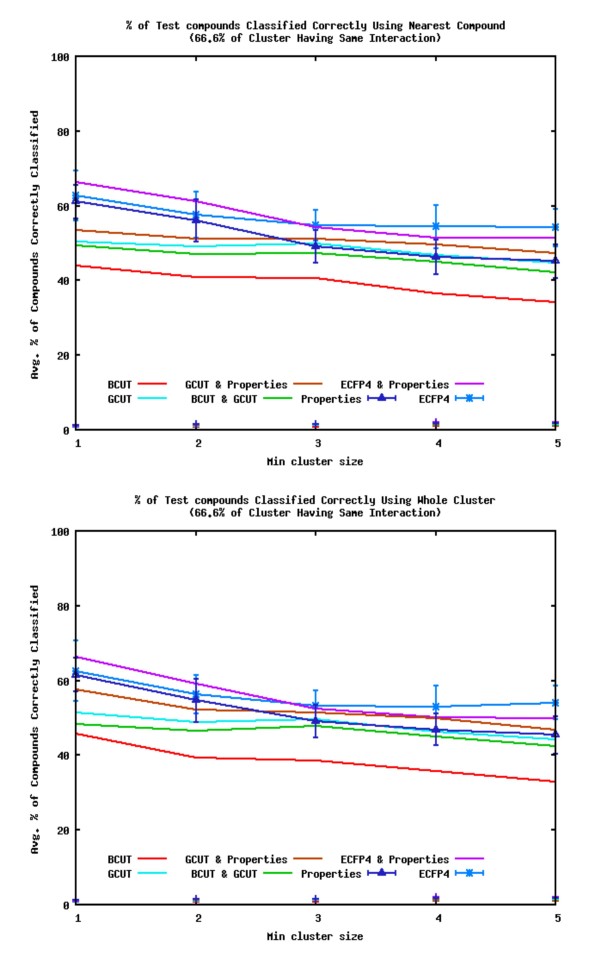
**Percentage of Correctly Identified Interactions using the Most Similar Compound to Identify the most Similar Cluster**. Average percentage of query compounds with interactions correctly identified by the most similar clusters using the most similar compound (top) or the average similarity of a cluster (bottom) to identify the most similar cluster. Interactions are assigned to query compounds if a cluster has at least 66.6% of its members having the same interaction.

Once again the ECFP4 with Property descriptors are the best performing descriptors. The order of the descriptors for their ability to identify the interaction of a compound according to the most similar cluster is slightly different to that for their ability to cluster compounds according to their interactions. The Property descriptors are worse at identifying interactions than classifying compounds, whereas the GCUT descriptors are better. The order for predicting interactions is ECFP4 with Property descriptors ≈ ECFP4 > GCUT with Property descriptors > GCUT ≈ Property descriptors > BCUT with GCUT > BCUT. When the smaller clusters are taken into account (those with two or less compounds) the ECFP4 with Property descriptors are better than ECFP4 descriptors, and the Property descriptors are better than the GCUT descriptors, but these are both reversed when only the larger clusters (with three or more compounds) are considered. If the percentage of clusters having the same interaction used in the identifications is 50%, the GCUT descriptors perform better than the ECFP4 with Property descriptors when only the larger clusters are considered. If the cut off is 66.6% or 75% ECFP4 with Property descriptors are better than the GCUT descriptors. The BCUT with GCUT descriptors are the second worse descriptors, but when the larger clusters are only considered for the identifications they have a similar performance to the Property descriptors. As the size of the minimum cluster allowed to be used in the analysis is increased, the performance of the descriptors decreases. Figure 9 shows the corresponding plots to the middle plot of figure 8 (clusters with 66.6% of their compounds having the same interaction are used to make predictions) when the most similar compound, or the average similarity to compounds in a cluster, is used to find the most similar cluster. There is little difference to the results when the method of how the nearest cluster to the query compound is chosen, or if the majority of compounds in a cluster used to assign the interaction of a query compound is set to 50%, 66.6% or 75%.

**Figure 10:**
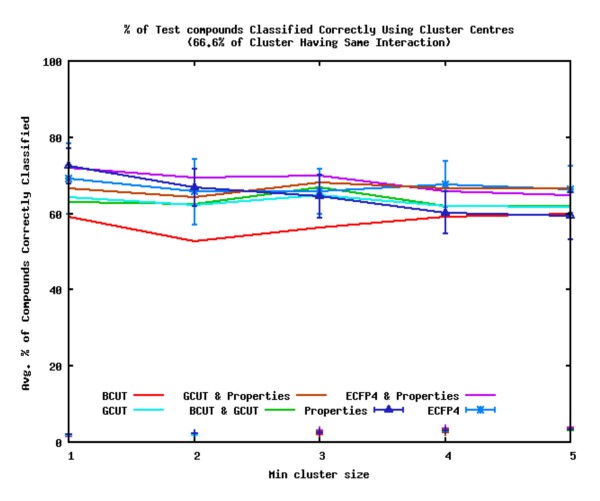
**Percentage of Correctly Identified Interactions using the most Similar Cluster Centre to Identify the most Similar Cluster**. Average percentage of query compounds with interactions correctly identified by the most similar clusters using the most similar cluster centre to identify the most similar cluster. Interactions are assigned to query compounds if a cluster has at least 66.6% of its members having the same interaction; only compounds with known interactions are considered in the analysis of the clusters.

If the compounds with unknown interactions are removed from the analysis, the quality of the predictions improves. Figure 10 shows the average percentage of query compounds' interactions correctly identified for the datasets using each of the descriptors in this case. The plot shows the results when clusters with at least 66.6% of their members having the same interaction are used. The performance of the descriptors increases by up to 20%. The performance of the descriptors is similar to that seen when the compounds with unknown interactions are included, with two exceptions: the GCUT with Property descriptors perform much better and are the second best descriptors, and the Property descriptors are the second worst descriptors when the clusters with at least three compounds are considered. The order of performance of the descriptors is now ECFP4 with Property descriptors > ECFP4 ≈ GCUT with Property descriptors > GCUT ≈ Property descriptors ≈ BCUT with GCUT > BCUT. Only the performance of the Property descriptors consistently decreases as the minimum size of clusters used in the analysis increases. After a drop in performance when the minimum cluster size used increases from one to two, the BCUT descriptors' performance increases as the minimum cluster size increases.

As before, varying the percentage of compounds in a cluster that must have the same interaction for that interaction to be assigned to a query compound has little effect on the order of performance of the descriptors, although there is a slight drop in the number of correctly identified interactions. Similarly, no difference is seen whether the most similar cluster is calculated using the compound of the most similar cluster, the most similar cluster centre, or the cluster with the highest average cluster.

### Similarity Threshold

**Figure 11:**
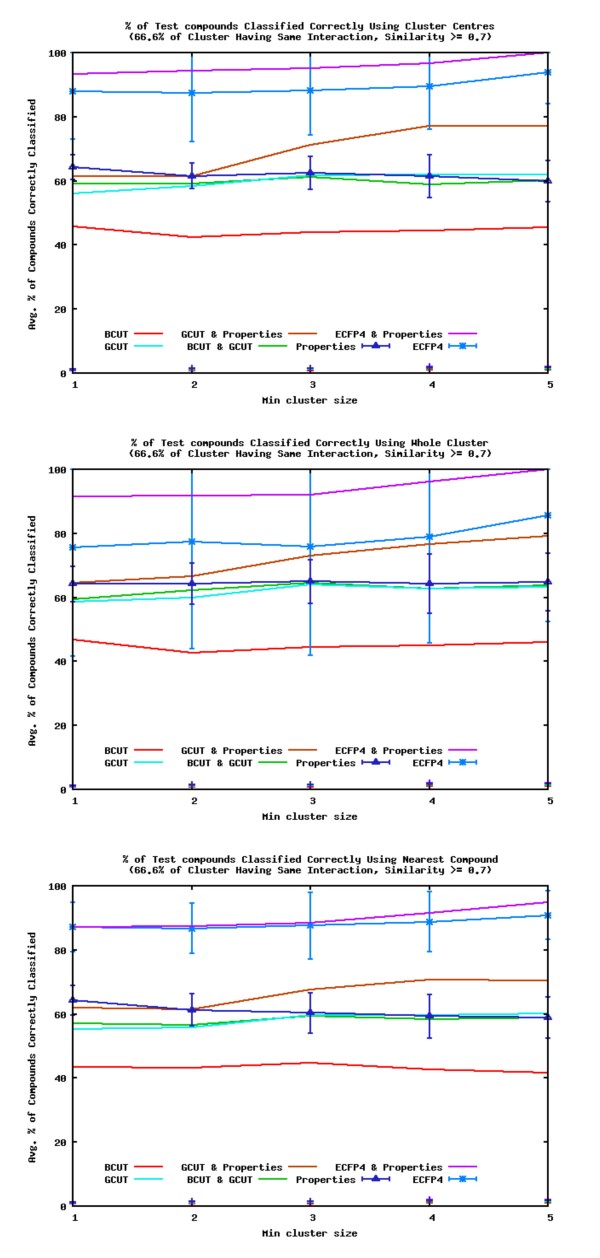
**Percentage of Correctly Identified Interactions when a Similarity Threshold is Applied in Finding the most Similar Cluster**. Average percentage of query compounds with interactions correctly identified by the most similar clusters with a similarity > = 0.7.

If the pathway interaction of a query compound is only predicted if the similarity of a query to a cluster is above a certain threshold there is an improvement in the number of interactions correctly identified. Figure 11 shows the percentage of query compounds' interactions correctly identified when the similarity threshold is set at 0.7 and interactions are assigned if 66.6% or greater of the most similar cluster have the same interaction. Table 2 shows the average number of queries that have a cluster with a similarity > = 0.7. All the descriptors show an improvement, with the ECFP4 and ECFP4 with Property descriptors showing the biggest improvement, however these descriptors only make predictions for less than a third of the query compounds. When only the larger clusters are taken into account, the GCUT with Property descriptors also show a significant increase in performance. The best predictions are made if the cluster centre or the average similarity to a cluster is used to calculate the most similar cluster. This is of no real surprise as the search space to the query compounds is now limited to a small area. According to the similarity property principle[14] similar compounds have similar properties, and as the searches are limited to compounds which are more structurally similar than before, we should obtain compounds with similar properties, which would be likely to have similar interactions, as is the case here. As previously stated there are flaws to the principle[15], but as a general rule it suffices, as shown by the work presented here.

**Table 2:** Average Number of Queries with A Similarity > = 0.7

Descriptor	Minimum Cluster Size				
	1	2	3	4	5
BCUT	54.32	51.68	48.08	45.12	40
GCUT	47.72	42.96	38.52	31.16	26.36
BCUT with GCUT	47.44	42.92	39.04	31.92	29.64
GCUT with Properties	42.72	38.56	30.76	24.64	19.52
ECFP4	13.24	12.08	10.12	9	7.96

ECFP4 with Properties	19.28	17.12	14.92	13.08	11.68
Properties	53.68	50.84	42.96	37.48	33.52

Table showing the average number of query compounds (out of 60) that have a similarity > = 0.7 to the most similar clusters

## Discussion

The drawback to this technique is that the identifications of the point of interactions are limited to compounds which are similar to those used in the initial clustering analysis. If a novel compound that is distinct in structure to the known compounds is found to interact with the pathway, the technique used here may not be sufficient to identify the point of interaction. Incorporating techniques used in scaffold-hopping, such as using reduced graphs, may help to overcome such limitations[23]. Representing molecules as a set of connected features (e.g. an aromatic ring system or an aliphatic link joining two other features together) and using these representations in a search would allow molecules with the same connections of features to be retrieved which would be less structurally similar than the work presented here whilst (hopefully) having the same functionality, allowing for more diverse molecules with similar interactions to be found. Other methods may include creating pharmacophores from molecules with the same interactions and finding compounds which fit the pharmacophore.

## Conclusion

In this analysis we have shown that it is possible to use noisy data obtained from the literature to link together chemoinformatics and network biology, specifically a cellular pathway network. The clusters produced from such data have been shown to be fairly robust, with the information gained from clustering able to help us to decide on the mechanism of action for compounds that are known to interact somewhere in the NF-κB pathway, and could be used to help infer which (and where in the pathway) other untested compounds interact. Here, ECFP4 and ECFP4 with Property descriptors have been shown to be the best at producing clusters which can be used to identify the interactions of an external set of compounds. One interesting feature would be if the techniques used here would be able to find compounds which can alter the timings, and hence the function, of the system[24]. The results presented also show the general applicability of the similar property principle.

## Authors' contributions

YP and CH searched the literature for compounds that were shown to interact with the NF-κB pathway. YP carried out the computational analysis of the compounds and wrote the manuscript. MW, CH and DBK helped draft the manuscript. DBK and MW directed the project. All authors read and approved the final manuscript.

## Supplementary Material

Additional file 1 **List of Compounds that interact with NF-kB**. Click here for file

Additional file 2 **Compounds clustered using ECFP_4 and Property Descriptors**. Click here for file

Additional file 3 **Clusters of Compounds Shown in Figure **6. Click here for file
