# Supplementary material for: Predicting the points of interaction of small molecules in the NF-κB pathway
Source: BMC Syst Biol. 2011 Feb 22;5:32. doi: 10.1186/1752-0509-5-32 (PMC3050742; doi:10.1186/1752-0509-5-32)
Supplement: Additional file 1 — List of Compounds that interact with NF-kB. [file 1752-0509-5-32-S1.ZIP › Additional Files 1/Additional Files 1.htm]

 

|  |  |  |  |  |  |  |  |  |  |  |  |  |
| --- | --- | --- | --- | --- | --- | --- | --- | --- | --- | --- | --- | --- |
| Molecule | Dataset\_1 | Dataset\_2 | Dataset\_3 | Dataset\_4 | Dataset\_5 | Ref | inhibits\_IKK\_activation | inhibits\_IkB\_degradation\_phosphorylation | activates\_IkB\_phosphorylation\_degradation | DNA\_interaction | inhibits\_translocation | ROS\_interaction |
|  | Training Set | Training Set | Training Set | Training Set | Training Set | Aikawa et al. (2002) Inflamm. res. (51) 188-194 | 0 | 0 | 0 | 0 | 0 | 0 |
|  | Test Set | Training Set | Training Set | Training Set | Training Set | Arbault et al. (1997) Biomed. Pharmacother. (51) 430-438 Singh & Aggarwal (1995) J Bio Chem (270) 10631-10639 | 0 | 1 | 0 | 0 | 0 | 0 |
|  | Training Set | Training Set | Training Set | Training Set | Training Set | Auphan et al (1995) Science (270) 286-290 Hass et al. (1992) Biochem. Pharm. (44) 1569-1576 Wang et al. (2006) Toxicol. App. Pharm. (214) 263-269 | 0 | 0 | 0 | 0 | 0 | 0 |
|  | Training Set | Training Set | Training Set | Training Set | Training Set | Bauman et al. (2002) J Bio Chem (277) 44791-44800 | 0 | 0 | 0 | 0 | 0 | 0 |
|  | Training Set | Training Set | Training Set | Training Set | Training Set | Bayon et al. (2003) Mol. Cell. Biol. (23) 1061 | 1 | 0 | 0 | 0 | 0 | 0 |
|  | Training Set | Training Set | Training Set | Test Set | Training Set | Behl, C. (2000) J. Neural Tansm. (107) 393-405 | 0 | 0 | 0 | 0 | 0 | 1 |
|  | Training Set | Training Set | Training Set | Test Set | Test Set | Bellas et al. (1997) Am J Pathol (151) 891-896 Fenteany & Schreiber (1998) J. Bio. Chem. (273) 8584 | 0 | 1 | 0 | 0 | 0 | 0 |
|  | Training Set | Training Set | Training Set | Training Set | Training Set | Berry et al, (2002) Experimental Cell Research (272) 176-184 | 0 | 0 | 1 | 0 | 0 | 0 |
|  | Test Set | Training Set | Test Set | Test Set | Training Set | Berry et al, (2002) Experimental Cell Research (272) 176-184 | 0 | 0 | 1 | 0 | 0 | 0 |
|  | Training Set | Test Set | Training Set | Test Set | Training Set | Berry et al, (2002) Experimental Cell Research (272) 176-184 | 0 | 0 | 1 | 0 | 0 | 0 |
|  | Test Set | Training Set | Training Set | Training Set | Training Set | Berry et al, (2002) Experimental Cell Research (272) 176-184 | 0 | 0 | 1 | 0 | 0 | 0 |
|  | Test Set | Test Set | Test Set | Test Set | Test Set | Berry et al, (2002) Experimental Cell Research (272) 176-184 | 0 | 0 | 1 | 0 | 0 | 0 |
|  | Training Set | Training Set | Training Set | Training Set | Training Set | Bork et al. (1999) Planta Med. (65) 297 | 0 | 0 | 0 | 0 | 0 | 0 |
|  | Training Set | Training Set | Training Set | Training Set | Training Set | Bratt et al (2000) Clin Exp Immunol (120) 79-84 | 0 | 0 | 0 | 0 | 0 | 0 |
|  | Test Set | Training Set | Training Set | Training Set | Test Set | Brennan & O'Neill (1998) Biochem. Phram. (55) 965-973 | 0 | 0 | 0 | 1 | 0 | 0 |
|  | Training Set | Training Set | Training Set | Training Set | Training Set | Bustos et al. (1998) J. Am. Coll. Cardiol. (32) 2057-5064 | 0 | 0 | 0 | 0 | 0 | 0 |
|  | Training Set | Training Set | Training Set | Training Set | Training Set | Carballo et al (1999) J Bio Chem (274) 93-100 | 0 | 0 | 0 | 0 | 0 | 1 |
|  | Training Set | Training Set | Test Set | Training Set | Test Set | Cernauda-Morollon et al. (2001) Am. Soc. Biochem. Mol. Bio. (276) 35530-35536 | 0 | 0 | 0 | 1 | 0 | 0 |
|  | Training Set | Training Set | Training Set | Training Set | Training Set | Chanani et al. (2002) Circulation (106) I284-I285 | 0 | 0 | 0 | 0 | 0 | 0 |
|  | Training Set | Training Set | Training Set | Test Set | Training Set | Chaturverdi et al (1997) J Bio Chem (272) 30129-30134 | 0 | 1 | 0 | 0 | 0 | 0 |
|  | Training Set | Test Set | Test Set | Test Set | Training Set | Chen & Cederbaum (1997) Am. Soc. Pharma. & Exp. Therap. (52) 648-657 | 0 | 0 | 0 | 0 | 0 | 1 |
|  | Training Set | Training Set | Test Set | Training Set | Training Set | Chen (2002) J. Cardio. Pharmacol. (39) 332-339 | 0 | 1 | 0 | 0 | 0 | 1 |
|  | Training Set | Test Set | Test Set | Training Set | Training Set | Chen et al (2002) Br. J. Pharm. (135) 37-47 | 0 | 0 | 0 | 0 | 1 | 0 |
|  | Training Set | Training Set | Training Set | Training Set | Training Set | Chiarugi (2002) Br. J. Pharamcol. (137) 761-770 | 0 | 0 | 0 | 0 | 0 | 0 |
|  | Training Set | Training Set | Training Set | Training Set | Test Set | Cho et al (2002) Int. Immunopharm (2) 105-116 Calzado et al. (2007) Curr. Med. Chem. (14) 367-376 | 0 | 1 | 0 | 0 | 0 | 0 |
|  | Training Set | Training Set | Training Set | Test Set | Training Set | Cho et al (2002) Int. Immunopharm (2) 105-116 Calzado et al. (2007) Curr. Med. Chem. (14) 367-376 | 0 | 1 | 0 | 0 | 0 | 0 |
|  | Training Set | Training Set | Training Set | Training Set | Training Set | Cho et al. (1998) Biochem & Biophys Res. Comm. (253) 104-108 | 0 | 1 | 0 | 0 | 0 | 0 |
|  | Training Set | Training Set | Training Set | Training Set | Training Set | Chun et al (2002)\* Environ. Pathol. Toxicol. Oncol. (21) 131 | 0 | 0 | 0 | 0 | 0 | 0 |
|  | Training Set | Training Set | Training Set | Training Set | Training Set | Chun et al (2002)\* Environ. Pathol. Toxicol. Oncol. (21) 131 | 0 | 0 | 0 | 0 | 0 | 0 |
|  | Training Set | Test Set | Test Set | Training Set | Test Set | D'Acquisto et al. (2000) Biochem J. (346) 793-798 | 0 | 1 | 0 | 0 | 0 | 0 |
|  | Test Set | Test Set | Test Set | Training Set | Test Set | Dhanalakshmi et al (2002) oncogen (21) 1759-1767 | 1 | 0 | 0 | 0 | 0 | 0 |
|  | Test Set | Training Set | Training Set | Training Set | Training Set | Erkel et al. (1996) Biocem & Biophys Res Comm (226) 214-221 Umezawa et al. (2000) Anti-Can Drug Des (15) 239-244 | 0 | 1 | 0 | 0 | 0 | 0 |
|  | Training Set | Training Set | Training Set | Training Set | Training Set | Erlandsson et al. (2002) Biochem Pharma (64) 487-495 | 0 | 0 | 0 | 0 | 0 | 0 |
|  | Training Set | Training Set | Training Set | Training Set | Training Set | Erlandsson et al. (2002) Biochem Pharma (64) 487-495 | 0 | 0 | 0 | 0 | 0 | 0 |
|  | Training Set | Training Set | Training Set | Training Set | Training Set | Ferry et al (2002) Carcinogenesis (23) 2031-2041 | 0 | 0 | 0 | 0 | 0 | 0 |
|  | Training Set | Training Set | Training Set | Training Set | Training Set | Gao et al. (2007) Bioorg. Med. Chem. Lett. (17) 4338-4312 Gao et al. (2004) Cancer Research (64) 278-688 | 0 | 0 | 0 | 0 | 0 | 0 |
|  | Training Set | Training Set | Training Set | Training Set | Training Set | Gao et al. (2007) Bioorg. Med. Chem. Lett. (17) 4338-4312 Gao et al. (2004) Cancer Research (64) 278-688 | 0 | 0 | 0 | 0 | 0 | 0 |
|  | Training Set | Training Set | Training Set | Training Set | Training Set | Gao et al. (2007) Bioorg. Med. Chem. Lett. (17) 4338-4312 Gao et al. (2004) Cancer Research (64) 278-688 | 0 | 0 | 0 | 0 | 0 | 0 |
|  | Training Set | Training Set | Training Set | Training Set | Training Set | Gao et al. (2007) Bioorg. Med. Chem. Lett. (17) 4338-4312 Gao et al. (2004) Cancer Research (64) 278-688 | 0 | 0 | 0 | 0 | 0 | 0 |
|  | Training Set | Training Set | Training Set | Training Set | Training Set | Gao et al. (2007) Bioorg. Med. Chem. Lett. (17) 4338-4312 Gao et al. (2004) Cancer Research (64) 278-688 | 0 | 0 | 0 | 0 | 0 | 0 |
|  | Training Set | Training Set | Training Set | Training Set | Training Set | Gao et al. (2007) Bioorg. Med. Chem. Lett. (17) 4338-4312 Gao et al. (2004) Cancer Research (64) 278-688 | 0 | 0 | 0 | 0 | 0 | 0 |
|  | Training Set | Training Set | Training Set | Training Set | Training Set | Gao et al. (2007) Bioorg. Med. Chem. Lett. (17) 4338-4312 Gao et al. (2004) Cancer Research (64) 278-688 | 0 | 0 | 0 | 0 | 0 | 0 |
|  | Training Set | Training Set | Training Set | Training Set | Training Set | Gao et al. (2007) Bioorg. Med. Chem. Lett. (17) 4338-4312 Gao et al. (2004) Cancer Research (64) 278-688 | 0 | 0 | 0 | 0 | 0 | 0 |
|  | Training Set | Training Set | Training Set | Training Set | Training Set | Gao et al. (2007) Bioorg. Med. Chem. Lett. (17) 4338-4312 Gao et al. (2004) Cancer Research (64) 278-688 | 0 | 0 | 0 | 0 | 0 | 0 |
|  | Training Set | Training Set | Training Set | Training Set | Training Set | Gao et al. (2007) Bioorg. Med. Chem. Lett. (17) 4338-4312 Gao et al. (2004) Cancer Research (64) 278-688 | 0 | 0 | 0 | 0 | 0 | 0 |
|  | Training Set | Training Set | Training Set | Training Set | Training Set | Gao et al. (2007) Bioorg. Med. Chem. Lett. (17) 4338-4312 Gao et al. (2004) Cancer Research (64) 278-688 | 0 | 0 | 0 | 0 | 0 | 0 |
|  | Training Set | Training Set | Test Set | Test Set | Training Set | Gehrt et al. (1998) J. antibiotics (51) 455 | 0 | 1 | 0 | 0 | 0 | 0 |
|  | Test Set | Test Set | Training Set | Training Set | Training Set | Geng et al. (1997) Free Rad. Bio. Med. (23) 345-350 | 0 | 0 | 0 | 0 | 0 | 1 |
|  | Test Set | Test Set | Training Set | Training Set | Test Set | Ghosh et al (2003) Blood (101) 2321 | 0 | 1 | 0 | 0 | 0 | 0 |
|  | Training Set | Training Set | Training Set | Training Set | Training Set | Gilad et al. (1998) FASEB Journal (12) 685-693 | 0 | 0 | 0 | 1 | 0 | 0 |
|  | Training Set | Training Set | Training Set | Training Set | Training Set | Haddad et al. (2002) Int. ImmunoPharmacology (2) 1567-1583 | 0 | 1 | 0 | 0 | 0 | 0 |
|  | Training Set | Training Set | Training Set | Training Set | Training Set | Hammes et al. (2003) Nature Med. (9) 294-299 | 0 | 0 | 0 | 0 | 0 | 0 |
|  | Training Set | Training Set | Training Set | Training Set | Training Set | Hernandez-Presa et al. (1998) J. Am. Path. (153) 1825 | 0 | 0 | 0 | 0 | 0 | 0 |
|  | Training Set | Test Set | Training Set | Test Set | Training Set | Hideshima et al. (2001) Cancer Research (61) 3071-3076 Calzado et al (2007) Curr. Med. Chem. (14) 367-376 | 0 | 1 | 0 | 0 | 0 | 0 |
|  | Training Set | Test Set | Training Set | Training Set | Test Set | Hiramoto et al. (1998) J. Immunol. (160) 810-819 | 0 | 0 | 0 | 1 | 0 | 0 |
|  | Training Set | Training Set | Training Set | Training Set | Training Set | Horton et al. (1999) Am. Soc. Biochem. & Mol. Bio. (274) 9200-9206 | 0 | 0 | 0 | 0 | 0 | 0 |
|  | Training Set | Training Set | Training Set | Training Set | Training Set | Horton et al. (1999) Am. Soc. Biochem. & Mol. Bio. (274) 9200-9206 | 0 | 0 | 0 | 0 | 0 | 0 |
|  | Training Set | Training Set | Training Set | Training Set | Training Set | Islam et al (1998) Circulation (98) 2255 | 0 | 0 | 0 | 0 | 0 | 0 |
|  | Training Set | Test Set | Test Set | Training Set | Test Set | Jeong et al (2002) Cytokine (18) 252-259 | 0 | 1 | 0 | 0 | 0 | 0 |
|  | Training Set | Training Set | Test Set | Test Set | Training Set | Jeong et al. (1997) Immunology (92) 267-273 | 0 | 1 | 0 | 0 | 0 | 0 |
|  | Training Set | Test Set | Training Set | Training Set | Training Set | Jeong et al. (1997) Immunology (92) 267-273 | 0 | 1 | 0 | 0 | 0 | 0 |
|  | Training Set | Training Set | Training Set | Training Set | Training Set | Jimenez et al. (2001) J. Pharmacol. Exp. Therap. (299) 753-759 | 0 | 0 | 0 | 0 | 0 | 0 |
|  | Training Set | Training Set | Training Set | Training Set | Training Set | Kang et al (2003) Biochem Pharmacol (65) 457-464 | 0 | 1 | 0 | 0 | 0 | 0 |
|  | Training Set | Training Set | Test Set | Test Set | Test Set | Karin et al (2004) Nat Rev Drug Disc (3) 17 | 1 | 0 | 0 | 0 | 0 | 0 |
|  | Test Set | Training Set | Training Set | Training Set | Training Set | Karin et al (2004) Nat Rev Drug Disc (3) 17 | 1 | 0 | 0 | 0 | 0 | 0 |
|  | Test Set | Training Set | Training Set | Training Set | Training Set | Burke et al (2003) J. Bio. Chem. (278) 1450-1456 Karin et al (2004) Nat Rev Drug Disc (3) 17 Calzado et al (2007) Curr. Med. Chem. (14) 367-376 | 1 | 0 | 0 | 0 | 0 | 0 |
|  | Training Set | Training Set | Training Set | Training Set | Training Set | Karin et al (2004) Nat Rev Drug Disc (3) 17 | 1 | 0 | 0 | 0 | 0 | 0 |
|  | Training Set | Test Set | Training Set | Training Set | Test Set | Karin et al (2004) Nat Rev Drug Disc (3) 17 | 1 | 0 | 0 | 0 | 0 | 0 |
|  | Test Set | Training Set | Training Set | Training Set | Test Set | Karin et al (2004) Nat Rev Drug Disc (3) 17 | 1 | 0 | 0 | 0 | 0 | 0 |
|  | Test Set | Training Set | Training Set | Training Set | Training Set | Karin et al (2004) Nat Rev Drug Disc (3) 17 | 1 | 0 | 0 | 0 | 0 | 0 |
|  | Training Set | Training Set | Training Set | Training Set | Training Set | Karin et al (2004) Nat Rev Drug Disc (3) 17 Calzado et al (2007) Curr. Med. Chem. (14) 367-376 | 1 | 0 | 0 | 0 | 0 | 0 |
|  | Test Set | Training Set | Training Set | Training Set | Training Set | Karin et al (2004) Nat Rev Drug Disc (3) 17 | 1 | 0 | 0 | 0 | 0 | 0 |
|  | Training Set | Training Set | Training Set | Test Set | Test Set | Karin et al (2004) Nat Rev Drug Disc (3) 17 | 1 | 0 | 0 | 0 | 0 | 0 |
|  | Training Set | Test Set | Test Set | Training Set | Training Set | Karin et al (2004) Nat Rev Drug Disc (3) 17 | 0 | 1 | 0 | 0 | 0 | 0 |
|  | Training Set | Training Set | Training Set | Training Set | Training Set | Kishore et al (2003) J. Bio. Chem. (278) 32861-32871 Karin et al (2004) Nat Rev Drug Disc (3) 17 Baxter et al (2004) Bioorg & Med Chem Letts. (14) 2817-2822 Calzado et al (2007) Curr. Med. Chem. (14) 367-376 | 1 | 0 | 0 | 0 | 0 | 0 |
|  | Test Set | Training Set | Training Set | Training Set | Training Set | Karin et al (2004) Nat Rev Drug Disc (3) 17 Calzado et al (2007) Curr. Med. Chem. (14) 367-376 | 1 | 0 | 0 | 0 | 0 | 0 |
|  | Training Set | Test Set | Training Set | Training Set | Test Set | Karin et al (2004) Nat Rev Drug Disc (3) 17 Calzado et al (2007) Curr. Med. Chem. (14) 367-376 | 1 | 0 | 0 | 0 | 0 | 0 |
|  | Training Set | Training Set | Test Set | Training Set | Training Set | Kafoury et al. (2007) Env. Toxicol. 159-168 | 0 | 0 | 0 | 1 | 0 | 0 |
|  | Training Set | Training Set | Training Set | Training Set | Training Set | Kawashima et al. (1998) J. Interferon Cytokine Res. (18) 423 | 0 | 0 | 0 | 0 | 0 | 0 |
|  | Test Set | Training Set | Training Set | Training Set | Test Set | Kazmi et al. (1995) J. Cell. Bio. (57) 299-310 Calzado et al. (2007) Curr. Med. Chem. (14) 367-376 | 0 | 0 | 0 | 0 | 0 | 1 |
|  | Training Set | Test Set | Test Set | Training Set | Training Set | Keifer et al. (2001) J. Bio. Chem. (25) 22382-22387 Aerbajinal et al (2007) Hematopoiesis (110) 2864-2871 | 0 | 1 | 0 | 0 | 0 | 0 |
|  | Training Set | Training Set | Training Set | Training Set | Training Set | Kelly et al. (1994) Infection & Immunity (62) 3122-3128 | 0 | 0 | 0 | 0 | 0 | 0 |
|  | Training Set | Training Set | Training Set | Training Set | Training Set | Kim et al (1999) Biochem. Biophys. Res. Comm. (259) 505-509 | 0 | 0 | 0 | 0 | 0 | 0 |
|  | Test Set | Training Set | Training Set | Training Set | Training Set | Kopp and Ghosh (1994) Science (265) 956-9 Kiss et al. (2004) J. Biochem. Biophys. Methods (61) 229-240 Pierce et al. (1996) J. Immun. (156) 3961-3969 | 0 | 1 | 0 | 0 | 0 | 0 |
|  | Training Set | Training Set | Training Set | Test Set | Training Set | Kopp and Ghosh (1994) Science (265) 956-9 Sakurada et al. (1996) International Immunology (8) 1483-1493 Calzado et al (2007) Curr. Med. Chem. (14) 367-376 Pierce et al. (1996) J. Immun. (156) 3961-3969 | 0 | 1 | 0 | 0 | 0 | 0 |
|  | Training Set | Training Set | Training Set | Test Set | Training Set | Kotake et al. (1998) Biochim. et Biophys. Acta mo. cell. res. (1448) 77-84 | 0 | 0 | 0 | 0 | 0 | 1 |
|  | Training Set | Training Set | Training Set | Test Set | Training Set | Largo et al (2003) OsteoArth. Cartil. (11) 290-298 | 0 | 1 | 0 | 0 | 0 | 0 |
|  | Training Set | Training Set | Training Set | Training Set | Training Set | Le Page et al. (1998) Biochem. Biophys. Res. Comm. (243) 451-457 | 0 | 0 | 0 | 0 | 0 | 0 |
|  | Training Set | Training Set | Training Set | Training Set | Training Set | Le Page et al. (1998) Biochem. Biophys. Res. Comm. (243) 451-457 | 0 | 0 | 0 | 0 | 0 | 0 |
|  | Training Set | Training Set | Training Set | Training Set | Training Set | Lee et al. (2002) J Bio. Chem. (277) 18411-18420 | 0 | 0 | 0 | 1 | 0 | 0 |
|  | Training Set | Training Set | Training Set | Training Set | Training Set | Lee et al. (2007) Cell Biol. Tox. (23) 105-112 | 0 | 1 | 0 | 0 | 0 | 0 |
|  | Training Set | Training Set | Training Set | Training Set | Test Set | Li et al. (1996) Carcinogenesis (17) 2395 - 2309 | 0 | 0 | 0 | 0 | 0 | 1 |
|  | Training Set | Training Set | Training Set | Training Set | Training Set | Li et al. (1996) Biochem & Biophys. Res. Comm. (229) 982-989 | 0 | 0 | 0 | 0 | 0 | 1 |
|  | Training Set | Training Set | Training Set | Training Set | Training Set | Li et al. (2002) Org. Letts. (4) 2367-2370 | 0 | 0 | 0 | 0 | 0 | 0 |
|  | Training Set | Training Set | Training Set | Training Set | Training Set | Lo et al. (2002)\* Carcinogenesis (23) 983 | 0 | 1 | 0 | 0 | 0 | 0 |
|  | Training Set | Training Set | Training Set | Training Set | Training Set | Loop et al. (2002) Anesthesiology (96) 1202-13 | 0 | 1 | 0 | 0 | 0 | 0 |
|  | Training Set | Training Set | Training Set | Training Set | Training Set | Asanuma, J.L. Cadet (1998) Molecular Brain Research, 60, 305-309 | 0 | 0 | 0 | 0 | 0 | 1 |
|  | Training Set | Training Set | Training Set | Training Set | Test Set | Mahon & O'Neill (1995) J Bio Chem (270) 28557-28564 | 0 | 0 | 0 | 1 | 0 | 0 |
|  | Training Set | Training Set | Test Set | Training Set | Test Set | Manna et al. (1999) Biochem Pharmacol (57) 763-774 | 0 | 1 | 0 | 0 | 0 | 0 |
|  | Training Set | Training Set | Training Set | Training Set | Training Set | Masamune et al. (1995) FEBS (367) 205-209 Matsumori et al. (2000) Life Sciences (67) 2513-2519 | 0 | 0 | 0 | 0 | 0 | 1 |
|  | Training Set | Training Set | Training Set | Training Set | Training Set | Matsumori et al (2000) Life Sciences (67) 2655-2661 | 0 | 0 | 0 | 0 | 0 | 0 |
|  | Training Set | Training Set | Training Set | Training Set | Training Set | Miyanahora et al. (2000) Laryngoscope (110) 126 | 0 | 0 | 0 | 0 | 0 | 0 |
|  | Training Set | Training Set | Training Set | Test Set | Training Set | Murata et al (2003) Bioorg. Med. Chem. Lett. (13) 913-918 | 1 | 0 | 0 | 0 | 0 | 0 |
|  | Training Set | Training Set | Training Set | Training Set | Training Set | Musonda & Chipman (1998) Carcinogenesis 1583-1589 Peet & Li (1999) J Bio Chem (274) 32655-32661 Calzado et al (2007) Curr. Med. Chem. (14) 367-376 Natarajan et al (1998) Arch. Biochem & Biophys (352) 59-70 | 1 | 0 | 0 | 0 | 0 | 0 |
|  | Training Set | Test Set | Training Set | Training Set | Test Set | Natarajan et al (1998) Arch. Biochem & Biophys (352) 59-70 | 0 | 1 | 0 | 0 | 0 | 0 |
|  | Training Set | Training Set | Training Set | Test Set | Training Set | Natarajan et al. (1996) Immunology (93) 9090-9095 | 0 | 0 | 0 | 1 | 0 | 0 |
|  | Training Set | Training Set | Training Set | Training Set | Training Set | Natarajan et al. (1996) Immunology (93) 9090-9095 | 0 | 0 | 0 | 1 | 0 | 0 |
|  | Training Set | Training Set | Training Set | Training Set | Training Set | Natarajan et al. (1996) Immunology (93) 9090-9095 | 0 | 0 | 0 | 1 | 0 | 0 |
|  | Test Set | Training Set | Training Set | Training Set | Test Set | Natarajan et al. (1996) Immunology (93) 9090-9095 | 0 | 0 | 0 | 1 | 0 | 0 |
|  | Training Set | Training Set | Training Set | Test Set | Test Set | Natarajan et al. (1996) Immunology (93) 9090-9095 | 0 | 0 | 0 | 1 | 0 | 0 |
|  | Training Set | Training Set | Test Set | Training Set | Training Set | Natarajan et al. (1996) Immunology (93) 9090-9095 | 0 | 0 | 0 | 1 | 0 | 0 |
|  | Training Set | Test Set | Training Set | Training Set | Training Set | Natarajan et al. (1996) Immunology (93) 9090-9095 | 0 | 0 | 0 | 1 | 0 | 0 |
|  | Training Set | Training Set | Training Set | Training Set | Training Set | Oh et al. (2001) Cancer Letts. (174) 17-24 | 0 | 0 | 0 | 0 | 0 | 0 |
|  | Training Set | Training Set | Training Set | Training Set | Training Set | Otsuka et al. (1995) J. Med. Chem. (37) 3264-3270 Otsuka et al. (1997) Bio. Med. Chem. (5) 205-215 | 0 | 0 | 0 | 0 | 0 | 0 |
|  | Training Set | Training Set | Training Set | Training Set | Training Set | Otsuka et al. (1995) J. Med. Chem. (37) 3264-3270 Otsuka et al. (1997) Bio. Med. Chem. (5) 205-215 | 0 | 0 | 0 | 0 | 0 | 0 |
|  | Training Set | Training Set | Training Set | Training Set | Training Set | Page et al. (1999) J. Bio. Chem. (274) 11611-11618 | 0 | 1 | 0 | 0 | 0 | 0 |
|  | Training Set | Training Set | Training Set | Test Set | Training Set | Pande et al. (2003) J. Comp. Mol.-Aid. Des. (17) 825-863 | 0 | 0 | 0 | 1 | 0 | 0 |
|  | Test Set | Training Set | Training Set | Training Set | Test Set | Pande et al. (2003) J. Comp. Mol.-Aid. Des. (17) 825-863 | 0 | 0 | 0 | 1 | 0 | 0 |
|  | Training Set | Training Set | Training Set | Training Set | Training Set | Pande et al. (2003) J. Comp. Mol.-Aid. Des. (17) 825-863 | 0 | 0 | 0 | 1 | 0 | 0 |
|  | Training Set | Training Set | Training Set | Training Set | Training Set | Pande et al. (2003) J. Comp. Mol.-Aid. Des. (17) 825-863 | 0 | 0 | 0 | 1 | 0 | 0 |
|  | Test Set | Training Set | Training Set | Training Set | Training Set | Pande et al. (2003) J. Comp. Mol.-Aid. Des. (17) 825-863 | 0 | 0 | 0 | 1 | 0 | 0 |
|  | Training Set | Training Set | Training Set | Training Set | Training Set | Pande et al. (2003) J. Comp. Mol.-Aid. Des. (17) 825-863 | 0 | 0 | 0 | 1 | 0 | 0 |
|  | Training Set | Training Set | Test Set | Training Set | Training Set | Pande et al. (2003) J. Comp. Mol.-Aid. Des. (17) 825-863 | 0 | 0 | 0 | 1 | 0 | 0 |
|  | Training Set | Training Set | Training Set | Training Set | Training Set | Pande et al. (2003) J. Comp. Mol.-Aid. Des. (17) 825-863 | 0 | 0 | 0 | 1 | 0 | 0 |
|  | Test Set | Training Set | Test Set | Training Set | Training Set | Park et al. (2002) Cell Biol. & Toxicol. (18) 121-130 | 0 | 1 | 0 | 0 | 0 | 0 |
|  | Training Set | Training Set | Test Set | Training Set | Training Set | Peet & Li (1999) J Bio Chem (274) 32655-32661 | 1 | 0 | 0 | 0 | 0 | 0 |
|  | Training Set | Training Set | Training Set | Training Set | Training Set | Peng et al. (1995) J. Bio. Chem. (270) 14214-14219 | 0 | 1 | 0 | 0 | 0 | 0 |
|  | Training Set | Training Set | Training Set | Test Set | Test Set | Peng et al. (1995) J. Bio. Chem. (270) 14214-14219 | 0 | 1 | 0 | 0 | 0 | 0 |
|  | Training Set | Training Set | Training Set | Test Set | Training Set | Pierce et al. (1997) J. Bio. Chem. (272) 21096-21103 | 0 | 1 | 0 | 0 | 0 | 0 |
|  | Training Set | Training Set | Training Set | Training Set | Training Set | Pierce et al. (1997) J. Bio. Chem. (272) 21096-21103 | 0 | 1 | 0 | 0 | 0 | 0 |
|  | Training Set | Test Set | Test Set | Training Set | Training Set | Posadas et al (2003) Biochem. Pharmacol. (65) 887-895 | 0 | 1 | 0 | 0 | 0 | 0 |
|  | Training Set | Training Set | Training Set | Training Set | Training Set | Post et al. (1998) J. Neuroscience (18) 8236-8246 | 0 | 0 | 0 | 0 | 0 | 0 |
|  | Training Set | Training Set | Training Set | Training Set | Training Set | Preciado et al. (2005) Journal of Immunology (174) 29642973. Pierce et al. (1996) J. Immun. (156) 3961-3969 | 0 | 1 | 0 | 0 | 0 | 0 |
|  | Training Set | Test Set | Test Set | Training Set | Test Set | Pyatt et al (1998) Toxicology (128) 83-90 | 0 | 0 | 0 | 0 | 0 | 1 |
|  | Training Set | Training Set | Training Set | Training Set | Training Set | Pyatt et al. (1998) Toxicol. Appl. Pharmacol. (149) 178-184 | 0 | 0 | 0 | 0 | 0 | 0 |
|  | Training Set | Training Set | Training Set | Training Set | Training Set | Rahman et al. (2003) Biochem. Biophys. Res. Comm. (302) 860-864 | 0 | 0 | 0 | 0 | 0 | 1 |
|  | Training Set | Training Set | Training Set | Test Set | Training Set | Rioja et al. (2002) Naunyn-Schmiedeberg's Arch Pharmacol. (365) 357-364 | 0 | 1 | 0 | 0 | 0 | 0 |
|  | Test Set | Training Set | Training Set | Training Set | Training Set | Wahl et al. (1998) J. Clin. Invest. 1163-1174 Robe et al. (2004) Clinical Cancer Research (10) 5595-5603 Weber et al. (2000) Gastro. (119) 1209-1218 | 1 | 0 | 0 | 0 | 0 | 0 |
|  | Test Set | Training Set | Training Set | Training Set | Training Set | Sakurada et al. (1996) International Immunology (8) 1483-1493 | 0 | 0 | 0 | 0 | 1 | 0 |
|  | Training Set | Training Set | Training Set | Training Set | Training Set | Sancho et al (2003) Mol. Pharamcol. (63) 429-438 | 1 | 0 | 0 | 0 | 0 | 0 |
|  | Training Set | Training Set | Training Set | Training Set | Test Set | Sancho et al. (2002) Eur. J. Immunol. (32) 1753-1763 | 1 | 0 | 0 | 0 | 0 | 0 |
|  | Training Set | Training Set | Training Set | Training Set | Training Set | Scheuren et al. (1998) Brit J Pharm (123) 645-652 | 0 | 0 | 0 | 0 | 1 | 0 |
|  | Training Set | Test Set | Training Set | Training Set | Training Set | Scheuren et al. (1998) Brit J Pharm (123) 645-652 | 0 | 0 | 0 | 1 | 0 | 0 |
|  | Training Set | Training Set | Training Set | Training Set | Training Set | Sen at al (1998) Biochem. Biophys. Res. Comm. (247) 223-228 | 0 | 0 | 0 | 0 | 0 | 0 |
|  | Training Set | Training Set | Training Set | Training Set | Test Set | Sharma et al. (2004) Bio Med Chem Lett (14) 61236127 | 0 | 0 | 0 | 1 | 0 | 0 |
|  | Test Set | Training Set | Training Set | Training Set | Training Set | Schreck et al. (1992) J Exp Med (175) 1181-1194 | 0 | 0 | 0 | 1 | 0 | 0 |
|  | Test Set | Training Set | Training Set | Test Set | Training Set | Schreck et al. (1992) J Exp Med (175) 1181-1194 | 0 | 0 | 0 | 1 | 0 | 0 |
|  | Training Set | Training Set | Training Set | Training Set | Training Set | Schreck et al. (1992) J Exp Med (175) 1181-1194 | 0 | 0 | 0 | 1 | 0 | 0 |
|  | Test Set | Training Set | Training Set | Training Set | Training Set | Schreck et al. (1992) J Exp Med (175) 1181-1194 | 0 | 0 | 0 | 1 | 0 | 0 |
|  | Training Set | Training Set | Training Set | Training Set | Training Set | Schreck et al. (1992) J Exp Med (175) 1181-1194 Bessho et al (1994) Biochem Pharma (48) 1883-1889 Ferran et al. (1995) Biochem. Biophys. Res. Comm. (214) 212-223 Cuzzocrea et al (2002) Bre. J. Pharmacol. (135) 496-510 | 0 | 1 | 0 | 0 | 0 | 1 |
|  | Training Set | Test Set | Test Set | Training Set | Training Set | Singh & Aggarwal (1995) J Bio Chem (270) 10631-10639 Cuzzocrea et al (2002) Br. J. Pharmacol (135) 496-510 | 0 | 1 | 0 | 0 | 0 | 0 |
|  | Training Set | Training Set | Training Set | Training Set | Training Set | Singh & Aggarwal (1995) J Bio. Chem. (20) 24995-25000 Calzado et al (2007) Curr. Med. Chem. (14) 367-376 Jeong et al. (2004) Pharm. Res. (21) 661-670 | 0 | 1 | 0 | 0 | 0 | 0 |
|  | Training Set | Training Set | Training Set | Training Set | Test Set | Singh et al. (1996) J Immunology (157) 4412-4420 Sancho et al. (2002) Eur. J. Immunol. (32) 1753-1763 | 0 | 1 | 0 | 0 | 0 | 0 |
|  | Training Set | Training Set | Training Set | Training Set | Training Set | Sueoka et al. (1998) Biochem. Biophys. Res. Comm. (252) 566-570 | 0 | 0 | 0 | 0 | 0 | 0 |
|  | Training Set | Training Set | Training Set | Training Set | Training Set | Sueoka et al. (1998) Biochem. Biophys. Res. Comm. (252) 566-570 | 0 | 0 | 0 | 0 | 0 | 0 |
|  | Training Set | Test Set | Training Set | Training Set | Training Set | Sugano et al (1998) Biochem. Biophys. Res. Comm. (252) 25-28 | 0 | 1 | 0 | 0 | 0 | 0 |
|  | Training Set | Training Set | Training Set | Training Set | Training Set | Sugimoto et al. (2000) Biochem and Biophys Res Comm (277) 330-333 | 0 | 1 | 0 | 0 | 0 | 0 |
|  | Test Set | Training Set | Training Set | Training Set | Training Set | Suk et al. (2003) J. Pharmacol. Exp. Therap. (305) 638-645 | 0 | 0 | 0 | 0 | 1 | 0 |
|  | Training Set | Training Set | Training Set | Training Set | Training Set | Sunn et al. (1998) Biocehm Biophys res comm. (244) 691-695 | 0 | 1 | 0 | 0 | 0 | 0 |
|  | Training Set | Test Set | Training Set | Test Set | Training Set | Swinney et al. (2002) J Bio Chem (277) 23573-23581 | 0 | 1 | 0 | 0 | 0 | 0 |
|  | Test Set | Test Set | Training Set | Training Set | Test Set | Takagi et al (2002) Redox Rep. (7) 283-289 | 0 | 0 | 0 | 1 | 0 | 0 |
|  | Training Set | Test Set | Training Set | Training Set | Training Set | Tando et al. (2002) Digestion (66) 237 | 0 | 1 | 0 | 0 | 0 | 0 |
|  | Training Set | Test Set | Training Set | Training Set | Training Set | Teper et al. (1995) J. Immunol. (155) 2427-2436 | 0 | 0 | 0 | 0 | 1 | 0 |
|  | Training Set | Training Set | Test Set | Training Set | Training Set | Uchiba et al (2003) Crit. Car. Med. (31) 1147 | 0 | 1 | 0 | 0 | 0 | 0 |
|  | Training Set | Training Set | Training Set | Training Set | Training Set | Gehrt et al. (1998) J. antibiotics (51) 455 Umezawa et al. (2000) Anti-Can Drug Des (15) 239-244 | 0 | 1 | 0 | 0 | 0 | 0 |
|  | Training Set | Training Set | Training Set | Test Set | Test Set | Umezawa et al. (2000) Anti-Can Drug Des (15) 239-244 | 0 | 1 | 0 | 0 | 0 | 0 |
|  | Training Set | Training Set | Test Set | Training Set | Training Set | Umezawa et al. (2002) Mol Cells (14) 163-167 Suzuki et al. (2004) Tetrahedron (60) 70617066 Matsumoto et al. (2005) Clinical Cancer Research (11) 1287-1293 | 0 | 0 | 0 | 0 | 1 | 0 |
|  | Training Set | Training Set | Training Set | Training Set | Training Set | Venkataraman et al. (1995) J. Exp. Med. (181) 1091 | 0 | 0 | 0 | 0 | 0 | 0 |
|  | Training Set | Training Set | Training Set | Training Set | Training Set | Wang et al. (1997) Immunity (6) 165-174 | 0 | 0 | 0 | 0 | 0 | 0 |
|  | Training Set | Training Set | Training Set | Training Set | Training Set | Wang et al. (1998) Liver (18) 180 | 0 | 0 | 0 | 0 | 0 | 0 |
|  | Training Set | Training Set | Training Set | Training Set | Training Set | Yadav et al. (2003) J Pharmacol. Exp. Tech. (305) 925-931 | 0 | 0 | 0 | 0 | 0 | 0 |
|  | Training Set | Test Set | Training Set | Training Set | Test Set | Yamamoto et al (1999) J. Bio. Chem. (274) 27307-27314 | 0 | 1 | 0 | 0 | 0 | 0 |
|  | Training Set | Training Set | Training Set | Training Set | Training Set | Yang et al. (1998) J. Nutr. (128) 2334-2340 Jeong et al. (2004) Pharm. Res. (21) 661-670 Lee et al. (2005) J. Ethnopharm. (96) 211-219 | 0 | 1 | 0 | 1 | 0 | 0 |
|  | Training Set | Training Set | Training Set | Training Set | Training Set | Ye et al. (2000) Am J Physiol Lung Cell Mol Physiol (279) 615-617; Farmer and Pugin (2000) Am J Physiol Lung Cell Mol Physiol (279) L675-L682 | 0 | 1 | 0 | 0 | 0 | 0 |
|  | Test Set | Training Set | Training Set | Test Set | Training Set | Yore et al. (2006) Mol Cancer Ther (5) 3232-3239 | 1 | 0 | 0 | 0 | 0 | 0 |
|  | Training Set | Training Set | Training Set | Training Set | Training Set | Yore et al. (2006) Mol Cancer Ther (5) 3232-3239 | 1 | 0 | 0 | 0 | 0 | 0 |
|  | Training Set | Training Set | Training Set | Training Set | Training Set | Yoshida et al. (1999) J Immunol. (163) 351358. | 0 | 0 | 1 | 0 | 0 | 0 |
|  | Training Set | Training Set | Training Set | Training Set | Test Set | Murata, T., et al. 2004. Bioorg. Med. Chem. Lett. 14, 4019. | 1 | 0 | 0 | 0 | 0 | 0 |
|  | Training Set | Training Set | Training Set | Training Set | Training Set | Erdogan, E., et al. 2006. J. Biol. Chem. 281, 28450. Stallings-Mann, M., et al. 2006. Cancer Res. 66, 1767. Jeon, K.I., et al. 2000. J. Immunol. 164, 5981. Handel, M.L., et al. 1995. Proc. Natl. Acad. Sci. USA 92, 4497. | 0 | 0 | 0 | 0 | 0 | 0 |
|  | Training Set | Training Set | Training Set | Test Set | Training Set | Fernandes, P.A. (2005) Theor Chem Acc, 113, 197-204 | 0 | 0 | 0 | 1 | 0 | 0 |
|  | Test Set | Training Set | Training Set | Training Set | Training Set | Fernandes, P.A. (2005) Theor Chem Acc, 113, 197-204 | 0 | 0 | 0 | 1 | 0 | 0 |
|  | Training Set | Training Set | Test Set | Test Set | Training Set | Fernandes, P.A. (2005) Theor Chem Acc, 113, 197-204 | 0 | 0 | 0 | 1 | 0 | 0 |
|  | Training Set | Training Set | Training Set | Test Set | Training Set | Fernandes, P.A. (2005) Theor Chem Acc, 113, 197-204 | 0 | 0 | 0 | 1 | 0 | 0 |
|  | Training Set | Training Set | Training Set | Training Set | Training Set | Fernandes, P.A. (2005) Theor Chem Acc, 113, 197-204 | 0 | 0 | 0 | 1 | 0 | 0 |
|  | Training Set | Test Set | Training Set | Training Set | Test Set | Fernandes, P.A. (2005) Theor Chem Acc, 113, 197-204 | 0 | 0 | 0 | 1 | 0 | 0 |
|  | Training Set | Training Set | Training Set | Test Set | Training Set | Fernandes, P.A. (2005) Theor Chem Acc, 113, 197-204 | 0 | 0 | 0 | 1 | 0 | 0 |
|  | Training Set | Training Set | Training Set | Training Set | Training Set | Fernandes, P.A. (2005) Theor Chem Acc, 113, 197-204 | 0 | 0 | 0 | 1 | 0 | 0 |
|  | Training Set | Training Set | Training Set | Test Set | Training Set | Fernandes, P.A. (2005) Theor Chem Acc, 113, 197-204 | 0 | 0 | 0 | 1 | 0 | 0 |
|  | Training Set | Training Set | Training Set | Test Set | Training Set | Fernandes, P.A. (2005) Theor Chem Acc, 113, 197-204 | 0 | 0 | 0 | 1 | 0 | 0 |
|  | Training Set | Training Set | Training Set | Training Set | Training Set | Fernandes, P.A. (2005) Theor Chem Acc, 113, 197-204 | 0 | 0 | 0 | 1 | 0 | 0 |
|  | Training Set | Training Set | Test Set | Test Set | Training Set | Fernandes, P.A. (2005) Theor Chem Acc, 113, 197-204 | 0 | 0 | 0 | 1 | 0 | 0 |
|  | Training Set | Training Set | Training Set | Training Set | Training Set | Fernandes, P.A. (2005) Theor Chem Acc, 113, 197-204 | 0 | 0 | 0 | 1 | 0 | 0 |
|  | Test Set | Training Set | Training Set | Training Set | Training Set | Fernandes, P.A. (2005) Theor Chem Acc, 113, 197-204 | 0 | 0 | 0 | 1 | 0 | 0 |
|  | Training Set | Training Set | Training Set | Training Set | Training Set | Fernandes, P.A. (2005) Theor Chem Acc, 113, 197-204 | 0 | 0 | 0 | 1 | 0 | 0 |
|  | Training Set | Training Set | Training Set | Training Set | Training Set | Fernandes, P.A. (2005) Theor Chem Acc, 113, 197-204 | 0 | 0 | 0 | 1 | 0 | 0 |
|  | Training Set | Test Set | Training Set | Training Set | Training Set | Fernandes, P.A. (2005) Theor Chem Acc, 113, 197-204 | 0 | 0 | 0 | 1 | 0 | 0 |
|  | Training Set | Training Set | Training Set | Training Set | Training Set | Fernandes, P.A. (2005) Theor Chem Acc, 113, 197-204 | 0 | 0 | 0 | 1 | 0 | 0 |
|  | Training Set | Training Set | Training Set | Training Set | Training Set | Fernandes, P.A. (2005) Theor Chem Acc, 113, 197-204 | 0 | 0 | 0 | 1 | 0 | 0 |
|  | Training Set | Training Set | Training Set | Training Set | Training Set | Fernandes, P.A. (2005) Theor Chem Acc, 113, 197-204 | 0 | 0 | 0 | 1 | 0 | 0 |
|  | Test Set | Training Set | Training Set | Training Set | Test Set | Fernandes, P.A. (2005) Theor Chem Acc, 113, 197-204 | 0 | 0 | 0 | 1 | 0 | 0 |
|  | Training Set | Training Set | Test Set | Test Set | Training Set | Fernandes, P.A. (2005) Theor Chem Acc, 113, 197-204 | 0 | 0 | 0 | 1 | 0 | 0 |
|  | Training Set | Training Set | Training Set | Training Set | Training Set | Fernandes, P.A. (2005) Theor Chem Acc, 113, 197-204 | 0 | 0 | 0 | 1 | 0 | 0 |
|  | Training Set | Training Set | Training Set | Training Set | Training Set | Gurova, k.V., et al (2005) PNAS 102, 17448-17453 | 0 | 0 | 0 | 1 | 0 | 0 |
|  | Training Set | Training Set | Training Set | Training Set | Training Set | Gurova, k.V., et al (2005) PNAS 102, 17448-17453 | 0 | 0 | 0 | 0 | 0 | 0 |
|  | Training Set | Training Set | Training Set | Training Set | Training Set | Gurova, k.V., et al (2005) PNAS 102, 17448-17453 | 0 | 0 | 0 | 0 | 0 | 0 |
|  | Training Set | Training Set | Training Set | Training Set | Training Set | Ashikawa, K. et al (2002) J Immun. (169) 6490-6497 | 0 | 1 | 0 | 0 | 0 | 0 |
|  | Training Set | Training Set | Training Set | Training Set | Training Set | Kim et al (2006) Aging Cell (5) 401-411 | 0 | 1 | 0 | 0 | 0 | 0 |
|  | Training Set | Test Set | Training Set | Training Set | Test Set | Sandur et al (2006) J. Bio. Chem. (281) 17023-17033 | 1 | 0 | 0 | 1 | 0 | 0 |
|  | Training Set | Training Set | Training Set | Training Set | Training Set | Ahn et al (2008) Biochemical Pharmacology 75, 907-913 | 0 | 0 | 0 | 0 | 0 | 0 |
|  | Training Set | Training Set | Training Set | Training Set | Training Set | Ahn et al (2008) Biochemical Pharmacology 75, 907-913 | 0 | 0 | 0 | 0 | 0 | 0 |
|  | Training Set | Training Set | Training Set | Training Set | Training Set | Ahn et al (2008) Biochemical Pharmacology 75, 907-913 | 0 | 0 | 0 | 0 | 0 | 0 |
|  | Training Set | Training Set | Training Set | Training Set | Training Set | Ahn et al (2008) Biochemical Pharmacology 75, 907-913 | 0 | 0 | 0 | 0 | 0 | 0 |
|  | Test Set | Training Set | Training Set | Training Set | Training Set | Xu et al. (2007) Rheumatology, 46, 920-926 | 0 | 1 | 0 | 0 | 0 | 0 |
|  | Training Set | Training Set | Test Set | Training Set | Training Set | Castro et al (2003) Bioorg & Med Chem Letts 2419-2422 | 1 | 0 | 0 | 0 | 0 | 0 |
|  | Test Set | Training Set | Training Set | Training Set | Training Set | Calzado et al (2007) Curr. Med. Chem. (14) 367-376 | 1 | 0 | 0 | 0 | 0 | 0 |
|  | Training Set | Training Set | Training Set | Test Set | Test Set | Baxter et al (2004) Bioorg & Med Chem Letts. (14) 2817-2822 | 1 | 0 | 0 | 0 | 0 | 0 |
|  | Training Set | Training Set | Training Set | Training Set | Training Set | Baxter et al (2004) Bioorg & Med Chem Letts. (14) 2817-2822 | 1 | 0 | 0 | 0 | 0 | 0 |
|  | Training Set | Training Set | Training Set | Training Set | Training Set | Baxter et al (2004) Bioorg & Med Chem Letts. (14) 2817-2822 | 1 | 0 | 0 | 0 | 0 | 0 |
|  | Training Set | Training Set | Training Set | Training Set | Training Set | Baxter et al (2004) Bioorg & Med Chem Letts. (14) 2817-2822 | 1 | 0 | 0 | 0 | 0 | 0 |
|  | Training Set | Training Set | Training Set | Training Set | Training Set | Baxter et al (2004) Bioorg & Med Chem Letts. (14) 2817-2822 | 1 | 0 | 0 | 0 | 0 | 0 |
|  | Training Set | Test Set | Training Set | Test Set | Training Set | Baxter et al (2004) Bioorg & Med Chem Letts. (14) 2817-2822 | 1 | 0 | 0 | 0 | 0 | 0 |
|  | Test Set | Test Set | Training Set | Test Set | Training Set | Baxter et al (2004) Bioorg & Med Chem Letts. (14) 2817-2822 | 1 | 0 | 0 | 0 | 0 | 0 |
|  | Training Set | Test Set | Training Set | Training Set | Training Set | Baxter et al (2004) Bioorg & Med Chem Letts. (14) 2817-2822 | 1 | 0 | 0 | 0 | 0 | 0 |
|  | Training Set | Training Set | Training Set | Test Set | Training Set | Baxter et al (2004) Bioorg & Med Chem Letts. (14) 2817-2822 | 1 | 0 | 0 | 0 | 0 | 0 |
|  | Training Set | Training Set | Training Set | Training Set | Training Set | Baxter et al (2004) Bioorg & Med Chem Letts. (14) 2817-2822 | 1 | 0 | 0 | 0 | 0 | 0 |
|  | Training Set | Training Set | Training Set | Training Set | Training Set | Baxter et al (2004) Bioorg & Med Chem Letts. (14) 2817-2822 | 1 | 0 | 0 | 0 | 0 | 0 |
|  | Training Set | Training Set | Training Set | Training Set | Test Set | Baxter et al (2004) Bioorg & Med Chem Letts. (14) 2817-2822 | 1 | 0 | 0 | 0 | 0 | 0 |
|  | Test Set | Test Set | Training Set | Training Set | Training Set | Baxter et al (2004) Bioorg & Med Chem Letts. (14) 2817-2822 | 1 | 0 | 0 | 0 | 0 | 0 |
|  | Test Set | Training Set | Training Set | Training Set | Training Set | Baxter et al (2004) Bioorg & Med Chem Letts. (14) 2817-2822 | 1 | 0 | 0 | 0 | 0 | 0 |
|  | Training Set | Training Set | Training Set | Training Set | Test Set | Baxter et al (2004) Bioorg & Med Chem Letts. (14) 2817-2822 | 1 | 0 | 0 | 0 | 0 | 0 |
|  | Training Set | Training Set | Training Set | Training Set | Training Set | Baxter et al (2004) Bioorg & Med Chem Letts. (14) 2817-2822 | 1 | 0 | 0 | 0 | 0 | 0 |
|  | Test Set | Training Set | Training Set | Training Set | Training Set | Heiss et al. (2001) J. Bio. Chem. 276 (94) 32008-32015 Jeong et al. (2004) Pharm. Res. (21) 661-670 | 0 | 0 | 0 | 1 | 0 | 0 |
|  | Test Set | Training Set | Training Set | Training Set | Training Set | Bayon et al. (2003) Mol. Cell. Biol. (23) 1061 | 1 | 0 | 0 | 0 | 0 | 0 |
|  | Training Set | Training Set | Training Set | Training Set | Training Set | Heynekamp et al. (2006) J. Med. Chem. 7182-7189 | 0 | 0 | 0 | 0 | 0 | 0 |
|  | Training Set | Training Set | Training Set | Training Set | Training Set | Heynekamp et al. (2006) J. Med. Chem. 7182-7189 | 0 | 0 | 0 | 0 | 0 | 0 |
|  | Training Set | Training Set | Training Set | Training Set | Training Set | Heynekamp et al. (2006) J. Med. Chem. 7182-7189 | 0 | 0 | 0 | 0 | 0 | 0 |
|  | Training Set | Training Set | Training Set | Training Set | Training Set | Heynekamp et al. (2006) J. Med. Chem. 7182-7189 | 0 | 0 | 0 | 0 | 0 | 0 |
|  | Training Set | Training Set | Training Set | Training Set | Training Set | Heynekamp et al. (2006) J. Med. Chem. 7182-7189 | 0 | 0 | 0 | 0 | 0 | 0 |
|  | Training Set | Training Set | Training Set | Training Set | Training Set | Heynekamp et al. (2006) J. Med. Chem. 7182-7189 | 0 | 0 | 0 | 0 | 0 | 0 |
|  | Training Set | Training Set | Training Set | Training Set | Training Set | Heynekamp et al. (2006) J. Med. Chem. 7182-7189 | 0 | 0 | 0 | 0 | 0 | 0 |
|  | Training Set | Training Set | Training Set | Training Set | Training Set | Heynekamp et al. (2006) J. Med. Chem. 7182-7189 | 0 | 0 | 0 | 0 | 0 | 0 |
|  | Training Set | Training Set | Training Set | Training Set | Training Set | Heynekamp et al. (2006) J. Med. Chem. 7182-7189 | 0 | 0 | 0 | 0 | 0 | 0 |
|  | Training Set | Training Set | Training Set | Training Set | Training Set | Heynekamp et al. (2006) J. Med. Chem. 7182-7189 | 0 | 0 | 0 | 0 | 0 | 0 |
|  | Training Set | Training Set | Training Set | Training Set | Training Set | Heynekamp et al. (2006) J. Med. Chem. 7182-7189 | 0 | 0 | 0 | 0 | 0 | 0 |
|  | Training Set | Training Set | Training Set | Training Set | Training Set | Heynekamp et al. (2006) J. Med. Chem. 7182-7189 | 0 | 0 | 0 | 0 | 0 | 0 |
|  | Training Set | Training Set | Training Set | Training Set | Training Set | Heynekamp et al. (2006) J. Med. Chem. 7182-7189 | 0 | 0 | 0 | 0 | 0 | 0 |
|  | Training Set | Training Set | Training Set | Training Set | Training Set | Heynekamp et al. (2006) J. Med. Chem. 7182-7189 | 0 | 0 | 0 | 0 | 0 | 0 |
|  | Training Set | Training Set | Training Set | Training Set | Training Set | Heynekamp et al. (2006) J. Med. Chem. 7182-7189 | 0 | 0 | 0 | 0 | 0 | 0 |
|  | Training Set | Training Set | Training Set | Training Set | Training Set | Heynekamp et al. (2006) J. Med. Chem. 7182-7189 | 0 | 0 | 0 | 0 | 0 | 0 |
|  | Training Set | Training Set | Training Set | Training Set | Training Set | Heynekamp et al. (2006) J. Med. Chem. 7182-7189 | 0 | 0 | 0 | 0 | 0 | 0 |
|  | Training Set | Training Set | Training Set | Training Set | Training Set | Heynekamp et al. (2006) J. Med. Chem. 7182-7189 | 0 | 0 | 0 | 0 | 0 | 0 |
|  | Training Set | Training Set | Training Set | Training Set | Training Set | Heynekamp et al. (2006) J. Med. Chem. 7182-7189 | 0 | 0 | 0 | 0 | 0 | 0 |
|  | Training Set | Training Set | Training Set | Training Set | Training Set | Heynekamp et al. (2006) J. Med. Chem. 7182-7189 | 0 | 0 | 0 | 0 | 0 | 0 |
|  | Training Set | Training Set | Training Set | Training Set | Training Set | Heynekamp et al. (2006) J. Med. Chem. 7182-7189 | 0 | 0 | 0 | 0 | 0 | 0 |
|  | Training Set | Training Set | Training Set | Training Set | Training Set | Heynekamp et al. (2006) J. Med. Chem. 7182-7189 | 0 | 0 | 0 | 0 | 0 | 0 |
|  | Training Set | Training Set | Training Set | Training Set | Training Set | Heynekamp et al. (2006) J. Med. Chem. 7182-7189 | 0 | 0 | 0 | 0 | 0 | 0 |
|  | Training Set | Training Set | Training Set | Training Set | Training Set | Heynekamp et al. (2006) J. Med. Chem. 7182-7189 | 0 | 0 | 0 | 0 | 0 | 0 |
|  | Training Set | Training Set | Training Set | Training Set | Training Set | Heynekamp et al. (2006) J. Med. Chem. 7182-7189 | 0 | 0 | 0 | 0 | 0 | 0 |
|  | Training Set | Training Set | Training Set | Training Set | Training Set | Heynekamp et al. (2006) J. Med. Chem. 7182-7189 | 0 | 0 | 0 | 0 | 0 | 0 |
|  | Training Set | Training Set | Training Set | Training Set | Training Set | Heynekamp et al. (2006) J. Med. Chem. 7182-7189 | 0 | 0 | 0 | 0 | 0 | 0 |
|  | Training Set | Training Set | Training Set | Training Set | Training Set | Heynekamp et al. (2006) J. Med. Chem. 7182-7189 | 0 | 0 | 0 | 0 | 0 | 0 |
|  | Training Set | Training Set | Training Set | Training Set | Training Set | Heynekamp et al. (2006) J. Med. Chem. 7182-7189 | 0 | 0 | 0 | 0 | 0 | 0 |
|  | Training Set | Training Set | Training Set | Training Set | Training Set | Heynekamp et al. (2006) J. Med. Chem. 7182-7189 | 0 | 0 | 0 | 0 | 0 | 0 |
|  | Training Set | Training Set | Training Set | Training Set | Training Set | Heynekamp et al. (2006) J. Med. Chem. 7182-7189 | 0 | 0 | 0 | 0 | 0 | 0 |
|  | Training Set | Training Set | Training Set | Training Set | Training Set | Heynekamp et al. (2006) J. Med. Chem. 7182-7189 | 0 | 0 | 0 | 0 | 0 | 0 |
|  | Training Set | Training Set | Training Set | Training Set | Training Set | Heynekamp et al. (2006) J. Med. Chem. 7182-7189 | 0 | 0 | 0 | 0 | 0 | 0 |
|  | Training Set | Training Set | Training Set | Training Set | Training Set | Heynekamp et al. (2006) J. Med. Chem. 7182-7189 | 0 | 0 | 0 | 0 | 0 | 0 |
|  | Training Set | Training Set | Training Set | Training Set | Training Set | Heynekamp et al. (2006) J. Med. Chem. 7182-7189 | 0 | 0 | 0 | 0 | 0 | 0 |
|  | Training Set | Training Set | Training Set | Training Set | Training Set | Heynekamp et al. (2006) J. Med. Chem. 7182-7189 | 0 | 0 | 0 | 0 | 0 | 0 |
|  | Training Set | Training Set | Training Set | Training Set | Training Set | Heynekamp et al. (2006) J. Med. Chem. 7182-7189 | 0 | 0 | 0 | 0 | 0 | 0 |
|  | Training Set | Training Set | Training Set | Training Set | Training Set | Heynekamp et al. (2006) J. Med. Chem. 7182-7189 | 0 | 0 | 0 | 0 | 0 | 0 |
|  | Training Set | Training Set | Training Set | Training Set | Training Set | Heynekamp et al. (2006) J. Med. Chem. 7182-7189 | 0 | 0 | 0 | 0 | 0 | 0 |
|  | Training Set | Training Set | Training Set | Training Set | Training Set | Heynekamp et al. (2006) J. Med. Chem. 7182-7189 | 0 | 0 | 0 | 0 | 0 | 0 |
|  | Training Set | Training Set | Training Set | Training Set | Training Set | Heynekamp et al. (2006) J. Med. Chem. 7182-7189 | 0 | 0 | 0 | 0 | 0 | 0 |
|  | Training Set | Training Set | Training Set | Training Set | Training Set | Heynekamp et al. (2006) J. Med. Chem. 7182-7189 | 0 | 0 | 0 | 0 | 0 | 0 |
|  | Training Set | Training Set | Training Set | Training Set | Training Set | Heynekamp et al. (2006) J. Med. Chem. 7182-7189 | 0 | 0 | 0 | 0 | 0 | 0 |
|  | Training Set | Training Set | Training Set | Training Set | Training Set | Heynekamp et al. (2006) J. Med. Chem. 7182-7189 | 0 | 0 | 0 | 0 | 0 | 0 |
|  | Training Set | Training Set | Training Set | Training Set | Training Set | Heynekamp et al. (2006) J. Med. Chem. 7182-7189 | 0 | 0 | 0 | 0 | 0 | 0 |
|  | Training Set | Training Set | Training Set | Training Set | Training Set | Heynekamp et al. (2006) J. Med. Chem. 7182-7189 | 0 | 0 | 0 | 0 | 0 | 0 |
|  | Training Set | Training Set | Training Set | Training Set | Training Set | Heynekamp et al. (2006) J. Med. Chem. 7182-7189 | 0 | 0 | 0 | 0 | 0 | 0 |
|  | Training Set | Training Set | Training Set | Training Set | Training Set | Heynekamp et al. (2006) J. Med. Chem. 7182-7189 | 0 | 0 | 0 | 0 | 0 | 0 |
|  | Training Set | Training Set | Training Set | Training Set | Training Set | Heynekamp et al. (2006) J. Med. Chem. 7182-7189 | 0 | 0 | 0 | 0 | 0 | 0 |
|  | Training Set | Training Set | Training Set | Training Set | Training Set | Heynekamp et al. (2006) J. Med. Chem. 7182-7189 | 0 | 0 | 0 | 0 | 0 | 0 |
|  | Training Set | Training Set | Training Set | Training Set | Training Set | Heynekamp et al. (2006) J. Med. Chem. 7182-7189 | 0 | 0 | 0 | 0 | 0 | 0 |
|  | Training Set | Training Set | Training Set | Training Set | Training Set | Heynekamp et al. (2006) J. Med. Chem. 7182-7189 | 0 | 0 | 0 | 0 | 0 | 0 |
|  | Training Set | Training Set | Training Set | Training Set | Training Set | Heynekamp et al. (2006) J. Med. Chem. 7182-7189 | 0 | 0 | 0 | 0 | 0 | 0 |
|  | Training Set | Training Set | Training Set | Training Set | Training Set | Heynekamp et al. (2006) J. Med. Chem. 7182-7189 | 0 | 0 | 0 | 0 | 0 | 0 |
|  | Training Set | Training Set | Training Set | Training Set | Training Set | Heynekamp et al. (2006) J. Med. Chem. 7182-7189 | 0 | 0 | 0 | 0 | 0 | 0 |
|  | Training Set | Training Set | Training Set | Training Set | Training Set | Heynekamp et al. (2006) J. Med. Chem. 7182-7189 | 0 | 0 | 0 | 0 | 0 | 0 |
|  | Training Set | Training Set | Training Set | Training Set | Training Set | Heynekamp et al. (2006) J. Med. Chem. 7182-7189 | 0 | 0 | 0 | 0 | 0 | 0 |
|  | Training Set | Training Set | Training Set | Training Set | Training Set | Heynekamp et al. (2006) J. Med. Chem. 7182-7189 | 0 | 0 | 0 | 0 | 0 | 0 |
|  | Training Set | Training Set | Training Set | Training Set | Training Set | Heynekamp et al. (2006) J. Med. Chem. 7182-7189 | 0 | 0 | 0 | 0 | 0 | 0 |
|  | Training Set | Training Set | Training Set | Training Set | Training Set | Heynekamp et al. (2006) J. Med. Chem. 7182-7189 | 0 | 0 | 0 | 0 | 0 | 0 |
|  | Training Set | Training Set | Training Set | Training Set | Training Set | Heynekamp et al. (2006) J. Med. Chem. 7182-7189 | 0 | 0 | 0 | 0 | 0 | 0 |
|  | Training Set | Training Set | Training Set | Training Set | Training Set | Heynekamp et al. (2006) J. Med. Chem. 7182-7189 | 0 | 0 | 0 | 0 | 0 | 0 |
|  | Training Set | Training Set | Training Set | Training Set | Training Set | Heynekamp et al. (2006) J. Med. Chem. 7182-7189 | 0 | 0 | 0 | 0 | 0 | 0 |
|  | Training Set | Training Set | Training Set | Training Set | Training Set | Heynekamp et al. (2006) J. Med. Chem. 7182-7189 | 0 | 0 | 0 | 0 | 0 | 0 |
|  | Training Set | Training Set | Training Set | Training Set | Training Set | Heynekamp et al. (2006) J. Med. Chem. 7182-7189 | 0 | 0 | 0 | 0 | 0 | 0 |
|  | Training Set | Training Set | Training Set | Training Set | Training Set | Heynekamp et al. (2006) J. Med. Chem. 7182-7189 | 0 | 0 | 0 | 0 | 0 | 0 |
|  | Training Set | Training Set | Training Set | Training Set | Training Set | Heynekamp et al. (2006) J. Med. Chem. 7182-7189 | 0 | 0 | 0 | 0 | 0 | 0 |
|  | Training Set | Training Set | Training Set | Training Set | Training Set | Heynekamp et al. (2006) J. Med. Chem. 7182-7189 | 0 | 0 | 0 | 0 | 0 | 0 |
|  | Training Set | Training Set | Training Set | Training Set | Training Set | Heynekamp et al. (2006) J. Med. Chem. 7182-7189 | 0 | 0 | 0 | 0 | 0 | 0 |
|  | Training Set | Training Set | Training Set | Training Set | Training Set | Heynekamp et al. (2006) J. Med. Chem. 7182-7189 | 0 | 0 | 0 | 0 | 0 | 0 |
|  | Training Set | Training Set | Training Set | Training Set | Training Set | Heynekamp et al. (2006) J. Med. Chem. 7182-7189 | 0 | 0 | 0 | 0 | 0 | 0 |
|  | Training Set | Training Set | Training Set | Training Set | Training Set | Heynekamp et al. (2006) J. Med. Chem. 7182-7189 | 0 | 0 | 0 | 0 | 0 | 0 |
|  | Training Set | Training Set | Training Set | Training Set | Training Set | Heynekamp et al. (2006) J. Med. Chem. 7182-7189 | 0 | 0 | 0 | 0 | 0 | 0 |
|  | Training Set | Training Set | Training Set | Training Set | Training Set | Heynekamp et al. (2006) J. Med. Chem. 7182-7189 Kundu & Surh (2004) Mut. Res. 65-80 Jeong et al. (2004) Pharm. Res. (21) 661-670 Lee et al. (2005) J. Ethnopharm. (96) 211-219 | 0 | 1 | 0 | 0 | 0 | 0 |
|  | Training Set | Training Set | Training Set | Training Set | Training Set | Heynekamp et al. (2006) J. Med. Chem. 7182-7189 | 0 | 0 | 0 | 0 | 0 | 0 |
|  | Training Set | Training Set | Training Set | Training Set | Training Set | Bauman et al. (2002) J Bio Chem (277) 44791-44800 | 0 | 0 | 0 | 0 | 0 | 0 |
|  | Training Set | Training Set | Training Set | Training Set | Training Set | Bauman et al. (2002) J Bio Chem (277) 44791-44800 | 0 | 0 | 0 | 0 | 0 | 0 |
|  | Training Set | Training Set | Training Set | Training Set | Training Set | Bauman et al. (2002) J Bio Chem (277) 44791-44800 | 0 | 0 | 0 | 0 | 0 | 0 |
|  | Training Set | Training Set | Training Set | Training Set | Training Set | Bauman et al. (2002) J Bio Chem (277) 44791-44800 | 0 | 0 | 0 | 0 | 0 | 0 |
|  | Training Set | Training Set | Training Set | Training Set | Training Set | Bauman et al. (2002) J Bio Chem (277) 44791-44800 | 0 | 0 | 0 | 0 | 0 | 0 |
|  | Training Set | Training Set | Training Set | Training Set | Training Set | Bauman et al. (2002) J Bio Chem (277) 44791-44800 | 0 | 0 | 0 | 0 | 0 | 0 |
|  | Training Set | Training Set | Training Set | Training Set | Training Set | Bauman et al. (2002) J Bio Chem (277) 44791-44800 | 0 | 0 | 0 | 0 | 0 | 0 |
|  | Training Set | Training Set | Training Set | Training Set | Training Set | Bauman et al. (2002) J Bio Chem (277) 44791-44800 | 0 | 0 | 0 | 0 | 0 | 0 |
|  | Training Set | Training Set | Training Set | Training Set | Training Set | Bauman et al. (2002) J Bio Chem (277) 44791-44800 | 0 | 0 | 0 | 0 | 0 | 0 |
|  | Training Set | Training Set | Training Set | Training Set | Training Set | Bauman et al. (2002) J Bio Chem (277) 44791-44800 | 0 | 0 | 0 | 0 | 0 | 0 |
|  | Training Set | Training Set | Training Set | Test Set | Training Set | Castro et al (2003) Bioorg & Med Chem Letts 2419-2422 | 1 | 0 | 0 | 0 | 0 | 0 |
|  | Training Set | Training Set | Training Set | Training Set | Training Set | Castro et al (2003) Bioorg & Med Chem Letts 2419-2422 | 1 | 0 | 0 | 0 | 0 | 0 |
|  | Training Set | Training Set | Training Set | Test Set | Test Set | Castro et al (2003) Bioorg & Med Chem Letts 2419-2422 | 1 | 0 | 0 | 0 | 0 | 0 |
|  | Training Set | Training Set | Test Set | Test Set | Training Set | Castro et al (2003) Bioorg & Med Chem Letts 2419-2422 | 1 | 0 | 0 | 0 | 0 | 0 |
|  | Training Set | Training Set | Training Set | Training Set | Training Set | Castro et al (2003) Bioorg & Med Chem Letts 2419-2422 | 1 | 0 | 0 | 0 | 0 | 0 |
|  | Test Set | Training Set | Training Set | Training Set | Training Set | Castro et al (2003) Bioorg & Med Chem Letts 2419-2422 | 1 | 0 | 0 | 0 | 0 | 0 |
|  | Training Set | Test Set | Training Set | Training Set | Training Set | Castro et al (2003) Bioorg & Med Chem Letts 2419-2422 | 1 | 0 | 0 | 0 | 0 | 0 |
|  | Training Set | Test Set | Test Set | Training Set | Training Set | Castro et al (2003) Bioorg & Med Chem Letts 2419-2422 | 1 | 0 | 0 | 0 | 0 | 0 |
|  | Training Set | Test Set | Test Set | Training Set | Test Set | Castro et al (2003) Bioorg & Med Chem Letts 2419-2422 | 1 | 0 | 0 | 0 | 0 | 0 |
|  | Training Set | Training Set | Test Set | Training Set | Training Set | Castro et al (2003) Bioorg & Med Chem Letts 2419-2422 | 1 | 0 | 0 | 0 | 0 | 0 |
|  | Training Set | Training Set | Training Set | Training Set | Test Set | Castro et al (2003) Bioorg & Med Chem Letts 2419-2422 | 1 | 0 | 0 | 0 | 0 | 0 |
|  | Test Set | Training Set | Test Set | Test Set | Training Set | Castro et al (2003) Bioorg & Med Chem Letts 2419-2422 | 1 | 0 | 0 | 0 | 0 | 0 |
|  | Training Set | Training Set | Training Set | Training Set | Training Set | Castro et al (2003) Bioorg & Med Chem Letts 2419-2422 | 1 | 0 | 0 | 0 | 0 | 0 |
|  | Training Set | Training Set | Training Set | Training Set | Training Set | Castro et al (2003) Bioorg & Med Chem Letts 2419-2422 | 1 | 0 | 0 | 0 | 0 | 0 |
|  | Training Set | Training Set | Test Set | Training Set | Training Set | Castro et al (2003) Bioorg & Med Chem Letts 2419-2422 | 1 | 0 | 0 | 0 | 0 | 0 |
|  | Training Set | Training Set | Training Set | Training Set | Test Set | Castro et al (2003) Bioorg & Med Chem Letts 2419-2422 | 1 | 0 | 0 | 0 | 0 | 0 |
|  | Training Set | Test Set | Training Set | Training Set | Training Set | Castro et al (2003) Bioorg & Med Chem Letts 2419-2422 | 1 | 0 | 0 | 0 | 0 | 0 |
|  | Training Set | Training Set | Test Set | Training Set | Training Set | Castro et al (2003) Bioorg & Med Chem Letts 2419-2422 | 1 | 0 | 0 | 0 | 0 | 0 |
|  | Training Set | Training Set | Training Set | Test Set | Training Set | Castro et al (2003) Bioorg & Med Chem Letts 2419-2422 | 1 | 0 | 0 | 0 | 0 | 0 |
|  | Training Set | Training Set | Training Set | Training Set | Training Set | Castro et al (2003) Bioorg & Med Chem Letts 2419-2422 | 1 | 0 | 0 | 0 | 0 | 0 |
|  | Training Set | Training Set | Training Set | Training Set | Training Set | Castro et al (2003) Bioorg & Med Chem Letts 2419-2422 | 1 | 0 | 0 | 0 | 0 | 0 |
|  | Training Set | Training Set | Test Set | Training Set | Training Set | Castro et al (2003) Bioorg & Med Chem Letts 2419-2422 | 1 | 0 | 0 | 0 | 0 | 0 |
|  | Training Set | Training Set | Training Set | Training Set | Training Set | Castro et al (2003) Bioorg & Med Chem Letts 2419-2422 | 1 | 0 | 0 | 0 | 0 | 0 |
|  | Training Set | Training Set | Training Set | Training Set | Training Set | Castro et al (2003) Bioorg & Med Chem Letts 2419-2422 | 1 | 0 | 0 | 0 | 0 | 0 |
|  | Training Set | Test Set | Test Set | Test Set | Training Set | Castro et al (2003) Bioorg & Med Chem Letts 2419-2422 | 1 | 0 | 0 | 0 | 0 | 0 |
|  | Training Set | Training Set | Test Set | Test Set | Training Set | Castro et al (2003) Bioorg & Med Chem Letts 2419-2422 | 1 | 0 | 0 | 0 | 0 | 0 |
|  | Training Set | Training Set | Training Set | Training Set | Training Set | Castro et al (2003) Bioorg & Med Chem Letts 2419-2422 | 1 | 0 | 0 | 0 | 0 | 0 |
|  | Training Set | Test Set | Test Set | Training Set | Training Set | Castro et al (2003) Bioorg & Med Chem Letts 2419-2422 | 1 | 0 | 0 | 0 | 0 | 0 |
|  | Training Set | Training Set | Training Set | Training Set | Test Set | Castro et al (2003) Bioorg & Med Chem Letts 2419-2422 | 1 | 0 | 0 | 0 | 0 | 0 |
|  | Training Set | Training Set | Training Set | Training Set | Training Set | Castro et al (2003) Bioorg & Med Chem Letts 2419-2422 | 1 | 0 | 0 | 0 | 0 | 0 |
|  | Training Set | Training Set | Training Set | Training Set | Training Set | Castro et al (2003) Bioorg & Med Chem Letts 2419-2422 | 1 | 0 | 0 | 0 | 0 | 0 |
|  | Training Set | Training Set | Training Set | Training Set | Training Set | Castro et al (2003) Bioorg & Med Chem Letts 2419-2422 | 1 | 0 | 0 | 0 | 0 | 0 |
|  | Training Set | Training Set | Training Set | Training Set | Test Set | Castro et al (2003) Bioorg & Med Chem Letts 2419-2422 | 1 | 0 | 0 | 0 | 0 | 0 |
|  | Training Set | Test Set | Training Set | Training Set | Training Set | Castro et al (2003) Bioorg & Med Chem Letts 2419-2422 | 1 | 0 | 0 | 0 | 0 | 0 |
|  | Training Set | Training Set | Training Set | Test Set | Test Set | Calzado et al (2007) Curr. Med. Chem. (14) 367-376 | 0 | 0 | 0 | 0 | 1 | 0 |
|  | Training Set | Training Set | Training Set | Training Set | Training Set | Chanani et al. (2002) Circulation (106) I284-I285 | 0 | 0 | 0 | 0 | 0 | 0 |
|  | Training Set | Training Set | Test Set | Training Set | Training Set | Haddad et al. (2002) Int. ImmunoPharmacology (2) 1567-1583 | 0 | 1 | 0 | 0 | 0 | 0 |
|  | Training Set | Training Set | Training Set | Training Set | Training Set | Jeong et al. (2004) Pharm. Res. (21) 661-670 | 0 | 1 | 0 | 0 | 0 | 0 |
|  | Training Set | Training Set | Training Set | Training Set | Test Set | Jeong et al. (2004) Pharm. Res. (21) 661-670 | 0 | 1 | 0 | 0 | 0 | 0 |
|  | Training Set | Training Set | Training Set | Training Set | Training Set | Kumar et al. (1998) Oncogene (17) 913-918 | 0 | 1 | 0 | 0 | 0 | 0 |
|  | Training Set | Training Set | Training Set | Training Set | Training Set | Lee et al. (2005) J. Ethnopharm. (96) 211-219 | 0 | 0 | 0 | 0 | 0 | 0 |
|  | Training Set | Training Set | Training Set | Training Set | Training Set | Lee et al. (2005) J. Ethnopharm. (96) 211-219 | 0 | 0 | 0 | 0 | 0 | 0 |
|  | Training Set | Training Set | Training Set | Training Set | Training Set | Lee et al. (2005) J. Ethnopharm. (96) 211-219 | 0 | 0 | 0 | 0 | 0 | 0 |
|  | Training Set | Training Set | Training Set | Training Set | Training Set | Lee et al. (2005) J. Ethnopharm. (96) 211-219 | 0 | 0 | 0 | 0 | 0 | 0 |
|  | Training Set | Training Set | Training Set | Training Set | Training Set | Lee et al. (2005) J. Ethnopharm. (96) 211-219 | 0 | 0 | 0 | 0 | 0 | 0 |
|  | Training Set | Training Set | Training Set | Training Set | Training Set | Li et al. (2005) Chin. Med. J. (118) 111-115 | 0 | 0 | 0 | 1 | 0 | 0 |
|  | Training Set | Training Set | Training Set | Training Set | Training Set | Li et al. (2002) Org. Letts. (4) 2367-2370 | 0 | 0 | 0 | 0 | 0 | 0 |
|  | Training Set | Training Set | Training Set | Training Set | Training Set | Li et al. (2002) Org. Letts. (4) 2367-2370 | 0 | 0 | 0 | 0 | 0 | 0 |
|  | Training Set | Training Set | Training Set | Training Set | Training Set | Li et al. (2002) Org. Letts. (4) 2367-2370 | 0 | 0 | 0 | 0 | 0 | 0 |
|  | Training Set | Training Set | Training Set | Training Set | Test Set | Liu & Malix (2006) Am. J. Physiol. Lung Cell Mol. Phys. (290) 622-645 | 0 | 1 | 0 | 0 | 0 | 0 |
|  | Training Set | Training Set | Training Set | Training Set | Training Set | Qiu et al. (1999) J. Biol. Chem. (274) 13443-13450 Ma. et al (2004) Am. J. Physiol Gast. Liv. Phys. (286) 367-376 | 0 | 0 | 0 | 0 | 0 | 0 |
|  | Training Set | Training Set | Training Set | Training Set | Training Set | Manna et al. (2003) Canc. Lett. 171-182 | 0 | 1 | 0 | 0 | 0 | 0 |
|  | Training Set | Training Set | Training Set | Training Set | Training Set | Manna et al. (2003) Canc. Lett. 171-182 | 0 | 1 | 0 | 0 | 0 | 0 |
|  | Training Set | Training Set | Training Set | Training Set | Test Set | Manna et al. (2003) Canc. Lett. 171-182 | 0 | 1 | 0 | 0 | 0 | 0 |
|  | Training Set | Training Set | Training Set | Test Set | Training Set | Manna et al. (2003) Canc. Lett. 171-182 | 0 | 1 | 0 | 0 | 0 | 0 |
|  | Training Set | Training Set | Training Set | Training Set | Training Set | Manna et al. (2003) Canc. Lett. 171-182 | 0 | 1 | 0 | 0 | 0 | 0 |
|  | Training Set | Training Set | Training Set | Test Set | Training Set | Kang et al. (2003) J. Ethnopharm. 151-154 (structures) Park et al. (2007) Arch. Pharm. Res. (30) 755-760 | 0 | 1 | 0 | 0 | 0 | 0 |
|  | Training Set | Training Set | Training Set | Training Set | Training Set | Kang et al. (2003) J. Ethnopharm. 151-154 (structures) Park et al. (2007) Arch. Pharm. Res. (30) 755-760 | 0 | 1 | 0 | 0 | 0 | 0 |
|  | Training Set | Training Set | Test Set | Training Set | Training Set | Kang et al. (2003) J. Ethnopharm. 151-154 (structures) Park et al. (2007) Arch. Pharm. Res. (30) 755-760 | 0 | 1 | 0 | 0 | 0 | 0 |
|  | Training Set | Training Set | Training Set | Training Set | Training Set | Kang et al. (2003) J. Ethnopharm. 151-154 (structures) Park et al. (2007) Arch. Pharm. Res. (30) 755-760 | 0 | 1 | 0 | 0 | 0 | 0 |
|  | Test Set | Training Set | Training Set | Training Set | Training Set | Kang et al. (2003) J. Ethnopharm. 151-154 (structures) Park et al. (2007) Arch. Pharm. Res. (30) 755-760 | 0 | 1 | 0 | 0 | 0 | 0 |
|  | Training Set | Training Set | Training Set | Test Set | Training Set | Murata et al (2003) Bioorg. Med. Chem. Lett. (13) 913-918 | 1 | 0 | 0 | 0 | 0 | 0 |
|  | Test Set | Training Set | Training Set | Training Set | Training Set | Murata et al (2003) Bioorg. Med. Chem. Lett. (13) 913-918 | 1 | 0 | 0 | 0 | 0 | 0 |
|  | Training Set | Training Set | Training Set | Training Set | Training Set | Murata et al (2003) Bioorg. Med. Chem. Lett. (13) 913-918 | 1 | 0 | 0 | 0 | 0 | 0 |
|  | Test Set | Training Set | Training Set | Training Set | Training Set | Murata et al (2003) Bioorg. Med. Chem. Lett. (13) 913-918 | 1 | 0 | 0 | 0 | 0 | 0 |
|  | Training Set | Test Set | Training Set | Test Set | Training Set | Murata et al (2003) Bioorg. Med. Chem. Lett. (13) 913-918 | 1 | 0 | 0 | 0 | 0 | 0 |
|  | Training Set | Test Set | Test Set | Training Set | Test Set | Murata et al (2003) Bioorg. Med. Chem. Lett. (13) 913-918 | 1 | 0 | 0 | 0 | 0 | 0 |
|  | Training Set | Training Set | Training Set | Training Set | Training Set | Murata et al (2003) Bioorg. Med. Chem. Lett. (13) 913-918 | 1 | 0 | 0 | 0 | 0 | 0 |
|  | Training Set | Training Set | Test Set | Training Set | Training Set | Murata et al (2003) Bioorg. Med. Chem. Lett. (13) 913-918 | 1 | 0 | 0 | 0 | 0 | 0 |
|  | Training Set | Training Set | Training Set | Training Set | Training Set | Murata et al (2003) Bioorg. Med. Chem. Lett. (13) 913-918 | 1 | 0 | 0 | 0 | 0 | 0 |
|  | Training Set | Training Set | Training Set | Test Set | Training Set | Murata et al (2003) Bioorg. Med. Chem. Lett. (13) 913-918 | 1 | 0 | 0 | 0 | 0 | 0 |
|  | Training Set | Training Set | Test Set | Training Set | Training Set | Murata et al (2003) Bioorg. Med. Chem. Lett. (13) 913-918 | 1 | 0 | 0 | 0 | 0 | 0 |
|  | Test Set | Training Set | Training Set | Training Set | Training Set | Murata et al (2003) Bioorg. Med. Chem. Lett. (13) 913-918 | 1 | 0 | 0 | 0 | 0 | 0 |
|  | Test Set | Training Set | Training Set | Training Set | Training Set | Murata et al (2003) Bioorg. Med. Chem. Lett. (13) 913-918 | 1 | 0 | 0 | 0 | 0 | 0 |
|  | Training Set | Test Set | Test Set | Test Set | Training Set | Murata et al (2003) Bioorg. Med. Chem. Lett. (13) 913-918 | 1 | 0 | 0 | 0 | 0 | 0 |
|  | Training Set | Training Set | Training Set | Training Set | Training Set | Murata et al (2003) Bioorg. Med. Chem. Lett. (13) 913-918 | 1 | 0 | 0 | 0 | 0 | 0 |
|  | Training Set | Training Set | Training Set | Training Set | Training Set | Murata et al (2003) Bioorg. Med. Chem. Lett. (13) 913-918 | 1 | 0 | 0 | 0 | 0 | 0 |
|  | Training Set | Training Set | Training Set | Training Set | Test Set | Murata et al (2003) Bioorg. Med. Chem. Lett. (13) 913-918 | 1 | 0 | 0 | 0 | 0 | 0 |
|  | Training Set | Training Set | Test Set | Training Set | Training Set | Murata et al (2003) Bioorg. Med. Chem. Lett. (13) 913-918 | 1 | 0 | 0 | 0 | 0 | 0 |
|  | Training Set | Training Set | Training Set | Training Set | Training Set | Murata et al (2003) Bioorg. Med. Chem. Lett. (13) 913-918 | 1 | 0 | 0 | 0 | 0 | 0 |
|  | Training Set | Test Set | Training Set | Test Set | Training Set | Murata et al (2003) Bioorg. Med. Chem. Lett. (13) 913-918 | 1 | 0 | 0 | 0 | 0 | 0 |
|  | Test Set | Test Set | Training Set | Training Set | Test Set | Murata et al (2003) Bioorg. Med. Chem. Lett. (13) 913-918 | 1 | 0 | 0 | 0 | 0 | 0 |
|  | Training Set | Training Set | Training Set | Training Set | Training Set | Murata et al (2003) Bioorg. Med. Chem. Lett. (13) 913-918 | 1 | 0 | 0 | 0 | 0 | 0 |
|  | Test Set | Training Set | Test Set | Training Set | Training Set | Murata et al (2003) Bioorg. Med. Chem. Lett. (13) 913-918 | 1 | 0 | 0 | 0 | 0 | 0 |
|  | Training Set | Training Set | Training Set | Training Set | Training Set | Murata et al (2003) Bioorg. Med. Chem. Lett. (13) 913-918 | 1 | 0 | 0 | 0 | 0 | 0 |
|  | Training Set | Training Set | Training Set | Test Set | Training Set | Murata et al (2003) Bioorg. Med. Chem. Lett. (13) 913-918 | 1 | 0 | 0 | 0 | 0 | 0 |
|  | Test Set | Training Set | Test Set | Training Set | Training Set | Murata et al (2003) Bioorg. Med. Chem. Lett. (13) 913-918 | 1 | 0 | 0 | 0 | 0 | 0 |
|  | Training Set | Training Set | Training Set | Training Set | Training Set | Murata, T., et al. 2004. Bioorg. Med. Chem. Lett. 14, 4019. | 1 | 0 | 0 | 0 | 0 | 0 |
|  | Training Set | Training Set | Training Set | Test Set | Training Set | Murata, T., et al. 2004. Bioorg. Med. Chem. Lett. 14, 4019. | 1 | 0 | 0 | 0 | 0 | 0 |
|  | Training Set | Test Set | Training Set | Training Set | Training Set | Murata, T., et al. 2004. Bioorg. Med. Chem. Lett. 14, 4019. | 1 | 0 | 0 | 0 | 0 | 0 |
|  | Test Set | Test Set | Training Set | Training Set | Training Set | Murata, T., et al. 2004. Bioorg. Med. Chem. Lett. 14, 4019. | 1 | 0 | 0 | 0 | 0 | 0 |
|  | Training Set | Training Set | Training Set | Training Set | Training Set | Murata, T., et al. 2004. Bioorg. Med. Chem. Lett. 14, 4019. | 1 | 0 | 0 | 0 | 0 | 0 |
|  | Training Set | Training Set | Training Set | Training Set | Training Set | Murata, T., et al. 2004. Bioorg. Med. Chem. Lett. 14, 4019. | 1 | 0 | 0 | 0 | 0 | 0 |
|  | Training Set | Training Set | Training Set | Training Set | Test Set | Murata, T., et al. 2004. Bioorg. Med. Chem. Lett. 14, 4019. | 1 | 0 | 0 | 0 | 0 | 0 |
|  | Training Set | Training Set | Training Set | Training Set | Training Set | Murata, T., et al. 2004. Bioorg. Med. Chem. Lett. 14, 4019. | 1 | 0 | 0 | 0 | 0 | 0 |
|  | Training Set | Training Set | Test Set | Training Set | Training Set | Murata, T., et al. 2004. Bioorg. Med. Chem. Lett. 14, 4019. | 1 | 0 | 0 | 0 | 0 | 0 |
|  | Training Set | Training Set | Test Set | Training Set | Test Set | Murata, T., et al. 2004. Bioorg. Med. Chem. Lett. 14, 4019. | 1 | 0 | 0 | 0 | 0 | 0 |
|  | Training Set | Training Set | Training Set | Training Set | Training Set | Murata, T., et al. 2004. Bioorg. Med. Chem. Lett. 14, 4019. | 1 | 0 | 0 | 0 | 0 | 0 |
|  | Test Set | Training Set | Training Set | Test Set | Training Set | Murata, T., et al. 2004. Bioorg. Med. Chem. Lett. 14, 4019. | 1 | 0 | 0 | 0 | 0 | 0 |
|  | Training Set | Training Set | Training Set | Training Set | Training Set | Murata, T., et al. 2004. Bioorg. Med. Chem. Lett. 14, 4019. | 1 | 0 | 0 | 0 | 0 | 0 |
|  | Test Set | Test Set | Training Set | Test Set | Training Set | Murata, T., et al. 2004. Bioorg. Med. Chem. Lett. 14, 4019. | 1 | 0 | 0 | 0 | 0 | 0 |
|  | Test Set | Training Set | Training Set | Training Set | Training Set | Murata, T., et al. 2004. Bioorg. Med. Chem. Lett. 14, 4019. | 1 | 0 | 0 | 0 | 0 | 0 |
|  | Training Set | Test Set | Training Set | Test Set | Training Set | Murata, T., et al. 2004. Bioorg. Med. Chem. Lett. 14, 4019. | 1 | 0 | 0 | 0 | 0 | 0 |
|  | Training Set | Training Set | Training Set | Training Set | Test Set | Murata, T., et al. 2004. Bioorg. Med. Chem. Lett. 14, 4019. | 1 | 0 | 0 | 0 | 0 | 0 |
|  | Test Set | Training Set | Training Set | Training Set | Training Set | Murata, T., et al. 2004. Bioorg. Med. Chem. Lett. 14, 4019. | 1 | 0 | 0 | 0 | 0 | 0 |
|  | Training Set | Training Set | Training Set | Training Set | Training Set | Murata, T., et al. 2004. Bioorg. Med. Chem. Lett. 14, 4019. | 1 | 0 | 0 | 0 | 0 | 0 |
|  | Training Set | Training Set | Test Set | Training Set | Training Set | Murata, T., et al. 2004. Bioorg. Med. Chem. Lett. 14, 4019. | 1 | 0 | 0 | 0 | 0 | 0 |
|  | Training Set | Test Set | Test Set | Training Set | Training Set | Murata, T., et al. 2004. Bioorg. Med. Chem. Lett. 14, 4019. | 1 | 0 | 0 | 0 | 0 | 0 |
|  | Test Set | Training Set | Training Set | Training Set | Training Set | Murata, T., et al. 2004. Bioorg. Med. Chem. Lett. 14, 4019. | 1 | 0 | 0 | 0 | 0 | 0 |
|  | Training Set | Training Set | Training Set | Training Set | Test Set | Murata, T., et al. 2004. Bioorg. Med. Chem. Lett. 14, 4019. | 1 | 0 | 0 | 0 | 0 | 0 |
|  | Test Set | Test Set | Test Set | Training Set | Training Set | Murata, T., et al. 2004. Bioorg. Med. Chem. Lett. 14, 4019. | 1 | 0 | 0 | 0 | 0 | 0 |
|  | Training Set | Training Set | Training Set | Training Set | Training Set | Murata, T., et al. 2004. Bioorg. Med. Chem. Lett. 14, 4019. | 1 | 0 | 0 | 0 | 0 | 0 |
|  | Training Set | Training Set | Test Set | Training Set | Training Set | Murata, T., et al. 2004. Bioorg. Med. Chem. Lett. 14, 4019. | 1 | 0 | 0 | 0 | 0 | 0 |
|  | Training Set | Training Set | Test Set | Test Set | Training Set | Murata, T., et al. 2004. Bioorg. Med. Chem. Lett. 14, 4019. | 1 | 0 | 0 | 0 | 0 | 0 |
|  | Test Set | Training Set | Training Set | Training Set | Test Set | Murata, T., et al. 2004. Bioorg. Med. Chem. Lett. 14, 4019. | 1 | 0 | 0 | 0 | 0 | 0 |
|  | Test Set | Training Set | Training Set | Training Set | Training Set | Murata, T., et al. 2004. Bioorg. Med. Chem. Lett. 14, 4019. | 1 | 0 | 0 | 0 | 0 | 0 |
|  | Test Set | Training Set | Training Set | Training Set | Test Set | Murata, T., et al. 2004. Bioorg. Med. Chem. Lett. 14, 4019. | 1 | 0 | 0 | 0 | 0 | 0 |
|  | Training Set | Test Set | Training Set | Training Set | Training Set | Natarajan et al (1998) Arch. Biochem & Biophys (352) 59-70 | 0 | 1 | 0 | 0 | 0 | 0 |
|  | Training Set | Training Set | Training Set | Training Set | Training Set | Sancho et al. (2002) Eur. J. Immunol. (32) 1753-1763 | 0 | 0 | 0 | 0 | 0 | 0 |
|  | Training Set | Training Set | Training Set | Training Set | Training Set | Sancho et al. (2002) Eur. J. Immunol. (32) 1753-1763 | 0 | 0 | 0 | 0 | 0 | 0 |
|  | Training Set | Training Set | Training Set | Training Set | Training Set | Sancho et al. (2002) Eur. J. Immunol. (32) 1753-1763 | 0 | 0 | 0 | 0 | 0 | 0 |
|  | Training Set | Training Set | Training Set | Training Set | Training Set | Singh et al. (1996) J Immunology (157) 4412-4420 | 0 | 0 | 0 | 0 | 0 | 0 |
|  | Training Set | Training Set | Training Set | Training Set | Training Set | Swinney et al. (2002) J Bio Chem (277) 23573-23581 | 0 | 1 | 0 | 0 | 0 | 0 |
|  | Training Set | Test Set | Test Set | Training Set | Training Set | Swinney et al. (2002) J Bio Chem (277) 23573-23581 | 0 | 1 | 0 | 0 | 0 | 0 |
|  | Training Set | Test Set | Training Set | Training Set | Training Set | Yore et al. (2006) Mol Cancer Ther (5) 3232-3239 | 1 | 0 | 0 | 0 | 0 | 0 |
|  | Test Set | Training Set | Test Set | Training Set | Test Set | Yore et al. (2006) Mol Cancer Ther (5) 3232-3239 | 1 | 0 | 0 | 0 | 0 | 0 |
|  | Training Set | Training Set | Training Set | Training Set | Training Set | Umezawa et al. (2000) Anti-Can Drug Des (15) 239-244 | 0 | 0 | 0 | 0 | 0 | 0 |
|  | Training Set | Training Set | Training Set | Training Set | Training Set | Keum et al. (2003) Mut. Res. 75-85 Pande & Ramos (2005) Curr. Med. Chem. (12) 357-374 | 0 | 0 | 0 | 0 | 0 | 0 |
|  | Training Set | Training Set | Training Set | Training Set | Training Set | Hirano et al. (1998) Immunopharm. (39) 31-38 Pande & Ramos (2005) Curr. Med. Chem. (12) 357-374 | 0 | 0 | 0 | 0 | 0 | 0 |
|  | Training Set | Training Set | Training Set | Training Set | Training Set | Cominacini et al. (1998) J. Hypertension (16) 1913 Pande & Ramos (2005) Curr. Med. Chem. (12) 357-374 | 0 | 0 | 0 | 0 | 0 | 0 |
|  | Training Set | Training Set | Training Set | Training Set | Training Set | Altavilla et al. (2000) Card. Res. (47) 515-528 Pande & Ramos (2005) Curr. Med. Chem. (12) 357-374 | 0 | 0 | 0 | 0 | 0 | 0 |
|  | Training Set | Test Set | Training Set | Training Set | Training Set | Hehner et al. (1998) J. Bio. Chem. (273) 1288-1297 Pande & Ramos (2005) Curr. Med. Chem. (12) 357-374 | 0 | 1 | 0 | 0 | 0 | 0 |
|  | Training Set | Test Set | Test Set | Training Set | Training Set | Hehner et al. (1998) J. Bio. Chem. (273) 1288-1297 Pande & Ramos (2005) Curr. Med. Chem. (12) 357-374 | 0 | 1 | 0 | 0 | 0 | 0 |
|  | Training Set | Test Set | Training Set | Training Set | Training Set | Schorr et al. (2002) Phytochem (60) 733-740 Pande & Ramos (2005) Curr. Med. Chem. (12) 357-374 | 0 | 0 | 0 | 1 | 0 | 0 |
|  | Training Set | Training Set | Training Set | Training Set | Training Set | Schorr et al. (2002) Phytochem (60) 733-740 Pande & Ramos (2005) Curr. Med. Chem. (12) 357-374 | 0 | 0 | 0 | 1 | 0 | 0 |
|  | Training Set | Training Set | Training Set | Training Set | Training Set | Schorr et al. (2002) Phytochem (60) 733-740 Pande & Ramos (2005) Curr. Med. Chem. (12) 357-374 | 0 | 0 | 0 | 1 | 0 | 0 |
|  | Training Set | Training Set | Training Set | Training Set | Test Set | Schmitz & Ecker (2008) Progress in Lipid Research (47) 147-155 Singer et al. (2008) Int. Care Med. (34) 1580-1592 Novak et al. (2002) Am. J. Physiol. Cell Mol. Physiol. (284) 84-89 Weldon et al. (2007) J. Nut. Biochem (18) 250-258 | 1 | 0 | 0 | 0 | 0 | 0 |
|  | Training Set | Training Set | Test Set | Training Set | Test Set | Schmitz & Ecker (2008) Progress in Lipid Research (47) 147-155 Singer et al. (2008) Int. Care Med. (34) 1580-1592 Novak et al. (2002) Am. J. Physiol. Cell Mol. Physiol. (284) 84-89 Weldon et al. (2007) J. Nut. Biochem (18) 250-258 | 1 | 0 | 0 | 0 | 0 | 0 |
|  | Training Set | Training Set | Training Set | Training Set | Training Set | Ramakers et al. (2007) Lipids (42) 687-698 | 0 | 0 | 0 | 0 | 0 | 0 |
|  | Training Set | Training Set | Training Set | Training Set | Training Set | Loscher et al. (2005) J. Immun. (175) 4990-4998 | 0 | 0 | 0 | 0 | 0 | 0 |
|  | Training Set | Training Set | Training Set | Training Set | Training Set | Olivier et al. (2006\_ Mol. Pharm. (69) 1615-1623 Calzado et al. (2007) Curr. Med. Chem. (14) 367-376 | 0 | 0 | 0 | 0 | 0 | 0 |
|  | Training Set | Test Set | Training Set | Training Set | Training Set | Saklatavala (2007) Curr. Drug Targ. (8) 305-313 | 0 | 1 | 0 | 0 | 0 | 0 |
