# Supplementary figures and images for: Predicting the points of interaction of small molecules in the NF-κB pathway (part 2 of 6)
Source: BMC Syst Biol. 2011 Feb 22;5:32. doi: 10.1186/1752-0509-5-32 (PMC3050742; doi:10.1186/1752-0509-5-32)

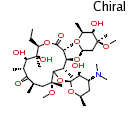

Supplement: Additional file 1 — List of Compounds that interact with NF-kB. [file 1752-0509-5-32-S1.ZIP › Additional Files 1/Additional Files 1_files/image29835.png]

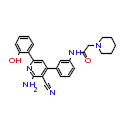

Supplement: Additional file 1 — List of Compounds that interact with NF-kB. [file 1752-0509-5-32-S1.ZIP › Additional Files 1/Additional Files 1_files/image29836.png]

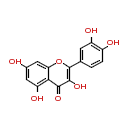

Supplement: Additional file 1 — List of Compounds that interact with NF-kB. [file 1752-0509-5-32-S1.ZIP › Additional Files 1/Additional Files 1_files/image29837.png]

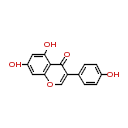

Supplement: Additional file 1 — List of Compounds that interact with NF-kB. [file 1752-0509-5-32-S1.ZIP › Additional Files 1/Additional Files 1_files/image29838.png]

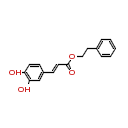

Supplement: Additional file 1 — List of Compounds that interact with NF-kB. [file 1752-0509-5-32-S1.ZIP › Additional Files 1/Additional Files 1_files/image29839.png]

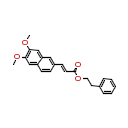

Supplement: Additional file 1 — List of Compounds that interact with NF-kB. [file 1752-0509-5-32-S1.ZIP › Additional Files 1/Additional Files 1_files/image29840.png]

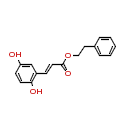

Supplement: Additional file 1 — List of Compounds that interact with NF-kB. [file 1752-0509-5-32-S1.ZIP › Additional Files 1/Additional Files 1_files/image29841.png]

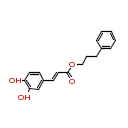

Supplement: Additional file 1 — List of Compounds that interact with NF-kB. [file 1752-0509-5-32-S1.ZIP › Additional Files 1/Additional Files 1_files/image29842.png]

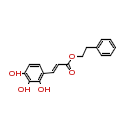

Supplement: Additional file 1 — List of Compounds that interact with NF-kB. [file 1752-0509-5-32-S1.ZIP › Additional Files 1/Additional Files 1_files/image29843.png]

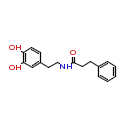

Supplement: Additional file 1 — List of Compounds that interact with NF-kB. [file 1752-0509-5-32-S1.ZIP › Additional Files 1/Additional Files 1_files/image29844.png]

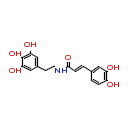

Supplement: Additional file 1 — List of Compounds that interact with NF-kB. [file 1752-0509-5-32-S1.ZIP › Additional Files 1/Additional Files 1_files/image29845.png]

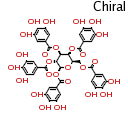

Supplement: Additional file 1 — List of Compounds that interact with NF-kB. [file 1752-0509-5-32-S1.ZIP › Additional Files 1/Additional Files 1_files/image29846.png]

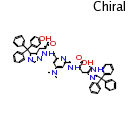

Supplement: Additional file 1 — List of Compounds that interact with NF-kB. [file 1752-0509-5-32-S1.ZIP › Additional Files 1/Additional Files 1_files/image29847.png]

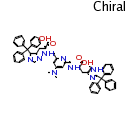

Supplement: Additional file 1 — List of Compounds that interact with NF-kB. [file 1752-0509-5-32-S1.ZIP › Additional Files 1/Additional Files 1_files/image29848.png]

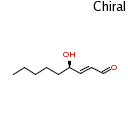

Supplement: Additional file 1 — List of Compounds that interact with NF-kB. [file 1752-0509-5-32-S1.ZIP › Additional Files 1/Additional Files 1_files/image29849.png]

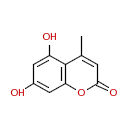

Supplement: Additional file 1 — List of Compounds that interact with NF-kB. [file 1752-0509-5-32-S1.ZIP › Additional Files 1/Additional Files 1_files/image29850.png]

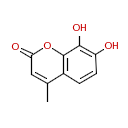

Supplement: Additional file 1 — List of Compounds that interact with NF-kB. [file 1752-0509-5-32-S1.ZIP › Additional Files 1/Additional Files 1_files/image29851.png]

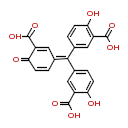

Supplement: Additional file 1 — List of Compounds that interact with NF-kB. [file 1752-0509-5-32-S1.ZIP › Additional Files 1/Additional Files 1_files/image29852.png]

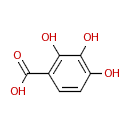

Supplement: Additional file 1 — List of Compounds that interact with NF-kB. [file 1752-0509-5-32-S1.ZIP › Additional Files 1/Additional Files 1_files/image29853.png]

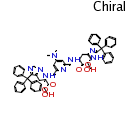

Supplement: Additional file 1 — List of Compounds that interact with NF-kB. [file 1752-0509-5-32-S1.ZIP › Additional Files 1/Additional Files 1_files/image29854.png]

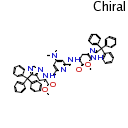

Supplement: Additional file 1 — List of Compounds that interact with NF-kB. [file 1752-0509-5-32-S1.ZIP › Additional Files 1/Additional Files 1_files/image29855.png]

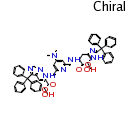

Supplement: Additional file 1 — List of Compounds that interact with NF-kB. [file 1752-0509-5-32-S1.ZIP › Additional Files 1/Additional Files 1_files/image29856.png]

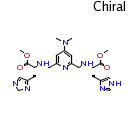

Supplement: Additional file 1 — List of Compounds that interact with NF-kB. [file 1752-0509-5-32-S1.ZIP › Additional Files 1/Additional Files 1_files/image29857.png]

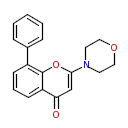

Supplement: Additional file 1 — List of Compounds that interact with NF-kB. [file 1752-0509-5-32-S1.ZIP › Additional Files 1/Additional Files 1_files/image29858.png]

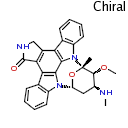

Supplement: Additional file 1 — List of Compounds that interact with NF-kB. [file 1752-0509-5-32-S1.ZIP › Additional Files 1/Additional Files 1_files/image29859.png]

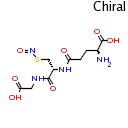

Supplement: Additional file 1 — List of Compounds that interact with NF-kB. [file 1752-0509-5-32-S1.ZIP › Additional Files 1/Additional Files 1_files/image29860.png]

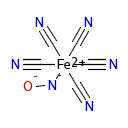

Supplement: Additional file 1 — List of Compounds that interact with NF-kB. [file 1752-0509-5-32-S1.ZIP › Additional Files 1/Additional Files 1_files/image29861.png]

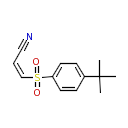

Supplement: Additional file 1 — List of Compounds that interact with NF-kB. [file 1752-0509-5-32-S1.ZIP › Additional Files 1/Additional Files 1_files/image29862.png]

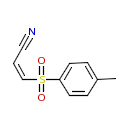

Supplement: Additional file 1 — List of Compounds that interact with NF-kB. [file 1752-0509-5-32-S1.ZIP › Additional Files 1/Additional Files 1_files/image29863.png]

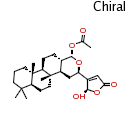

Supplement: Additional file 1 — List of Compounds that interact with NF-kB. [file 1752-0509-5-32-S1.ZIP › Additional Files 1/Additional Files 1_files/image29864.png]

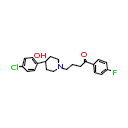

Supplement: Additional file 1 — List of Compounds that interact with NF-kB. [file 1752-0509-5-32-S1.ZIP › Additional Files 1/Additional Files 1_files/image29865.png]

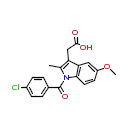

Supplement: Additional file 1 — List of Compounds that interact with NF-kB. [file 1752-0509-5-32-S1.ZIP › Additional Files 1/Additional Files 1_files/image29866.png]

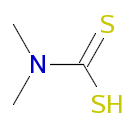

Supplement: Additional file 1 — List of Compounds that interact with NF-kB. [file 1752-0509-5-32-S1.ZIP › Additional Files 1/Additional Files 1_files/image29867.png]

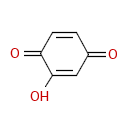

Supplement: Additional file 1 — List of Compounds that interact with NF-kB. [file 1752-0509-5-32-S1.ZIP › Additional Files 1/Additional Files 1_files/image29868.png]

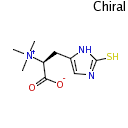

Supplement: Additional file 1 — List of Compounds that interact with NF-kB. [file 1752-0509-5-32-S1.ZIP › Additional Files 1/Additional Files 1_files/image29869.png]

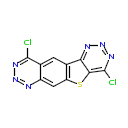

Supplement: Additional file 1 — List of Compounds that interact with NF-kB. [file 1752-0509-5-32-S1.ZIP › Additional Files 1/Additional Files 1_files/image29870.png]

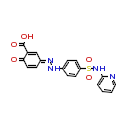

Supplement: Additional file 1 — List of Compounds that interact with NF-kB. [file 1752-0509-5-32-S1.ZIP › Additional Files 1/Additional Files 1_files/image29871.png]

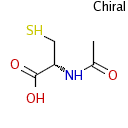

Supplement: Additional file 1 — List of Compounds that interact with NF-kB. [file 1752-0509-5-32-S1.ZIP › Additional Files 1/Additional Files 1_files/image29872.png]

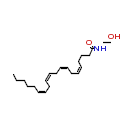

Supplement: Additional file 1 — List of Compounds that interact with NF-kB. [file 1752-0509-5-32-S1.ZIP › Additional Files 1/Additional Files 1_files/image29873.png]

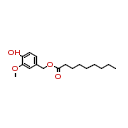

Supplement: Additional file 1 — List of Compounds that interact with NF-kB. [file 1752-0509-5-32-S1.ZIP › Additional Files 1/Additional Files 1_files/image29874.png]

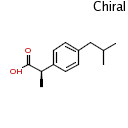

Supplement: Additional file 1 — List of Compounds that interact with NF-kB. [file 1752-0509-5-32-S1.ZIP › Additional Files 1/Additional Files 1_files/image29875.png]

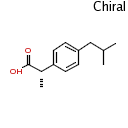

Supplement: Additional file 1 — List of Compounds that interact with NF-kB. [file 1752-0509-5-32-S1.ZIP › Additional Files 1/Additional Files 1_files/image29876.png]

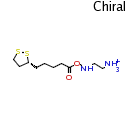

Supplement: Additional file 1 — List of Compounds that interact with NF-kB. [file 1752-0509-5-32-S1.ZIP › Additional Files 1/Additional Files 1_files/image29877.png]

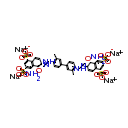

Supplement: Additional file 1 — List of Compounds that interact with NF-kB. [file 1752-0509-5-32-S1.ZIP › Additional Files 1/Additional Files 1_files/image29878.png]

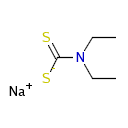

Supplement: Additional file 1 — List of Compounds that interact with NF-kB. [file 1752-0509-5-32-S1.ZIP › Additional Files 1/Additional Files 1_files/image29879.png]

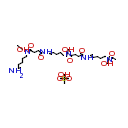

Supplement: Additional file 1 — List of Compounds that interact with NF-kB. [file 1752-0509-5-32-S1.ZIP › Additional Files 1/Additional Files 1_files/image29880.png]

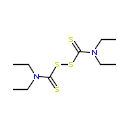

Supplement: Additional file 1 — List of Compounds that interact with NF-kB. [file 1752-0509-5-32-S1.ZIP › Additional Files 1/Additional Files 1_files/image29881.png]

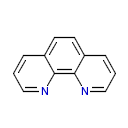

Supplement: Additional file 1 — List of Compounds that interact with NF-kB. [file 1752-0509-5-32-S1.ZIP › Additional Files 1/Additional Files 1_files/image29882.png]

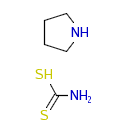

Supplement: Additional file 1 — List of Compounds that interact with NF-kB. [file 1752-0509-5-32-S1.ZIP › Additional Files 1/Additional Files 1_files/image29883.png]

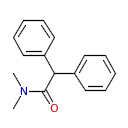

Supplement: Additional file 1 — List of Compounds that interact with NF-kB. [file 1752-0509-5-32-S1.ZIP › Additional Files 1/Additional Files 1_files/image29884.png]

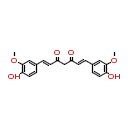

Supplement: Additional file 1 — List of Compounds that interact with NF-kB. [file 1752-0509-5-32-S1.ZIP › Additional Files 1/Additional Files 1_files/image29885.png]

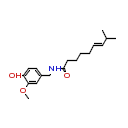

Supplement: Additional file 1 — List of Compounds that interact with NF-kB. [file 1752-0509-5-32-S1.ZIP › Additional Files 1/Additional Files 1_files/image29886.png]

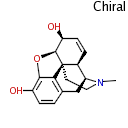

Supplement: Additional file 1 — List of Compounds that interact with NF-kB. [file 1752-0509-5-32-S1.ZIP › Additional Files 1/Additional Files 1_files/image29887.png]

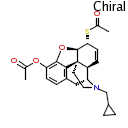

Supplement: Additional file 1 — List of Compounds that interact with NF-kB. [file 1752-0509-5-32-S1.ZIP › Additional Files 1/Additional Files 1_files/image29888.png]

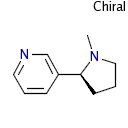

Supplement: Additional file 1 — List of Compounds that interact with NF-kB. [file 1752-0509-5-32-S1.ZIP › Additional Files 1/Additional Files 1_files/image29889.png]

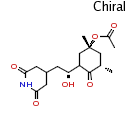

Supplement: Additional file 1 — List of Compounds that interact with NF-kB. [file 1752-0509-5-32-S1.ZIP › Additional Files 1/Additional Files 1_files/image29890.png]

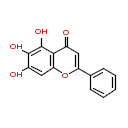

Supplement: Additional file 1 — List of Compounds that interact with NF-kB. [file 1752-0509-5-32-S1.ZIP › Additional Files 1/Additional Files 1_files/image29891.png]

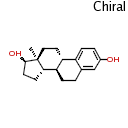

Supplement: Additional file 1 — List of Compounds that interact with NF-kB. [file 1752-0509-5-32-S1.ZIP › Additional Files 1/Additional Files 1_files/image29892.png]

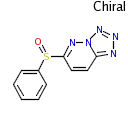

Supplement: Additional file 1 — List of Compounds that interact with NF-kB. [file 1752-0509-5-32-S1.ZIP › Additional Files 1/Additional Files 1_files/image29893.png]

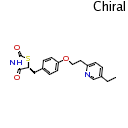

Supplement: Additional file 1 — List of Compounds that interact with NF-kB. [file 1752-0509-5-32-S1.ZIP › Additional Files 1/Additional Files 1_files/image29894.png]

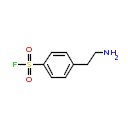

Supplement: Additional file 1 — List of Compounds that interact with NF-kB. [file 1752-0509-5-32-S1.ZIP › Additional Files 1/Additional Files 1_files/image29895.png]

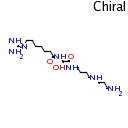

Supplement: Additional file 1 — List of Compounds that interact with NF-kB. [file 1752-0509-5-32-S1.ZIP › Additional Files 1/Additional Files 1_files/image29896.png]

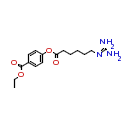

Supplement: Additional file 1 — List of Compounds that interact with NF-kB. [file 1752-0509-5-32-S1.ZIP › Additional Files 1/Additional Files 1_files/image29897.png]

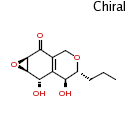

Supplement: Additional file 1 — List of Compounds that interact with NF-kB. [file 1752-0509-5-32-S1.ZIP › Additional Files 1/Additional Files 1_files/image29898.png]

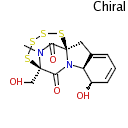

Supplement: Additional file 1 — List of Compounds that interact with NF-kB. [file 1752-0509-5-32-S1.ZIP › Additional Files 1/Additional Files 1_files/image29899.png]

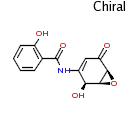

Supplement: Additional file 1 — List of Compounds that interact with NF-kB. [file 1752-0509-5-32-S1.ZIP › Additional Files 1/Additional Files 1_files/image29900.png]

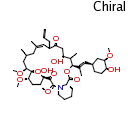

Supplement: Additional file 1 — List of Compounds that interact with NF-kB. [file 1752-0509-5-32-S1.ZIP › Additional Files 1/Additional Files 1_files/image29901.png]

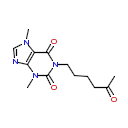

Supplement: Additional file 1 — List of Compounds that interact with NF-kB. [file 1752-0509-5-32-S1.ZIP › Additional Files 1/Additional Files 1_files/image29902.png]

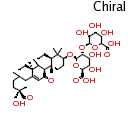

Supplement: Additional file 1 — List of Compounds that interact with NF-kB. [file 1752-0509-5-32-S1.ZIP › Additional Files 1/Additional Files 1_files/image29903.png]

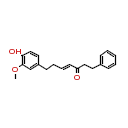

Supplement: Additional file 1 — List of Compounds that interact with NF-kB. [file 1752-0509-5-32-S1.ZIP › Additional Files 1/Additional Files 1_files/image29904.png]

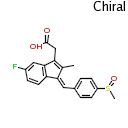

Supplement: Additional file 1 — List of Compounds that interact with NF-kB. [file 1752-0509-5-32-S1.ZIP › Additional Files 1/Additional Files 1_files/image29905.png]

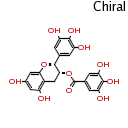

Supplement: Additional file 1 — List of Compounds that interact with NF-kB. [file 1752-0509-5-32-S1.ZIP › Additional Files 1/Additional Files 1_files/image29906.png]

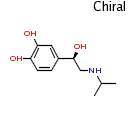

Supplement: Additional file 1 — List of Compounds that interact with NF-kB. [file 1752-0509-5-32-S1.ZIP › Additional Files 1/Additional Files 1_files/image29907.png]

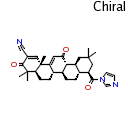

Supplement: Additional file 1 — List of Compounds that interact with NF-kB. [file 1752-0509-5-32-S1.ZIP › Additional Files 1/Additional Files 1_files/image29908.png]

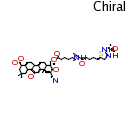

Supplement: Additional file 1 — List of Compounds that interact with NF-kB. [file 1752-0509-5-32-S1.ZIP › Additional Files 1/Additional Files 1_files/image29909.png]

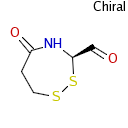

Supplement: Additional file 1 — List of Compounds that interact with NF-kB. [file 1752-0509-5-32-S1.ZIP › Additional Files 1/Additional Files 1_files/image29910.png]

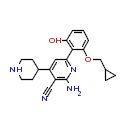

Supplement: Additional file 1 — List of Compounds that interact with NF-kB. [file 1752-0509-5-32-S1.ZIP › Additional Files 1/Additional Files 1_files/image29911.png]

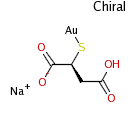

Supplement: Additional file 1 — List of Compounds that interact with NF-kB. [file 1752-0509-5-32-S1.ZIP › Additional Files 1/Additional Files 1_files/image29912.png]

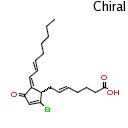

Supplement: Additional file 1 — List of Compounds that interact with NF-kB. [file 1752-0509-5-32-S1.ZIP › Additional Files 1/Additional Files 1_files/image29913.png]

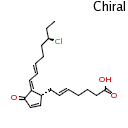

Supplement: Additional file 1 — List of Compounds that interact with NF-kB. [file 1752-0509-5-32-S1.ZIP › Additional Files 1/Additional Files 1_files/image29914.png]

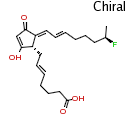

Supplement: Additional file 1 — List of Compounds that interact with NF-kB. [file 1752-0509-5-32-S1.ZIP › Additional Files 1/Additional Files 1_files/image29915.png]

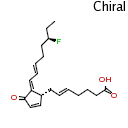

Supplement: Additional file 1 — List of Compounds that interact with NF-kB. [file 1752-0509-5-32-S1.ZIP › Additional Files 1/Additional Files 1_files/image29916.png]

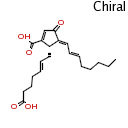

Supplement: Additional file 1 — List of Compounds that interact with NF-kB. [file 1752-0509-5-32-S1.ZIP › Additional Files 1/Additional Files 1_files/image29917.png]

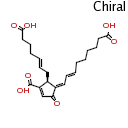

Supplement: Additional file 1 — List of Compounds that interact with NF-kB. [file 1752-0509-5-32-S1.ZIP › Additional Files 1/Additional Files 1_files/image29918.png]

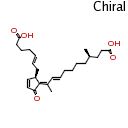

Supplement: Additional file 1 — List of Compounds that interact with NF-kB. [file 1752-0509-5-32-S1.ZIP › Additional Files 1/Additional Files 1_files/image29919.png]

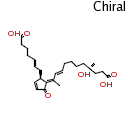

Supplement: Additional file 1 — List of Compounds that interact with NF-kB. [file 1752-0509-5-32-S1.ZIP › Additional Files 1/Additional Files 1_files/image29920.png]

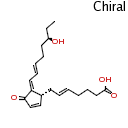

Supplement: Additional file 1 — List of Compounds that interact with NF-kB. [file 1752-0509-5-32-S1.ZIP › Additional Files 1/Additional Files 1_files/image29921.png]

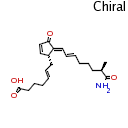

Supplement: Additional file 1 — List of Compounds that interact with NF-kB. [file 1752-0509-5-32-S1.ZIP › Additional Files 1/Additional Files 1_files/image29922.png]

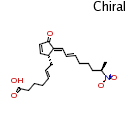

Supplement: Additional file 1 — List of Compounds that interact with NF-kB. [file 1752-0509-5-32-S1.ZIP › Additional Files 1/Additional Files 1_files/image29923.png]

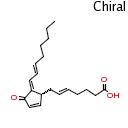

Supplement: Additional file 1 — List of Compounds that interact with NF-kB. [file 1752-0509-5-32-S1.ZIP › Additional Files 1/Additional Files 1_files/image29924.png]

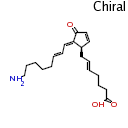

Supplement: Additional file 1 — List of Compounds that interact with NF-kB. [file 1752-0509-5-32-S1.ZIP › Additional Files 1/Additional Files 1_files/image29925.png]

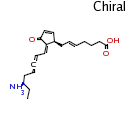

Supplement: Additional file 1 — List of Compounds that interact with NF-kB. [file 1752-0509-5-32-S1.ZIP › Additional Files 1/Additional Files 1_files/image29926.png]

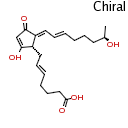

Supplement: Additional file 1 — List of Compounds that interact with NF-kB. [file 1752-0509-5-32-S1.ZIP › Additional Files 1/Additional Files 1_files/image29927.png]

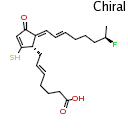

Supplement: Additional file 1 — List of Compounds that interact with NF-kB. [file 1752-0509-5-32-S1.ZIP › Additional Files 1/Additional Files 1_files/image29928.png]

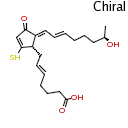

Supplement: Additional file 1 — List of Compounds that interact with NF-kB. [file 1752-0509-5-32-S1.ZIP › Additional Files 1/Additional Files 1_files/image29929.png]

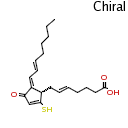

Supplement: Additional file 1 — List of Compounds that interact with NF-kB. [file 1752-0509-5-32-S1.ZIP › Additional Files 1/Additional Files 1_files/image29930.png]

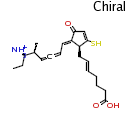

Supplement: Additional file 1 — List of Compounds that interact with NF-kB. [file 1752-0509-5-32-S1.ZIP › Additional Files 1/Additional Files 1_files/image29931.png]

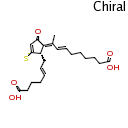

Supplement: Additional file 1 — List of Compounds that interact with NF-kB. [file 1752-0509-5-32-S1.ZIP › Additional Files 1/Additional Files 1_files/image29932.png]

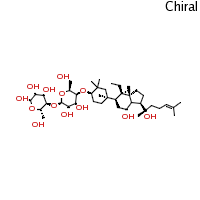

Supplement: Additional file 2 — Compounds clustered using ECFP_4 and Property Descriptors. [file 1752-0509-5-32-S2.ZIP › Additional Files 2/Additional Files 2_files/image36998.png]

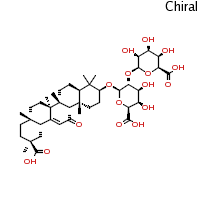

Supplement: Additional file 2 — Compounds clustered using ECFP_4 and Property Descriptors. [file 1752-0509-5-32-S2.ZIP › Additional Files 2/Additional Files 2_files/image36999.png]
