# Supplementary figures and images for: Predicting the points of interaction of small molecules in the NF-κB pathway (part 1 of 6)
Source: BMC Syst Biol. 2011 Feb 22;5:32. doi: 10.1186/1752-0509-5-32 (PMC3050742; doi:10.1186/1752-0509-5-32)

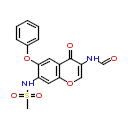

Supplement: Additional file 1 — List of Compounds that interact with NF-kB. [file 1752-0509-5-32-S1.ZIP › Additional Files 1/Additional Files 1_files/image29734.png]

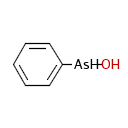

Supplement: Additional file 1 — List of Compounds that interact with NF-kB. [file 1752-0509-5-32-S1.ZIP › Additional Files 1/Additional Files 1_files/image29735.png]

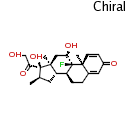

Supplement: Additional file 1 — List of Compounds that interact with NF-kB. [file 1752-0509-5-32-S1.ZIP › Additional Files 1/Additional Files 1_files/image29736.png]

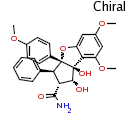

Supplement: Additional file 1 — List of Compounds that interact with NF-kB. [file 1752-0509-5-32-S1.ZIP › Additional Files 1/Additional Files 1_files/image29737.png]

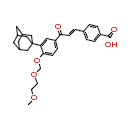

Supplement: Additional file 1 — List of Compounds that interact with NF-kB. [file 1752-0509-5-32-S1.ZIP › Additional Files 1/Additional Files 1_files/image29738.png]

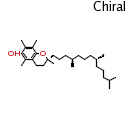

Supplement: Additional file 1 — List of Compounds that interact with NF-kB. [file 1752-0509-5-32-S1.ZIP › Additional Files 1/Additional Files 1_files/image29739.png]

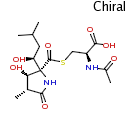

Supplement: Additional file 1 — List of Compounds that interact with NF-kB. [file 1752-0509-5-32-S1.ZIP › Additional Files 1/Additional Files 1_files/image29740.png]

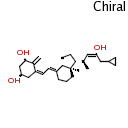

Supplement: Additional file 1 — List of Compounds that interact with NF-kB. [file 1752-0509-5-32-S1.ZIP › Additional Files 1/Additional Files 1_files/image29741.png]

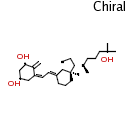

Supplement: Additional file 1 — List of Compounds that interact with NF-kB. [file 1752-0509-5-32-S1.ZIP › Additional Files 1/Additional Files 1_files/image29742.png]

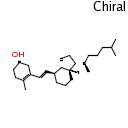

Supplement: Additional file 1 — List of Compounds that interact with NF-kB. [file 1752-0509-5-32-S1.ZIP › Additional Files 1/Additional Files 1_files/image29743.png]

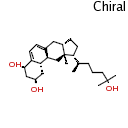

Supplement: Additional file 1 — List of Compounds that interact with NF-kB. [file 1752-0509-5-32-S1.ZIP › Additional Files 1/Additional Files 1_files/image29744.png]

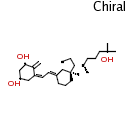

Supplement: Additional file 1 — List of Compounds that interact with NF-kB. [file 1752-0509-5-32-S1.ZIP › Additional Files 1/Additional Files 1_files/image29745.png]

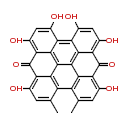

Supplement: Additional file 1 — List of Compounds that interact with NF-kB. [file 1752-0509-5-32-S1.ZIP › Additional Files 1/Additional Files 1_files/image29746.png]

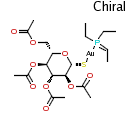

Supplement: Additional file 1 — List of Compounds that interact with NF-kB. [file 1752-0509-5-32-S1.ZIP › Additional Files 1/Additional Files 1_files/image29747.png]

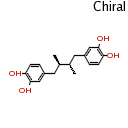

Supplement: Additional file 1 — List of Compounds that interact with NF-kB. [file 1752-0509-5-32-S1.ZIP › Additional Files 1/Additional Files 1_files/image29748.png]

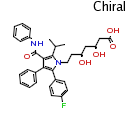

Supplement: Additional file 1 — List of Compounds that interact with NF-kB. [file 1752-0509-5-32-S1.ZIP › Additional Files 1/Additional Files 1_files/image29749.png]

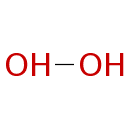

Supplement: Additional file 1 — List of Compounds that interact with NF-kB. [file 1752-0509-5-32-S1.ZIP › Additional Files 1/Additional Files 1_files/image29750.png]

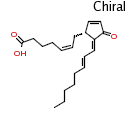

Supplement: Additional file 1 — List of Compounds that interact with NF-kB. [file 1752-0509-5-32-S1.ZIP › Additional Files 1/Additional Files 1_files/image29751.png]

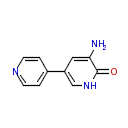

Supplement: Additional file 1 — List of Compounds that interact with NF-kB. [file 1752-0509-5-32-S1.ZIP › Additional Files 1/Additional Files 1_files/image29752.png]

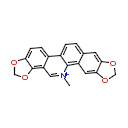

Supplement: Additional file 1 — List of Compounds that interact with NF-kB. [file 1752-0509-5-32-S1.ZIP › Additional Files 1/Additional Files 1_files/image29753.png]

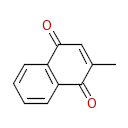

Supplement: Additional file 1 — List of Compounds that interact with NF-kB. [file 1752-0509-5-32-S1.ZIP › Additional Files 1/Additional Files 1_files/image29754.png]

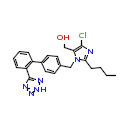

Supplement: Additional file 1 — List of Compounds that interact with NF-kB. [file 1752-0509-5-32-S1.ZIP › Additional Files 1/Additional Files 1_files/image29755.png]

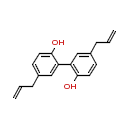

Supplement: Additional file 1 — List of Compounds that interact with NF-kB. [file 1752-0509-5-32-S1.ZIP › Additional Files 1/Additional Files 1_files/image29756.png]

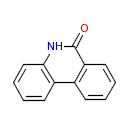

Supplement: Additional file 1 — List of Compounds that interact with NF-kB. [file 1752-0509-5-32-S1.ZIP › Additional Files 1/Additional Files 1_files/image29757.png]

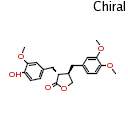

Supplement: Additional file 1 — List of Compounds that interact with NF-kB. [file 1752-0509-5-32-S1.ZIP › Additional Files 1/Additional Files 1_files/image29758.png]

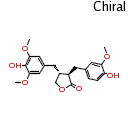

Supplement: Additional file 1 — List of Compounds that interact with NF-kB. [file 1752-0509-5-32-S1.ZIP › Additional Files 1/Additional Files 1_files/image29759.png]

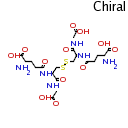

Supplement: Additional file 1 — List of Compounds that interact with NF-kB. [file 1752-0509-5-32-S1.ZIP › Additional Files 1/Additional Files 1_files/image29760.png]

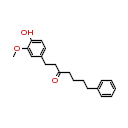

Supplement: Additional file 1 — List of Compounds that interact with NF-kB. [file 1752-0509-5-32-S1.ZIP › Additional Files 1/Additional Files 1_files/image29761.png]

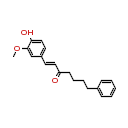

Supplement: Additional file 1 — List of Compounds that interact with NF-kB. [file 1752-0509-5-32-S1.ZIP › Additional Files 1/Additional Files 1_files/image29762.png]

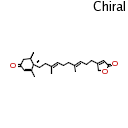

Supplement: Additional file 1 — List of Compounds that interact with NF-kB. [file 1752-0509-5-32-S1.ZIP › Additional Files 1/Additional Files 1_files/image29763.png]

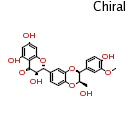

Supplement: Additional file 1 — List of Compounds that interact with NF-kB. [file 1752-0509-5-32-S1.ZIP › Additional Files 1/Additional Files 1_files/image29764.png]

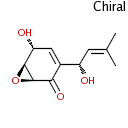

Supplement: Additional file 1 — List of Compounds that interact with NF-kB. [file 1752-0509-5-32-S1.ZIP › Additional Files 1/Additional Files 1_files/image29765.png]

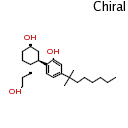

Supplement: Additional file 1 — List of Compounds that interact with NF-kB. [file 1752-0509-5-32-S1.ZIP › Additional Files 1/Additional Files 1_files/image29766.png]

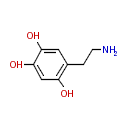

Supplement: Additional file 1 — List of Compounds that interact with NF-kB. [file 1752-0509-5-32-S1.ZIP › Additional Files 1/Additional Files 1_files/image29767.png]

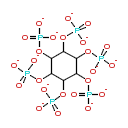

Supplement: Additional file 1 — List of Compounds that interact with NF-kB. [file 1752-0509-5-32-S1.ZIP › Additional Files 1/Additional Files 1_files/image29768.png]

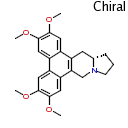

Supplement: Additional file 1 — List of Compounds that interact with NF-kB. [file 1752-0509-5-32-S1.ZIP › Additional Files 1/Additional Files 1_files/image29769.png]

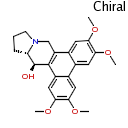

Supplement: Additional file 1 — List of Compounds that interact with NF-kB. [file 1752-0509-5-32-S1.ZIP › Additional Files 1/Additional Files 1_files/image29770.png]

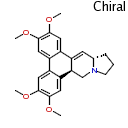

Supplement: Additional file 1 — List of Compounds that interact with NF-kB. [file 1752-0509-5-32-S1.ZIP › Additional Files 1/Additional Files 1_files/image29771.png]

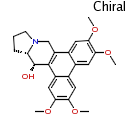

Supplement: Additional file 1 — List of Compounds that interact with NF-kB. [file 1752-0509-5-32-S1.ZIP › Additional Files 1/Additional Files 1_files/image29772.png]

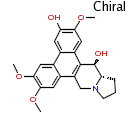

Supplement: Additional file 1 — List of Compounds that interact with NF-kB. [file 1752-0509-5-32-S1.ZIP › Additional Files 1/Additional Files 1_files/image29773.png]

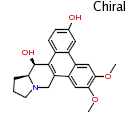

Supplement: Additional file 1 — List of Compounds that interact with NF-kB. [file 1752-0509-5-32-S1.ZIP › Additional Files 1/Additional Files 1_files/image29774.png]

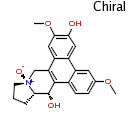

Supplement: Additional file 1 — List of Compounds that interact with NF-kB. [file 1752-0509-5-32-S1.ZIP › Additional Files 1/Additional Files 1_files/image29775.png]

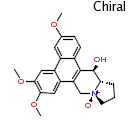

Supplement: Additional file 1 — List of Compounds that interact with NF-kB. [file 1752-0509-5-32-S1.ZIP › Additional Files 1/Additional Files 1_files/image29776.png]

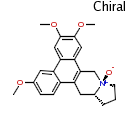

Supplement: Additional file 1 — List of Compounds that interact with NF-kB. [file 1752-0509-5-32-S1.ZIP › Additional Files 1/Additional Files 1_files/image29777.png]

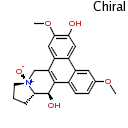

Supplement: Additional file 1 — List of Compounds that interact with NF-kB. [file 1752-0509-5-32-S1.ZIP › Additional Files 1/Additional Files 1_files/image29779.png]

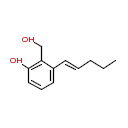

Supplement: Additional file 1 — List of Compounds that interact with NF-kB. [file 1752-0509-5-32-S1.ZIP › Additional Files 1/Additional Files 1_files/image29780.png]

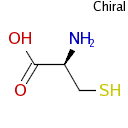

Supplement: Additional file 1 — List of Compounds that interact with NF-kB. [file 1752-0509-5-32-S1.ZIP › Additional Files 1/Additional Files 1_files/image29781.png]

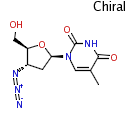

Supplement: Additional file 1 — List of Compounds that interact with NF-kB. [file 1752-0509-5-32-S1.ZIP › Additional Files 1/Additional Files 1_files/image29782.png]

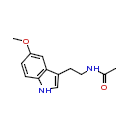

Supplement: Additional file 1 — List of Compounds that interact with NF-kB. [file 1752-0509-5-32-S1.ZIP › Additional Files 1/Additional Files 1_files/image29783.png]

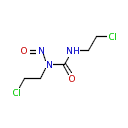

Supplement: Additional file 1 — List of Compounds that interact with NF-kB. [file 1752-0509-5-32-S1.ZIP › Additional Files 1/Additional Files 1_files/image29784.png]

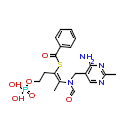

Supplement: Additional file 1 — List of Compounds that interact with NF-kB. [file 1752-0509-5-32-S1.ZIP › Additional Files 1/Additional Files 1_files/image29785.png]

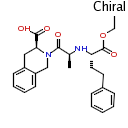

Supplement: Additional file 1 — List of Compounds that interact with NF-kB. [file 1752-0509-5-32-S1.ZIP › Additional Files 1/Additional Files 1_files/image29786.png]

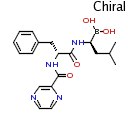

Supplement: Additional file 1 — List of Compounds that interact with NF-kB. [file 1752-0509-5-32-S1.ZIP › Additional Files 1/Additional Files 1_files/image29787.png]

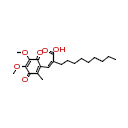

Supplement: Additional file 1 — List of Compounds that interact with NF-kB. [file 1752-0509-5-32-S1.ZIP › Additional Files 1/Additional Files 1_files/image29788.png]

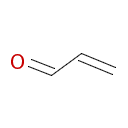

Supplement: Additional file 1 — List of Compounds that interact with NF-kB. [file 1752-0509-5-32-S1.ZIP › Additional Files 1/Additional Files 1_files/image29789.png]

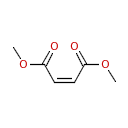

Supplement: Additional file 1 — List of Compounds that interact with NF-kB. [file 1752-0509-5-32-S1.ZIP › Additional Files 1/Additional Files 1_files/image29790.png]

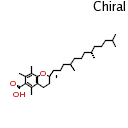

Supplement: Additional file 1 — List of Compounds that interact with NF-kB. [file 1752-0509-5-32-S1.ZIP › Additional Files 1/Additional Files 1_files/image29791.png]

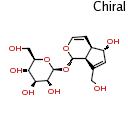

Supplement: Additional file 1 — List of Compounds that interact with NF-kB. [file 1752-0509-5-32-S1.ZIP › Additional Files 1/Additional Files 1_files/image29792.png]

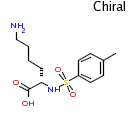

Supplement: Additional file 1 — List of Compounds that interact with NF-kB. [file 1752-0509-5-32-S1.ZIP › Additional Files 1/Additional Files 1_files/image29793.png]

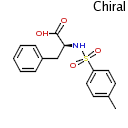

Supplement: Additional file 1 — List of Compounds that interact with NF-kB. [file 1752-0509-5-32-S1.ZIP › Additional Files 1/Additional Files 1_files/image29794.png]

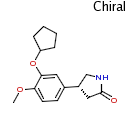

Supplement: Additional file 1 — List of Compounds that interact with NF-kB. [file 1752-0509-5-32-S1.ZIP › Additional Files 1/Additional Files 1_files/image29795.png]

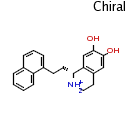

Supplement: Additional file 1 — List of Compounds that interact with NF-kB. [file 1752-0509-5-32-S1.ZIP › Additional Files 1/Additional Files 1_files/image29796.png]

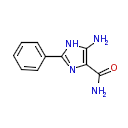

Supplement: Additional file 1 — List of Compounds that interact with NF-kB. [file 1752-0509-5-32-S1.ZIP › Additional Files 1/Additional Files 1_files/image29797.png]

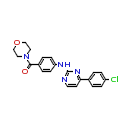

Supplement: Additional file 1 — List of Compounds that interact with NF-kB. [file 1752-0509-5-32-S1.ZIP › Additional Files 1/Additional Files 1_files/image29798.png]

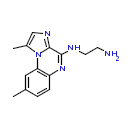

Supplement: Additional file 1 — List of Compounds that interact with NF-kB. [file 1752-0509-5-32-S1.ZIP › Additional Files 1/Additional Files 1_files/image29799.png]

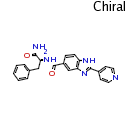

Supplement: Additional file 1 — List of Compounds that interact with NF-kB. [file 1752-0509-5-32-S1.ZIP › Additional Files 1/Additional Files 1_files/image29800.png]

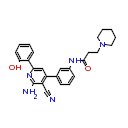

Supplement: Additional file 1 — List of Compounds that interact with NF-kB. [file 1752-0509-5-32-S1.ZIP › Additional Files 1/Additional Files 1_files/image29801.png]

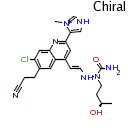

Supplement: Additional file 1 — List of Compounds that interact with NF-kB. [file 1752-0509-5-32-S1.ZIP › Additional Files 1/Additional Files 1_files/image29802.png]

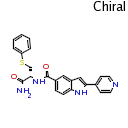

Supplement: Additional file 1 — List of Compounds that interact with NF-kB. [file 1752-0509-5-32-S1.ZIP › Additional Files 1/Additional Files 1_files/image29803.png]

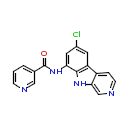

Supplement: Additional file 1 — List of Compounds that interact with NF-kB. [file 1752-0509-5-32-S1.ZIP › Additional Files 1/Additional Files 1_files/image29804.png]

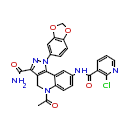

Supplement: Additional file 1 — List of Compounds that interact with NF-kB. [file 1752-0509-5-32-S1.ZIP › Additional Files 1/Additional Files 1_files/image29805.png]

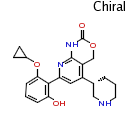

Supplement: Additional file 1 — List of Compounds that interact with NF-kB. [file 1752-0509-5-32-S1.ZIP › Additional Files 1/Additional Files 1_files/image29806.png]

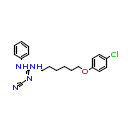

Supplement: Additional file 1 — List of Compounds that interact with NF-kB. [file 1752-0509-5-32-S1.ZIP › Additional Files 1/Additional Files 1_files/image29807.png]

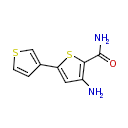

Supplement: Additional file 1 — List of Compounds that interact with NF-kB. [file 1752-0509-5-32-S1.ZIP › Additional Files 1/Additional Files 1_files/image29808.png]

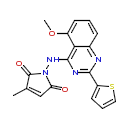

Supplement: Additional file 1 — List of Compounds that interact with NF-kB. [file 1752-0509-5-32-S1.ZIP › Additional Files 1/Additional Files 1_files/image29809.png]

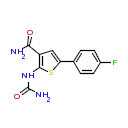

Supplement: Additional file 1 — List of Compounds that interact with NF-kB. [file 1752-0509-5-32-S1.ZIP › Additional Files 1/Additional Files 1_files/image29810.png]

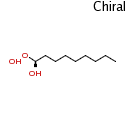

Supplement: Additional file 1 — List of Compounds that interact with NF-kB. [file 1752-0509-5-32-S1.ZIP › Additional Files 1/Additional Files 1_files/image29811.png]

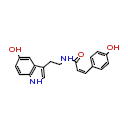

Supplement: Additional file 1 — List of Compounds that interact with NF-kB. [file 1752-0509-5-32-S1.ZIP › Additional Files 1/Additional Files 1_files/image29812.png]

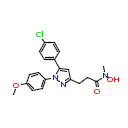

Supplement: Additional file 1 — List of Compounds that interact with NF-kB. [file 1752-0509-5-32-S1.ZIP › Additional Files 1/Additional Files 1_files/image29813.png]

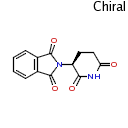

Supplement: Additional file 1 — List of Compounds that interact with NF-kB. [file 1752-0509-5-32-S1.ZIP › Additional Files 1/Additional Files 1_files/image29814.png]

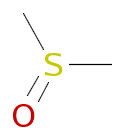

Supplement: Additional file 1 — List of Compounds that interact with NF-kB. [file 1752-0509-5-32-S1.ZIP › Additional Files 1/Additional Files 1_files/image29815.png]

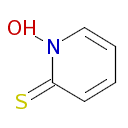

Supplement: Additional file 1 — List of Compounds that interact with NF-kB. [file 1752-0509-5-32-S1.ZIP › Additional Files 1/Additional Files 1_files/image29816.png]

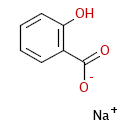

Supplement: Additional file 1 — List of Compounds that interact with NF-kB. [file 1752-0509-5-32-S1.ZIP › Additional Files 1/Additional Files 1_files/image29817.png]

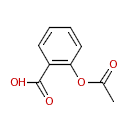

Supplement: Additional file 1 — List of Compounds that interact with NF-kB. [file 1752-0509-5-32-S1.ZIP › Additional Files 1/Additional Files 1_files/image29818.png]

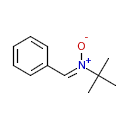

Supplement: Additional file 1 — List of Compounds that interact with NF-kB. [file 1752-0509-5-32-S1.ZIP › Additional Files 1/Additional Files 1_files/image29819.png]

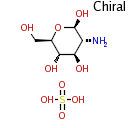

Supplement: Additional file 1 — List of Compounds that interact with NF-kB. [file 1752-0509-5-32-S1.ZIP › Additional Files 1/Additional Files 1_files/image29820.png]

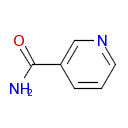

Supplement: Additional file 1 — List of Compounds that interact with NF-kB. [file 1752-0509-5-32-S1.ZIP › Additional Files 1/Additional Files 1_files/image29821.png]

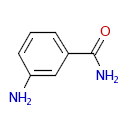

Supplement: Additional file 1 — List of Compounds that interact with NF-kB. [file 1752-0509-5-32-S1.ZIP › Additional Files 1/Additional Files 1_files/image29822.png]

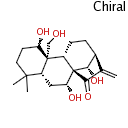

Supplement: Additional file 1 — List of Compounds that interact with NF-kB. [file 1752-0509-5-32-S1.ZIP › Additional Files 1/Additional Files 1_files/image29823.png]

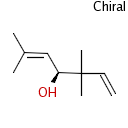

Supplement: Additional file 1 — List of Compounds that interact with NF-kB. [file 1752-0509-5-32-S1.ZIP › Additional Files 1/Additional Files 1_files/image29824.png]

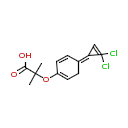

Supplement: Additional file 1 — List of Compounds that interact with NF-kB. [file 1752-0509-5-32-S1.ZIP › Additional Files 1/Additional Files 1_files/image29825.png]

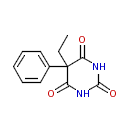

Supplement: Additional file 1 — List of Compounds that interact with NF-kB. [file 1752-0509-5-32-S1.ZIP › Additional Files 1/Additional Files 1_files/image29826.png]

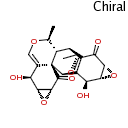

Supplement: Additional file 1 — List of Compounds that interact with NF-kB. [file 1752-0509-5-32-S1.ZIP › Additional Files 1/Additional Files 1_files/image29827.png]

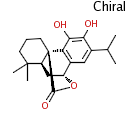

Supplement: Additional file 1 — List of Compounds that interact with NF-kB. [file 1752-0509-5-32-S1.ZIP › Additional Files 1/Additional Files 1_files/image29828.png]

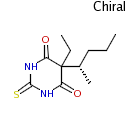

Supplement: Additional file 1 — List of Compounds that interact with NF-kB. [file 1752-0509-5-32-S1.ZIP › Additional Files 1/Additional Files 1_files/image29829.png]

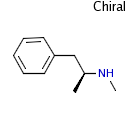

Supplement: Additional file 1 — List of Compounds that interact with NF-kB. [file 1752-0509-5-32-S1.ZIP › Additional Files 1/Additional Files 1_files/image29830.png]

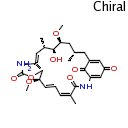

Supplement: Additional file 1 — List of Compounds that interact with NF-kB. [file 1752-0509-5-32-S1.ZIP › Additional Files 1/Additional Files 1_files/image29831.png]

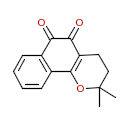

Supplement: Additional file 1 — List of Compounds that interact with NF-kB. [file 1752-0509-5-32-S1.ZIP › Additional Files 1/Additional Files 1_files/image29832.png]

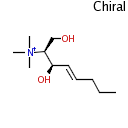

Supplement: Additional file 1 — List of Compounds that interact with NF-kB. [file 1752-0509-5-32-S1.ZIP › Additional Files 1/Additional Files 1_files/image29833.png]

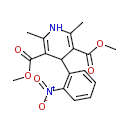

Supplement: Additional file 1 — List of Compounds that interact with NF-kB. [file 1752-0509-5-32-S1.ZIP › Additional Files 1/Additional Files 1_files/image29834.png]
