# Supplementary material for: Predicting the points of interaction of small molecules in the NF-κB pathway
Source: BMC Syst Biol. 2011 Feb 22;5:32. doi: 10.1186/1752-0509-5-32 (PMC3050742; doi:10.1186/1752-0509-5-32)
Supplement: Additional file 2 — Compounds clustered using ECFP_4 and Property Descriptors. [file 1752-0509-5-32-S2.ZIP › Additional Files 2/Additional Files 2.htm]

 

|  |
| --- |
| Clusters |
| |  |  |  |  |  |  |  |  |  |  |  |  |  |  |  |  |  |  |  |  |  |  |  |  |  |  |  |  |  | | --- | --- | --- | --- | --- | --- | --- | --- | --- | --- | --- | --- | --- | --- | --- | --- | --- | --- | --- | --- | --- | --- | --- | --- | --- | --- | --- | --- | --- | | |  | | --- | | Cluster: 1 | | |  |  |  |  |  |  |  |  |  |  |  |  |  |  |  | | --- | --- | --- | --- | --- | --- | --- | --- | --- | --- | --- | --- | --- | --- | --- | | |  |  | | --- | --- | | |  | | --- | |  | | | |  |  | | --- | --- | | |  | | --- | |  | | | |  |  | | --- | --- | | |  | | --- | |  | | | |  |  | | --- | --- | | |  | | --- | |  | | | |  |  | | --- | --- | | |  | | --- | |  | | | | |  |  | | --- | --- | | |  | | --- | |  | | | |  |  | | --- | --- | | |  | | --- | |  | | | |  |  | | --- | --- | | |  | | --- | |  | | |  |  | | | |
| |  |  |  |  |  |  |  |  |  |  |  |  |  |  |  |  |  |  |  |  |  |  |  |  |  |  |  | | --- | --- | --- | --- | --- | --- | --- | --- | --- | --- | --- | --- | --- | --- | --- | --- | --- | --- | --- | --- | --- | --- | --- | --- | --- | --- | --- | | |  | | --- | | Cluster: 2 | | |  |  |  |  |  |  |  |  |  |  |  |  |  |  |  | | --- | --- | --- | --- | --- | --- | --- | --- | --- | --- | --- | --- | --- | --- | --- | | |  |  | | --- | --- | | |  | | --- | |  | | | |  |  | | --- | --- | | |  | | --- | |  | | | |  |  | | --- | --- | | |  | | --- | |  | | | |  |  | | --- | --- | | |  | | --- | |  | | | |  |  | | --- | --- | | |  | | --- | |  | | | | |  |  | | --- | --- | | |  | | --- | |  | | | |  |  | | --- | --- | | |  | | --- | |  | | |  |  |  | | | |
| |  |  |  |  |  |  |  |  |  |  |  |  | | --- | --- | --- | --- | --- | --- | --- | --- | --- | --- | --- | --- | | |  | | --- | | Cluster: 3 | | |  |  |  |  |  |  |  |  |  | | --- | --- | --- | --- | --- | --- | --- | --- | --- | | |  |  | | --- | --- | | |  | | --- | |  | | | |  |  | | --- | --- | | |  | | --- | |  | | |  |  |  | | | |
| |  |  |  |  |  |  |  |  |  |  |  |  |  |  | | --- | --- | --- | --- | --- | --- | --- | --- | --- | --- | --- | --- | --- | --- | | |  | | --- | | Cluster: 4 | | |  |  |  |  |  |  |  |  |  |  |  | | --- | --- | --- | --- | --- | --- | --- | --- | --- | --- | --- | | |  |  | | --- | --- | | |  | | --- | |  | | | |  |  | | --- | --- | | |  | | --- | |  | | | |  |  | | --- | --- | | |  | | --- | |  | | |  |  | | | |
| |  |  |  |  |  |  |  |  |  |  |  |  |  |  |  |  |  |  |  |  |  |  |  |  |  |  |  |  |  |  |  | | --- | --- | --- | --- | --- | --- | --- | --- | --- | --- | --- | --- | --- | --- | --- | --- | --- | --- | --- | --- | --- | --- | --- | --- | --- | --- | --- | --- | --- | --- | --- | | |  | | --- | | Cluster: 5 | | |  |  |  |  |  |  |  |  |  |  |  |  |  |  |  | | --- | --- | --- | --- | --- | --- | --- | --- | --- | --- | --- | --- | --- | --- | --- | | |  |  | | --- | --- | | |  | | --- | |  | | | |  |  | | --- | --- | | |  | | --- | |  | | | |  |  | | --- | --- | | |  | | --- | |  | | | |  |  | | --- | --- | | |  | | --- | |  | | | |  |  | | --- | --- | | |  | | --- | |  | | | | |  |  | | --- | --- | | |  | | --- | |  | | | |  |  | | --- | --- | | |  | | --- | |  | | | |  |  | | --- | --- | | |  | | --- | |  | | | |  |  | | --- | --- | | |  | | --- | |  | | |  | | | |
| |  |  |  |  |  |  |  |  |  |  | | --- | --- | --- | --- | --- | --- | --- | --- | --- | --- | | |  | | --- | | Cluster: 6 | | |  |  |  |  |  |  |  | | --- | --- | --- | --- | --- | --- | --- | | |  |  | | --- | --- | | |  | | --- | |  | | |  |  |  |  | | | |
| |  |  |  |  |  |  |  |  |  |  | | --- | --- | --- | --- | --- | --- | --- | --- | --- | --- | | |  | | --- | | Cluster: 7 | | |  |  |  |  |  |  |  | | --- | --- | --- | --- | --- | --- | --- | | |  |  | | --- | --- | | |  | | --- | |  | | |  |  |  |  | | | |
| |  |  |  |  |  |  |  |  |  |  |  |  |  |  | | --- | --- | --- | --- | --- | --- | --- | --- | --- | --- | --- | --- | --- | --- | | |  | | --- | | Cluster: 8 | | |  |  |  |  |  |  |  |  |  |  |  | | --- | --- | --- | --- | --- | --- | --- | --- | --- | --- | --- | | |  |  | | --- | --- | | |  | | --- | |  | | | |  |  | | --- | --- | | |  | | --- | |  | | | |  |  | | --- | --- | | |  | | --- | |  | | |  |  | | | |
| |  |  |  |  |  |  |  |  |  |  |  |  |  |  | | --- | --- | --- | --- | --- | --- | --- | --- | --- | --- | --- | --- | --- | --- | | |  | | --- | | Cluster: 9 | | |  |  |  |  |  |  |  |  |  |  |  | | --- | --- | --- | --- | --- | --- | --- | --- | --- | --- | --- | | |  |  | | --- | --- | | |  | | --- | |  | | | |  |  | | --- | --- | | |  | | --- | |  | | | |  |  | | --- | --- | | |  | | --- | |  | | |  |  | | | |
| |  |  |  |  |  |  |  |  |  |  |  |  |  |  | | --- | --- | --- | --- | --- | --- | --- | --- | --- | --- | --- | --- | --- | --- | | |  | | --- | | Cluster: 10 | | |  |  |  |  |  |  |  |  |  |  |  | | --- | --- | --- | --- | --- | --- | --- | --- | --- | --- | --- | | |  |  | | --- | --- | | |  | | --- | |  | | | |  |  | | --- | --- | | |  | | --- | |  | | | |  |  | | --- | --- | | |  | | --- | |  | | |  |  | | | |
| |  |  |  |  |  |  |  |  |  |  |  |  |  |  | | --- | --- | --- | --- | --- | --- | --- | --- | --- | --- | --- | --- | --- | --- | | |  | | --- | | Cluster: 11 | | |  |  |  |  |  |  |  |  |  |  |  | | --- | --- | --- | --- | --- | --- | --- | --- | --- | --- | --- | | |  |  | | --- | --- | | |  | | --- | |  | | | |  |  | | --- | --- | | |  | | --- | |  | | | |  |  | | --- | --- | | |  | | --- | |  | | |  |  | | | |
| |  |  |  |  |  |  |  |  |  |  |  |  |  |  |  |  |  |  |  |  |  |  |  |  |  |  |  |  |  |  |  | | --- | --- | --- | --- | --- | --- | --- | --- | --- | --- | --- | --- | --- | --- | --- | --- | --- | --- | --- | --- | --- | --- | --- | --- | --- | --- | --- | --- | --- | --- | --- | | |  | | --- | | Cluster: 12 | | |  |  |  |  |  |  |  |  |  |  |  |  |  |  |  | | --- | --- | --- | --- | --- | --- | --- | --- | --- | --- | --- | --- | --- | --- | --- | | |  |  | | --- | --- | | |  | | --- | |  | | | |  |  | | --- | --- | | |  | | --- | |  | | | |  |  | | --- | --- | | |  | | --- | |  | | | |  |  | | --- | --- | | |  | | --- | |  | | | |  |  | | --- | --- | | |  | | --- | |  | | | | |  |  | | --- | --- | | |  | | --- | |  | | | |  |  | | --- | --- | | |  | | --- | |  | | | |  |  | | --- | --- | | |  | | --- | |  | | | |  |  | | --- | --- | | |  | | --- | |  | | |  | | | |
| |  |  |  |  |  |  |  |  |  |  |  |  |  |  |  |  |  |  |  |  |  |  |  |  |  |  |  |  |  |  |  |  |  |  |  |  |  |  |  |  | | --- | --- | --- | --- | --- | --- | --- | --- | --- | --- | --- | --- | --- | --- | --- | --- | --- | --- | --- | --- | --- | --- | --- | --- | --- | --- | --- | --- | --- | --- | --- | --- | --- | --- | --- | --- | --- | --- | --- | --- | | |  | | --- | | Cluster: 13 | | |  |  |  |  |  |  |  |  |  |  |  |  |  |  |  | | --- | --- | --- | --- | --- | --- | --- | --- | --- | --- | --- | --- | --- | --- | --- | | |  |  | | --- | --- | | |  | | --- | |  | | | |  |  | | --- | --- | | |  | | --- | |  | | | |  |  | | --- | --- | | |  | | --- | |  | | | |  |  | | --- | --- | | |  | | --- | |  | | | |  |  | | --- | --- | | |  | | --- | |  | | | | |  |  | | --- | --- | | |  | | --- | |  | | | |  |  | | --- | --- | | |  | | --- | |  | | | |  |  | | --- | --- | | |  | | --- | |  | | | |  |  | | --- | --- | | |  | | --- | |  | | | |  |  | | --- | --- | | |  | | --- | |  | | | | |  |  | | --- | --- | | |  | | --- | |  | | |  |  |  |  | | | |
| |  |  |  |  |  |  |  |  |  |  |  |  |  |  |  |  |  |  |  |  |  |  |  |  |  |  |  |  |  |  |  |  |  |  |  |  |  |  |  |  |  |  |  |  | | --- | --- | --- | --- | --- | --- | --- | --- | --- | --- | --- | --- | --- | --- | --- | --- | --- | --- | --- | --- | --- | --- | --- | --- | --- | --- | --- | --- | --- | --- | --- | --- | --- | --- | --- | --- | --- | --- | --- | --- | --- | --- | --- | --- | | |  | | --- | | Cluster: 14 | | |  |  |  |  |  |  |  |  |  |  |  |  |  |  |  | | --- | --- | --- | --- | --- | --- | --- | --- | --- | --- | --- | --- | --- | --- | --- | | |  |  | | --- | --- | | |  | | --- | |  | | | |  |  | | --- | --- | | |  | | --- | |  | | | |  |  | | --- | --- | | |  | | --- | |  | | | |  |  | | --- | --- | | |  | | --- | |  | | | |  |  | | --- | --- | | |  | | --- | |  | | | | |  |  | | --- | --- | | |  | | --- | |  | | | |  |  | | --- | --- | | |  | | --- | |  | | | |  |  | | --- | --- | | |  | | --- | |  | | | |  |  | | --- | --- | | |  | | --- | |  | | | |  |  | | --- | --- | | |  | | --- | |  | | | | |  |  | | --- | --- | | |  | | --- | |  | | | |  |  | | --- | --- | | |  | | --- | |  | | | |  |  | | --- | --- | | |  | | --- | |  | | |  |  | | | |
| |  |  |  |  |  |  |  |  |  |  |  |  |  |  |  |  |  |  | | --- | --- | --- | --- | --- | --- | --- | --- | --- | --- | --- | --- | --- | --- | --- | --- | --- | --- | | |  | | --- | | Cluster: 15 | | |  |  |  |  |  |  |  |  |  |  |  |  |  |  |  | | --- | --- | --- | --- | --- | --- | --- | --- | --- | --- | --- | --- | --- | --- | --- | | |  |  | | --- | --- | | |  | | --- | |  | | | |  |  | | --- | --- | | |  | | --- | |  | | | |  |  | | --- | --- | | |  | | --- | |  | | | |  |  | | --- | --- | | |  | | --- | |  | | | |  |  | | --- | --- | | |  | | --- | |  | | | | | |
| |  |  |  |  |  |  |  |  |  |  | | --- | --- | --- | --- | --- | --- | --- | --- | --- | --- | | |  | | --- | | Cluster: 16 | | |  |  |  |  |  |  |  | | --- | --- | --- | --- | --- | --- | --- | | |  |  | | --- | --- | | |  | | --- | |  | | |  |  |  |  | | | |
| |  |  |  |  |  |  |  |  |  |  | | --- | --- | --- | --- | --- | --- | --- | --- | --- | --- | | |  | | --- | | Cluster: 17 | | |  |  |  |  |  |  |  | | --- | --- | --- | --- | --- | --- | --- | | |  |  | | --- | --- | | |  | | --- | |  | | |  |  |  |  | | | |
| |  |  |  |  |  |  |  |  |  |  | | --- | --- | --- | --- | --- | --- | --- | --- | --- | --- | | |  | | --- | | Cluster: 18 | | |  |  |  |  |  |  |  | | --- | --- | --- | --- | --- | --- | --- | | |  |  | | --- | --- | | |  | | --- | |  | | |  |  |  |  | | | |
| |  |  |  |  |  |  |  |  |  |  | | --- | --- | --- | --- | --- | --- | --- | --- | --- | --- | | |  | | --- | | Cluster: 19 | | |  |  |  |  |  |  |  | | --- | --- | --- | --- | --- | --- | --- | | |  |  | | --- | --- | | |  | | --- | |  | | |  |  |  |  | | | |
| |  |  |  |  |  |  |  |  |  |  |  |  |  |  |  |  |  |  |  |  |  |  |  |  |  |  |  |  |  |  |  | | --- | --- | --- | --- | --- | --- | --- | --- | --- | --- | --- | --- | --- | --- | --- | --- | --- | --- | --- | --- | --- | --- | --- | --- | --- | --- | --- | --- | --- | --- | --- | | |  | | --- | | Cluster: 20 | | |  |  |  |  |  |  |  |  |  |  |  |  |  |  |  | | --- | --- | --- | --- | --- | --- | --- | --- | --- | --- | --- | --- | --- | --- | --- | | |  |  | | --- | --- | | |  | | --- | |  | | | |  |  | | --- | --- | | |  | | --- | |  | | | |  |  | | --- | --- | | |  | | --- | |  | | | |  |  | | --- | --- | | |  | | --- | |  | | | |  |  | | --- | --- | | |  | | --- | |  | | | | |  |  | | --- | --- | | |  | | --- | |  | | | |  |  | | --- | --- | | |  | | --- | |  | | | |  |  | | --- | --- | | |  | | --- | |  | | | |  |  | | --- | --- | | |  | | --- | |  | | |  | | | |
| |  |  |  |  |  |  |  |  |  |  |  |  |  |  |  |  |  |  |  |  |  |  |  |  |  |  |  |  |  |  |  |  |  |  |  |  |  |  |  |  |  |  |  |  |  |  |  |  |  |  |  |  |  |  |  |  |  |  |  |  |  |  |  | | --- | --- | --- | --- | --- | --- | --- | --- | --- | --- | --- | --- | --- | --- | --- | --- | --- | --- | --- | --- | --- | --- | --- | --- | --- | --- | --- | --- | --- | --- | --- | --- | --- | --- | --- | --- | --- | --- | --- | --- | --- | --- | --- | --- | --- | --- | --- | --- | --- | --- | --- | --- | --- | --- | --- | --- | --- | --- | --- | --- | --- | --- | --- | | |  | | --- | | Cluster: 21 | | |  |  |  |  |  |  |  |  |  |  |  |  |  |  |  | | --- | --- | --- | --- | --- | --- | --- | --- | --- | --- | --- | --- | --- | --- | --- | | |  |  | | --- | --- | | |  | | --- | |  | | | |  |  | | --- | --- | | |  | | --- | |  | | | |  |  | | --- | --- | | |  | | --- | |  | | | |  |  | | --- | --- | | |  | | --- | |  | | | |  |  | | --- | --- | | |  | | --- | |  | | | | |  |  | | --- | --- | | |  | | --- | |  | | | |  |  | | --- | --- | | |  | | --- | |  | | | |  |  | | --- | --- | | |  | | --- | |  | | | |  |  | | --- | --- | | |  | | --- | |  | | | |  |  | | --- | --- | | |  | | --- | |  | | | | |  |  | | --- | --- | | |  | | --- | |  | | | |  |  | | --- | --- | | |  | | --- | |  | | | |  |  | | --- | --- | | |  | | --- | |  | | | |  |  | | --- | --- | | |  | | --- | |  | | | |  |  | | --- | --- | | |  | | --- | |  | | | | |  |  | | --- | --- | | |  | | --- | |  | | | |  |  | | --- | --- | | |  | | --- | |  | | | |  |  | | --- | --- | | |  | | --- | |  | | | |  |  | | --- | --- | | |  | | --- | |  | | | |  |  | | --- | --- | | |  | | --- | |  | | | | | |
| |  |  |  |  |  |  |  |  |  |  | | --- | --- | --- | --- | --- | --- | --- | --- | --- | --- | | |  | | --- | | Cluster: 22 | | |  |  |  |  |  |  |  | | --- | --- | --- | --- | --- | --- | --- | | |  |  | | --- | --- | | |  | | --- | |  | | |  |  |  |  | | | |
| |  |  |  |  |  |  |  |  |  |  |  |  | | --- | --- | --- | --- | --- | --- | --- | --- | --- | --- | --- | --- | | |  | | --- | | Cluster: 23 | | |  |  |  |  |  |  |  |  |  | | --- | --- | --- | --- | --- | --- | --- | --- | --- | | |  |  | | --- | --- | | |  | | --- | |  | | | |  |  | | --- | --- | | |  | | --- | |  | | |  |  |  | | | |
| |  |  |  |  |  |  |  |  |  |  | | --- | --- | --- | --- | --- | --- | --- | --- | --- | --- | | |  | | --- | | Cluster: 24 | | |  |  |  |  |  |  |  | | --- | --- | --- | --- | --- | --- | --- | | |  |  | | --- | --- | | |  | | --- | |  | | |  |  |  |  | | | |
| |  |  |  |  |  |  |  |  |  |  |  |  |  |  |  |  |  |  |  |  |  |  |  |  |  | | --- | --- | --- | --- | --- | --- | --- | --- | --- | --- | --- | --- | --- | --- | --- | --- | --- | --- | --- | --- | --- | --- | --- | --- | --- | | |  | | --- | | Cluster: 25 | | |  |  |  |  |  |  |  |  |  |  |  |  |  |  |  | | --- | --- | --- | --- | --- | --- | --- | --- | --- | --- | --- | --- | --- | --- | --- | | |  |  | | --- | --- | | |  | | --- | |  | | | |  |  | | --- | --- | | |  | | --- | |  | | | |  |  | | --- | --- | | |  | | --- | |  | | | |  |  | | --- | --- | | |  | | --- | |  | | | |  |  | | --- | --- | | |  | | --- | |  | | | | |  |  | | --- | --- | | |  | | --- | |  | | |  |  |  |  | | | |
| |  |  |  |  |  |  |  |  |  |  |  |  |  |  |  |  | | --- | --- | --- | --- | --- | --- | --- | --- | --- | --- | --- | --- | --- | --- | --- | --- | | |  | | --- | | Cluster: 26 | | |  |  |  |  |  |  |  |  |  |  |  |  |  | | --- | --- | --- | --- | --- | --- | --- | --- | --- | --- | --- | --- | --- | | |  |  | | --- | --- | | |  | | --- | |  | | | |  |  | | --- | --- | | |  | | --- | |  | | | |  |  | | --- | --- | | |  | | --- | |  | | | |  |  | | --- | --- | | |  | | --- | |  | | |  | | | |
| |  |  |  |  |  |  |  |  |  |  | | --- | --- | --- | --- | --- | --- | --- | --- | --- | --- | | |  | | --- | | Cluster: 27 | | |  |  |  |  |  |  |  | | --- | --- | --- | --- | --- | --- | --- | | |  |  | | --- | --- | | |  | | --- | |  | | |  |  |  |  | | | |
| |  |  |  |  |  |  |  |  |  |  |  |  |  |  | | --- | --- | --- | --- | --- | --- | --- | --- | --- | --- | --- | --- | --- | --- | | |  | | --- | | Cluster: 28 | | |  |  |  |  |  |  |  |  |  |  |  | | --- | --- | --- | --- | --- | --- | --- | --- | --- | --- | --- | | |  |  | | --- | --- | | |  | | --- | |  | | | |  |  | | --- | --- | | |  | | --- | |  | | | |  |  | | --- | --- | | |  | | --- | |  | | |  |  | | | |
| |  |  |  |  |  |  |  |  |  |  |  |  |  |  | | --- | --- | --- | --- | --- | --- | --- | --- | --- | --- | --- | --- | --- | --- | | |  | | --- | | Cluster: 29 | | |  |  |  |  |  |  |  |  |  |  |  | | --- | --- | --- | --- | --- | --- | --- | --- | --- | --- | --- | | |  |  | | --- | --- | | |  | | --- | |  | | | |  |  | | --- | --- | | |  | | --- | |  | | | |  |  | | --- | --- | | |  | | --- | |  | | |  |  | | | |
| |  |  |  |  |  |  |  |  |  |  |  |  | | --- | --- | --- | --- | --- | --- | --- | --- | --- | --- | --- | --- | | |  | | --- | | Cluster: 30 | | |  |  |  |  |  |  |  |  |  | | --- | --- | --- | --- | --- | --- | --- | --- | --- | | |  |  | | --- | --- | | |  | | --- | |  | | | |  |  | | --- | --- | | |  | | --- | |  | | |  |  |  | | | |
| |  |  |  |  |  |  |  |  |  |  | | --- | --- | --- | --- | --- | --- | --- | --- | --- | --- | | |  | | --- | | Cluster: 31 | | |  |  |  |  |  |  |  | | --- | --- | --- | --- | --- | --- | --- | | |  |  | | --- | --- | | |  | | --- | |  | | |  |  |  |  | | | |
| |  |  |  |  |  |  |  |  |  |  | | --- | --- | --- | --- | --- | --- | --- | --- | --- | --- | | |  | | --- | | Cluster: 32 | | |  |  |  |  |  |  |  | | --- | --- | --- | --- | --- | --- | --- | | |  |  | | --- | --- | | |  | | --- | |  | | |  |  |  |  | | | |
| |  |  |  |  |  |  |  |  |  |  | | --- | --- | --- | --- | --- | --- | --- | --- | --- | --- | | |  | | --- | | Cluster: 33 | | |  |  |  |  |  |  |  | | --- | --- | --- | --- | --- | --- | --- | | |  |  | | --- | --- | | |  | | --- | |  | | |  |  |  |  | | | |
| |  |  |  |  |  |  |  |  |  |  |  |  |  |  | | --- | --- | --- | --- | --- | --- | --- | --- | --- | --- | --- | --- | --- | --- | | |  | | --- | | Cluster: 34 | | |  |  |  |  |  |  |  |  |  |  |  | | --- | --- | --- | --- | --- | --- | --- | --- | --- | --- | --- | | |  |  | | --- | --- | | |  | | --- | |  | | | |  |  | | --- | --- | | |  | | --- | |  | | | |  |  | | --- | --- | | |  | | --- | |  | | |  |  | | | |
| |  |  |  |  |  |  |  |  |  |  | | --- | --- | --- | --- | --- | --- | --- | --- | --- | --- | | |  | | --- | | Cluster: 35 | | |  |  |  |  |  |  |  | | --- | --- | --- | --- | --- | --- | --- | | |  |  | | --- | --- | | |  | | --- | |  | | |  |  |  |  | | | |
| |  |  |  |  |  |  |  |  |  |  |  |  | | --- | --- | --- | --- | --- | --- | --- | --- | --- | --- | --- | --- | | |  | | --- | | Cluster: 36 | | |  |  |  |  |  |  |  |  |  | | --- | --- | --- | --- | --- | --- | --- | --- | --- | | |  |  | | --- | --- | | |  | | --- | |  | | | |  |  | | --- | --- | | |  | | --- | |  | | |  |  |  | | | |
| |  |  |  |  |  |  |  |  |  |  | | --- | --- | --- | --- | --- | --- | --- | --- | --- | --- | | |  | | --- | | Cluster: 37 | | |  |  |  |  |  |  |  | | --- | --- | --- | --- | --- | --- | --- | | |  |  | | --- | --- | | |  | | --- | |  | | |  |  |  |  | | | |
| |  |  |  |  |  |  |  |  |  |  |  |  | | --- | --- | --- | --- | --- | --- | --- | --- | --- | --- | --- | --- | | |  | | --- | | Cluster: 38 | | |  |  |  |  |  |  |  |  |  | | --- | --- | --- | --- | --- | --- | --- | --- | --- | | |  |  | | --- | --- | | |  | | --- | |  | | | |  |  | | --- | --- | | |  | | --- | |  | | |  |  |  | | | |
| |  |  |  |  |  |  |  |  |  |  |  |  | | --- | --- | --- | --- | --- | --- | --- | --- | --- | --- | --- | --- | | |  | | --- | | Cluster: 39 | | |  |  |  |  |  |  |  |  |  | | --- | --- | --- | --- | --- | --- | --- | --- | --- | | |  |  | | --- | --- | | |  | | --- | |  | | | |  |  | | --- | --- | | |  | | --- | |  | | |  |  |  | | | |
| |  |  |  |  |  |  |  |  |  |  | | --- | --- | --- | --- | --- | --- | --- | --- | --- | --- | | |  | | --- | | Cluster: 40 | | |  |  |  |  |  |  |  | | --- | --- | --- | --- | --- | --- | --- | | |  |  | | --- | --- | | |  | | --- | |  | | |  |  |  |  | | | |
| |  |  |  |  |  |  |  |  |  |  | | --- | --- | --- | --- | --- | --- | --- | --- | --- | --- | | |  | | --- | | Cluster: 41 | | |  |  |  |  |  |  |  | | --- | --- | --- | --- | --- | --- | --- | | |  |  | | --- | --- | | |  | | --- | |  | | |  |  |  |  | | | |
| |  |  |  |  |  |  |  |  |  |  |  |  | | --- | --- | --- | --- | --- | --- | --- | --- | --- | --- | --- | --- | | |  | | --- | | Cluster: 42 | | |  |  |  |  |  |  |  |  |  | | --- | --- | --- | --- | --- | --- | --- | --- | --- | | |  |  | | --- | --- | | |  | | --- | |  | | | |  |  | | --- | --- | | |  | | --- | |  | | |  |  |  | | | |
| |  |  |  |  |  |  |  |  |  |  |  |  |  |  |  |  |  |  |  |  |  |  |  |  |  |  |  |  |  |  |  |  |  |  |  |  |  |  |  |  |  |  |  |  |  |  |  |  |  |  |  |  |  |  |  |  |  |  |  | | --- | --- | --- | --- | --- | --- | --- | --- | --- | --- | --- | --- | --- | --- | --- | --- | --- | --- | --- | --- | --- | --- | --- | --- | --- | --- | --- | --- | --- | --- | --- | --- | --- | --- | --- | --- | --- | --- | --- | --- | --- | --- | --- | --- | --- | --- | --- | --- | --- | --- | --- | --- | --- | --- | --- | --- | --- | --- | --- | | |  | | --- | | Cluster: 43 | | |  |  |  |  |  |  |  |  |  |  |  |  |  |  |  | | --- | --- | --- | --- | --- | --- | --- | --- | --- | --- | --- | --- | --- | --- | --- | | |  |  | | --- | --- | | |  | | --- | |  | | | |  |  | | --- | --- | | |  | | --- | |  | | | |  |  | | --- | --- | | |  | | --- | |  | | | |  |  | | --- | --- | | |  | | --- | |  | | | |  |  | | --- | --- | | |  | | --- | |  | | | | |  |  | | --- | --- | | |  | | --- | |  | | | |  |  | | --- | --- | | |  | | --- | |  | | | |  |  | | --- | --- | | |  | | --- | |  | | | |  |  | | --- | --- | | |  | | --- | |  | | | |  |  | | --- | --- | | |  | | --- | |  | | | | |  |  | | --- | --- | | |  | | --- | |  | | | |  |  | | --- | --- | | |  | | --- | |  | | | |  |  | | --- | --- | | |  | | --- | |  | | | |  |  | | --- | --- | | |  | | --- | |  | | | |  |  | | --- | --- | | |  | | --- | |  | | | | |  |  | | --- | --- | | |  | | --- | |  | | | |  |  | | --- | --- | | |  | | --- | |  | | | |  |  | | --- | --- | | |  | | --- | |  | | |  |  | | | |
| |  |  |  |  |  |  |  |  |  |  | | --- | --- | --- | --- | --- | --- | --- | --- | --- | --- | | |  | | --- | | Cluster: 44 | | |  |  |  |  |  |  |  | | --- | --- | --- | --- | --- | --- | --- | | |  |  | | --- | --- | | |  | | --- | |  | | |  |  |  |  | | | |
| |  |  |  |  |  |  |  |  |  |  | | --- | --- | --- | --- | --- | --- | --- | --- | --- | --- | | |  | | --- | | Cluster: 45 | | |  |  |  |  |  |  |  | | --- | --- | --- | --- | --- | --- | --- | | |  |  | | --- | --- | | |  | | --- | |  | | |  |  |  |  | | | |
| |  |  |  |  |  |  |  |  |  |  | | --- | --- | --- | --- | --- | --- | --- | --- | --- | --- | | |  | | --- | | Cluster: 46 | | |  |  |  |  |  |  |  | | --- | --- | --- | --- | --- | --- | --- | | |  |  | | --- | --- | | |  | | --- | |  | | |  |  |  |  | | | |
| |  |  |  |  |  |  |  |  |  |  | | --- | --- | --- | --- | --- | --- | --- | --- | --- | --- | | |  | | --- | | Cluster: 47 | | |  |  |  |  |  |  |  | | --- | --- | --- | --- | --- | --- | --- | | |  |  | | --- | --- | | |  | | --- | |  | | |  |  |  |  | | | |
| |  |  |  |  |  |  |  |  |  |  | | --- | --- | --- | --- | --- | --- | --- | --- | --- | --- | | |  | | --- | | Cluster: 48 | | |  |  |  |  |  |  |  | | --- | --- | --- | --- | --- | --- | --- | | |  |  | | --- | --- | | |  | | --- | |  | | |  |  |  |  | | | |
| |  |  |  |  |  |  |  |  |  |  |  |  |  |  | | --- | --- | --- | --- | --- | --- | --- | --- | --- | --- | --- | --- | --- | --- | | |  | | --- | | Cluster: 49 | | |  |  |  |  |  |  |  |  |  |  |  | | --- | --- | --- | --- | --- | --- | --- | --- | --- | --- | --- | | |  |  | | --- | --- | | |  | | --- | |  | | | |  |  | | --- | --- | | |  | | --- | |  | | | |  |  | | --- | --- | | |  | | --- | |  | | |  |  | | | |
| |  |  |  |  |  |  |  |  |  |  | | --- | --- | --- | --- | --- | --- | --- | --- | --- | --- | | |  | | --- | | Cluster: 50 | | |  |  |  |  |  |  |  | | --- | --- | --- | --- | --- | --- | --- | | |  |  | | --- | --- | | |  | | --- | |  | | |  |  |  |  | | | |
| |  |  |  |  |  |  |  |  |  |  |  |  |  |  |  |  |  |  |  |  |  |  |  |  |  | | --- | --- | --- | --- | --- | --- | --- | --- | --- | --- | --- | --- | --- | --- | --- | --- | --- | --- | --- | --- | --- | --- | --- | --- | --- | | |  | | --- | | Cluster: 51 | | |  |  |  |  |  |  |  |  |  |  |  |  |  |  |  | | --- | --- | --- | --- | --- | --- | --- | --- | --- | --- | --- | --- | --- | --- | --- | | |  |  | | --- | --- | | |  | | --- | |  | | | |  |  | | --- | --- | | |  | | --- | |  | | | |  |  | | --- | --- | | |  | | --- | |  | | | |  |  | | --- | --- | | |  | | --- | |  | | | |  |  | | --- | --- | | |  | | --- | |  | | | | |  |  | | --- | --- | | |  | | --- | |  | | |  |  |  |  | | | |
| |  |  |  |  |  |  |  |  |  |  | | --- | --- | --- | --- | --- | --- | --- | --- | --- | --- | | |  | | --- | | Cluster: 52 | | |  |  |  |  |  |  |  | | --- | --- | --- | --- | --- | --- | --- | | |  |  | | --- | --- | | |  | | --- | |  | | |  |  |  |  | | | |
| |  |  |  |  |  |  |  |  |  |  | | --- | --- | --- | --- | --- | --- | --- | --- | --- | --- | | |  | | --- | | Cluster: 53 | | |  |  |  |  |  |  |  | | --- | --- | --- | --- | --- | --- | --- | | |  |  | | --- | --- | | |  | | --- | |  | | |  |  |  |  | | | |
| |  |  |  |  |  |  |  |  |  |  | | --- | --- | --- | --- | --- | --- | --- | --- | --- | --- | | |  | | --- | | Cluster: 54 | | |  |  |  |  |  |  |  | | --- | --- | --- | --- | --- | --- | --- | | |  |  | | --- | --- | | |  | | --- | |  | | |  |  |  |  | | | |
| |  |  |  |  |  |  |  |  |  |  | | --- | --- | --- | --- | --- | --- | --- | --- | --- | --- | | |  | | --- | | Cluster: 55 | | |  |  |  |  |  |  |  | | --- | --- | --- | --- | --- | --- | --- | | |  |  | | --- | --- | | |  | | --- | |  | | |  |  |  |  | | | |
| |  |  |  |  |  |  |  |  |  |  | | --- | --- | --- | --- | --- | --- | --- | --- | --- | --- | | |  | | --- | | Cluster: 56 | | |  |  |  |  |  |  |  | | --- | --- | --- | --- | --- | --- | --- | | |  |  | | --- | --- | | |  | | --- | |  | | |  |  |  |  | | | |
| |  |  |  |  |  |  |  |  |  |  |  |  | | --- | --- | --- | --- | --- | --- | --- | --- | --- | --- | --- | --- | | |  | | --- | | Cluster: 57 | | |  |  |  |  |  |  |  |  |  | | --- | --- | --- | --- | --- | --- | --- | --- | --- | | |  |  | | --- | --- | | |  | | --- | |  | | | |  |  | | --- | --- | | |  | | --- | |  | | |  |  |  | | | |
| |  |  |  |  |  |  |  |  |  |  | | --- | --- | --- | --- | --- | --- | --- | --- | --- | --- | | |  | | --- | | Cluster: 58 | | |  |  |  |  |  |  |  | | --- | --- | --- | --- | --- | --- | --- | | |  |  | | --- | --- | | |  | | --- | |  | | |  |  |  |  | | | |
| |  |  |  |  |  |  |  |  |  |  | | --- | --- | --- | --- | --- | --- | --- | --- | --- | --- | | |  | | --- | | Cluster: 59 | | |  |  |  |  |  |  |  | | --- | --- | --- | --- | --- | --- | --- | | |  |  | | --- | --- | | |  | | --- | |  | | |  |  |  |  | | | |
| |  |  |  |  |  |  |  |  |  |  |  |  |  |  |  |  |  |  |  |  |  |  |  |  |  | | --- | --- | --- | --- | --- | --- | --- | --- | --- | --- | --- | --- | --- | --- | --- | --- | --- | --- | --- | --- | --- | --- | --- | --- | --- | | |  | | --- | | Cluster: 60 | | |  |  |  |  |  |  |  |  |  |  |  |  |  |  |  | | --- | --- | --- | --- | --- | --- | --- | --- | --- | --- | --- | --- | --- | --- | --- | | |  |  | | --- | --- | | |  | | --- | |  | | | |  |  | | --- | --- | | |  | | --- | |  | | | |  |  | | --- | --- | | |  | | --- | |  | | | |  |  | | --- | --- | | |  | | --- | |  | | | |  |  | | --- | --- | | |  | | --- | |  | | | | |  |  | | --- | --- | | |  | | --- | |  | | |  |  |  |  | | | |
| |  |  |  |  |  |  |  |  |  |  | | --- | --- | --- | --- | --- | --- | --- | --- | --- | --- | | |  | | --- | | Cluster: 61 | | |  |  |  |  |  |  |  | | --- | --- | --- | --- | --- | --- | --- | | |  |  | | --- | --- | | |  | | --- | |  | | |  |  |  |  | | | |
| |  |  |  |  |  |  |  |  |  |  |  |  |  |  | | --- | --- | --- | --- | --- | --- | --- | --- | --- | --- | --- | --- | --- | --- | | |  | | --- | | Cluster: 62 | | |  |  |  |  |  |  |  |  |  |  |  | | --- | --- | --- | --- | --- | --- | --- | --- | --- | --- | --- | | |  |  | | --- | --- | | |  | | --- | |  | | | |  |  | | --- | --- | | |  | | --- | |  | | | |  |  | | --- | --- | | |  | | --- | |  | | |  |  | | | |
| |  |  |  |  |  |  |  |  |  |  |  |  |  |  |  |  |  |  |  |  |  |  |  |  |  |  |  |  |  | | --- | --- | --- | --- | --- | --- | --- | --- | --- | --- | --- | --- | --- | --- | --- | --- | --- | --- | --- | --- | --- | --- | --- | --- | --- | --- | --- | --- | --- | | |  | | --- | | Cluster: 63 | | |  |  |  |  |  |  |  |  |  |  |  |  |  |  |  | | --- | --- | --- | --- | --- | --- | --- | --- | --- | --- | --- | --- | --- | --- | --- | | |  |  | | --- | --- | | |  | | --- | |  | | | |  |  | | --- | --- | | |  | | --- | |  | | | |  |  | | --- | --- | | |  | | --- | |  | | | |  |  | | --- | --- | | |  | | --- | |  | | | |  |  | | --- | --- | | |  | | --- | |  | | | | |  |  | | --- | --- | | |  | | --- | |  | | | |  |  | | --- | --- | | |  | | --- | |  | | | |  |  | | --- | --- | | |  | | --- | |  | | |  |  | | | |
| |  |  |  |  |  |  |  |  |  |  |  |  | | --- | --- | --- | --- | --- | --- | --- | --- | --- | --- | --- | --- | | |  | | --- | | Cluster: 64 | | |  |  |  |  |  |  |  |  |  | | --- | --- | --- | --- | --- | --- | --- | --- | --- | | |  |  | | --- | --- | | |  | | --- | |  | | | |  |  | | --- | --- | | |  | | --- | |  | | |  |  |  | | | |
| |  |  |  |  |  |  |  |  |  |  | | --- | --- | --- | --- | --- | --- | --- | --- | --- | --- | | |  | | --- | | Cluster: 65 | | |  |  |  |  |  |  |  | | --- | --- | --- | --- | --- | --- | --- | | |  |  | | --- | --- | | |  | | --- | |  | | |  |  |  |  | | | |
| |  |  |  |  |  |  |  |  |  |  | | --- | --- | --- | --- | --- | --- | --- | --- | --- | --- | | |  | | --- | | Cluster: 66 | | |  |  |  |  |  |  |  | | --- | --- | --- | --- | --- | --- | --- | | |  |  | | --- | --- | | |  | | --- | |  | | |  |  |  |  | | | |
| |  |  |  |  |  |  |  |  |  |  | | --- | --- | --- | --- | --- | --- | --- | --- | --- | --- | | |  | | --- | | Cluster: 67 | | |  |  |  |  |  |  |  | | --- | --- | --- | --- | --- | --- | --- | | |  |  | | --- | --- | | |  | | --- | |  | | |  |  |  |  | | | |
| |  |  |  |  |  |  |  |  |  |  |  |  | | --- | --- | --- | --- | --- | --- | --- | --- | --- | --- | --- | --- | | |  | | --- | | Cluster: 68 | | |  |  |  |  |  |  |  |  |  | | --- | --- | --- | --- | --- | --- | --- | --- | --- | | |  |  | | --- | --- | | |  | | --- | |  | | | |  |  | | --- | --- | | |  | | --- | |  | | |  |  |  | | | |
| |  |  |  |  |  |  |  |  |  |  |  |  | | --- | --- | --- | --- | --- | --- | --- | --- | --- | --- | --- | --- | | |  | | --- | | Cluster: 69 | | |  |  |  |  |  |  |  |  |  | | --- | --- | --- | --- | --- | --- | --- | --- | --- | | |  |  | | --- | --- | | |  | | --- | |  | | | |  |  | | --- | --- | | |  | | --- | |  | | |  |  |  | | | |
| |  |  |  |  |  |  |  |  |  |  |  |  |  |  |  |  |  |  | | --- | --- | --- | --- | --- | --- | --- | --- | --- | --- | --- | --- | --- | --- | --- | --- | --- | --- | | |  | | --- | | Cluster: 70 | | |  |  |  |  |  |  |  |  |  |  |  |  |  |  |  | | --- | --- | --- | --- | --- | --- | --- | --- | --- | --- | --- | --- | --- | --- | --- | | |  |  | | --- | --- | | |  | | --- | |  | | | |  |  | | --- | --- | | |  | | --- | |  | | | |  |  | | --- | --- | | |  | | --- | |  | | | |  |  | | --- | --- | | |  | | --- | |  | | | |  |  | | --- | --- | | |  | | --- | |  | | | | | |
| |  |  |  |  |  |  |  |  |  |  |  |  | | --- | --- | --- | --- | --- | --- | --- | --- | --- | --- | --- | --- | | |  | | --- | | Cluster: 71 | | |  |  |  |  |  |  |  |  |  | | --- | --- | --- | --- | --- | --- | --- | --- | --- | | |  |  | | --- | --- | | |  | | --- | |  | | | |  |  | | --- | --- | | |  | | --- | |  | | |  |  |  | | | |
| |  |  |  |  |  |  |  |  |  |  |  |  | | --- | --- | --- | --- | --- | --- | --- | --- | --- | --- | --- | --- | | |  | | --- | | Cluster: 72 | | |  |  |  |  |  |  |  |  |  | | --- | --- | --- | --- | --- | --- | --- | --- | --- | | |  |  | | --- | --- | | |  | | --- | |  | | | |  |  | | --- | --- | | |  | | --- | |  | | |  |  |  | | | |
| |  |  |  |  |  |  |  |  |  |  | | --- | --- | --- | --- | --- | --- | --- | --- | --- | --- | | |  | | --- | | Cluster: 73 | | |  |  |  |  |  |  |  | | --- | --- | --- | --- | --- | --- | --- | | |  |  | | --- | --- | | |  | | --- | |  | | |  |  |  |  | | | |
| |  |  |  |  |  |  |  |  |  |  |  |  |  |  |  |  |  |  |  |  |  |  |  |  |  |  |  | | --- | --- | --- | --- | --- | --- | --- | --- | --- | --- | --- | --- | --- | --- | --- | --- | --- | --- | --- | --- | --- | --- | --- | --- | --- | --- | --- | | |  | | --- | | Cluster: 74 | | |  |  |  |  |  |  |  |  |  |  |  |  |  |  |  | | --- | --- | --- | --- | --- | --- | --- | --- | --- | --- | --- | --- | --- | --- | --- | | |  |  | | --- | --- | | |  | | --- | |  | | | |  |  | | --- | --- | | |  | | --- | |  | | | |  |  | | --- | --- | | |  | | --- | |  | | | |  |  | | --- | --- | | |  | | --- | |  | | | |  |  | | --- | --- | | |  | | --- | |  | | | | |  |  | | --- | --- | | |  | | --- | |  | | | |  |  | | --- | --- | | |  | | --- | |  | | |  |  |  | | | |
| |  |  |  |  |  |  |  |  |  |  |  |  | | --- | --- | --- | --- | --- | --- | --- | --- | --- | --- | --- | --- | | |  | | --- | | Cluster: 75 | | |  |  |  |  |  |  |  |  |  | | --- | --- | --- | --- | --- | --- | --- | --- | --- | | |  |  | | --- | --- | | |  | | --- | |  | | | |  |  | | --- | --- | | |  | | --- | |  | | |  |  |  | | | |
| |  |  |  |  |  |  |  |  |  |  |  |  | | --- | --- | --- | --- | --- | --- | --- | --- | --- | --- | --- | --- | | |  | | --- | | Cluster: 76 | | |  |  |  |  |  |  |  |  |  | | --- | --- | --- | --- | --- | --- | --- | --- | --- | | |  |  | | --- | --- | | |  | | --- | |  | | | |  |  | | --- | --- | | |  | | --- | |  | | |  |  |  | | | |
| |  |  |  |  |  |  |  |  |  |  | | --- | --- | --- | --- | --- | --- | --- | --- | --- | --- | | |  | | --- | | Cluster: 77 | | |  |  |  |  |  |  |  | | --- | --- | --- | --- | --- | --- | --- | | |  |  | | --- | --- | | |  | | --- | |  | | |  |  |  |  | | | |
| |  |  |  |  |  |  |  |  |  |  | | --- | --- | --- | --- | --- | --- | --- | --- | --- | --- | | |  | | --- | | Cluster: 78 | | |  |  |  |  |  |  |  | | --- | --- | --- | --- | --- | --- | --- | | |  |  | | --- | --- | | |  | | --- | |  | | |  |  |  |  | | | |
| |  |  |  |  |  |  |  |  |  |  |  |  | | --- | --- | --- | --- | --- | --- | --- | --- | --- | --- | --- | --- | | |  | | --- | | Cluster: 79 | | |  |  |  |  |  |  |  |  |  | | --- | --- | --- | --- | --- | --- | --- | --- | --- | | |  |  | | --- | --- | | |  | | --- | |  | | | |  |  | | --- | --- | | |  | | --- | |  | | |  |  |  | | | |
| |  |  |  |  |  |  |  |  |  |  | | --- | --- | --- | --- | --- | --- | --- | --- | --- | --- | | |  | | --- | | Cluster: 80 | | |  |  |  |  |  |  |  | | --- | --- | --- | --- | --- | --- | --- | | |  |  | | --- | --- | | |  | | --- | |  | | |  |  |  |  | | | |
| |  |  |  |  |  |  |  |  |  |  |  |  | | --- | --- | --- | --- | --- | --- | --- | --- | --- | --- | --- | --- | | |  | | --- | | Cluster: 81 | | |  |  |  |  |  |  |  |  |  | | --- | --- | --- | --- | --- | --- | --- | --- | --- | | |  |  | | --- | --- | | |  | | --- | |  | | | |  |  | | --- | --- | | |  | | --- | |  | | |  |  |  | | | |
| |  |  |  |  |  |  |  |  |  |  |  |  | | --- | --- | --- | --- | --- | --- | --- | --- | --- | --- | --- | --- | | |  | | --- | | Cluster: 82 | | |  |  |  |  |  |  |  |  |  | | --- | --- | --- | --- | --- | --- | --- | --- | --- | | |  |  | | --- | --- | | |  | | --- | |  | | | |  |  | | --- | --- | | |  | | --- | |  | | |  |  |  | | | |
| |  |  |  |  |  |  |  |  |  |  | | --- | --- | --- | --- | --- | --- | --- | --- | --- | --- | | |  | | --- | | Cluster: 83 | | |  |  |  |  |  |  |  | | --- | --- | --- | --- | --- | --- | --- | | |  |  | | --- | --- | | |  | | --- | |  | | |  |  |  |  | | | |
| |  |  |  |  |  |  |  |  |  |  |  |  | | --- | --- | --- | --- | --- | --- | --- | --- | --- | --- | --- | --- | | |  | | --- | | Cluster: 84 | | |  |  |  |  |  |  |  |  |  | | --- | --- | --- | --- | --- | --- | --- | --- | --- | | |  |  | | --- | --- | | |  | | --- | |  | | | |  |  | | --- | --- | | |  | | --- | |  | | |  |  |  | | | |
| |  |  |  |  |  |  |  |  |  |  |  |  | | --- | --- | --- | --- | --- | --- | --- | --- | --- | --- | --- | --- | | |  | | --- | | Cluster: 85 | | |  |  |  |  |  |  |  |  |  | | --- | --- | --- | --- | --- | --- | --- | --- | --- | | |  |  | | --- | --- | | |  | | --- | |  | | | |  |  | | --- | --- | | |  | | --- | |  | | |  |  |  | | | |
| |  |  |  |  |  |  |  |  |  |  |  |  | | --- | --- | --- | --- | --- | --- | --- | --- | --- | --- | --- | --- | | |  | | --- | | Cluster: 86 | | |  |  |  |  |  |  |  |  |  | | --- | --- | --- | --- | --- | --- | --- | --- | --- | | |  |  | | --- | --- | | |  | | --- | |  | | | |  |  | | --- | --- | | |  | | --- | |  | | |  |  |  | | | |
| |  |  |  |  |  |  |  |  |  |  | | --- | --- | --- | --- | --- | --- | --- | --- | --- | --- | | |  | | --- | | Cluster: 87 | | |  |  |  |  |  |  |  | | --- | --- | --- | --- | --- | --- | --- | | |  |  | | --- | --- | | |  | | --- | |  | | |  |  |  |  | | | |
| |  |  |  |  |  |  |  |  |  |  | | --- | --- | --- | --- | --- | --- | --- | --- | --- | --- | | |  | | --- | | Cluster: 88 | | |  |  |  |  |  |  |  | | --- | --- | --- | --- | --- | --- | --- | | |  |  | | --- | --- | | |  | | --- | |  | | |  |  |  |  | | | |
| |  |  |  |  |  |  |  |  |  |  | | --- | --- | --- | --- | --- | --- | --- | --- | --- | --- | | |  | | --- | | Cluster: 89 | | |  |  |  |  |  |  |  | | --- | --- | --- | --- | --- | --- | --- | | |  |  | | --- | --- | | |  | | --- | |  | | |  |  |  |  | | | |
| |  |  |  |  |  |  |  |  |  |  | | --- | --- | --- | --- | --- | --- | --- | --- | --- | --- | | |  | | --- | | Cluster: 90 | | |  |  |  |  |  |  |  | | --- | --- | --- | --- | --- | --- | --- | | |  |  | | --- | --- | | |  | | --- | |  | | |  |  |  |  | | | |
| |  |  |  |  |  |  |  |  |  |  |  |  |  |  |  |  | | --- | --- | --- | --- | --- | --- | --- | --- | --- | --- | --- | --- | --- | --- | --- | --- | | |  | | --- | | Cluster: 91 | | |  |  |  |  |  |  |  |  |  |  |  |  |  | | --- | --- | --- | --- | --- | --- | --- | --- | --- | --- | --- | --- | --- | | |  |  | | --- | --- | | |  | | --- | |  | | | |  |  | | --- | --- | | |  | | --- | |  | | | |  |  | | --- | --- | | |  | | --- | |  | | | |  |  | | --- | --- | | |  | | --- | |  | | |  | | | |
| |  |  |  |  |  |  |  |  |  |  |  |  | | --- | --- | --- | --- | --- | --- | --- | --- | --- | --- | --- | --- | | |  | | --- | | Cluster: 92 | | |  |  |  |  |  |  |  |  |  | | --- | --- | --- | --- | --- | --- | --- | --- | --- | | |  |  | | --- | --- | | |  | | --- | |  | | | |  |  | | --- | --- | | |  | | --- | |  | | |  |  |  | | | |
| |  |  |  |  |  |  |  |  |  |  | | --- | --- | --- | --- | --- | --- | --- | --- | --- | --- | | |  | | --- | | Cluster: 93 | | |  |  |  |  |  |  |  | | --- | --- | --- | --- | --- | --- | --- | | |  |  | | --- | --- | | |  | | --- | |  | | |  |  |  |  | | | |
| |  |  |  |  |  |  |  |  |  |  | | --- | --- | --- | --- | --- | --- | --- | --- | --- | --- | | |  | | --- | | Cluster: 94 | | |  |  |  |  |  |  |  | | --- | --- | --- | --- | --- | --- | --- | | |  |  | | --- | --- | | |  | | --- | |  | | |  |  |  |  | | | |
| |  |  |  |  |  |  |  |  |  |  | | --- | --- | --- | --- | --- | --- | --- | --- | --- | --- | | |  | | --- | | Cluster: 95 | | |  |  |  |  |  |  |  | | --- | --- | --- | --- | --- | --- | --- | | |  |  | | --- | --- | | |  | | --- | |  | | |  |  |  |  | | | |
| |  |  |  |  |  |  |  |  |  |  |  |  |  |  |  |  |  |  |  |  |  |  |  |  |  |  |  |  |  |  |  |  |  |  |  |  |  |  |  |  |  |  |  |  | | --- | --- | --- | --- | --- | --- | --- | --- | --- | --- | --- | --- | --- | --- | --- | --- | --- | --- | --- | --- | --- | --- | --- | --- | --- | --- | --- | --- | --- | --- | --- | --- | --- | --- | --- | --- | --- | --- | --- | --- | --- | --- | --- | --- | | |  | | --- | | Cluster: 96 | | |  |  |  |  |  |  |  |  |  |  |  |  |  |  |  | | --- | --- | --- | --- | --- | --- | --- | --- | --- | --- | --- | --- | --- | --- | --- | | |  |  | | --- | --- | | |  | | --- | |  | | | |  |  | | --- | --- | | |  | | --- | |  | | | |  |  | | --- | --- | | |  | | --- | |  | | | |  |  | | --- | --- | | |  | | --- | |  | | | |  |  | | --- | --- | | |  | | --- | |  | | | | |  |  | | --- | --- | | |  | | --- | |  | | | |  |  | | --- | --- | | |  | | --- | |  | | | |  |  | | --- | --- | | |  | | --- | |  | | | |  |  | | --- | --- | | |  | | --- | |  | | | |  |  | | --- | --- | | |  | | --- | |  | | | | |  |  | | --- | --- | | |  | | --- | |  | | | |  |  | | --- | --- | | |  | | --- | |  | | | |  |  | | --- | --- | | |  | | --- | |  | | |  |  | | | |
| |  |  |  |  |  |  |  |  |  |  |  |  | | --- | --- | --- | --- | --- | --- | --- | --- | --- | --- | --- | --- | | |  | | --- | | Cluster: 97 | | |  |  |  |  |  |  |  |  |  | | --- | --- | --- | --- | --- | --- | --- | --- | --- | | |  |  | | --- | --- | | |  | | --- | |  | | | |  |  | | --- | --- | | |  | | --- | |  | | |  |  |  | | | |
| |  |  |  |  |  |  |  |  |  |  | | --- | --- | --- | --- | --- | --- | --- | --- | --- | --- | | |  | | --- | | Cluster: 98 | | |  |  |  |  |  |  |  | | --- | --- | --- | --- | --- | --- | --- | | |  |  | | --- | --- | | |  | | --- | |  | | |  |  |  |  | | | |
| |  |  |  |  |  |  |  |  |  |  |  |  |  |  | | --- | --- | --- | --- | --- | --- | --- | --- | --- | --- | --- | --- | --- | --- | | |  | | --- | | Cluster: 99 | | |  |  |  |  |  |  |  |  |  |  |  | | --- | --- | --- | --- | --- | --- | --- | --- | --- | --- | --- | | |  |  | | --- | --- | | |  | | --- | |  | | | |  |  | | --- | --- | | |  | | --- | |  | | | |  |  | | --- | --- | | |  | | --- | |  | | |  |  | | | |
| |  |  |  |  |  |  |  |  |  |  |  |  |  |  |  |  |  |  |  |  |  |  |  |  |  |  |  |  |  |  |  | | --- | --- | --- | --- | --- | --- | --- | --- | --- | --- | --- | --- | --- | --- | --- | --- | --- | --- | --- | --- | --- | --- | --- | --- | --- | --- | --- | --- | --- | --- | --- | | |  | | --- | | Cluster: 100 | | |  |  |  |  |  |  |  |  |  |  |  |  |  |  |  | | --- | --- | --- | --- | --- | --- | --- | --- | --- | --- | --- | --- | --- | --- | --- | | |  |  | | --- | --- | | |  | | --- | |  | | | |  |  | | --- | --- | | |  | | --- | |  | | | |  |  | | --- | --- | | |  | | --- | |  | | | |  |  | | --- | --- | | |  | | --- | |  | | | |  |  | | --- | --- | | |  | | --- | |  | | | | |  |  | | --- | --- | | |  | | --- | |  | | | |  |  | | --- | --- | | |  | | --- | |  | | | |  |  | | --- | --- | | |  | | --- | |  | | | |  |  | | --- | --- | | |  | | --- | |  | | |  | | | |
| |  |  |  |  |  |  |  |  |  |  | | --- | --- | --- | --- | --- | --- | --- | --- | --- | --- | | |  | | --- | | Cluster: 101 | | |  |  |  |  |  |  |  | | --- | --- | --- | --- | --- | --- | --- | | |  |  | | --- | --- | | |  | | --- | |  | | |  |  |  |  | | | |
| |  |  |  |  |  |  |  |  |  |  | | --- | --- | --- | --- | --- | --- | --- | --- | --- | --- | | |  | | --- | | Cluster: 102 | | |  |  |  |  |  |  |  | | --- | --- | --- | --- | --- | --- | --- | | |  |  | | --- | --- | | |  | | --- | |  | | |  |  |  |  | | | |
| |  |  |  |  |  |  |  |  |  |  | | --- | --- | --- | --- | --- | --- | --- | --- | --- | --- | | |  | | --- | | Cluster: 103 | | |  |  |  |  |  |  |  | | --- | --- | --- | --- | --- | --- | --- | | |  |  | | --- | --- | | |  | | --- | |  | | |  |  |  |  | | | |
| |  |  |  |  |  |  |  |  |  |  | | --- | --- | --- | --- | --- | --- | --- | --- | --- | --- | | |  | | --- | | Cluster: 104 | | |  |  |  |  |  |  |  | | --- | --- | --- | --- | --- | --- | --- | | |  |  | | --- | --- | | |  | | --- | |  | | |  |  |  |  | | | |
| |  |  |  |  |  |  |  |  |  |  | | --- | --- | --- | --- | --- | --- | --- | --- | --- | --- | | |  | | --- | | Cluster: 105 | | |  |  |  |  |  |  |  | | --- | --- | --- | --- | --- | --- | --- | | |  |  | | --- | --- | | |  | | --- | |  | | |  |  |  |  | | | |
| |  |  |  |  |  |  |  |  |  |  |  |  | | --- | --- | --- | --- | --- | --- | --- | --- | --- | --- | --- | --- | | |  | | --- | | Cluster: 106 | | |  |  |  |  |  |  |  |  |  | | --- | --- | --- | --- | --- | --- | --- | --- | --- | | |  |  | | --- | --- | | |  | | --- | |  | | | |  |  | | --- | --- | | |  | | --- | |  | | |  |  |  | | | |
| |  |  |  |  |  |  |  |  |  |  |  |  | | --- | --- | --- | --- | --- | --- | --- | --- | --- | --- | --- | --- | | |  | | --- | | Cluster: 107 | | |  |  |  |  |  |  |  |  |  | | --- | --- | --- | --- | --- | --- | --- | --- | --- | | |  |  | | --- | --- | | |  | | --- | |  | | | |  |  | | --- | --- | | |  | | --- | |  | | |  |  |  | | | |
| |  |  |  |  |  |  |  |  |  |  | | --- | --- | --- | --- | --- | --- | --- | --- | --- | --- | | |  | | --- | | Cluster: 108 | | |  |  |  |  |  |  |  | | --- | --- | --- | --- | --- | --- | --- | | |  |  | | --- | --- | | |  | | --- | |  | | |  |  |  |  | | | |
| |  |  |  |  |  |  |  |  |  |  |  |  |  |  |  |  |  |  |  |  |  |  |  |  |  |  |  |  |  |  |  |  |  |  |  |  |  |  |  |  |  |  |  |  |  |  |  |  |  |  |  |  |  |  |  |  |  |  |  |  |  |  |  |  |  |  |  |  |  |  |  |  |  |  |  |  |  |  |  |  |  |  |  |  |  |  |  |  |  |  |  |  |  |  |  |  |  |  |  |  |  |  | | --- | --- | --- | --- | --- | --- | --- | --- | --- | --- | --- | --- | --- | --- | --- | --- | --- | --- | --- | --- | --- | --- | --- | --- | --- | --- | --- | --- | --- | --- | --- | --- | --- | --- | --- | --- | --- | --- | --- | --- | --- | --- | --- | --- | --- | --- | --- | --- | --- | --- | --- | --- | --- | --- | --- | --- | --- | --- | --- | --- | --- | --- | --- | --- | --- | --- | --- | --- | --- | --- | --- | --- | --- | --- | --- | --- | --- | --- | --- | --- | --- | --- | --- | --- | --- | --- | --- | --- | --- | --- | --- | --- | --- | --- | --- | --- | --- | --- | --- | --- | --- | --- | | |  | | --- | | Cluster: 109 | | |  |  |  |  |  |  |  |  |  |  |  |  |  |  |  | | --- | --- | --- | --- | --- | --- | --- | --- | --- | --- | --- | --- | --- | --- | --- | | |  |  | | --- | --- | | |  | | --- | |  | | | |  |  | | --- | --- | | |  | | --- | |  | | | |  |  | | --- | --- | | |  | | --- | |  | | | |  |  | | --- | --- | | |  | | --- | |  | | | |  |  | | --- | --- | | |  | | --- | |  | | | | |  |  | | --- | --- | | |  | | --- | |  | | | |  |  | | --- | --- | | |  | | --- | |  | | | |  |  | | --- | --- | | |  | | --- | |  | | | |  |  | | --- | --- | | |  | | --- | |  | | | |  |  | | --- | --- | | |  | | --- | |  | | | | |  |  | | --- | --- | | |  | | --- | |  | | | |  |  | | --- | --- | | |  | | --- | |  | | | |  |  | | --- | --- | | |  | | --- | |  | | | |  |  | | --- | --- | | |  | | --- | |  | | | |  |  | | --- | --- | | |  | | --- | |  | | | | |  |  | | --- | --- | | |  | | --- | |  | | | |  |  | | --- | --- | | |  | | --- | |  | | | |  |  | | --- | --- | | |  | | --- | |  | | | |  |  | | --- | --- | | |  | | --- | |  | | | |  |  | | --- | --- | | |  | | --- | |  | | | | |  |  | | --- | --- | | |  | | --- | |  | | | |  |  | | --- | --- | | |  | | --- | |  | | | |  |  | | --- | --- | | |  | | --- | |  | | | |  |  | | --- | --- | | |  | | --- | |  | | | |  |  | | --- | --- | | |  | | --- | |  | | | | |  |  | | --- | --- | | |  | | --- | |  | | | |  |  | | --- | --- | | |  | | --- | |  | | | |  |  | | --- | --- | | |  | | --- | |  | | | |  |  | | --- | --- | | |  | | --- | |  | | | |  |  | | --- | --- | | |  | | --- | |  | | | | |  |  | | --- | --- | | |  | | --- | |  | | | |  |  | | --- | --- | | |  | | --- | |  | | |  |  |  | | | |
| |  |  |  |  |  |  |  |  |  |  |  |  | | --- | --- | --- | --- | --- | --- | --- | --- | --- | --- | --- | --- | | |  | | --- | | Cluster: 110 | | |  |  |  |  |  |  |  |  |  | | --- | --- | --- | --- | --- | --- | --- | --- | --- | | |  |  | | --- | --- | | |  | | --- | |  | | | |  |  | | --- | --- | | |  | | --- | |  | | |  |  |  | | | |
| |  |  |  |  |  |  |  |  |  |  |  |  |  |  | | --- | --- | --- | --- | --- | --- | --- | --- | --- | --- | --- | --- | --- | --- | | |  | | --- | | Cluster: 111 | | |  |  |  |  |  |  |  |  |  |  |  | | --- | --- | --- | --- | --- | --- | --- | --- | --- | --- | --- | | |  |  | | --- | --- | | |  | | --- | |  | | | |  |  | | --- | --- | | |  | | --- | |  | | | |  |  | | --- | --- | | |  | | --- | |  | | |  |  | | | |
| |  |  |  |  |  |  |  |  |  |  |  |  |  |  | | --- | --- | --- | --- | --- | --- | --- | --- | --- | --- | --- | --- | --- | --- | | |  | | --- | | Cluster: 112 | | |  |  |  |  |  |  |  |  |  |  |  | | --- | --- | --- | --- | --- | --- | --- | --- | --- | --- | --- | | |  |  | | --- | --- | | |  | | --- | |  | | | |  |  | | --- | --- | | |  | | --- | |  | | | |  |  | | --- | --- | | |  | | --- | |  | | |  |  | | | |
| |  |  |  |  |  |  |  |  |  |  | | --- | --- | --- | --- | --- | --- | --- | --- | --- | --- | | |  | | --- | | Cluster: 113 | | |  |  |  |  |  |  |  | | --- | --- | --- | --- | --- | --- | --- | | |  |  | | --- | --- | | |  | | --- | |  | | |  |  |  |  | | | |
| |  |  |  |  |  |  |  |  |  |  |  |  |  |  |  |  |  |  |  |  |  |  |  |  |  |  |  | | --- | --- | --- | --- | --- | --- | --- | --- | --- | --- | --- | --- | --- | --- | --- | --- | --- | --- | --- | --- | --- | --- | --- | --- | --- | --- | --- | | |  | | --- | | Cluster: 114 | | |  |  |  |  |  |  |  |  |  |  |  |  |  |  |  | | --- | --- | --- | --- | --- | --- | --- | --- | --- | --- | --- | --- | --- | --- | --- | | |  |  | | --- | --- | | |  | | --- | |  | | | |  |  | | --- | --- | | |  | | --- | |  | | | |  |  | | --- | --- | | |  | | --- | |  | | | |  |  | | --- | --- | | |  | | --- | |  | | | |  |  | | --- | --- | | |  | | --- | |  | | | | |  |  | | --- | --- | | |  | | --- | |  | | | |  |  | | --- | --- | | |  | | --- | |  | | |  |  |  | | | |
| |  |  |  |  |  |  |  |  |  |  |  |  |  |  |  |  |  |  |  |  |  |  |  |  |  |  |  |  |  |  |  | | --- | --- | --- | --- | --- | --- | --- | --- | --- | --- | --- | --- | --- | --- | --- | --- | --- | --- | --- | --- | --- | --- | --- | --- | --- | --- | --- | --- | --- | --- | --- | | |  | | --- | | Cluster: 115 | | |  |  |  |  |  |  |  |  |  |  |  |  |  |  |  | | --- | --- | --- | --- | --- | --- | --- | --- | --- | --- | --- | --- | --- | --- | --- | | |  |  | | --- | --- | | |  | | --- | |  | | | |  |  | | --- | --- | | |  | | --- | |  | | | |  |  | | --- | --- | | |  | | --- | |  | | | |  |  | | --- | --- | | |  | | --- | |  | | | |  |  | | --- | --- | | |  | | --- | |  | | | | |  |  | | --- | --- | | |  | | --- | |  | | | |  |  | | --- | --- | | |  | | --- | |  | | | |  |  | | --- | --- | | |  | | --- | |  | | | |  |  | | --- | --- | | |  | | --- | |  | | |  | | | |
| |  |  |  |  |  |  |  |  |  |  | | --- | --- | --- | --- | --- | --- | --- | --- | --- | --- | | |  | | --- | | Cluster: 116 | | |  |  |  |  |  |  |  | | --- | --- | --- | --- | --- | --- | --- | | |  |  | | --- | --- | | |  | | --- | |  | | |  |  |  |  | | | |
| |  |  |  |  |  |  |  |  |  |  |  |  | | --- | --- | --- | --- | --- | --- | --- | --- | --- | --- | --- | --- | | |  | | --- | | Cluster: 117 | | |  |  |  |  |  |  |  |  |  | | --- | --- | --- | --- | --- | --- | --- | --- | --- | | |  |  | | --- | --- | | |  | | --- | |  | | | |  |  | | --- | --- | | |  | | --- | |  | | |  |  |  | | | |
| |  |  |  |  |  |  |  |  |  |  | | --- | --- | --- | --- | --- | --- | --- | --- | --- | --- | | |  | | --- | | Cluster: 118 | | |  |  |  |  |  |  |  | | --- | --- | --- | --- | --- | --- | --- | | |  |  | | --- | --- | | |  | | --- | |  | | |  |  |  |  | | | |
| |  |  |  |  |  |  |  |  |  |  |  |  | | --- | --- | --- | --- | --- | --- | --- | --- | --- | --- | --- | --- | | |  | | --- | | Cluster: 119 | | |  |  |  |  |  |  |  |  |  | | --- | --- | --- | --- | --- | --- | --- | --- | --- | | |  |  | | --- | --- | | |  | | --- | |  | | | |  |  | | --- | --- | | |  | | --- | |  | | |  |  |  | | | |
| |  |  |  |  |  |  |  |  |  |  | | --- | --- | --- | --- | --- | --- | --- | --- | --- | --- | | |  | | --- | | Cluster: 120 | | |  |  |  |  |  |  |  | | --- | --- | --- | --- | --- | --- | --- | | |  |  | | --- | --- | | |  | | --- | |  | | |  |  |  |  | | | |
| |  |  |  |  |  |  |  |  |  |  |  |  | | --- | --- | --- | --- | --- | --- | --- | --- | --- | --- | --- | --- | | |  | | --- | | Cluster: 121 | | |  |  |  |  |  |  |  |  |  | | --- | --- | --- | --- | --- | --- | --- | --- | --- | | |  |  | | --- | --- | | |  | | --- | |  | | | |  |  | | --- | --- | | |  | | --- | |  | | |  |  |  | | | |
| |  |  |  |  |  |  |  |  |  |  |  |  |  |  |  |  |  |  |  |  |  |  |  |  |  |  |  | | --- | --- | --- | --- | --- | --- | --- | --- | --- | --- | --- | --- | --- | --- | --- | --- | --- | --- | --- | --- | --- | --- | --- | --- | --- | --- | --- | | |  | | --- | | Cluster: 122 | | |  |  |  |  |  |  |  |  |  |  |  |  |  |  |  | | --- | --- | --- | --- | --- | --- | --- | --- | --- | --- | --- | --- | --- | --- | --- | | |  |  | | --- | --- | | |  | | --- | |  | | | |  |  | | --- | --- | | |  | | --- | |  | | | |  |  | | --- | --- | | |  | | --- | |  | | | |  |  | | --- | --- | | |  | | --- | |  | | | |  |  | | --- | --- | | |  | | --- | |  | | | | |  |  | | --- | --- | | |  | | --- | |  | | | |  |  | | --- | --- | | |  | | --- | |  | | |  |  |  | | | |
| |  |  |  |  |  |  |  |  |  |  | | --- | --- | --- | --- | --- | --- | --- | --- | --- | --- | | |  | | --- | | Cluster: 123 | | |  |  |  |  |  |  |  | | --- | --- | --- | --- | --- | --- | --- | | |  |  | | --- | --- | | |  | | --- | |  | | |  |  |  |  | | | |
| |  |  |  |  |  |  |  |  |  |  | | --- | --- | --- | --- | --- | --- | --- | --- | --- | --- | | |  | | --- | | Cluster: 124 | | |  |  |  |  |  |  |  | | --- | --- | --- | --- | --- | --- | --- | | |  |  | | --- | --- | | |  | | --- | |  | | |  |  |  |  | | | |
| |  |  |  |  |  |  |  |  |  |  | | --- | --- | --- | --- | --- | --- | --- | --- | --- | --- | | |  | | --- | | Cluster: 125 | | |  |  |  |  |  |  |  | | --- | --- | --- | --- | --- | --- | --- | | |  |  | | --- | --- | | |  | | --- | |  | | |  |  |  |  | | | |
| |  |  |  |  |  |  |  |  |  |  | | --- | --- | --- | --- | --- | --- | --- | --- | --- | --- | | |  | | --- | | Cluster: 126 | | |  |  |  |  |  |  |  | | --- | --- | --- | --- | --- | --- | --- | | |  |  | | --- | --- | | |  | | --- | |  | | |  |  |  |  | | | |
| |  |  |  |  |  |  |  |  |  |  | | --- | --- | --- | --- | --- | --- | --- | --- | --- | --- | | |  | | --- | | Cluster: 127 | | |  |  |  |  |  |  |  | | --- | --- | --- | --- | --- | --- | --- | | |  |  | | --- | --- | | |  | | --- | |  | | |  |  |  |  | | | |
| |  |  |  |  |  |  |  |  |  |  | | --- | --- | --- | --- | --- | --- | --- | --- | --- | --- | | |  | | --- | | Cluster: 128 | | |  |  |  |  |  |  |  | | --- | --- | --- | --- | --- | --- | --- | | |  |  | | --- | --- | | |  | | --- | |  | | |  |  |  |  | | | |
| |  |  |  |  |  |  |  |  |  |  | | --- | --- | --- | --- | --- | --- | --- | --- | --- | --- | | |  | | --- | | Cluster: 129 | | |  |  |  |  |  |  |  | | --- | --- | --- | --- | --- | --- | --- | | |  |  | | --- | --- | | |  | | --- | |  | | |  |  |  |  | | | |
| |  |  |  |  |  |  |  |  |  |  |  |  | | --- | --- | --- | --- | --- | --- | --- | --- | --- | --- | --- | --- | | |  | | --- | | Cluster: 130 | | |  |  |  |  |  |  |  |  |  | | --- | --- | --- | --- | --- | --- | --- | --- | --- | | |  |  | | --- | --- | | |  | | --- | |  | | | |  |  | | --- | --- | | |  | | --- | |  | | |  |  |  | | | |
| |  |  |  |  |  |  |  |  |  |  | | --- | --- | --- | --- | --- | --- | --- | --- | --- | --- | | |  | | --- | | Cluster: 131 | | |  |  |  |  |  |  |  | | --- | --- | --- | --- | --- | --- | --- | | |  |  | | --- | --- | | |  | | --- | |  | | |  |  |  |  | | | |
| |  |  |  |  |  |  |  |  |  |  | | --- | --- | --- | --- | --- | --- | --- | --- | --- | --- | | |  | | --- | | Cluster: 132 | | |  |  |  |  |  |  |  | | --- | --- | --- | --- | --- | --- | --- | | |  |  | | --- | --- | | |  | | --- | |  | | |  |  |  |  | | | |
| |  |  |  |  |  |  |  |  |  |  | | --- | --- | --- | --- | --- | --- | --- | --- | --- | --- | | |  | | --- | | Cluster: 133 | | |  |  |  |  |  |  |  | | --- | --- | --- | --- | --- | --- | --- | | |  |  | | --- | --- | | |  | | --- | |  | | |  |  |  |  | | | |
| |  |  |  |  |  |  |  |  |  |  |  |  |  |  | | --- | --- | --- | --- | --- | --- | --- | --- | --- | --- | --- | --- | --- | --- | | |  | | --- | | Cluster: 134 | | |  |  |  |  |  |  |  |  |  |  |  | | --- | --- | --- | --- | --- | --- | --- | --- | --- | --- | --- | | |  |  | | --- | --- | | |  | | --- | |  | | | |  |  | | --- | --- | | |  | | --- | |  | | | |  |  | | --- | --- | | |  | | --- | |  | | |  |  | | | |
| |  |  |  |  |  |  |  |  |  |  |  |  | | --- | --- | --- | --- | --- | --- | --- | --- | --- | --- | --- | --- | | |  | | --- | | Cluster: 135 | | |  |  |  |  |  |  |  |  |  | | --- | --- | --- | --- | --- | --- | --- | --- | --- | | |  |  | | --- | --- | | |  | | --- | |  | | | |  |  | | --- | --- | | |  | | --- | |  | | |  |  |  | | | |
