# Supplementary figures and images for: Predicting the points of interaction of small molecules in the NF-κB pathway (part 4 of 6)
Source: BMC Syst Biol. 2011 Feb 22;5:32. doi: 10.1186/1752-0509-5-32 (PMC3050742; doi:10.1186/1752-0509-5-32)

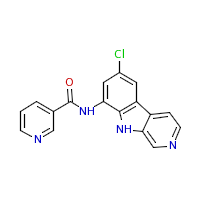

Supplement: Additional file 2 — Compounds clustered using ECFP_4 and Property Descriptors. [file 1752-0509-5-32-S2.ZIP › Additional Files 2/Additional Files 2_files/image37102.png]

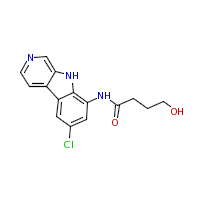

Supplement: Additional file 2 — Compounds clustered using ECFP_4 and Property Descriptors. [file 1752-0509-5-32-S2.ZIP › Additional Files 2/Additional Files 2_files/image37103.png]

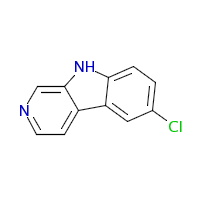

Supplement: Additional file 2 — Compounds clustered using ECFP_4 and Property Descriptors. [file 1752-0509-5-32-S2.ZIP › Additional Files 2/Additional Files 2_files/image37104.png]

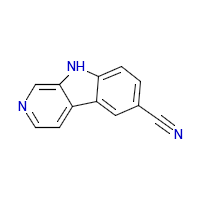

Supplement: Additional file 2 — Compounds clustered using ECFP_4 and Property Descriptors. [file 1752-0509-5-32-S2.ZIP › Additional Files 2/Additional Files 2_files/image37105.png]

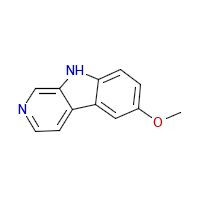

Supplement: Additional file 2 — Compounds clustered using ECFP_4 and Property Descriptors. [file 1752-0509-5-32-S2.ZIP › Additional Files 2/Additional Files 2_files/image37106.png]

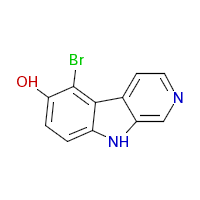

Supplement: Additional file 2 — Compounds clustered using ECFP_4 and Property Descriptors. [file 1752-0509-5-32-S2.ZIP › Additional Files 2/Additional Files 2_files/image37107.png]

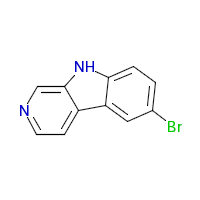

Supplement: Additional file 2 — Compounds clustered using ECFP_4 and Property Descriptors. [file 1752-0509-5-32-S2.ZIP › Additional Files 2/Additional Files 2_files/image37108.png]

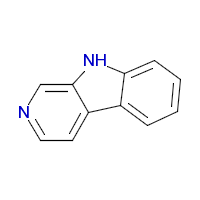

Supplement: Additional file 2 — Compounds clustered using ECFP_4 and Property Descriptors. [file 1752-0509-5-32-S2.ZIP › Additional Files 2/Additional Files 2_files/image37109.png]

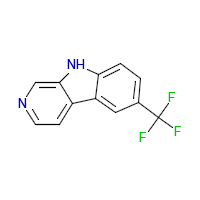

Supplement: Additional file 2 — Compounds clustered using ECFP_4 and Property Descriptors. [file 1752-0509-5-32-S2.ZIP › Additional Files 2/Additional Files 2_files/image37110.png]

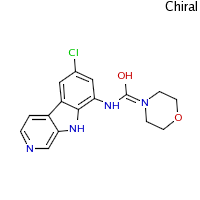

Supplement: Additional file 2 — Compounds clustered using ECFP_4 and Property Descriptors. [file 1752-0509-5-32-S2.ZIP › Additional Files 2/Additional Files 2_files/image37111.png]

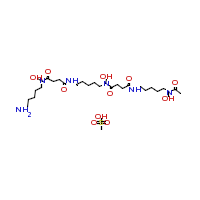

Supplement: Additional file 2 — Compounds clustered using ECFP_4 and Property Descriptors. [file 1752-0509-5-32-S2.ZIP › Additional Files 2/Additional Files 2_files/image37112.png]

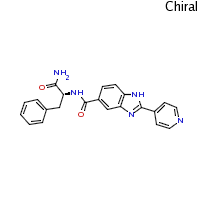

Supplement: Additional file 2 — Compounds clustered using ECFP_4 and Property Descriptors. [file 1752-0509-5-32-S2.ZIP › Additional Files 2/Additional Files 2_files/image37113.png]

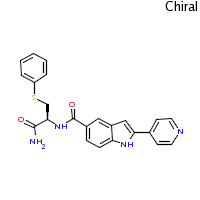

Supplement: Additional file 2 — Compounds clustered using ECFP_4 and Property Descriptors. [file 1752-0509-5-32-S2.ZIP › Additional Files 2/Additional Files 2_files/image37114.png]

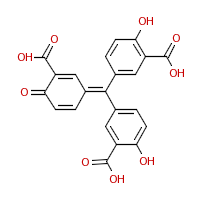

Supplement: Additional file 2 — Compounds clustered using ECFP_4 and Property Descriptors. [file 1752-0509-5-32-S2.ZIP › Additional Files 2/Additional Files 2_files/image37115.png]

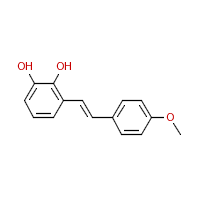

Supplement: Additional file 2 — Compounds clustered using ECFP_4 and Property Descriptors. [file 1752-0509-5-32-S2.ZIP › Additional Files 2/Additional Files 2_files/image37116.png]

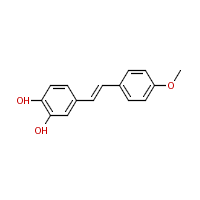

Supplement: Additional file 2 — Compounds clustered using ECFP_4 and Property Descriptors. [file 1752-0509-5-32-S2.ZIP › Additional Files 2/Additional Files 2_files/image37117.png]

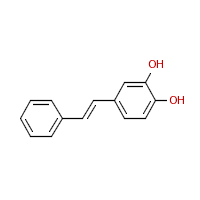

Supplement: Additional file 2 — Compounds clustered using ECFP_4 and Property Descriptors. [file 1752-0509-5-32-S2.ZIP › Additional Files 2/Additional Files 2_files/image37118.png]

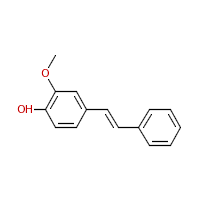

Supplement: Additional file 2 — Compounds clustered using ECFP_4 and Property Descriptors. [file 1752-0509-5-32-S2.ZIP › Additional Files 2/Additional Files 2_files/image37119.png]

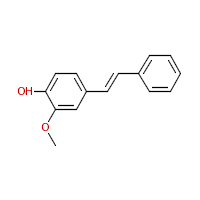

Supplement: Additional file 2 — Compounds clustered using ECFP_4 and Property Descriptors. [file 1752-0509-5-32-S2.ZIP › Additional Files 2/Additional Files 2_files/image37120.png]

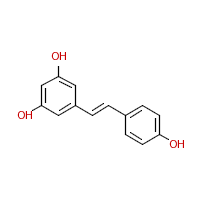

Supplement: Additional file 2 — Compounds clustered using ECFP_4 and Property Descriptors. [file 1752-0509-5-32-S2.ZIP › Additional Files 2/Additional Files 2_files/image37121.png]

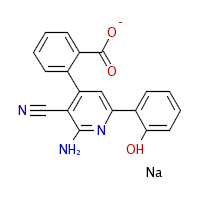

Supplement: Additional file 2 — Compounds clustered using ECFP_4 and Property Descriptors. [file 1752-0509-5-32-S2.ZIP › Additional Files 2/Additional Files 2_files/image37122.png]

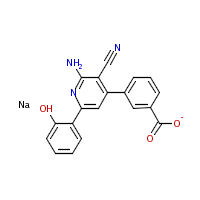

Supplement: Additional file 2 — Compounds clustered using ECFP_4 and Property Descriptors. [file 1752-0509-5-32-S2.ZIP › Additional Files 2/Additional Files 2_files/image37123.png]

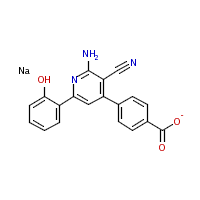

Supplement: Additional file 2 — Compounds clustered using ECFP_4 and Property Descriptors. [file 1752-0509-5-32-S2.ZIP › Additional Files 2/Additional Files 2_files/image37124.png]

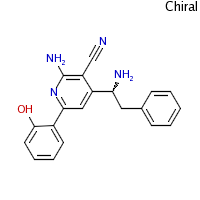

Supplement: Additional file 2 — Compounds clustered using ECFP_4 and Property Descriptors. [file 1752-0509-5-32-S2.ZIP › Additional Files 2/Additional Files 2_files/image37125.png]

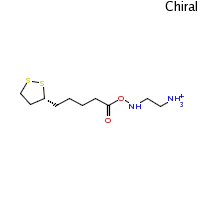

Supplement: Additional file 2 — Compounds clustered using ECFP_4 and Property Descriptors. [file 1752-0509-5-32-S2.ZIP › Additional Files 2/Additional Files 2_files/image37126.png]

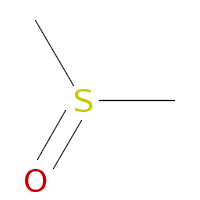

Supplement: Additional file 2 — Compounds clustered using ECFP_4 and Property Descriptors. [file 1752-0509-5-32-S2.ZIP › Additional Files 2/Additional Files 2_files/image37127.png]

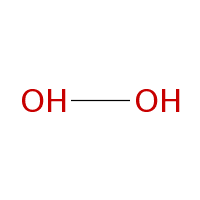

Supplement: Additional file 2 — Compounds clustered using ECFP_4 and Property Descriptors. [file 1752-0509-5-32-S2.ZIP › Additional Files 2/Additional Files 2_files/image37128.png]

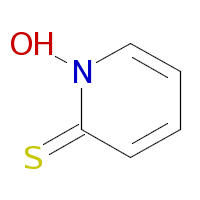

Supplement: Additional file 2 — Compounds clustered using ECFP_4 and Property Descriptors. [file 1752-0509-5-32-S2.ZIP › Additional Files 2/Additional Files 2_files/image37129.png]

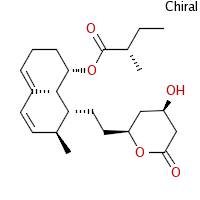

Supplement: Additional file 2 — Compounds clustered using ECFP_4 and Property Descriptors. [file 1752-0509-5-32-S2.ZIP › Additional Files 2/Additional Files 2_files/image37130.png]

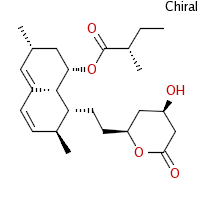

Supplement: Additional file 2 — Compounds clustered using ECFP_4 and Property Descriptors. [file 1752-0509-5-32-S2.ZIP › Additional Files 2/Additional Files 2_files/image37131.png]

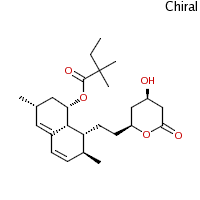

Supplement: Additional file 2 — Compounds clustered using ECFP_4 and Property Descriptors. [file 1752-0509-5-32-S2.ZIP › Additional Files 2/Additional Files 2_files/image37132.png]

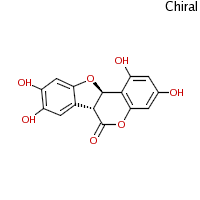

Supplement: Additional file 2 — Compounds clustered using ECFP_4 and Property Descriptors. [file 1752-0509-5-32-S2.ZIP › Additional Files 2/Additional Files 2_files/image37133.png]

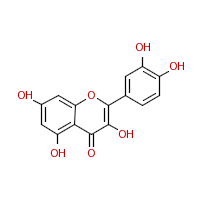

Supplement: Additional file 2 — Compounds clustered using ECFP_4 and Property Descriptors. [file 1752-0509-5-32-S2.ZIP › Additional Files 2/Additional Files 2_files/image37134.png]

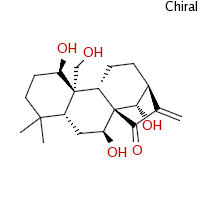

Supplement: Additional file 2 — Compounds clustered using ECFP_4 and Property Descriptors. [file 1752-0509-5-32-S2.ZIP › Additional Files 2/Additional Files 2_files/image37135.png]

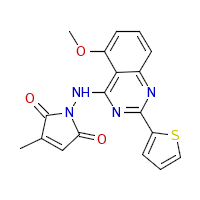

Supplement: Additional file 2 — Compounds clustered using ECFP_4 and Property Descriptors. [file 1752-0509-5-32-S2.ZIP › Additional Files 2/Additional Files 2_files/image37136.png]

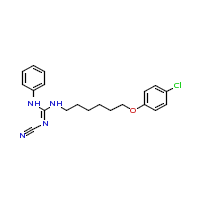

Supplement: Additional file 2 — Compounds clustered using ECFP_4 and Property Descriptors. [file 1752-0509-5-32-S2.ZIP › Additional Files 2/Additional Files 2_files/image37137.png]

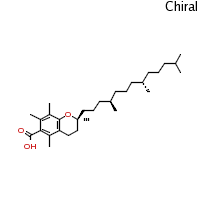

Supplement: Additional file 2 — Compounds clustered using ECFP_4 and Property Descriptors. [file 1752-0509-5-32-S2.ZIP › Additional Files 2/Additional Files 2_files/image37138.png]

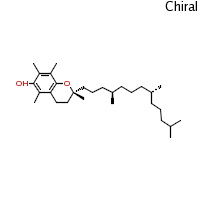

Supplement: Additional file 2 — Compounds clustered using ECFP_4 and Property Descriptors. [file 1752-0509-5-32-S2.ZIP › Additional Files 2/Additional Files 2_files/image37139.png]

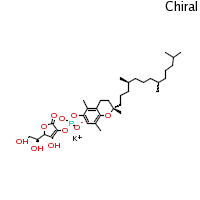

Supplement: Additional file 2 — Compounds clustered using ECFP_4 and Property Descriptors. [file 1752-0509-5-32-S2.ZIP › Additional Files 2/Additional Files 2_files/image37140.png]

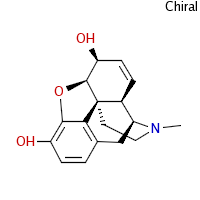

Supplement: Additional file 2 — Compounds clustered using ECFP_4 and Property Descriptors. [file 1752-0509-5-32-S2.ZIP › Additional Files 2/Additional Files 2_files/image37141.png]

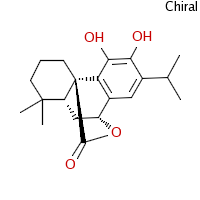

Supplement: Additional file 2 — Compounds clustered using ECFP_4 and Property Descriptors. [file 1752-0509-5-32-S2.ZIP › Additional Files 2/Additional Files 2_files/image37142.png]

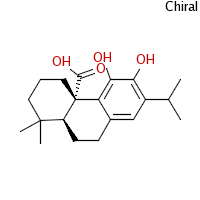

Supplement: Additional file 2 — Compounds clustered using ECFP_4 and Property Descriptors. [file 1752-0509-5-32-S2.ZIP › Additional Files 2/Additional Files 2_files/image37143.png]

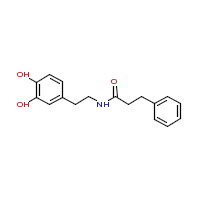

Supplement: Additional file 2 — Compounds clustered using ECFP_4 and Property Descriptors. [file 1752-0509-5-32-S2.ZIP › Additional Files 2/Additional Files 2_files/image37144.png]

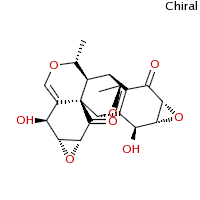

Supplement: Additional file 2 — Compounds clustered using ECFP_4 and Property Descriptors. [file 1752-0509-5-32-S2.ZIP › Additional Files 2/Additional Files 2_files/image37145.png]

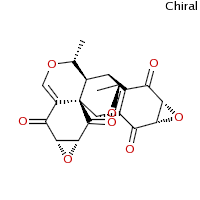

Supplement: Additional file 2 — Compounds clustered using ECFP_4 and Property Descriptors. [file 1752-0509-5-32-S2.ZIP › Additional Files 2/Additional Files 2_files/image37146.png]

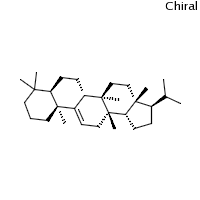

Supplement: Additional file 2 — Compounds clustered using ECFP_4 and Property Descriptors. [file 1752-0509-5-32-S2.ZIP › Additional Files 2/Additional Files 2_files/image37147.png]

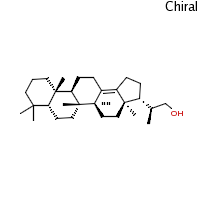

Supplement: Additional file 2 — Compounds clustered using ECFP_4 and Property Descriptors. [file 1752-0509-5-32-S2.ZIP › Additional Files 2/Additional Files 2_files/image37148.png]

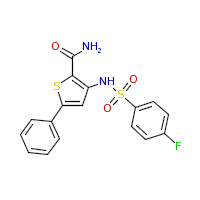

Supplement: Additional file 2 — Compounds clustered using ECFP_4 and Property Descriptors. [file 1752-0509-5-32-S2.ZIP › Additional Files 2/Additional Files 2_files/image37149.png]

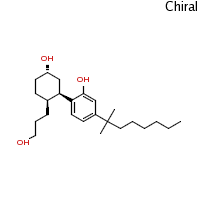

Supplement: Additional file 2 — Compounds clustered using ECFP_4 and Property Descriptors. [file 1752-0509-5-32-S2.ZIP › Additional Files 2/Additional Files 2_files/image37150.png]

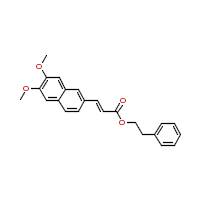

Supplement: Additional file 2 — Compounds clustered using ECFP_4 and Property Descriptors. [file 1752-0509-5-32-S2.ZIP › Additional Files 2/Additional Files 2_files/image37151.png]

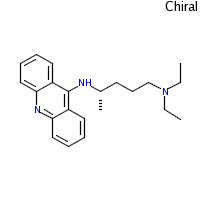

Supplement: Additional file 2 — Compounds clustered using ECFP_4 and Property Descriptors. [file 1752-0509-5-32-S2.ZIP › Additional Files 2/Additional Files 2_files/image37152.png]

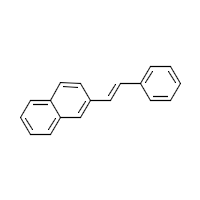

Supplement: Additional file 2 — Compounds clustered using ECFP_4 and Property Descriptors. [file 1752-0509-5-32-S2.ZIP › Additional Files 2/Additional Files 2_files/image37153.png]

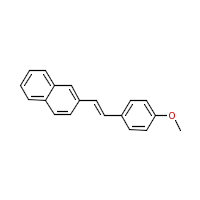

Supplement: Additional file 2 — Compounds clustered using ECFP_4 and Property Descriptors. [file 1752-0509-5-32-S2.ZIP › Additional Files 2/Additional Files 2_files/image37154.png]

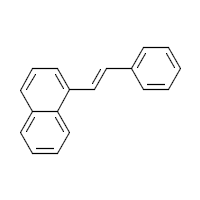

Supplement: Additional file 2 — Compounds clustered using ECFP_4 and Property Descriptors. [file 1752-0509-5-32-S2.ZIP › Additional Files 2/Additional Files 2_files/image37155.png]

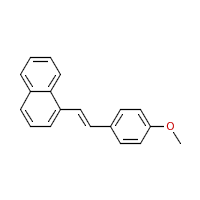

Supplement: Additional file 2 — Compounds clustered using ECFP_4 and Property Descriptors. [file 1752-0509-5-32-S2.ZIP › Additional Files 2/Additional Files 2_files/image37156.png]

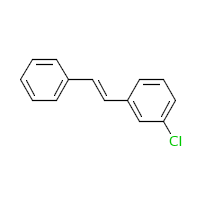

Supplement: Additional file 2 — Compounds clustered using ECFP_4 and Property Descriptors. [file 1752-0509-5-32-S2.ZIP › Additional Files 2/Additional Files 2_files/image37157.png]

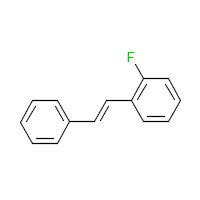

Supplement: Additional file 2 — Compounds clustered using ECFP_4 and Property Descriptors. [file 1752-0509-5-32-S2.ZIP › Additional Files 2/Additional Files 2_files/image37158.png]

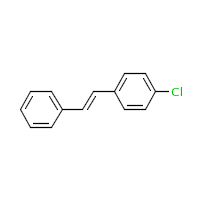

Supplement: Additional file 2 — Compounds clustered using ECFP_4 and Property Descriptors. [file 1752-0509-5-32-S2.ZIP › Additional Files 2/Additional Files 2_files/image37159.png]

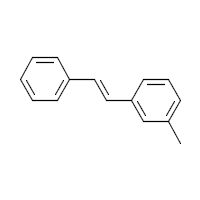

Supplement: Additional file 2 — Compounds clustered using ECFP_4 and Property Descriptors. [file 1752-0509-5-32-S2.ZIP › Additional Files 2/Additional Files 2_files/image37160.png]

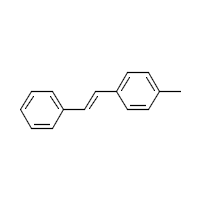

Supplement: Additional file 2 — Compounds clustered using ECFP_4 and Property Descriptors. [file 1752-0509-5-32-S2.ZIP › Additional Files 2/Additional Files 2_files/image37161.png]

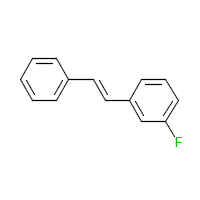

Supplement: Additional file 2 — Compounds clustered using ECFP_4 and Property Descriptors. [file 1752-0509-5-32-S2.ZIP › Additional Files 2/Additional Files 2_files/image37162.png]

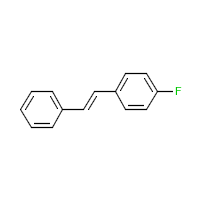

Supplement: Additional file 2 — Compounds clustered using ECFP_4 and Property Descriptors. [file 1752-0509-5-32-S2.ZIP › Additional Files 2/Additional Files 2_files/image37163.png]

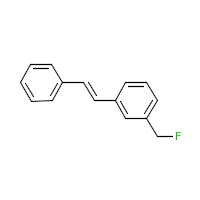

Supplement: Additional file 2 — Compounds clustered using ECFP_4 and Property Descriptors. [file 1752-0509-5-32-S2.ZIP › Additional Files 2/Additional Files 2_files/image37164.png]

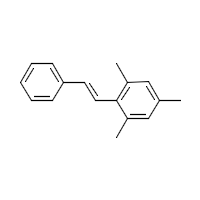

Supplement: Additional file 2 — Compounds clustered using ECFP_4 and Property Descriptors. [file 1752-0509-5-32-S2.ZIP › Additional Files 2/Additional Files 2_files/image37165.png]

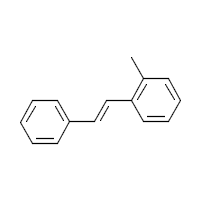

Supplement: Additional file 2 — Compounds clustered using ECFP_4 and Property Descriptors. [file 1752-0509-5-32-S2.ZIP › Additional Files 2/Additional Files 2_files/image37166.png]

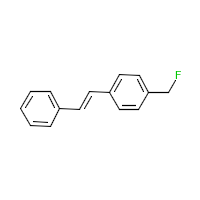

Supplement: Additional file 2 — Compounds clustered using ECFP_4 and Property Descriptors. [file 1752-0509-5-32-S2.ZIP › Additional Files 2/Additional Files 2_files/image37167.png]

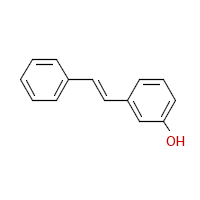

Supplement: Additional file 2 — Compounds clustered using ECFP_4 and Property Descriptors. [file 1752-0509-5-32-S2.ZIP › Additional Files 2/Additional Files 2_files/image37168.png]

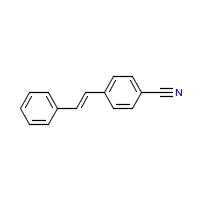

Supplement: Additional file 2 — Compounds clustered using ECFP_4 and Property Descriptors. [file 1752-0509-5-32-S2.ZIP › Additional Files 2/Additional Files 2_files/image37169.png]

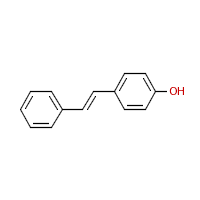

Supplement: Additional file 2 — Compounds clustered using ECFP_4 and Property Descriptors. [file 1752-0509-5-32-S2.ZIP › Additional Files 2/Additional Files 2_files/image37170.png]

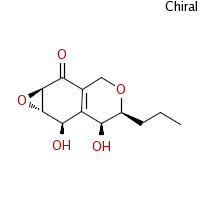

Supplement: Additional file 2 — Compounds clustered using ECFP_4 and Property Descriptors. [file 1752-0509-5-32-S2.ZIP › Additional Files 2/Additional Files 2_files/image37171.png]

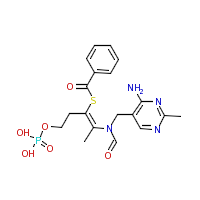

Supplement: Additional file 2 — Compounds clustered using ECFP_4 and Property Descriptors. [file 1752-0509-5-32-S2.ZIP › Additional Files 2/Additional Files 2_files/image37172.png]

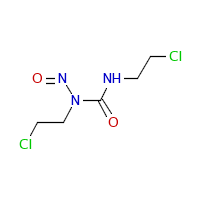

Supplement: Additional file 2 — Compounds clustered using ECFP_4 and Property Descriptors. [file 1752-0509-5-32-S2.ZIP › Additional Files 2/Additional Files 2_files/image37173.png]

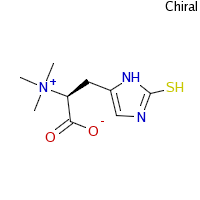

Supplement: Additional file 2 — Compounds clustered using ECFP_4 and Property Descriptors. [file 1752-0509-5-32-S2.ZIP › Additional Files 2/Additional Files 2_files/image37174.png]

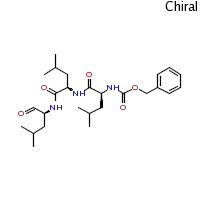

Supplement: Additional file 2 — Compounds clustered using ECFP_4 and Property Descriptors. [file 1752-0509-5-32-S2.ZIP › Additional Files 2/Additional Files 2_files/image37175.png]

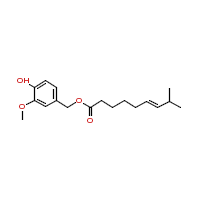

Supplement: Additional file 2 — Compounds clustered using ECFP_4 and Property Descriptors. [file 1752-0509-5-32-S2.ZIP › Additional Files 2/Additional Files 2_files/image37176.png]

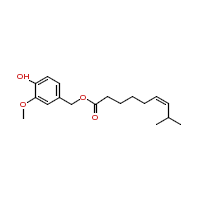

Supplement: Additional file 2 — Compounds clustered using ECFP_4 and Property Descriptors. [file 1752-0509-5-32-S2.ZIP › Additional Files 2/Additional Files 2_files/image37177.png]

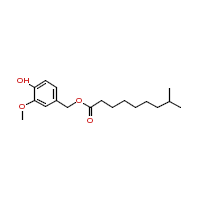

Supplement: Additional file 2 — Compounds clustered using ECFP_4 and Property Descriptors. [file 1752-0509-5-32-S2.ZIP › Additional Files 2/Additional Files 2_files/image37178.png]

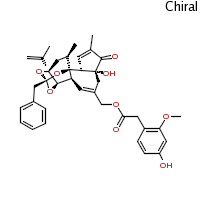

Supplement: Additional file 2 — Compounds clustered using ECFP_4 and Property Descriptors. [file 1752-0509-5-32-S2.ZIP › Additional Files 2/Additional Files 2_files/image37179.png]

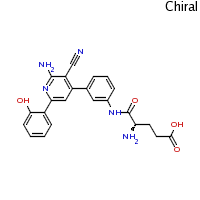

Supplement: Additional file 2 — Compounds clustered using ECFP_4 and Property Descriptors. [file 1752-0509-5-32-S2.ZIP › Additional Files 2/Additional Files 2_files/image37180.png]

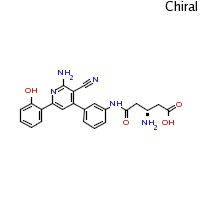

Supplement: Additional file 2 — Compounds clustered using ECFP_4 and Property Descriptors. [file 1752-0509-5-32-S2.ZIP › Additional Files 2/Additional Files 2_files/image37181.png]

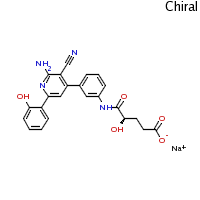

Supplement: Additional file 2 — Compounds clustered using ECFP_4 and Property Descriptors. [file 1752-0509-5-32-S2.ZIP › Additional Files 2/Additional Files 2_files/image37182.png]

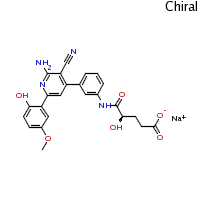

Supplement: Additional file 2 — Compounds clustered using ECFP_4 and Property Descriptors. [file 1752-0509-5-32-S2.ZIP › Additional Files 2/Additional Files 2_files/image37183.png]

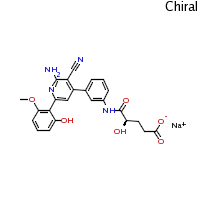

Supplement: Additional file 2 — Compounds clustered using ECFP_4 and Property Descriptors. [file 1752-0509-5-32-S2.ZIP › Additional Files 2/Additional Files 2_files/image37184.png]

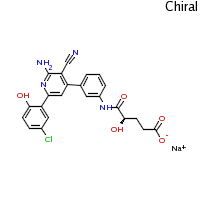

Supplement: Additional file 2 — Compounds clustered using ECFP_4 and Property Descriptors. [file 1752-0509-5-32-S2.ZIP › Additional Files 2/Additional Files 2_files/image37185.png]

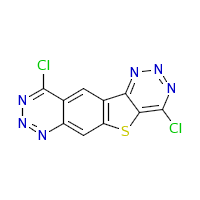

Supplement: Additional file 2 — Compounds clustered using ECFP_4 and Property Descriptors. [file 1752-0509-5-32-S2.ZIP › Additional Files 2/Additional Files 2_files/image37186.png]

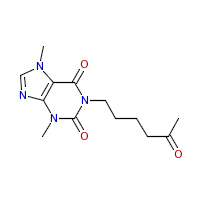

Supplement: Additional file 2 — Compounds clustered using ECFP_4 and Property Descriptors. [file 1752-0509-5-32-S2.ZIP › Additional Files 2/Additional Files 2_files/image37187.png]

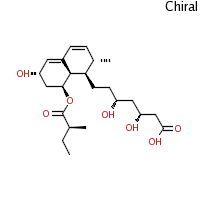

Supplement: Additional file 2 — Compounds clustered using ECFP_4 and Property Descriptors. [file 1752-0509-5-32-S2.ZIP › Additional Files 2/Additional Files 2_files/image37188.png]

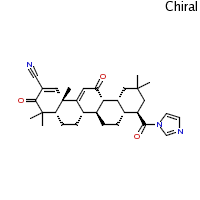

Supplement: Additional file 2 — Compounds clustered using ECFP_4 and Property Descriptors. [file 1752-0509-5-32-S2.ZIP › Additional Files 2/Additional Files 2_files/image37189.png]

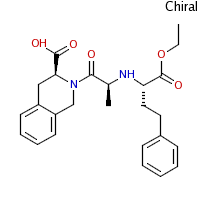

Supplement: Additional file 2 — Compounds clustered using ECFP_4 and Property Descriptors. [file 1752-0509-5-32-S2.ZIP › Additional Files 2/Additional Files 2_files/image37190.png]

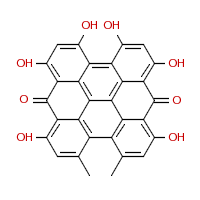

Supplement: Additional file 2 — Compounds clustered using ECFP_4 and Property Descriptors. [file 1752-0509-5-32-S2.ZIP › Additional Files 2/Additional Files 2_files/image37191.png]

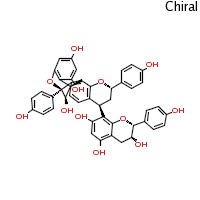

Supplement: Additional file 2 — Compounds clustered using ECFP_4 and Property Descriptors. [file 1752-0509-5-32-S2.ZIP › Additional Files 2/Additional Files 2_files/image37192.png]

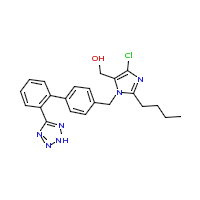

Supplement: Additional file 2 — Compounds clustered using ECFP_4 and Property Descriptors. [file 1752-0509-5-32-S2.ZIP › Additional Files 2/Additional Files 2_files/image37193.png]

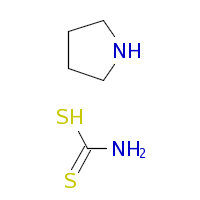

Supplement: Additional file 2 — Compounds clustered using ECFP_4 and Property Descriptors. [file 1752-0509-5-32-S2.ZIP › Additional Files 2/Additional Files 2_files/image37194.png]

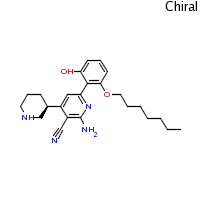

Supplement: Additional file 2 — Compounds clustered using ECFP_4 and Property Descriptors. [file 1752-0509-5-32-S2.ZIP › Additional Files 2/Additional Files 2_files/image37195.png]

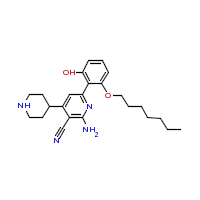

Supplement: Additional file 2 — Compounds clustered using ECFP_4 and Property Descriptors. [file 1752-0509-5-32-S2.ZIP › Additional Files 2/Additional Files 2_files/image37196.png]

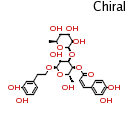

Supplement: Additional file 3 — Clusters of Compounds Shown in Figure 6. [file 1752-0509-5-32-S3.ZIP › Additional Files 3/Clustering_excluding_compounds_with_unknown_interactions_files/image5293.png]

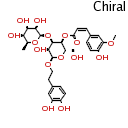

Supplement: Additional file 3 — Clusters of Compounds Shown in Figure 6. [file 1752-0509-5-32-S3.ZIP › Additional Files 3/Clustering_excluding_compounds_with_unknown_interactions_files/image5294.png]

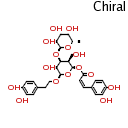

Supplement: Additional file 3 — Clusters of Compounds Shown in Figure 6. [file 1752-0509-5-32-S3.ZIP › Additional Files 3/Clustering_excluding_compounds_with_unknown_interactions_files/image5295.png]

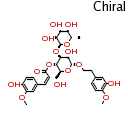

Supplement: Additional file 3 — Clusters of Compounds Shown in Figure 6. [file 1752-0509-5-32-S3.ZIP › Additional Files 3/Clustering_excluding_compounds_with_unknown_interactions_files/image5296.png]

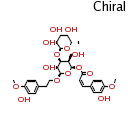

Supplement: Additional file 3 — Clusters of Compounds Shown in Figure 6. [file 1752-0509-5-32-S3.ZIP › Additional Files 3/Clustering_excluding_compounds_with_unknown_interactions_files/image5297.png]
