# Supplementary figures and images for: Predicting the points of interaction of small molecules in the NF-κB pathway (part 3 of 6)
Source: BMC Syst Biol. 2011 Feb 22;5:32. doi: 10.1186/1752-0509-5-32 (PMC3050742; doi:10.1186/1752-0509-5-32)

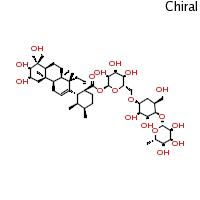

Supplement: Additional file 2 — Compounds clustered using ECFP_4 and Property Descriptors. [file 1752-0509-5-32-S2.ZIP › Additional Files 2/Additional Files 2_files/image37000.png]

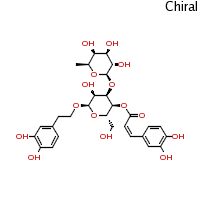

Supplement: Additional file 2 — Compounds clustered using ECFP_4 and Property Descriptors. [file 1752-0509-5-32-S2.ZIP › Additional Files 2/Additional Files 2_files/image37001.png]

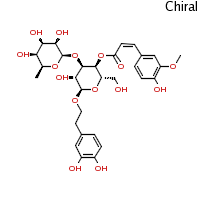

Supplement: Additional file 2 — Compounds clustered using ECFP_4 and Property Descriptors. [file 1752-0509-5-32-S2.ZIP › Additional Files 2/Additional Files 2_files/image37002.png]

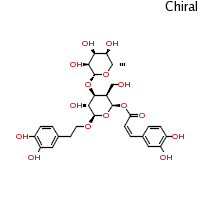

Supplement: Additional file 2 — Compounds clustered using ECFP_4 and Property Descriptors. [file 1752-0509-5-32-S2.ZIP › Additional Files 2/Additional Files 2_files/image37003.png]

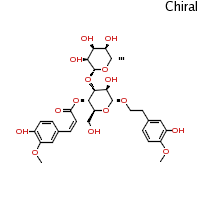

Supplement: Additional file 2 — Compounds clustered using ECFP_4 and Property Descriptors. [file 1752-0509-5-32-S2.ZIP › Additional Files 2/Additional Files 2_files/image37004.png]

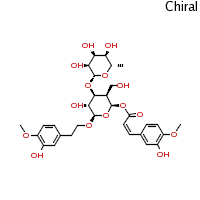

Supplement: Additional file 2 — Compounds clustered using ECFP_4 and Property Descriptors. [file 1752-0509-5-32-S2.ZIP › Additional Files 2/Additional Files 2_files/image37005.png]

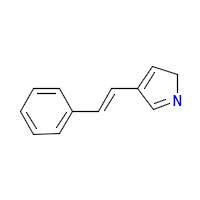

Supplement: Additional file 2 — Compounds clustered using ECFP_4 and Property Descriptors. [file 1752-0509-5-32-S2.ZIP › Additional Files 2/Additional Files 2_files/image37006.png]

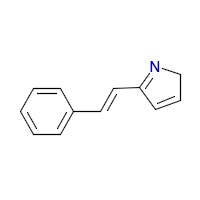

Supplement: Additional file 2 — Compounds clustered using ECFP_4 and Property Descriptors. [file 1752-0509-5-32-S2.ZIP › Additional Files 2/Additional Files 2_files/image37007.png]

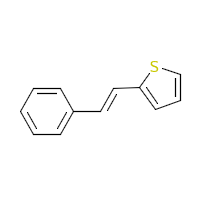

Supplement: Additional file 2 — Compounds clustered using ECFP_4 and Property Descriptors. [file 1752-0509-5-32-S2.ZIP › Additional Files 2/Additional Files 2_files/image37008.png]

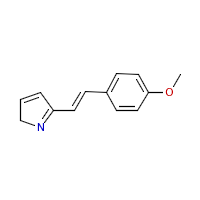

Supplement: Additional file 2 — Compounds clustered using ECFP_4 and Property Descriptors. [file 1752-0509-5-32-S2.ZIP › Additional Files 2/Additional Files 2_files/image37009.png]

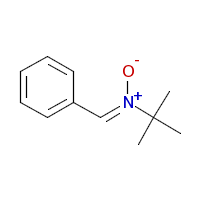

Supplement: Additional file 2 — Compounds clustered using ECFP_4 and Property Descriptors. [file 1752-0509-5-32-S2.ZIP › Additional Files 2/Additional Files 2_files/image37010.png]

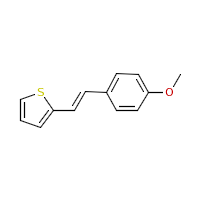

Supplement: Additional file 2 — Compounds clustered using ECFP_4 and Property Descriptors. [file 1752-0509-5-32-S2.ZIP › Additional Files 2/Additional Files 2_files/image37011.png]

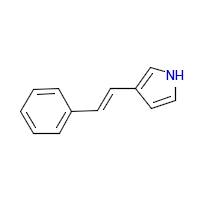

Supplement: Additional file 2 — Compounds clustered using ECFP_4 and Property Descriptors. [file 1752-0509-5-32-S2.ZIP › Additional Files 2/Additional Files 2_files/image37012.png]

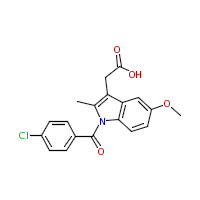

Supplement: Additional file 2 — Compounds clustered using ECFP_4 and Property Descriptors. [file 1752-0509-5-32-S2.ZIP › Additional Files 2/Additional Files 2_files/image37013.png]

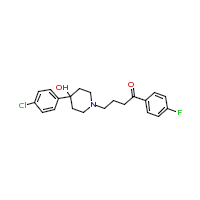

Supplement: Additional file 2 — Compounds clustered using ECFP_4 and Property Descriptors. [file 1752-0509-5-32-S2.ZIP › Additional Files 2/Additional Files 2_files/image37014.png]

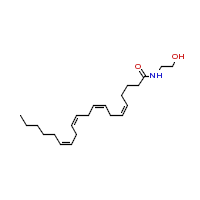

Supplement: Additional file 2 — Compounds clustered using ECFP_4 and Property Descriptors. [file 1752-0509-5-32-S2.ZIP › Additional Files 2/Additional Files 2_files/image37015.png]

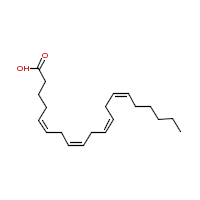

Supplement: Additional file 2 — Compounds clustered using ECFP_4 and Property Descriptors. [file 1752-0509-5-32-S2.ZIP › Additional Files 2/Additional Files 2_files/image37016.png]

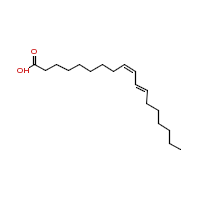

Supplement: Additional file 2 — Compounds clustered using ECFP_4 and Property Descriptors. [file 1752-0509-5-32-S2.ZIP › Additional Files 2/Additional Files 2_files/image37017.png]

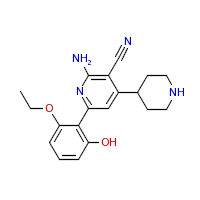

Supplement: Additional file 2 — Compounds clustered using ECFP_4 and Property Descriptors. [file 1752-0509-5-32-S2.ZIP › Additional Files 2/Additional Files 2_files/image37018.png]

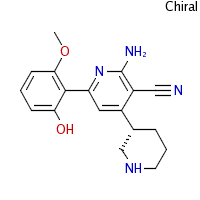

Supplement: Additional file 2 — Compounds clustered using ECFP_4 and Property Descriptors. [file 1752-0509-5-32-S2.ZIP › Additional Files 2/Additional Files 2_files/image37019.png]

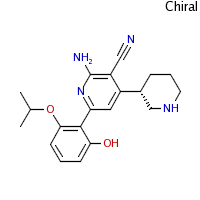

Supplement: Additional file 2 — Compounds clustered using ECFP_4 and Property Descriptors. [file 1752-0509-5-32-S2.ZIP › Additional Files 2/Additional Files 2_files/image37020.png]

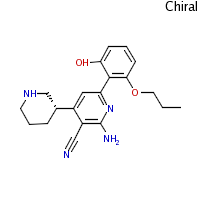

Supplement: Additional file 2 — Compounds clustered using ECFP_4 and Property Descriptors. [file 1752-0509-5-32-S2.ZIP › Additional Files 2/Additional Files 2_files/image37021.png]

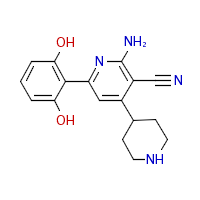

Supplement: Additional file 2 — Compounds clustered using ECFP_4 and Property Descriptors. [file 1752-0509-5-32-S2.ZIP › Additional Files 2/Additional Files 2_files/image37022.png]

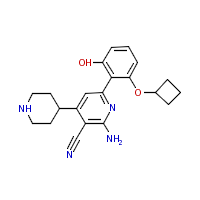

Supplement: Additional file 2 — Compounds clustered using ECFP_4 and Property Descriptors. [file 1752-0509-5-32-S2.ZIP › Additional Files 2/Additional Files 2_files/image37023.png]

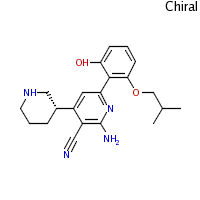

Supplement: Additional file 2 — Compounds clustered using ECFP_4 and Property Descriptors. [file 1752-0509-5-32-S2.ZIP › Additional Files 2/Additional Files 2_files/image37024.png]

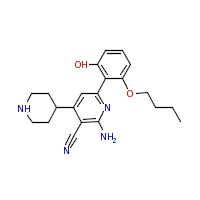

Supplement: Additional file 2 — Compounds clustered using ECFP_4 and Property Descriptors. [file 1752-0509-5-32-S2.ZIP › Additional Files 2/Additional Files 2_files/image37025.png]

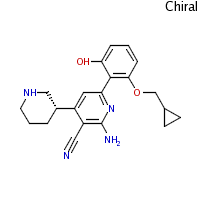

Supplement: Additional file 2 — Compounds clustered using ECFP_4 and Property Descriptors. [file 1752-0509-5-32-S2.ZIP › Additional Files 2/Additional Files 2_files/image37026.png]

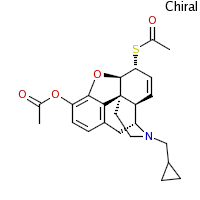

Supplement: Additional file 2 — Compounds clustered using ECFP_4 and Property Descriptors. [file 1752-0509-5-32-S2.ZIP › Additional Files 2/Additional Files 2_files/image37027.png]

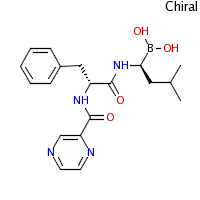

Supplement: Additional file 2 — Compounds clustered using ECFP_4 and Property Descriptors. [file 1752-0509-5-32-S2.ZIP › Additional Files 2/Additional Files 2_files/image37028.png]

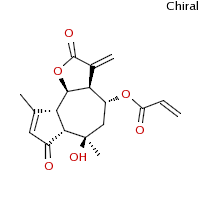

Supplement: Additional file 2 — Compounds clustered using ECFP_4 and Property Descriptors. [file 1752-0509-5-32-S2.ZIP › Additional Files 2/Additional Files 2_files/image37029.png]

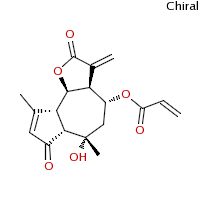

Supplement: Additional file 2 — Compounds clustered using ECFP_4 and Property Descriptors. [file 1752-0509-5-32-S2.ZIP › Additional Files 2/Additional Files 2_files/image37030.png]

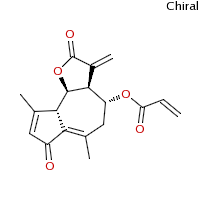

Supplement: Additional file 2 — Compounds clustered using ECFP_4 and Property Descriptors. [file 1752-0509-5-32-S2.ZIP › Additional Files 2/Additional Files 2_files/image37031.png]

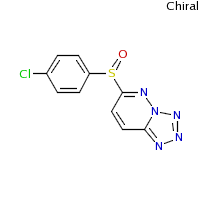

Supplement: Additional file 2 — Compounds clustered using ECFP_4 and Property Descriptors. [file 1752-0509-5-32-S2.ZIP › Additional Files 2/Additional Files 2_files/image37032.png]

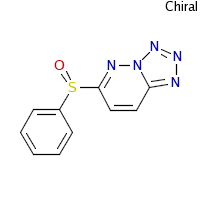

Supplement: Additional file 2 — Compounds clustered using ECFP_4 and Property Descriptors. [file 1752-0509-5-32-S2.ZIP › Additional Files 2/Additional Files 2_files/image37033.png]

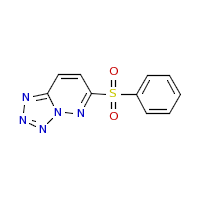

Supplement: Additional file 2 — Compounds clustered using ECFP_4 and Property Descriptors. [file 1752-0509-5-32-S2.ZIP › Additional Files 2/Additional Files 2_files/image37034.png]

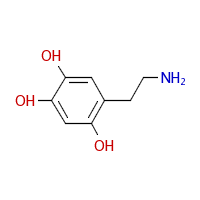

Supplement: Additional file 2 — Compounds clustered using ECFP_4 and Property Descriptors. [file 1752-0509-5-32-S2.ZIP › Additional Files 2/Additional Files 2_files/image37035.png]

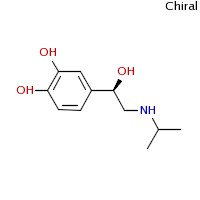

Supplement: Additional file 2 — Compounds clustered using ECFP_4 and Property Descriptors. [file 1752-0509-5-32-S2.ZIP › Additional Files 2/Additional Files 2_files/image37036.png]

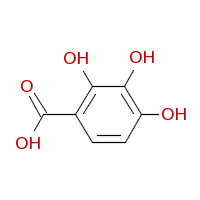

Supplement: Additional file 2 — Compounds clustered using ECFP_4 and Property Descriptors. [file 1752-0509-5-32-S2.ZIP › Additional Files 2/Additional Files 2_files/image37037.png]

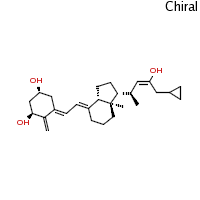

Supplement: Additional file 2 — Compounds clustered using ECFP_4 and Property Descriptors. [file 1752-0509-5-32-S2.ZIP › Additional Files 2/Additional Files 2_files/image37038.png]

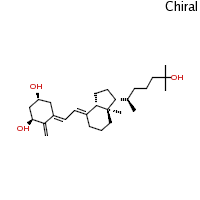

Supplement: Additional file 2 — Compounds clustered using ECFP_4 and Property Descriptors. [file 1752-0509-5-32-S2.ZIP › Additional Files 2/Additional Files 2_files/image37039.png]

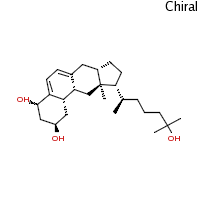

Supplement: Additional file 2 — Compounds clustered using ECFP_4 and Property Descriptors. [file 1752-0509-5-32-S2.ZIP › Additional Files 2/Additional Files 2_files/image37040.png]

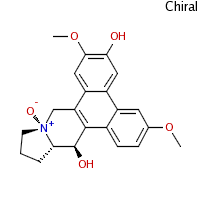

Supplement: Additional file 2 — Compounds clustered using ECFP_4 and Property Descriptors. [file 1752-0509-5-32-S2.ZIP › Additional Files 2/Additional Files 2_files/image37041.png]

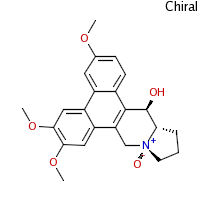

Supplement: Additional file 2 — Compounds clustered using ECFP_4 and Property Descriptors. [file 1752-0509-5-32-S2.ZIP › Additional Files 2/Additional Files 2_files/image37043.png]

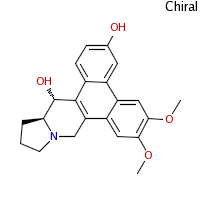

Supplement: Additional file 2 — Compounds clustered using ECFP_4 and Property Descriptors. [file 1752-0509-5-32-S2.ZIP › Additional Files 2/Additional Files 2_files/image37045.png]

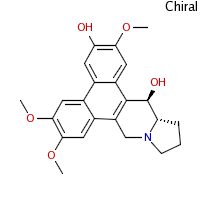

Supplement: Additional file 2 — Compounds clustered using ECFP_4 and Property Descriptors. [file 1752-0509-5-32-S2.ZIP › Additional Files 2/Additional Files 2_files/image37046.png]

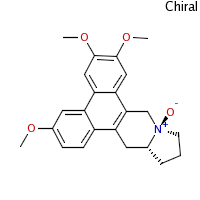

Supplement: Additional file 2 — Compounds clustered using ECFP_4 and Property Descriptors. [file 1752-0509-5-32-S2.ZIP › Additional Files 2/Additional Files 2_files/image37047.png]

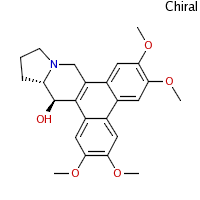

Supplement: Additional file 2 — Compounds clustered using ECFP_4 and Property Descriptors. [file 1752-0509-5-32-S2.ZIP › Additional Files 2/Additional Files 2_files/image37048.png]

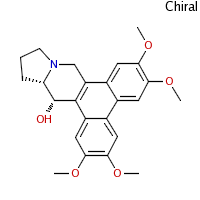

Supplement: Additional file 2 — Compounds clustered using ECFP_4 and Property Descriptors. [file 1752-0509-5-32-S2.ZIP › Additional Files 2/Additional Files 2_files/image37049.png]

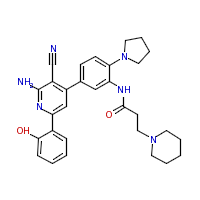

Supplement: Additional file 2 — Compounds clustered using ECFP_4 and Property Descriptors. [file 1752-0509-5-32-S2.ZIP › Additional Files 2/Additional Files 2_files/image37050.png]

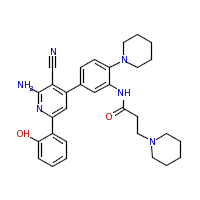

Supplement: Additional file 2 — Compounds clustered using ECFP_4 and Property Descriptors. [file 1752-0509-5-32-S2.ZIP › Additional Files 2/Additional Files 2_files/image37051.png]

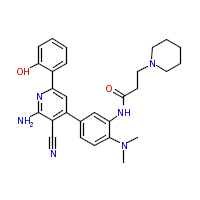

Supplement: Additional file 2 — Compounds clustered using ECFP_4 and Property Descriptors. [file 1752-0509-5-32-S2.ZIP › Additional Files 2/Additional Files 2_files/image37052.png]

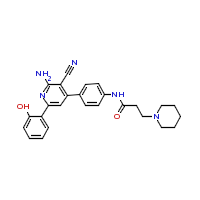

Supplement: Additional file 2 — Compounds clustered using ECFP_4 and Property Descriptors. [file 1752-0509-5-32-S2.ZIP › Additional Files 2/Additional Files 2_files/image37053.png]

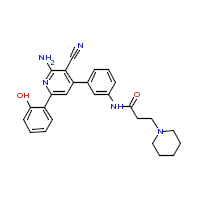

Supplement: Additional file 2 — Compounds clustered using ECFP_4 and Property Descriptors. [file 1752-0509-5-32-S2.ZIP › Additional Files 2/Additional Files 2_files/image37054.png]

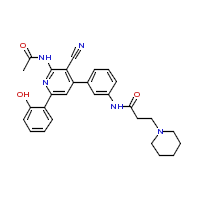

Supplement: Additional file 2 — Compounds clustered using ECFP_4 and Property Descriptors. [file 1752-0509-5-32-S2.ZIP › Additional Files 2/Additional Files 2_files/image37055.png]

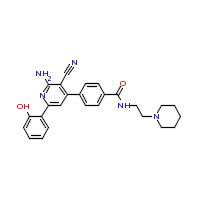

Supplement: Additional file 2 — Compounds clustered using ECFP_4 and Property Descriptors. [file 1752-0509-5-32-S2.ZIP › Additional Files 2/Additional Files 2_files/image37056.png]

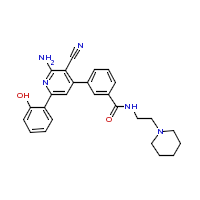

Supplement: Additional file 2 — Compounds clustered using ECFP_4 and Property Descriptors. [file 1752-0509-5-32-S2.ZIP › Additional Files 2/Additional Files 2_files/image37057.png]

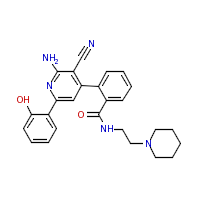

Supplement: Additional file 2 — Compounds clustered using ECFP_4 and Property Descriptors. [file 1752-0509-5-32-S2.ZIP › Additional Files 2/Additional Files 2_files/image37058.png]

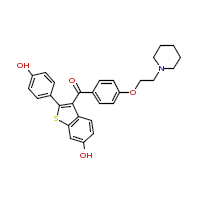

Supplement: Additional file 2 — Compounds clustered using ECFP_4 and Property Descriptors. [file 1752-0509-5-32-S2.ZIP › Additional Files 2/Additional Files 2_files/image37059.png]

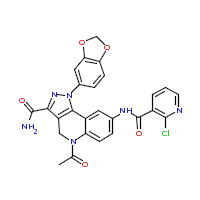

Supplement: Additional file 2 — Compounds clustered using ECFP_4 and Property Descriptors. [file 1752-0509-5-32-S2.ZIP › Additional Files 2/Additional Files 2_files/image37060.png]

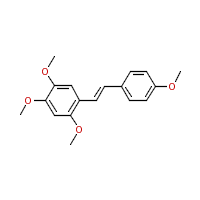

Supplement: Additional file 2 — Compounds clustered using ECFP_4 and Property Descriptors. [file 1752-0509-5-32-S2.ZIP › Additional Files 2/Additional Files 2_files/image37061.png]

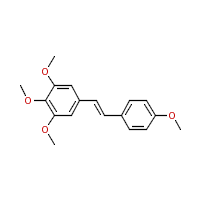

Supplement: Additional file 2 — Compounds clustered using ECFP_4 and Property Descriptors. [file 1752-0509-5-32-S2.ZIP › Additional Files 2/Additional Files 2_files/image37062.png]

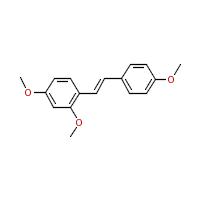

Supplement: Additional file 2 — Compounds clustered using ECFP_4 and Property Descriptors. [file 1752-0509-5-32-S2.ZIP › Additional Files 2/Additional Files 2_files/image37063.png]

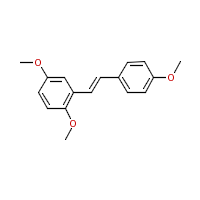

Supplement: Additional file 2 — Compounds clustered using ECFP_4 and Property Descriptors. [file 1752-0509-5-32-S2.ZIP › Additional Files 2/Additional Files 2_files/image37064.png]

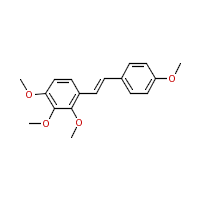

Supplement: Additional file 2 — Compounds clustered using ECFP_4 and Property Descriptors. [file 1752-0509-5-32-S2.ZIP › Additional Files 2/Additional Files 2_files/image37065.png]

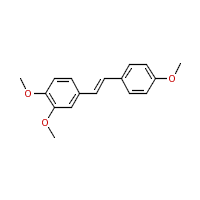

Supplement: Additional file 2 — Compounds clustered using ECFP_4 and Property Descriptors. [file 1752-0509-5-32-S2.ZIP › Additional Files 2/Additional Files 2_files/image37066.png]

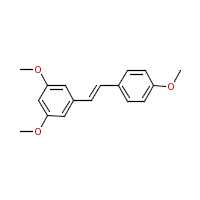

Supplement: Additional file 2 — Compounds clustered using ECFP_4 and Property Descriptors. [file 1752-0509-5-32-S2.ZIP › Additional Files 2/Additional Files 2_files/image37067.png]

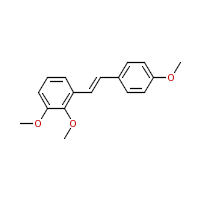

Supplement: Additional file 2 — Compounds clustered using ECFP_4 and Property Descriptors. [file 1752-0509-5-32-S2.ZIP › Additional Files 2/Additional Files 2_files/image37068.png]

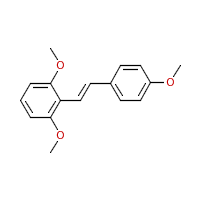

Supplement: Additional file 2 — Compounds clustered using ECFP_4 and Property Descriptors. [file 1752-0509-5-32-S2.ZIP › Additional Files 2/Additional Files 2_files/image37069.png]

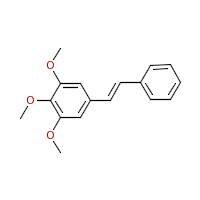

Supplement: Additional file 2 — Compounds clustered using ECFP_4 and Property Descriptors. [file 1752-0509-5-32-S2.ZIP › Additional Files 2/Additional Files 2_files/image37070.png]

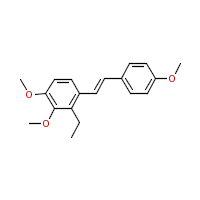

Supplement: Additional file 2 — Compounds clustered using ECFP_4 and Property Descriptors. [file 1752-0509-5-32-S2.ZIP › Additional Files 2/Additional Files 2_files/image37071.png]

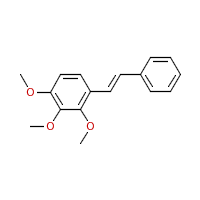

Supplement: Additional file 2 — Compounds clustered using ECFP_4 and Property Descriptors. [file 1752-0509-5-32-S2.ZIP › Additional Files 2/Additional Files 2_files/image37072.png]

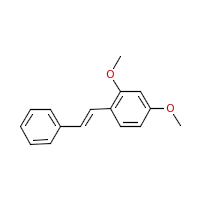

Supplement: Additional file 2 — Compounds clustered using ECFP_4 and Property Descriptors. [file 1752-0509-5-32-S2.ZIP › Additional Files 2/Additional Files 2_files/image37073.png]

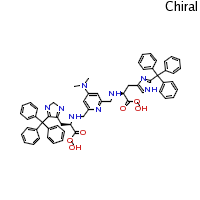

Supplement: Additional file 2 — Compounds clustered using ECFP_4 and Property Descriptors. [file 1752-0509-5-32-S2.ZIP › Additional Files 2/Additional Files 2_files/image37074.png]

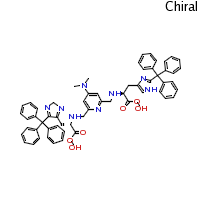

Supplement: Additional file 2 — Compounds clustered using ECFP_4 and Property Descriptors. [file 1752-0509-5-32-S2.ZIP › Additional Files 2/Additional Files 2_files/image37075.png]

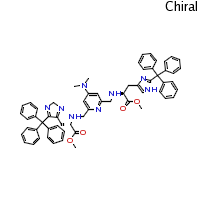

Supplement: Additional file 2 — Compounds clustered using ECFP_4 and Property Descriptors. [file 1752-0509-5-32-S2.ZIP › Additional Files 2/Additional Files 2_files/image37076.png]

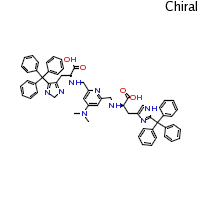

Supplement: Additional file 2 — Compounds clustered using ECFP_4 and Property Descriptors. [file 1752-0509-5-32-S2.ZIP › Additional Files 2/Additional Files 2_files/image37077.png]

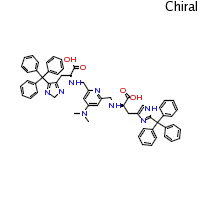

Supplement: Additional file 2 — Compounds clustered using ECFP_4 and Property Descriptors. [file 1752-0509-5-32-S2.ZIP › Additional Files 2/Additional Files 2_files/image37078.png]

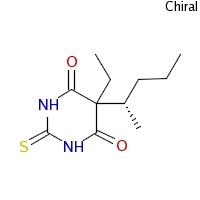

Supplement: Additional file 2 — Compounds clustered using ECFP_4 and Property Descriptors. [file 1752-0509-5-32-S2.ZIP › Additional Files 2/Additional Files 2_files/image37079.png]

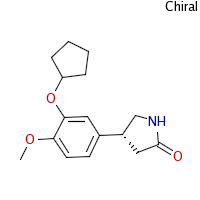

Supplement: Additional file 2 — Compounds clustered using ECFP_4 and Property Descriptors. [file 1752-0509-5-32-S2.ZIP › Additional Files 2/Additional Files 2_files/image37080.png]

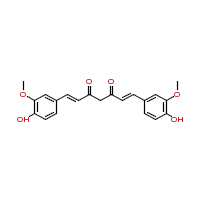

Supplement: Additional file 2 — Compounds clustered using ECFP_4 and Property Descriptors. [file 1752-0509-5-32-S2.ZIP › Additional Files 2/Additional Files 2_files/image37081.png]

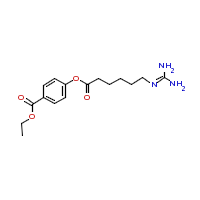

Supplement: Additional file 2 — Compounds clustered using ECFP_4 and Property Descriptors. [file 1752-0509-5-32-S2.ZIP › Additional Files 2/Additional Files 2_files/image37082.png]

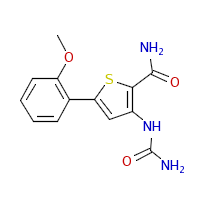

Supplement: Additional file 2 — Compounds clustered using ECFP_4 and Property Descriptors. [file 1752-0509-5-32-S2.ZIP › Additional Files 2/Additional Files 2_files/image37083.png]

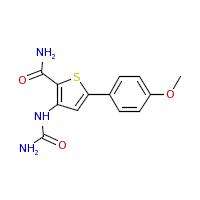

Supplement: Additional file 2 — Compounds clustered using ECFP_4 and Property Descriptors. [file 1752-0509-5-32-S2.ZIP › Additional Files 2/Additional Files 2_files/image37084.png]

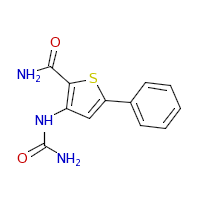

Supplement: Additional file 2 — Compounds clustered using ECFP_4 and Property Descriptors. [file 1752-0509-5-32-S2.ZIP › Additional Files 2/Additional Files 2_files/image37085.png]

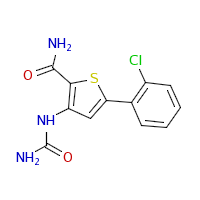

Supplement: Additional file 2 — Compounds clustered using ECFP_4 and Property Descriptors. [file 1752-0509-5-32-S2.ZIP › Additional Files 2/Additional Files 2_files/image37086.png]

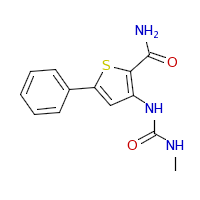

Supplement: Additional file 2 — Compounds clustered using ECFP_4 and Property Descriptors. [file 1752-0509-5-32-S2.ZIP › Additional Files 2/Additional Files 2_files/image37087.png]

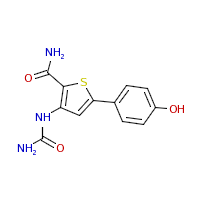

Supplement: Additional file 2 — Compounds clustered using ECFP_4 and Property Descriptors. [file 1752-0509-5-32-S2.ZIP › Additional Files 2/Additional Files 2_files/image37088.png]

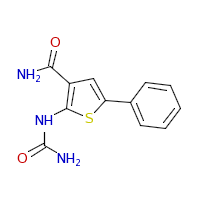

Supplement: Additional file 2 — Compounds clustered using ECFP_4 and Property Descriptors. [file 1752-0509-5-32-S2.ZIP › Additional Files 2/Additional Files 2_files/image37089.png]

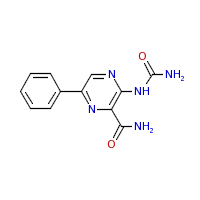

Supplement: Additional file 2 — Compounds clustered using ECFP_4 and Property Descriptors. [file 1752-0509-5-32-S2.ZIP › Additional Files 2/Additional Files 2_files/image37090.png]

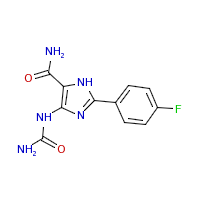

Supplement: Additional file 2 — Compounds clustered using ECFP_4 and Property Descriptors. [file 1752-0509-5-32-S2.ZIP › Additional Files 2/Additional Files 2_files/image37091.png]

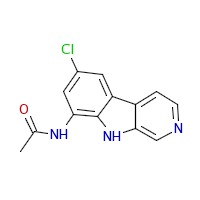

Supplement: Additional file 2 — Compounds clustered using ECFP_4 and Property Descriptors. [file 1752-0509-5-32-S2.ZIP › Additional Files 2/Additional Files 2_files/image37092.png]

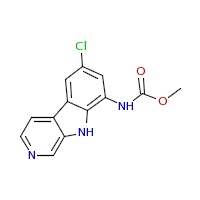

Supplement: Additional file 2 — Compounds clustered using ECFP_4 and Property Descriptors. [file 1752-0509-5-32-S2.ZIP › Additional Files 2/Additional Files 2_files/image37093.png]

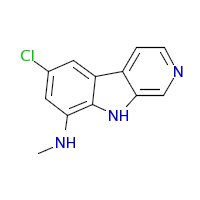

Supplement: Additional file 2 — Compounds clustered using ECFP_4 and Property Descriptors. [file 1752-0509-5-32-S2.ZIP › Additional Files 2/Additional Files 2_files/image37094.png]

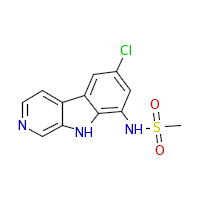

Supplement: Additional file 2 — Compounds clustered using ECFP_4 and Property Descriptors. [file 1752-0509-5-32-S2.ZIP › Additional Files 2/Additional Files 2_files/image37095.png]

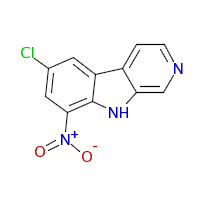

Supplement: Additional file 2 — Compounds clustered using ECFP_4 and Property Descriptors. [file 1752-0509-5-32-S2.ZIP › Additional Files 2/Additional Files 2_files/image37096.png]

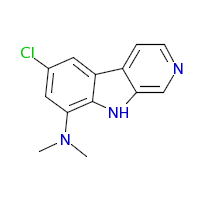

Supplement: Additional file 2 — Compounds clustered using ECFP_4 and Property Descriptors. [file 1752-0509-5-32-S2.ZIP › Additional Files 2/Additional Files 2_files/image37097.png]

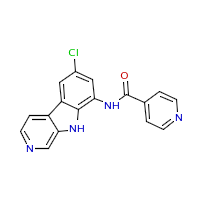

Supplement: Additional file 2 — Compounds clustered using ECFP_4 and Property Descriptors. [file 1752-0509-5-32-S2.ZIP › Additional Files 2/Additional Files 2_files/image37098.png]

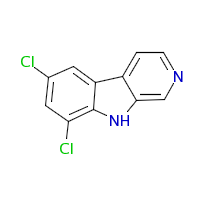

Supplement: Additional file 2 — Compounds clustered using ECFP_4 and Property Descriptors. [file 1752-0509-5-32-S2.ZIP › Additional Files 2/Additional Files 2_files/image37099.png]

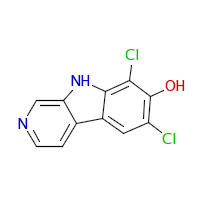

Supplement: Additional file 2 — Compounds clustered using ECFP_4 and Property Descriptors. [file 1752-0509-5-32-S2.ZIP › Additional Files 2/Additional Files 2_files/image37100.png]

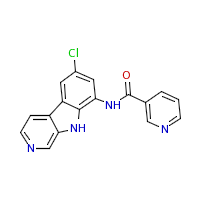

Supplement: Additional file 2 — Compounds clustered using ECFP_4 and Property Descriptors. [file 1752-0509-5-32-S2.ZIP › Additional Files 2/Additional Files 2_files/image37101.png]
