# Supplementary material for: Predicting the points of interaction of small molecules in the NF-κB pathway
Source: BMC Syst Biol. 2011 Feb 22;5:32. doi: 10.1186/1752-0509-5-32 (PMC3050742; doi:10.1186/1752-0509-5-32)
Supplement: Additional file 3 — Clusters of Compounds Shown in Figure 6. [file 1752-0509-5-32-S3.ZIP › Additional Files 3/Clustering_excluding_compounds_with_unknown_interactions.htm]

|  |  |  |  |  |  |  |  |  |  |  |
| --- | --- | --- | --- | --- | --- | --- | --- | --- | --- | --- |
| Molecule | Name | Cluster | DNA\_interaction | ROS\_interaction | activates\_IkB\_phosphorylation\_degradation | inhibits\_IKK\_activation | inhibits\_IkB\_degradation\_phosphorylation | inhibits\_translocation | Smiles | InChI |
|  | acteoside | 1 | 0 | 0 | 0 | 0 | 1 | 0 | C[C@H]1[C@@H](O)[C@H](O)[C@H](O)[C@@H](O[C@H]2[C@@H](OC(\C=C/c3cc(O)c(O)cc3)=O)[C@H](CO)O[C@@H](OCCc4cc(O)c(O)cc4)[C@@H]2O)O1 | InChI=1/C29H36O15/c1-13-22(36)23(37)24(38)29(41-13)44-27-25(39)28(40-9-8-15-3-6-17(32)19(34)11-15)42-20(12-30)26(27)43-21(35)7-4-14-2-5-16(31)18(33)10-14/h2-7,10-11,13,20,22-34,36-39H,8-9,12H2,1H3/b7-4-/t13-,20-,22+,23-,24-,25+,26-,27+,28+,29+/m0/s1 |
|  | leucosceptoside A | 1 | 0 | 0 | 0 | 0 | 1 | 0 | C[C@H]1[C@@H](O)[C@H](O)[C@H](O)[C@@H](O[C@H]2[C@@H](OC(\C=C/c3cc(OC)c(O)cc3)=O)[C@H](CO)O[C@@H](OCCc4cc(O)c(O)cc4)[C@@H]2O)O1 | InChI=1/C30H38O15/c1-14-23(36)24(37)25(38)30(42-14)45-28-26(39)29(41-10-9-16-3-6-17(32)19(34)11-16)43-21(13-31)27(28)44-22(35)8-5-15-4-7-18(33)20(12-15)40-2/h3-8,11-12,14,21,23-34,36-39H,9-10,13H2,1-2H3/b8-5-/t14-,21-,23+,24-,25-,26+,27-,28+,29+,30+/m0/s1 |
|  | acteoside isomer | 1 | 0 | 0 | 0 | 0 | 1 | 0 | C[C@H]1[C@@H](O)[C@H](O)[C@H](O)[C@@H](O[C@H]2[C@@H](CO)[C@@H](OC(\C=C/c3cc(O)c(O)cc3)=O)O[C@@H](OCCc4cc(O)c(O)cc4)[C@@H]2O)O1 | InChI=1/C29H36O15/c1-13-22(36)23(37)24(38)29(41-13)43-26-16(12-30)27(42-21(35)7-4-14-2-5-17(31)19(33)10-14)44-28(25(26)39)40-9-8-15-3-6-18(32)20(34)11-15/h2-7,10-11,13,16,22-34,36-39H,8-9,12H2,1H3/b7-4-/t13-,16+,22+,23-,24-,25+,26-,27-,28+,29+/m0/s1 |
|  | martynoside | 1 | 0 | 0 | 0 | 0 | 1 | 0 | C[C@H]1[C@@H](O)[C@H](O)[C@H](O)[C@@H](O[C@H]2[C@@H](OC(\C=C/c3cc(OC)c(O)cc3)=O)[C@H](CO)O[C@@H](OCCc4cc(O)c(OC)cc4)[C@@H]2O)O1 | InChI=1/C31H40O15/c1-15-24(36)25(37)26(38)31(43-15)46-29-27(39)30(42-11-10-17-5-8-20(40-2)19(34)12-17)44-22(14-32)28(29)45-23(35)9-6-16-4-7-18(33)21(13-16)41-3/h4-9,12-13,15,22,24-34,36-39H,10-11,14H2,1-3H3/b9-6-/t15-,22-,24+,25-,26-,27+,28-,29+,30+,31+/m0/s1 |
|  | isomartynoside | 1 | 0 | 0 | 0 | 0 | 1 | 0 | C[C@H]1[C@@H](O)[C@H](O)[C@H](O)[C@@H](O[C@H]2[C@@H](CO)[C@@H](OC(\C=C/c3cc(O)c(OC)cc3)=O)O[C@@H](OCCc4cc(O)c(OC)cc4)[C@@H]2O)O1 | InChI=1/C31H40O15/c1-15-24(36)25(37)26(38)31(43-15)45-28-18(14-32)29(44-23(35)9-6-16-4-7-21(40-2)19(33)12-16)46-30(27(28)39)42-11-10-17-5-8-22(41-3)20(34)13-17/h4-9,12-13,15,18,24-34,36-39H,10-11,14H2,1-3H3/b9-6-/t15-,18+,24+,25-,26-,27+,28-,29-,30+,31+/m0/s1 |
|  | Compound 4c | 5 | 0 | 0 | 0 | 1 | 0 | 0 | c1(C#N)c(N)nc(c2c(O)cccc2OCC)cc1C3CCNCC3 | InChI=1/C19H22N4O2/c1-2-25-17-5-3-4-16(24)18(17)15-10-13(12-6-8-22-9-7-12)14(11-20)19(21)23-15/h3-5,10,12,22,24H,2,6-9H2,1H3,(H2,21,23) |
|  | Compound 3h | 5 | 0 | 0 | 0 | 1 | 0 | 0 | c1(C#N)c(N)nc(c2c(OC)cccc2O)cc1[C@@H]3CCCNC3 | InChI=1/C18H20N4O2/c1-24-16-6-2-5-15(23)17(16)14-8-12(11-4-3-7-21-10-11)13(9-19)18(20)22-14/h2,5-6,8,11,21,23H,3-4,7,10H2,1H3,(H2,20,22)/t11-/m1/s1 |
|  | Compound 3m | 5 | 0 | 0 | 0 | 1 | 0 | 0 | c1(C#N)c(N)nc(c2c(OC(C)C)cccc2O)cc1[C@@H]3CCCNC3 | InChI=1/C20H24N4O2/c1-12(2)26-18-7-3-6-17(25)19(18)16-9-14(13-5-4-8-23-11-13)15(10-21)20(22)24-16/h3,6-7,9,12-13,23,25H,4-5,8,11H2,1-2H3,(H2,22,24)/t13-/m1/s1 |
|  | Compound 3j | 5 | 0 | 0 | 0 | 1 | 0 | 0 | c1(C#N)c(N)nc(c2c(OCCC)cccc2O)cc1[C@@H]3CCCNC3 | InChI=1/C20H24N4O2/c1-2-9-26-18-7-3-6-17(25)19(18)16-10-14(13-5-4-8-23-12-13)15(11-21)20(22)24-16/h3,6-7,10,13,23,25H,2,4-5,8-9,12H2,1H3,(H2,22,24)/t13-/m1/s1 |
|  | Compound 4b | 5 | 0 | 0 | 0 | 1 | 0 | 0 | c1(C#N)c(N)nc(c2c(O)cccc2O)cc1C3CCNCC3 | InChI=1/C17H18N4O2/c18-9-12-11(10-4-6-20-7-5-10)8-13(21-17(12)19)16-14(22)2-1-3-15(16)23/h1-3,8,10,20,22-23H,4-7H2,(H2,19,21) |
|  | Compound 4k | 5 | 0 | 0 | 0 | 1 | 0 | 0 | c1(C#N)c(N)nc(c2c(O)cccc2OC3CCC3)cc1C4CCNCC4 | InChI=1/C21H24N4O2/c22-12-16-15(13-7-9-24-10-8-13)11-17(25-21(16)23)20-18(26)5-2-6-19(20)27-14-3-1-4-14/h2,5-6,11,13-14,24,26H,1,3-4,7-10H2,(H2,23,25) |
|  | Compound 3n | 5 | 0 | 0 | 0 | 1 | 0 | 0 | c1(C#N)c(N)nc(c2c(OCC(C)C)cccc2O)cc1[C@@H]3CCCNC3 | InChI=1/C21H26N4O2/c1-13(2)12-27-19-7-3-6-18(26)20(19)17-9-15(14-5-4-8-24-11-14)16(10-22)21(23)25-17/h3,6-7,9,13-14,24,26H,4-5,8,11-12H2,1-2H3,(H2,23,25)/t14-/m1/s1 |
|  | Compound 4e | 5 | 0 | 0 | 0 | 1 | 0 | 0 | c1(C#N)c(N)nc(c2c(O)cccc2OCCCC)cc1C3CCNCC3 | InChI=1/C21H26N4O2/c1-2-3-11-27-19-6-4-5-18(26)20(19)17-12-15(14-7-9-24-10-8-14)16(13-22)21(23)25-17/h4-6,12,14,24,26H,2-3,7-11H2,1H3,(H2,23,25) |
|  | Compound 3o | 5 | 0 | 0 | 0 | 1 | 0 | 0 | c1(C#N)c(N)nc(c2c(OCC3CC3)cccc2O)cc1[C@@H]4CCCNC4 | InChI=1/C21H24N4O2/c22-10-16-15(14-3-2-8-24-11-14)9-17(25-21(16)23)20-18(26)4-1-5-19(20)27-12-13-6-7-13/h1,4-5,9,13-14,24,26H,2-3,6-8,11-12H2,(H2,23,25)/t14-/m1/s1 |
|  | Compound 4 | 8 | 1 | 0 | 0 | 0 | 0 | 0 | C=C1[C@@H]([C@@H](C[C@@]([C@H]2[C@@H]3C(C)=CC2=O)(C)O)OC(=O)C=C)[C@@H]3OC1=O | InChI=1/C18H20O6/c1-5-12(20)23-11-7-18(4,22)15-10(19)6-8(2)13(15)16-14(11)9(3)17(21)24-16/h5-6,11,13-16,22H,1,3,7H2,2,4H3/t11-,13+,14+,15-,16-,18-/m1/s1 |
|  | Compound 3 | 8 | 1 | 0 | 0 | 0 | 0 | 0 | C=C1[C@@H]([C@@H](C[C@]([C@H]2[C@@H]3C(C)=CC2=O)(C)O)OC(=O)C=C)[C@@H]3OC1=O | InChI=1/C18H20O6/c1-5-12(20)23-11-7-18(4,22)15-10(19)6-8(2)13(15)16-14(11)9(3)17(21)24-16/h5-6,11,13-16,22H,1,3,7H2,2,4H3/t11-,13+,14+,15-,16-,18+/m1/s1 |
|  | Compound 1 | 8 | 1 | 0 | 0 | 0 | 0 | 0 | C=C1[C@@H]([C@@H](CC(=C2[C@@H]3C(C)=CC2=O)C)OC(=O)C=C)[C@@H]3OC1=O | InChI=1/C18H18O5/c1-5-13(20)22-12-7-9(3)14-11(19)6-8(2)15(14)17-16(12)10(4)18(21)23-17/h5-6,12,15-17H,1,4,7H2,2-3H3/t12-,15+,16+,17-/m1/s1 |
|  | 2-active | 9 | 0 | 0 | 0 | 0 | 1 | 0 | c1(ccc(Cl)cc1)S(c2nn(nnn3)c3cc2)=O | InChI=1/C10H6ClN5OS/c11-7-1-3-8(4-2-7)18(17)10-6-5-9-12-14-15-16(9)13-10/h1-6H |
|  | Ro106-9920 | 9 | 0 | 0 | 0 | 0 | 1 | 0 | c1(ccccc1)S(c2nn(nnn3)c3cc2)=O | InChI=1/C10H7N5OS/c16-17(8-4-2-1-3-5-8)10-7-6-9-11-13-14-15(9)12-10/h1-7H |
|  | 1-active | 9 | 0 | 0 | 0 | 0 | 1 | 0 | c1(ccccc1)S(c2nn(nnn3)c3cc2)(=O)=O | InChI=1/C10H7N5O2S/c16-18(17,8-4-2-1-3-5-8)10-7-6-9-11-13-14-15(9)12-10/h1-7H |
|  | isoproterenol | 10 | 0 | 0 | 0 | 0 | 1 | 0 | Oc1c(O)cc([C@H](CNC(C)C)O)cc1 | InChI=1/C11H17NO3/c1-7(2)12-6-11(15)8-3-4-9(13)10(14)5-8/h3-5,7,11-15H,6H2,1-2H3/t11-/m0/s1 |
|  | Gallic Acid | 10 | 1 | 0 | 0 | 0 | 0 | 0 | c1cc(O)c(O)c(O)c1C(O)=O | InChI=1/C7H6O5/c8-4-2-1-3(7(11)12)5(9)6(4)10/h1-2,8-10H,(H,11,12) |
|  | 1,24-dihydroxy-22-ene-24-cyclopropyl-vitamin D | 11 | 0 | 0 | 1 | 0 | 0 | 0 | C[C@@H]([C@@H]1[C@]2(C)[C@H](\C(=C\C=C(\C(=C)[C@@H](O)C[C@@H]3O)/C3)\CCC2)CC1)\C=C(/CC4CC4)\O | InChI=1/C27H40O3/c1-17(13-22(28)14-19-6-7-19)24-10-11-25-20(5-4-12-27(24,25)3)8-9-21-15-23(29)16-26(30)18(21)2/h8-9,13,17,19,23-26,28-30H,2,4-7,10-12,14-16H2,1,3H3/b20-8+,21-9+,22-13+/t17-,23-,24-,25+,26+,27-/m1/s1 |
|  | 1,25-dihydroxyvitamin D3 | 11 | 0 | 0 | 1 | 0 | 0 | 0 | C[C@@H]([C@@H]1[C@]2(C)[C@H](\C(=C\C=C(\C(=C)[C@@H](O)C[C@@H]3O)/C3)\CCC2)CC1)CCCC(O)(C)C | InChI=1/C27H44O3/c1-18(8-6-14-26(3,4)30)23-12-13-24-20(9-7-15-27(23,24)5)10-11-21-16-22(28)17-25(29)19(21)2/h10-11,18,22-25,28-30H,2,6-9,12-17H2,1,3-5H3/b20-10+,21-11+/t18-,22-,23-,24+,25+,27-/m1/s1 |
|  | 1a,25-dihydroxylumisterol | 11 | 0 | 0 | 1 | 0 | 0 | 0 | C[C@@H]([C@@H]1[C@@](C)(C[C@H]([C@H]2C(=C3)[C@@H](O)C[C@H](O)C2)C(=C3)C4)[C@H]4CC1)CCCC(C)(C)O | InChI=1/C26H42O3/c1-16(6-5-11-25(2,3)29)23-10-8-18-12-17-7-9-20-21(13-19(27)14-24(20)28)22(17)15-26(18,23)4/h7,9,16,18-19,21-24,27-29H,5-6,8,10-15H2,1-4H3/t16-,18+,19-,21-,22+,23-,24+,26+/m1/s1 |
|  | Compound 13 | 13 | 0 | 0 | 0 | 1 | 0 | 0 | c1(ccccc1O)c2cc(c3ccc(N4CCCC4)c(NC(=O)CCN5CCCCC5)c3)c(C#N)c(N)n2 | InChI=1/C30H34N6O2/c31-20-24-23(19-25(34-30(24)32)22-8-2-3-9-28(22)37)21-10-11-27(36-15-6-7-16-36)26(18-21)33-29(38)12-17-35-13-4-1-5-14-35/h2-3,8-11,18-19,37H,1,4-7,12-17H2,(H2,32,34)(H,33,38) |
|  | Compound 43 | 13 | 0 | 0 | 0 | 1 | 0 | 0 | c1(ccccc1O)c2cc(c3ccc(N4CCCCC4)c(NC(=O)CCN5CCCCC5)c3)c(C#N)c(N)n2 | InChI=1/C31H36N6O2/c32-21-25-24(20-26(35-31(25)33)23-9-3-4-10-29(23)38)22-11-12-28(37-16-7-2-8-17-37)27(19-22)34-30(39)13-18-36-14-5-1-6-15-36/h3-4,9-12,19-20,38H,1-2,5-8,13-18H2,(H2,33,35)(H,34,39) |
|  | Compound 12 | 13 | 0 | 0 | 0 | 1 | 0 | 0 | c1(ccccc1O)c2cc(c3ccc(N(C)C)c(NC(=O)CCN4CCCCC4)c3)c(C#N)c(N)n2 | InChI=1/C28H32N6O2/c1-33(2)25-11-10-19(16-24(25)31-27(36)12-15-34-13-6-3-7-14-34)21-17-23(32-28(30)22(21)18-29)20-8-4-5-9-26(20)35/h4-5,8-11,16-17,35H,3,6-7,12-15H2,1-2H3,(H2,30,32)(H,31,36) |
|  | Compound 27 | 13 | 0 | 0 | 0 | 1 | 0 | 0 | c1(ccccc1O)c2cc(c3ccc(NC(CCN4CCCCC4)=O)cc3)c(C#N)c(N)n2 | InChI=1/C26H27N5O2/c27-17-22-21(16-23(30-26(22)28)20-6-2-3-7-24(20)32)18-8-10-19(11-9-18)29-25(33)12-15-31-13-4-1-5-14-31/h2-3,6-11,16,32H,1,4-5,12-15H2,(H2,28,30)(H,29,33) |
|  | Compound 26 | 13 | 0 | 0 | 0 | 1 | 0 | 0 | c1(ccccc1O)c2cc(c3cccc(NC(=O)CCN4CCCCC4)c3)c(C#N)c(N)n2 | InChI=1/C26H27N5O2/c27-17-22-21(16-23(30-26(22)28)20-9-2-3-10-24(20)32)18-7-6-8-19(15-18)29-25(33)11-14-31-12-4-1-5-13-31/h2-3,6-10,15-16,32H,1,4-5,11-14H2,(H2,28,30)(H,29,33) |
|  | Compound 42 | 13 | 0 | 0 | 0 | 1 | 0 | 0 | c1(ccccc1O)c2cc(c3cccc(NC(=O)CCN4CCCCC4)c3)c(C#N)c(NC(=O)C)n2 | InChI=1/C28H29N5O3/c1-19(34)30-28-24(18-29)23(17-25(32-28)22-10-3-4-11-26(22)35)20-8-7-9-21(16-20)31-27(36)12-15-33-13-5-2-6-14-33/h3-4,7-11,16-17,35H,2,5-6,12-15H2,1H3,(H,31,36)(H,30,32,34) |
|  | Compound 30 | 13 | 0 | 0 | 0 | 1 | 0 | 0 | c1(ccccc1O)c2cc(c3ccc(C(=O)NCCN4CCCCC4)cc3)c(C#N)c(N)n2 | InChI=1/C26H27N5O2/c27-17-22-21(16-23(30-25(22)28)20-6-2-3-7-24(20)32)18-8-10-19(11-9-18)26(33)29-12-15-31-13-4-1-5-14-31/h2-3,6-11,16,32H,1,4-5,12-15H2,(H2,28,30)(H,29,33) |
|  | Compound 29 | 13 | 0 | 0 | 0 | 1 | 0 | 0 | c1(ccccc1O)c2cc(c3cccc(C(=O)NCCN4CCCCC4)c3)c(C#N)c(N)n2 | InChI=1/C26H27N5O2/c27-17-22-21(16-23(30-25(22)28)20-9-2-3-10-24(20)32)18-7-6-8-19(15-18)26(33)29-11-14-31-12-4-1-5-13-31/h2-3,6-10,15-16,32H,1,4-5,11-14H2,(H2,28,30)(H,29,33) |
|  | Compound 28 | 13 | 0 | 0 | 0 | 1 | 0 | 0 | c1(ccccc1O)c2cc(c3ccccc3C(=O)NCCN4CCCCC4)c(C#N)c(N)n2 | InChI=1/C26H27N5O2/c27-17-22-21(16-23(30-25(22)28)20-10-4-5-11-24(20)32)18-8-2-3-9-19(18)26(33)29-12-15-31-13-6-1-7-14-31/h2-5,8-11,16,32H,1,6-7,12-15H2,(H2,28,30)(H,29,33) |
|  | Pyrazolo[4,3-c]quinoline derivative | 13 | 0 | 0 | 0 | 1 | 0 | 0 | c12c(n(c3cc(OCO4)c4cc3)nc1C(=O)N)c5c(ccc(NC(=O)c6c(Cl)nccc6)c5)N(C(=O)C)C2 | InChI=1/C26H19ClN6O5/c1-13(34)32-11-18-22(25(28)35)31-33(15-5-7-20-21(10-15)38-12-37-20)23(18)17-9-14(4-6-19(17)32)30-26(36)16-3-2-8-29-24(16)27/h2-10H,11-12H2,1H3,(H2,28,35)(H,30,36) |
|  | N,N'-[4-(dimethylamino)-2,6-pyridinylidenedimethyl] bis (S,S) (2'-tritylperoxyhistidine) | 15 | 1 | 0 | 0 | 0 | 0 | 0 | c1c(CN[C@@H](C(OO)=O)CC2=NCN=C2C(c3ccccc3)(c4ccccc4)c5ccccc5)nc(CN[C@H](C(OO)=O)Cc6c[nH]c(C(c7ccccc7)(c8ccccc8)c9ccccc9)n6)cc1N(C)C | InChI=1/C59H56N8O6/c1-67(2)50-33-47(37-60-52(55(68)72-70)35-49-39-62-57(66-49)59(44-27-15-6-16-28-44,45-29-17-7-18-30-45)46-31-19-8-20-32-46)65-48(34-50)38-61-53(56(69)73-71)36-51-54(64-40-63-51)58(41-21-9-3-10-22-41,42-23-11-4-12-24-42)43-25-13-5-14-26-43/h3-34,39,52-53,60-61,70-71H,35-38,40H2,1-2H3,(H,62,66)/t52-,53+/m0/s1 |
|  | N,N'-[4-(dimethylamino)-2,6-pyridinylidenedimethyl] bis (R,S) (2'-tritylperoxyhistidine) | 15 | 1 | 0 | 0 | 0 | 0 | 0 | c1c(CN[C@H](C(OO)=O)CC2=NCN=C2C(c3ccccc3)(c4ccccc4)c5ccccc5)nc(CN[C@H](C(OO)=O)Cc6c[nH]c(C(c7ccccc7)(c8ccccc8)c9ccccc9)n6)cc1N(C)C | InChI=1/C59H56N8O6/c1-67(2)50-33-47(37-60-52(55(68)72-70)35-49-39-62-57(66-49)59(44-27-15-6-16-28-44,45-29-17-7-18-30-45)46-31-19-8-20-32-46)65-48(34-50)38-61-53(56(69)73-71)36-51-54(64-40-63-51)58(41-21-9-3-10-22-41,42-23-11-4-12-24-42)43-25-13-5-14-26-43/h3-34,39,52-53,60-61,70-71H,35-38,40H2,1-2H3,(H,62,66)/t52-,53-/m0/s1 |
|  | N,N'-[4-(dimethylamino)-2,6-pyridinylidenedimethyl] bis (S,S) (2'-tritylhistidine)-dimethylester | 15 | 1 | 0 | 0 | 0 | 0 | 0 | c1c(CN[C@H](C(OC)=O)CC2=NCN=C2C(c3ccccc3)(c4ccccc4)c5ccccc5)nc(CN[C@@H](C(OC)=O)Cc6c[nH]c(C(c7ccccc7)(c8ccccc8)c9ccccc9)n6)cc1N(C)C | InChI=1/C61H60N8O4/c1-69(2)52-35-49(39-62-54(57(70)72-3)37-51-41-64-59(68-51)61(46-29-17-8-18-30-46,47-31-19-9-20-32-47)48-33-21-10-22-34-48)67-50(36-52)40-63-55(58(71)73-4)38-53-56(66-42-65-53)60(43-23-11-5-12-24-43,44-25-13-6-14-26-44)45-27-15-7-16-28-45/h5-36,41,54-55,62-63H,37-40,42H2,1-4H3,(H,64,68)/t54-,55+/m1/s1 |
|  | thiophenecarboxamide\_baxter\_16 | 20 | 0 | 0 | 0 | 1 | 0 | 0 | COc1c(c2sc(C(=O)N)c(NC(=O)N)c2)cccc1 | InChI=1/C13H13N3O3S/c1-19-9-5-3-2-4-7(9)10-6-8(16-13(15)18)11(20-10)12(14)17/h2-6H,1H3,(H2,14,17)(H3,15,16,18) |
|  | thiophenecarboxamide\_baxter\_18 | 20 | 0 | 0 | 0 | 1 | 0 | 0 | COc1ccc(c2sc(C(=O)N)c(NC(=O)N)c2)cc1 | InChI=1/C13H13N3O3S/c1-19-8-4-2-7(3-5-8)10-6-9(16-13(15)18)11(20-10)12(14)17/h2-6H,1H3,(H2,14,17)(H3,15,16,18) |
|  | thiophenecarboxamide\_baxter\_4 | 20 | 0 | 0 | 0 | 1 | 0 | 0 | NC(Nc1c(C(=O)N)sc(c2ccccc2)c1)=O | InChI=1/C12H11N3O2S/c13-11(16)10-8(15-12(14)17)6-9(18-10)7-4-2-1-3-5-7/h1-6H,(H2,13,16)(H3,14,15,17) |
|  | thiophenecarboxamide\_baxter\_19 | 20 | 0 | 0 | 0 | 1 | 0 | 0 | NC(Nc1c(C(=O)N)sc(c2c(Cl)cccc2)c1)=O | InChI=1/C12H10ClN3O2S/c13-7-4-2-1-3-6(7)9-5-8(16-12(15)18)10(19-9)11(14)17/h1-5H,(H2,14,17)(H3,15,16,18) |
|  | thiophenecarboxamide\_baxter\_8 | 20 | 0 | 0 | 0 | 1 | 0 | 0 | CNC(Nc1c(C(=O)N)sc(c2ccccc2)c1)=O | InChI=1/C13H13N3O2S/c1-15-13(18)16-9-7-10(19-11(9)12(14)17)8-5-3-2-4-6-8/h2-7H,1H3,(H2,14,17)(H2,15,16,18) |
|  | thiophenecarboxamide\_baxter\_21 | 20 | 0 | 0 | 0 | 1 | 0 | 0 | NC(Nc1c(C(=O)N)sc(c2ccc(O)cc2)c1)=O | InChI=1/C12H11N3O3S/c13-11(17)10-8(15-12(14)18)5-9(19-10)6-1-3-7(16)4-2-6/h1-5,16H,(H2,13,17)(H3,14,15,18) |
|  | thiophenecarboxamide\_baxter\_13 | 20 | 0 | 0 | 0 | 1 | 0 | 0 | NC(Nc1c(C(=O)N)cc(c2ccccc2)s1)=O | InChI=1/C12H11N3O2S/c13-10(16)8-6-9(7-4-2-1-3-5-7)18-11(8)15-12(14)17/h1-6H,(H2,13,16)(H3,14,15,17) |
|  | thiophenecarboxamide\_baxter\_15 | 20 | 0 | 0 | 0 | 1 | 0 | 0 | NC(Nc1c(C(=O)N)nc(c2ccccc2)cn1)=O | InChI=1/C12H11N5O2/c13-10(18)9-11(17-12(14)19)15-6-8(16-9)7-4-2-1-3-5-7/h1-6H,(H2,13,18)(H3,14,15,17,19) |
|  | thiophenecarboxamide\_baxter\_14 | 20 | 0 | 0 | 0 | 1 | 0 | 0 | NC(Nc1c(C(=O)N)[nH]c(c2ccc(F)cc2)n1)=O | InChI=1/C11H10FN5O2/c12-6-3-1-5(2-4-6)9-15-7(8(13)18)10(16-9)17-11(14)19/h1-4H,(H2,13,18)(H,15,16)(H3,14,17,19) |
|  | beta Carboline 21 | 21 | 0 | 0 | 0 | 1 | 0 | 0 | c12c(ccnc1)c(cc(Cl)cc3NC(=O)C)c3[nH]2 | InChI=1/C13H10ClN3O/c1-7(18)16-11-5-8(14)4-10-9-2-3-15-6-12(9)17-13(10)11/h2-6,17H,1H3,(H,16,18) |
|  | beta Carboline 33 | 21 | 0 | 0 | 0 | 1 | 0 | 0 | c12c(ccnc1)c(cc(Cl)cc3NC(=O)OC)c3[nH]2 | InChI=1/C13H10ClN3O2/c1-19-13(18)17-10-5-7(14)4-9-8-2-3-15-6-11(8)16-12(9)10/h2-6,16H,1H3,(H,17,18) |
|  | beta Carboline 18 | 21 | 0 | 0 | 0 | 1 | 0 | 0 | c12c(ccnc1)c(cc(Cl)cc3NC)c3[nH]2 | InChI=1/C12H10ClN3/c1-14-10-5-7(13)4-9-8-2-3-15-6-11(8)16-12(9)10/h2-6,14,16H,1H3 |
|  | beta Carboline 31 | 21 | 0 | 0 | 0 | 1 | 0 | 0 | c12c(ccnc1)c(cc(Cl)cc3NS(=O)(=O)C)c3[nH]2 | InChI=1/C12H10ClN3O2S/c1-19(17,18)16-10-5-7(13)4-9-8-2-3-14-6-11(8)15-12(9)10/h2-6,15-16H,1H3 |
|  | beta Carboline 16 | 21 | 0 | 0 | 0 | 1 | 0 | 0 | c12c(ccnc1)c(cc(Cl)cc3[N+]([O-])=O)c3[nH]2 | InChI=1/C11H6ClN3O2/c12-6-3-8-7-1-2-13-5-9(7)14-11(8)10(4-6)15(16)17/h1-5,14H |
|  | beta Carboline 19 | 21 | 0 | 0 | 0 | 1 | 0 | 0 | c12c(ccnc1)c(cc(Cl)cc3N(C)C)c3[nH]2 | InChI=1/C13H12ClN3/c1-17(2)12-6-8(14)5-10-9-3-4-15-7-11(9)16-13(10)12/h3-7,16H,1-2H3 |
|  | beta Carboline 27 | 21 | 0 | 0 | 0 | 1 | 0 | 0 | c12c(ccnc1)c(cc(Cl)cc3NC(=O)c4ccncc4)c3[nH]2 | InChI=1/C17H11ClN4O/c18-11-7-13-12-3-6-20-9-15(12)21-16(13)14(8-11)22-17(23)10-1-4-19-5-2-10/h1-9,21H,(H,22,23) |
|  | beta-Carboline 9 | 21 | 0 | 0 | 0 | 1 | 0 | 0 | c12c(ccnc1)c(cc(Cl)cc3Cl)c3[nH]2 | InChI=1/C11H6Cl2N2/c12-6-3-8-7-1-2-14-5-10(7)15-11(8)9(13)4-6/h1-5,15H |
|  | beta Carboline 15 | 21 | 0 | 0 | 0 | 1 | 0 | 0 | c12c(ccnc1)c(cc(Cl)c(O)c3Cl)c3[nH]2 | InChI=1/C11H6Cl2N2O/c12-7-3-6-5-1-2-14-4-8(5)15-10(6)9(13)11(7)16/h1-4,15-16H |
|  | beta Carboline 26 | 21 | 0 | 0 | 0 | 1 | 0 | 0 | c12c(ccnc1)c(cc(Cl)cc3NC(=O)c4cnccc4)c3[nH]2 | InChI=1/C17H11ClN4O/c18-11-6-13-12-3-5-20-9-15(12)21-16(13)14(7-11)22-17(23)10-2-1-4-19-8-10/h1-9,21H,(H,22,23) |
|  | PS-1145 | 21 | 0 | 0 | 0 | 1 | 0 | 0 | c12c(c3c(cncc3)[nH]1)cc(Cl)cc2NC(=O)c4cccnc4 | InChI=1/C17H11ClN4O/c18-11-6-13-12-3-5-20-9-15(12)21-16(13)14(7-11)22-17(23)10-2-1-4-19-8-10/h1-9,21H,(H,22,23) |
|  | beta Carboline 22 | 21 | 0 | 0 | 0 | 1 | 0 | 0 | c12c(ccnc1)c(cc(Cl)cc3NC(=O)CCCO)c3[nH]2 | InChI=1/C15H14ClN3O2/c16-9-6-11-10-3-4-17-8-13(10)19-15(11)12(7-9)18-14(21)2-1-5-20/h3-4,6-8,19-20H,1-2,5H2,(H,18,21) |
|  | beta-Carboline 6 | 21 | 0 | 0 | 0 | 1 | 0 | 0 | c12c(ccnc1)c(cc(Cl)cc3)c3[nH]2 | InChI=1/C11H7ClN2/c12-7-1-2-10-9(5-7)8-3-4-13-6-11(8)14-10/h1-6,14H |
|  | beta-Carboline 8 | 21 | 0 | 0 | 0 | 1 | 0 | 0 | c12c(ccnc1)c(cc(C#N)cc3)c3[nH]2 | InChI=1/C12H7N3/c13-6-8-1-2-11-10(5-8)9-3-4-14-7-12(9)15-11/h1-5,7,15H |
|  | beta-Carboline 3 | 21 | 0 | 0 | 0 | 1 | 0 | 0 | c12c(ccnc1)c(cc(OC)cc3)c3[nH]2 | InChI=1/C12H10N2O/c1-15-8-2-3-11-10(6-8)9-4-5-13-7-12(9)14-11/h2-7,14H,1H3 |
|  | 5-bromo-6-methoxy-b-carboline | 21 | 0 | 0 | 0 | 1 | 0 | 0 | c12c(ccnc1)c(c(Br)c(O)cc3)c3[nH]2 | InChI=1/C11H7BrN2O/c12-11-9(15)2-1-7-10(11)6-3-4-13-5-8(6)14-7/h1-5,14-15H |
|  | beta-Carboline 5 | 21 | 0 | 0 | 0 | 1 | 0 | 0 | c12c(ccnc1)c(cc(Br)cc3)c3[nH]2 | InChI=1/C11H7BrN2/c12-7-1-2-10-9(5-7)8-3-4-13-6-11(8)14-10/h1-6,14H |
|  | beta-Carboline 2 | 21 | 0 | 0 | 0 | 1 | 0 | 0 | c12c(ccnc1)c(cccc3)c3[nH]2 | InChI=1/C11H8N2/c1-2-4-10-8(3-1)9-5-6-12-7-11(9)13-10/h1-7,13H |
|  | beta-Carboline 7 | 21 | 0 | 0 | 0 | 1 | 0 | 0 | c12c(ccnc1)c(cc(C(F)(F)F)cc3)c3[nH]2 | InChI=1/C12H7F3N2/c13-12(14,15)7-1-2-10-9(5-7)8-3-4-16-6-11(8)17-10/h1-6,17H |
|  | beta Carboline 35 | 21 | 0 | 0 | 0 | 1 | 0 | 0 | c12c(ccnc1)c(cc(Cl)cc3NC(O)=N(CCOC4)C4)c3[nH]2 | InChI=1/C16H16ClN4O2/c17-10-7-12-11-1-2-18-9-14(11)19-15(12)13(8-10)20-16(22)21-3-5-23-6-4-21/h1-2,7-9,19-20,22H,3-6H2 |
|  | Benzoimidazole carboxamide | 23 | 0 | 0 | 0 | 1 | 0 | 0 | c1nccc(c2[nH]c(ccc(C(=O)N[C@@H](C(=O)N)Cc3ccccc3)c4)c4n2)c1 | InChI=1/C22H19N5O2/c23-20(28)19(12-14-4-2-1-3-5-14)27-22(29)16-6-7-17-18(13-16)26-21(25-17)15-8-10-24-11-9-15/h1-11,13,19H,12H2,(H2,23,28)(H,25,26)(H,27,29)/t19-/m1/s1 |
|  | Indolecarboxamide derivative | 23 | 0 | 0 | 0 | 1 | 0 | 0 | c1nccc(c2[nH]c(ccc(C(=O)N[C@@H](C(=O)N)CSc3ccccc3)c4)c4c2)c1 | InChI=1/C23H20N4O2S/c24-22(28)21(14-30-18-4-2-1-3-5-18)27-23(29)16-6-7-19-17(12-16)13-20(26-19)15-8-10-25-11-9-15/h1-13,21,26H,14H2,(H2,24,28)(H,27,29)/t21-/m1/s1 |
|  | Compound 18 | 26 | 0 | 0 | 0 | 1 | 0 | 0 | c1(ccccc1O)c2cc(c3ccccc3C([O-])=O)c(C#N)c(N)n2.[Na] | InChI=1/C19H13N3O3.Na.H/c20-10-15-14(11-5-1-2-6-12(11)19(24)25)9-16(22-18(15)21)13-7-3-4-8-17(13)23;;/h1-9,23H,(H2,21,22)(H,24,25);;/p-1 |
|  | Compound 19 | 26 | 0 | 0 | 0 | 1 | 0 | 0 | c1(ccccc1O)c2cc(c3cccc(C([O-])=O)c3)c(C#N)c(N)n2.[Na] | InChI=1/C19H13N3O3.Na.H/c20-10-15-14(11-4-3-5-12(8-11)19(24)25)9-16(22-18(15)21)13-6-1-2-7-17(13)23;;/h1-9,23H,(H2,21,22)(H,24,25);;/p-1 |
|  | Compound 20 | 26 | 0 | 0 | 0 | 1 | 0 | 0 | c1(ccccc1O)c2cc(c3ccc(C([O-])=O)cc3)c(C#N)c(N)n2.[Na] | InChI=1/C19H13N3O3.Na.H/c20-10-15-14(11-5-7-12(8-6-11)19(24)25)9-16(22-18(15)21)13-3-1-2-4-17(13)23;;/h1-9,23H,(H2,21,22)(H,24,25);;/p-1 |
|  | Compound 2 | 26 | 0 | 0 | 0 | 1 | 0 | 0 | c1(C#N)c(N)nc(c2ccccc2O)cc1[C@@H](Cc3ccccc3)N | InChI=1/C20H18N4O/c21-12-16-15(17(22)10-13-6-2-1-3-7-13)11-18(24-20(16)23)14-8-4-5-9-19(14)25/h1-9,11,17,25H,10,22H2,(H2,23,24)/t17-/m1/s1 |
|  | Wedelolactone | 30 | 0 | 0 | 0 | 1 | 0 | 0 | c12c([C@H]3[C@@H](c(cc(O)c(O)c4)c4O3)C(O1)=O)c(O)cc(O)c2 | InChI=1/C15H10O7/c16-5-1-9(19)13-11(2-5)22-15(20)12-6-3-7(17)8(18)4-10(6)21-14(12)13/h1-4,12,14,16-19H/t12-,14-/m1/s1 |
|  | quercetin | 30 | 0 | 0 | 0 | 1 | 0 | 0 | O=C1C(O)=C(c2cc(O)c(O)cc2)Oc(cc(cc3O)O)c13 | InChI=1/C15H10O7/c16-7-4-10(19)12-11(5-7)22-15(14(21)13(12)20)6-1-2-8(17)9(18)3-6/h1-5,16-19,21H |
|  | fern-9(11)ene | 39 | 0 | 0 | 0 | 0 | 1 | 0 | CC([C@@H]1[C@](CC[C@]2([C@@]3(C)CC=C([C@@](C)(CCCC4(C)C)[C@@H]4CC5)[C@@H]25)C)(C)[C@@H]3CC1)C | InChI=1/C30H50/c1-20(2)21-10-13-25-28(21,6)18-19-29(7)23-11-12-24-26(3,4)15-9-16-27(24,5)22(23)14-17-30(25,29)8/h14,20-21,23-25H,9-13,15-19H2,1-8H3/t21-,23+,24-,25+,27-,28-,29-,30+/m1/s1 |
|  | dryocrassol | 39 | 0 | 0 | 0 | 0 | 1 | 0 | C([C@H]([C@@H]1[C@]2(C)C(=C(CC[C@H]([C@@](C)(CCCC3(C)C)[C@@H]3CC4)[C@@]45C)[C@@]5(C)CC2)CC1)C)O | InChI=1/C30H50O/c1-20(19-31)21-9-10-22-23-11-12-25-28(5)15-8-14-26(2,3)24(28)13-16-30(25,7)29(23,6)18-17-27(21,22)4/h20-21,24-25,31H,8-19H2,1-7H3/t20-,21-,24-,25-,27-,28+,29-,30-/m1/s1 |
|  | Compound 24 | 51 | 0 | 0 | 0 | 1 | 0 | 0 | c1(ccccc1O)c2cc(c3cccc(NC(=O)[C@H](CCC(O)=O)N)c3)c(C#N)c(N)n2 | InChI=1/C23H21N5O4/c24-12-17-16(11-19(28-22(17)26)15-6-1-2-7-20(15)29)13-4-3-5-14(10-13)27-23(32)18(25)8-9-21(30)31/h1-7,10-11,18,29H,8-9,25H2,(H2,26,28)(H,27,32)(H,30,31)/t18-/m0/s1 |
|  | Compound 25 | 51 | 0 | 0 | 0 | 1 | 0 | 0 | c1(ccccc1O)c2cc(c3cccc(NC(=O)C[C@@H](N)CC(O)=O)c3)c(C#N)c(N)n2 | InChI=1/C23H21N5O4/c24-12-18-17(11-19(28-23(18)26)16-6-1-2-7-20(16)29)13-4-3-5-15(8-13)27-21(30)9-14(25)10-22(31)32/h1-8,11,14,29H,9-10,25H2,(H2,26,28)(H,27,30)(H,31,32)/t14-/m1/s1 |
|  | Compound 6 | 51 | 0 | 0 | 0 | 1 | 0 | 0 | c1(ccccc1O)c2cc(c3cccc(NC(=O)[C@@H](O)CCC([O-])=O)c3)c(C#N)c(N)n2.[Na+] | InChI=1/C23H20N4O5.Na/c24-12-17-16(11-18(27-22(17)25)15-6-1-2-7-19(15)28)13-4-3-5-14(10-13)26-23(32)20(29)8-9-21(30)31;/h1-7,10-11,20,28-29H,8-9H2,(H2,25,27)(H,26,32)(H,30,31);/q;+1/p-1/t20-;/m0./s1 |
|  | Compound 36 | 51 | 0 | 0 | 0 | 1 | 0 | 0 | c1(cc(OC)ccc1O)c2cc(c3cccc(NC(=O)[C@@H](O)CCC([O-])=O)c3)c(C#N)c(N)n2.[Na+] | InChI=1/C24H22N4O6.Na/c1-34-15-5-6-20(29)17(10-15)19-11-16(18(12-25)23(26)28-19)13-3-2-4-14(9-13)27-24(33)21(30)7-8-22(31)32;/h2-6,9-11,21,29-30H,7-8H2,1H3,(H2,26,28)(H,27,33)(H,31,32);/q;+1/p-1/t21-;/m0./s1 |
|  | Compound 38 | 51 | 0 | 0 | 0 | 1 | 0 | 0 | c1(c(OC)cccc1O)c2cc(c3cccc(NC(=O)[C@@H](O)CCC([O-])=O)c3)c(C#N)c(N)n2.[Na+] | InChI=1/C24H22N4O6.Na/c1-34-20-7-3-6-18(29)22(20)17-11-15(16(12-25)23(26)28-17)13-4-2-5-14(10-13)27-24(33)19(30)8-9-21(31)32;/h2-7,10-11,19,29-30H,8-9H2,1H3,(H2,26,28)(H,27,33)(H,31,32);/q;+1/p-1/t19-;/m0./s1 |
|  | Compound 35 | 51 | 0 | 0 | 0 | 1 | 0 | 0 | c1(cc(Cl)ccc1O)c2cc(c3cccc(NC(=O)[C@@H](O)CCC([O-])=O)c3)c(C#N)c(N)n2.[Na+] | InChI=1/C23H19ClN4O5.Na/c24-13-4-5-19(29)16(9-13)18-10-15(17(11-25)22(26)28-18)12-2-1-3-14(8-12)27-23(33)20(30)6-7-21(31)32;/h1-5,8-10,20,29-30H,6-7H2,(H2,26,28)(H,27,33)(H,31,32);/q;+1/p-1/t20-;/m0./s1 |
|  | Compound 3l | 60 | 0 | 0 | 0 | 1 | 0 | 0 | c1(C#N)c(N)nc(c2c(OCCCCCCC)cccc2O)cc1[C@@H]3CCCNC3 | InChI=1/C24H32N4O2/c1-2-3-4-5-6-13-30-22-11-7-10-21(29)23(22)20-14-18(17-9-8-12-27-16-17)19(15-25)24(26)28-20/h7,10-11,14,17,27,29H,2-6,8-9,12-13,16H2,1H3,(H2,26,28)/t17-/m1/s1 |
|  | Compound 4h | 60 | 0 | 0 | 0 | 1 | 0 | 0 | c1(C#N)c(N)nc(c2c(O)cccc2OCCCCCCC)cc1C3CCNCC3 | InChI=1/C24H32N4O2/c1-2-3-4-5-6-14-30-22-9-7-8-21(29)23(22)20-15-18(17-10-12-27-13-11-17)19(16-25)24(26)28-20/h7-9,15,17,27,29H,2-6,10-14H2,1H3,(H2,26,28) |
|  | Compound 4g | 60 | 0 | 0 | 0 | 1 | 0 | 0 | c1(C#N)c(N)nc(c2c(O)cccc2OCCCCCC)cc1C3CCNCC3 | InChI=1/C23H30N4O2/c1-2-3-4-5-13-29-21-8-6-7-20(28)22(21)19-14-17(16-9-11-26-12-10-16)18(15-24)23(25)27-19/h6-8,14,16,26,28H,2-5,9-13H2,1H3,(H2,25,27) |
|  | Compound 3k | 60 | 0 | 0 | 0 | 1 | 0 | 0 | c1(C#N)c(N)nc(c2c(OCCCCC)cccc2O)cc1[C@@H]3CCCNC3 | InChI=1/C22H28N4O2/c1-2-3-4-11-28-20-9-5-8-19(27)21(20)18-12-16(15-7-6-10-25-14-15)17(13-23)22(24)26-18/h5,8-9,12,15,25,27H,2-4,6-7,10-11,14H2,1H3,(H2,24,26)/t15-/m1/s1 |
|  | Compound 4f | 60 | 0 | 0 | 0 | 1 | 0 | 0 | c1(C#N)c(N)nc(c2c(O)cccc2OCCCCC)cc1C3CCNCC3 | InChI=1/C22H28N4O2/c1-2-3-4-12-28-20-7-5-6-19(27)21(20)18-13-16(15-8-10-25-11-9-15)17(14-23)22(24)26-18/h5-7,13,15,25,27H,2-4,8-12H2,1H3,(H2,24,26) |
|  | Compound 3q | 60 | 0 | 0 | 0 | 1 | 0 | 0 | c1(C#N)c(N)nc(c2c(OCC3CCCCC3)cccc2O)cc1[C@@H]4CCCNC4 | InChI=1/C24H30N4O2/c25-13-19-18(17-8-5-11-27-14-17)12-20(28-24(19)26)23-21(29)9-4-10-22(23)30-15-16-6-2-1-3-7-16/h4,9-10,12,16-17,27,29H,1-3,5-8,11,14-15H2,(H2,26,28)/t17-/m1/s1 |
|  | Erbstatin | 62 | 0 | 0 | 0 | 0 | 1 | 0 | c1(cc(O)ccc1O)\C=C\C(OC)=O | InChI=1/C10H10O4/c1-14-10(13)5-2-7-6-8(11)3-4-9(7)12/h2-6,11-12H,1H3/b5-2+ |
|  | Aspirin | 62 | 0 | 0 | 0 | 0 | 1 | 0 | CC(Oc1c(C(O)=O)cccc1)=O | InChI=1/C9H8O4/c1-6(10)13-8-5-3-2-4-7(8)9(11)12/h2-5H,1H3,(H,11,12) |
|  | 194 | 63 | 1 | 0 | 0 | 0 | 0 | 0 | [C@@H]1(\C(=C\C=C=CC[C@@H]([NH3+])CC)\C(=O)C=C1)C\C=C\CCCC(O)=O | InChI=1/C20H27NO3/c1-2-17(21)11-7-5-8-12-18-16(14-15-19(18)22)10-6-3-4-9-13-20(23)24/h3,6-8,12,14-17H,2,4,9-11,13,21H2,1H3,(H,23,24)/p+1/b6-3+,18-12-/t5?,16-,17+/m1/s1 |
|  | 189 | 63 | 1 | 0 | 0 | 0 | 0 | 0 | [C@@H]1(\C(=C/C=C/CC[C@@H](O)CC)\C(=O)C=C1)C\C=C\CCCC(O)=O | InChI=1/C20H28O4/c1-2-17(21)11-7-5-8-12-18-16(14-15-19(18)22)10-6-3-4-9-13-20(23)24/h3,5-6,8,12,14-17,21H,2,4,7,9-11,13H2,1H3,(H,23,24)/b6-3+,8-5+,18-12+/t16-,17+/m1/s1 |
|  | 190 | 63 | 1 | 0 | 0 | 0 | 0 | 0 | [C@H]1(C\C=C\CCCC(O)=O)\C(=C/C=C/CCC[C@@H](C(N)=O)C)\C(=O)C=C1 | InChI=1/C21H29NO4/c1-16(21(22)26)10-6-2-4-8-12-18-17(14-15-19(18)23)11-7-3-5-9-13-20(24)25/h3-4,7-8,12,14-17H,2,5-6,9-11,13H2,1H3,(H2,22,26)(H,24,25)/b7-3+,8-4+,18-12+/t16-,17+/m0/s1 |
|  | 193 | 63 | 1 | 0 | 0 | 0 | 0 | 0 | [C@H]1(C\C=C\CCCC(O)=O)\C(=C/C=C/CCCCCN)\C(=O)C=C1 | InChI=1/C20H29NO3/c21-16-10-6-2-1-3-8-12-18-17(14-15-19(18)22)11-7-4-5-9-13-20(23)24/h3-4,7-8,12,14-15,17H,1-2,5-6,9-11,13,16,21H2,(H,23,24)/b7-4+,8-3+,18-12+/t17-/m1/s1 |
|  | 199 | 63 | 1 | 0 | 0 | 0 | 0 | 0 | [C@@H]1(\C(=C\C=C=C[C@@H](C)[C@@H]([NH3+])CC)\C(=O)C=C1S)C\C=C\CCCC(O)=O | InChI=1/C21H29NO3S/c1-3-18(22)15(2)10-8-9-11-16-17(20(26)14-19(16)23)12-6-4-5-7-13-21(24)25/h4,6,9-11,14-15,17-18,26H,3,5,7,12-13,22H2,1-2H3,(H,24,25)/p+1/b6-4+,16-11-/t8?,15-,17+,18+/m1/s1 |
|  | 184 | 63 | 1 | 0 | 0 | 0 | 0 | 0 | [C@@H]1(\C(=C/C=C/CC[C@@H](F)CC)\C(=O)C=C1)C\C=C\CCCC(O)=O | InChI=1/C20H27FO3/c1-2-17(21)11-7-5-8-12-18-16(14-15-19(18)22)10-6-3-4-9-13-20(23)24/h3,5-6,8,12,14-17H,2,4,7,9-11,13H2,1H3,(H,23,24)/b6-3+,8-5+,18-12+/t16-,17+/m1/s1 |
|  | 182 | 63 | 1 | 0 | 0 | 0 | 0 | 0 | [C@@H]1(\C(=C/C=C/CC[C@@H](Cl)CC)\C(=O)C=C1)C\C=C\CCCC(O)=O | InChI=1/C20H27ClO3/c1-2-17(21)11-7-5-8-12-18-16(14-15-19(18)22)10-6-3-4-9-13-20(23)24/h3,5-6,8,12,14-17H,2,4,7,9-11,13H2,1H3,(H,23,24)/b6-3+,8-5+,18-12+/t16-,17+/m1/s1 |
|  | 192 | 63 | 1 | 0 | 0 | 0 | 0 | 0 | [C@@H]1(\C(=C/C=C/CCCCC)\C(=O)C=C1)C\C=C\CCCC(O)=O | InChI=1/C20H28O3/c1-2-3-4-5-6-10-13-18-17(15-16-19(18)21)12-9-7-8-11-14-20(22)23/h6-7,9-10,13,15-17H,2-5,8,11-12,14H2,1H3,(H,22,23)/b9-7+,10-6+,18-13+/t17-/m1/s1 |
|  | Parthenolide | 64 | 0 | 0 | 0 | 0 | 1 | 0 | C=C1[C@@H](CC\C(=C/CC[C@@]2([C@@H]3O2)C)\C)[C@@H]3OC1=O | InChI=1/C15H20O3/c1-9-5-4-8-15(3)13(18-15)12-11(7-6-9)10(2)14(16)17-12/h5,11-13H,2,4,6-8H2,1,3H3/b9-5-/t11-,12+,13-,15-/m1/s1 |
|  | Isohelenin | 64 | 0 | 0 | 0 | 0 | 1 | 0 | C=C1[C@H](C[C@@H](C(=C)C(=O)O2)[C@@H]2C3)[C@]3(C)CCC1 | InChI=1/C15H20O2/c1-9-5-4-6-15(3)8-13-11(7-12(9)15)10(2)14(16)17-13/h11-13H,1-2,4-8H2,3H3/t11-,12-,13-,15+/m0/s1 |
|  | Emodin | 70 | 0 | 0 | 0 | 0 | 1 | 0 | Cc1cc(C(=O)c(cc(O)cc2O)c2C3=O)c3c(O)c1 | InChI=1/C15H10O5/c1-6-2-8-12(10(17)3-6)15(20)13-9(14(8)19)4-7(16)5-11(13)18/h2-5,16-18H,1H3 |
|  | Baicalein | 70 | 0 | 0 | 0 | 0 | 0 | 1 | O=C1C=C(c2ccccc2)Oc(cc(O)c(O)c3O)c13 | InChI=1/C15H10O5/c16-9-6-11(8-4-2-1-3-5-8)20-12-7-10(17)14(18)15(19)13(9)12/h1-7,17-19H |
|  | hesperetin | 70 | 0 | 0 | 0 | 0 | 1 | 0 | O=C1C[C@@H](c2cc(O)c(OC)cc2)Oc(cc(cc3O)O)c13 | InChI=1/C16H14O6/c1-21-13-3-2-8(4-10(13)18)14-7-12(20)16-11(19)5-9(17)6-15(16)22-14/h2-6,14,17-19H,7H2,1H3/t14-/m0/s1 |
|  | 3,4,3',5'-tetrahydroxy-trans-stilbene | 70 | 0 | 0 | 0 | 0 | 1 | 0 | c1(cc(O)cc(O)c1)\C=C\c2cc(O)cc(O)c2 | InChI=1/C14H12O4/c15-11-3-9(4-12(16)7-11)1-2-10-5-13(17)8-14(18)6-10/h1-8,15-18H/b2-1+ |
|  | beta Carboline 14 | 72 | 0 | 0 | 0 | 1 | 0 | 0 | c12c(ccnc1)c(cc(Cl)c(OC(=O)N3CCOCC3)c4Cl)c4[nH]2 | InChI=1/C16H13Cl2N3O3/c17-11-7-10-9-1-2-19-8-12(9)20-14(10)13(18)15(11)24-16(22)21-3-5-23-6-4-21/h1-2,7-8,20H,3-6H2 |
|  | Anilino-pyrimidine | 72 | 0 | 0 | 0 | 1 | 0 | 0 | c1c(c2nc(Nc3ccc(C(=O)N4CCOCC4)cc3)ncc2)ccc(Cl)c1 | InChI=1/C21H19ClN4O2/c22-17-5-1-15(2-6-17)19-9-10-23-21(25-19)24-18-7-3-16(4-8-18)20(27)26-11-13-28-14-12-26/h1-10H,11-14H2,(H,23,24,25) |
|  | Sodium salicylate | 74 | 0 | 0 | 0 | 0 | 1 | 0 | [Na+].O=C(c1c(O)cccc1)[O-] | InChI=1/C7H6O3.Na/c8-6-4-2-1-3-5(6)7(9)10;/h1-4,8H,(H,9,10);/q;+1/p-1 |
|  | nicotine | 74 | 0 | 0 | 0 | 0 | 1 | 0 | n1cccc([C@@H]2CCCN2C)c1 | InChI=1/C10H14N2/c1-12-7-3-5-10(12)9-4-2-6-11-8-9/h2,4,6,8,10H,3,5,7H2,1H3/t10-/m0/s1 |
|  | thiophenecarboxamide\_baxter\_2 | 74 | 0 | 0 | 0 | 1 | 0 | 0 | NC(Nc1c(C(=O)N)scc1)=O | InChI=1/C6H7N3O2S/c7-5(10)4-3(1-2-12-4)9-6(8)11/h1-2H,(H2,7,10)(H3,8,9,11) |
|  | CAPE | 76 | 1 | 0 | 0 | 0 | 0 | 0 | Oc1c(O)cc(\C=C\C(=O)OCCc2ccccc2)cc1 | InChI=1/C17H16O4/c18-15-8-6-14(12-16(15)19)7-9-17(20)21-11-10-13-4-2-1-3-5-13/h1-9,12,18-19H,10-11H2/b9-7+ |
|  | CAPE analogue 1 | 76 | 1 | 0 | 0 | 0 | 0 | 0 | c1cc(O)c(\C=C\C(=O)OCCc2ccccc2)cc1O | InChI=1/C17H16O4/c18-15-7-8-16(19)14(12-15)6-9-17(20)21-11-10-13-4-2-1-3-5-13/h1-9,12,18-19H,10-11H2/b9-6+ |
|  | TP301 | 81 | 0 | 0 | 0 | 1 | 0 | 0 | [C@@]12([C@H](CC[C@@H]3C1=CC(=O)[C@H]([C@H](CC(C)(C)C[C@H]4C(=O)NCCCCCCCNC(=O)CCCC[C@@H]5S[C@H](NC(=O)N6)[C@H]6C5)[C@H]4CC7)[C@H]37)C(C)(C)C(=O)C(C#N)=C2)C | InChI=1/C46H67N5O5S/c1-44(2)24-32-29(15-16-31-30-17-18-37-45(3,4)40(54)27(26-47)23-46(37,5)34(30)22-36(52)39(31)32)33(25-44)41(55)49-20-12-8-6-7-11-19-48-38(53)14-10-9-13-28-21-35-42(57-28)51-43(56)50-35/h22-23,28-33,35,37,39,42H,6-21,24-25H2,1-5H3,(H,48,53)(H,49,55)(H2,50,51,56)/t28-,29+,30-,31-,32+,33+,35+,37+,39-,42-,46+/m0/s1 |
|  | TP304 | 81 | 0 | 0 | 0 | 1 | 0 | 0 | [C@@]12([C@H](CC[C@@H]3C1=CC(=O)[C@H]([C@H](CC(C)(C)C[C@H]4C(=O)OC)[C@H]4CC5)[C@H]35)[C@](C)(COC(=O)CCCCCNC(=O)CCCC[C@@H]6S[C@H](NC(=O)N7)[C@H]7C6)C(=O)C(C#N)=C2)C | InChI=1/C46H64N4O8S/c1-44(2)22-31-28(32(23-44)42(55)57-5)14-15-30-29-16-17-36-45(3,33(29)20-35(51)39(30)31)21-26(24-47)40(54)46(36,4)25-58-38(53)13-7-6-10-18-48-37(52)12-9-8-11-27-19-34-41(59-27)50-43(56)49-34/h20-21,27-32,34,36,39,41H,6-19,22-23,25H2,1-5H3,(H,48,52)(H2,49,50,56)/t27-,28+,29-,30-,31+,32+,34+,36-,39-,41-,45+,46-/m0/s1 |
|  | N-alpha-p-Tosyl-L-lysine chloromethyl ketone | 84 | 0 | 0 | 0 | 0 | 1 | 0 | O=S(c1ccc(C)cc1)(N[C@H](C(=O)O)CCCCN)=O | InChI=1/C13H20N2O4S/c1-10-5-7-11(8-6-10)20(18,19)15-12(13(16)17)4-2-3-9-14/h5-8,12,15H,2-4,9,14H2,1H3,(H,16,17)/t12-/m0/s1 |
|  | L-buthionine-(S,R)-sulfoximine | 84 | 0 | 0 | 0 | 0 | 1 | 0 | CCCC[S@](CC[C@H](C(O)=O)N)(=O)=N | InChI=1/C8H18N2O3S/c1-2-3-5-14(10,13)6-4-7(9)8(11)12/h7,10H,2-6,9H2,1H3,(H,11,12)/t7-,14-/m1/s1 |
|  | BAY 11-7085 | 86 | 0 | 0 | 0 | 0 | 1 | 0 | c1(ccc(S(\C=C/C#N)(=O)=O)cc1)C(C)(C)C | InChI=1/C13H15NO2S/c1-13(2,3)11-5-7-12(8-6-11)17(15,16)10-4-9-14/h4-8,10H,1-3H3/b10-4- |
|  | BAY 11-7082 | 86 | 0 | 0 | 0 | 0 | 1 | 0 | c1(ccc(S(\C=C/C#N)(=O)=O)cc1)C | InChI=1/C10H9NO2S/c1-9-3-5-10(6-4-9)14(12,13)8-2-7-11/h2-6,8H,1H3/b8-2- |
|  | SC-514 | 91 | 0 | 0 | 0 | 1 | 0 | 0 | s1c(C(N)=O)c(cc1c(c2)ccs2)N | InChI=1/C9H8N2OS2/c10-6-3-7(5-1-2-13-4-5)14-8(6)9(11)12/h1-4H,10H2,(H2,11,12) |
|  | thiophenecarboxamide\_baxter\_1 | 91 | 0 | 0 | 0 | 1 | 0 | 0 | NC(c1c(N)cc(c2cscc2)s1)=O | InChI=1/C9H8N2OS2/c10-6-3-7(5-1-2-13-4-5)14-8(6)9(11)12/h1-4H,10H2,(H2,11,12) |
|  | thiophenecarboxamide\_baxter\_5 | 91 | 0 | 0 | 0 | 1 | 0 | 0 | NC(c1c(N)cc(c2ccccc2)s1)=O | InChI=1/C11H10N2OS/c12-8-6-9(15-10(8)11(13)14)7-4-2-1-3-5-7/h1-6H,12H2,(H2,13,14) |
|  | thiophenecarboxamide\_baxter\_3 | 91 | 0 | 0 | 0 | 1 | 0 | 0 | NC(Nc1c(C(=O)N)sc(c2cscc2)c1)=O | InChI=1/C10H9N3O2S2/c11-9(14)8-6(13-10(12)15)3-7(17-8)5-1-2-16-4-5/h1-4H,(H2,11,14)(H3,12,13,15) |
|  | 200 | 96 | 1 | 0 | 0 | 0 | 0 | 0 | [C@H]1(C\C=C\CCCC(O)=O)\C(=C(\C)/C=C/CCCCCC(O)=O)\C(=O)C=C1[S-] | InChI=1/C22H30O5S/c1-16(11-7-3-2-4-9-13-20(24)25)22-17(19(28)15-18(22)23)12-8-5-6-10-14-21(26)27/h5,7-8,11,15,17,28H,2-4,6,9-10,12-14H2,1H3,(H,24,25)(H,26,27)/p-1/b8-5+,11-7+,22-16+/t17-/m1/s1 |
|  | 203 | 96 | 1 | 0 | 0 | 0 | 0 | 0 | [C@H]1(C\C=C\CCCC(O)=O)\C(=C/C=C/CCCCCC(O)=O)\C(=O)C=C1[S-] | InChI=1/C21H28O5S/c22-18-15-19(27)17(12-8-5-6-10-14-21(25)26)16(18)11-7-3-1-2-4-9-13-20(23)24/h3,5,7-8,11,15,17,27H,1-2,4,6,9-10,12-14H2,(H,23,24)(H,25,26)/p-1/b7-3+,8-5+,16-11+/t17-/m0/s1 |
|  | 188 | 96 | 1 | 0 | 0 | 0 | 0 | 0 | [C@H]1(C\C=C\CCCC(O)=O)\C(=C(\C)/C=C/CCCC[C@@](C)(CCC(O)=O)O)\C(=O)C=C1 | InChI=1/C25H36O6/c1-19(11-7-5-6-10-17-25(2,31)18-16-23(29)30)24-20(14-15-21(24)26)12-8-3-4-9-13-22(27)28/h3,7-8,11,14-15,20,31H,4-6,9-10,12-13,16-18H2,1-2H3,(H,27,28)(H,29,30)/b8-3+,11-7+,24-19+/t20-,25+/m1/s1 |
|  | 185 | 96 | 1 | 0 | 0 | 0 | 0 | 0 | [C@H]1(C\C=C\CCCC(O)=O)\C(=C\C=C\CCCCC)\C(=O)C=C1C(O)=O | InChI=1/C21H28O5/c1-2-3-4-5-6-10-13-17-16(18(21(25)26)15-19(17)22)12-9-7-8-11-14-20(23)24/h6-7,9-10,13,15-16H,2-5,8,11-12,14H2,1H3,(H,23,24)(H,25,26)/b9-7+,10-6+,17-13-/t16-/m0/s1 |
|  | 187 | 96 | 1 | 0 | 0 | 0 | 0 | 0 | [C@H]1(C\C=C\CCCC(O)=O)\C(=C(\C)/C=C/CCCC[C@@H](C)CCC(O)=O)\C(=O)C=C1 | InChI=1/C25H36O5/c1-19(15-18-24(29)30)11-7-3-4-8-12-20(2)25-21(16-17-22(25)26)13-9-5-6-10-14-23(27)28/h5,8-9,12,16-17,19,21H,3-4,6-7,10-11,13-15,18H2,1-2H3,(H,27,28)(H,29,30)/b9-5+,12-8+,25-20+/t19-,21-/m1/s1 |
|  | 202 | 96 | 1 | 0 | 0 | 0 | 0 | 0 | [C@H]1(C\C=C\CCCC(O)=O)\C(=C\C=C\CCCCC)\C(=O)C=C1[S-] | InChI=1/C20H28O3S/c1-2-3-4-5-6-9-12-16-17(19(24)15-18(16)21)13-10-7-8-11-14-20(22)23/h6-7,9-10,12,15,17,24H,2-5,8,11,13-14H2,1H3,(H,22,23)/p-1/b9-6+,10-7+,16-12-/t17-/m0/s1 |
|  | 183 | 96 | 1 | 0 | 0 | 0 | 0 | 0 | [C@H]1(C\C=C\CCCC(O)=O)\C(=C/C=C/CCC[C@@H](C)F)\C(=O)C=C1O | InChI=1/C20H27FO4/c1-15(21)10-6-2-3-7-11-16-17(19(23)14-18(16)22)12-8-4-5-9-13-20(24)25/h3-4,7-8,11,14-15,17,23H,2,5-6,9-10,12-13H2,1H3,(H,24,25)/b7-3+,8-4+,16-11+/t15-,17+/m1/s1 |
|  | 198 | 96 | 1 | 0 | 0 | 0 | 0 | 0 | [C@H]1(C\C=C\CCCC(O)=O)\C(=C\C=C\CCCCC)\C(=O)C=C1S | InChI=1/C20H28O3S/c1-2-3-4-5-6-9-12-16-17(19(24)15-18(16)21)13-10-7-8-11-14-20(22)23/h6-7,9-10,12,15,17,24H,2-5,8,11,13-14H2,1H3,(H,22,23)/b9-6+,10-7+,16-12-/t17-/m0/s1 |
|  | 196 | 96 | 1 | 0 | 0 | 0 | 0 | 0 | [C@H]1(C\C=C\CCCC(O)=O)\C(=C/C=C/CCC[C@@H](C)F)\C(=O)C=C1S | InChI=1/C20H27FO3S/c1-15(21)10-6-2-3-7-11-16-17(19(25)14-18(16)22)12-8-4-5-9-13-20(23)24/h3-4,7-8,11,14-15,17,25H,2,5-6,9-10,12-13H2,1H3,(H,23,24)/b7-3+,8-4+,16-11+/t15-,17+/m1/s1 |
|  | 181 | 96 | 1 | 0 | 0 | 0 | 0 | 0 | [C@H]1(C\C=C\CCCC(O)=O)\C(=C\C=C\CCCCC)\C(=O)C=C1Br | InChI=1/C20H27BrO3/c1-2-3-4-5-6-10-13-17-16(18(21)15-19(17)22)12-9-7-8-11-14-20(23)24/h6-7,9-10,13,15-16H,2-5,8,11-12,14H2,1H3,(H,23,24)/b9-7+,10-6+,17-13-/t16-/m0/s1 |
|  | 197 | 96 | 1 | 0 | 0 | 0 | 0 | 0 | [C@H]1(C\C=C\CCCC(O)=O)\C(=C/C=C/CCC[C@@H](C)O)\C(=O)C=C1S | InChI=1/C20H28O4S/c1-15(21)10-6-2-3-7-11-16-17(19(25)14-18(16)22)12-8-4-5-9-13-20(23)24/h3-4,7-8,11,14-15,17,21,25H,2,5-6,9-10,12-13H2,1H3,(H,23,24)/b7-3+,8-4+,16-11+/t15-,17+/m1/s1 |
|  | 195 | 96 | 1 | 0 | 0 | 0 | 0 | 0 | [C@H]1(C\C=C\CCCC(O)=O)\C(=C/C=C/CCC[C@@H](C)O)\C(=O)C=C1O | InChI=1/C20H28O5/c1-15(21)10-6-2-3-7-11-16-17(19(23)14-18(16)22)12-8-4-5-9-13-20(24)25/h3-4,7-8,11,14-15,17,21,23H,2,5-6,9-10,12-13H2,1H3,(H,24,25)/b7-3+,8-4+,16-11+/t15-,17+/m1/s1 |
|  | 191 | 96 | 1 | 0 | 0 | 0 | 0 | 0 | [C@H]1(C\C=C\CCCC(O)=O)\C(=C/C=C/CCC[C@@H]([N+]([O-])=O)C)\C(=O)C=C1 | InChI=1/C20H27NO5/c1-16(21(25)26)10-6-2-4-8-12-18-17(14-15-19(18)22)11-7-3-5-9-13-20(23)24/h3-4,7-8,12,14-17H,2,5-6,9-11,13H2,1H3,(H,23,24)/b7-3+,8-4+,18-12+/t16-,17+/m0/s1 |
|  | 1-hydroxy-1-hydroperoxynonane | 97 | 1 | 0 | 0 | 0 | 0 | 0 | CCCCCCCC[C@H](OO)O | InChI=1/C9H20O3/c1-2-3-4-5-6-7-8-9(10)12-11/h9-11H,2-8H2,1H3/t9-/m0/s1 |
|  | 4-hydroxynonenal | 97 | 0 | 0 | 0 | 0 | 1 | 0 | CCCCC[C@H](\C=C\C=O)O | InChI=1/C9H16O2/c1-2-3-4-6-9(11)7-5-8-10/h5,7-9,11H,2-4,6H2,1H3/b7-5+/t9-/m1/s1 |
|  | N-acetyl-L-cysteine (NAC) | 99 | 0 | 0 | 0 | 0 | 0 | 1 | O=C([C@H](CS)NC(C)=O)O | InChI=1/C5H9NO3S/c1-3(7)6-4(2-10)5(8)9/h4,10H,2H2,1H3,(H,6,7)(H,8,9)/t4-/m0/s1 |
|  | S-ALLYL CYSTEINE | 99 | 0 | 1 | 0 | 0 | 0 | 0 | O=C([C@H](CS)N)O | InChI=1/C3H7NO2S/c4-2(1-7)3(5)6/h2,7H,1,4H2,(H,5,6)/t2-/m0/s1 |
|  | beta Carboline 20 | 100 | 0 | 0 | 0 | 1 | 0 | 0 | c12c(ccnc1)c(cc(Cl)cc3N(C)c4ccccc4)c3[nH]2 | InChI=1/C18H14ClN3/c1-22(13-5-3-2-4-6-13)17-10-12(19)9-15-14-7-8-20-11-16(14)21-18(15)17/h2-11,21H,1H3 |
|  | beta Carboline 23 | 100 | 0 | 0 | 0 | 1 | 0 | 0 | c12c(ccnc1)c(cc(Cl)cc3NC(=O)c4ccccc4)c3[nH]2 | InChI=1/C18H12ClN3O/c19-12-8-14-13-6-7-20-10-16(13)21-17(14)15(9-12)22-18(23)11-4-2-1-3-5-11/h1-10,21H,(H,22,23) |
|  | beta Carboline 32 | 100 | 0 | 0 | 0 | 1 | 0 | 0 | c12c(ccnc1)c(cc(Cl)cc3NS(=O)(=O)c4ccccc4)c3[nH]2 | InChI=1/C17H12ClN3O2S/c18-11-8-14-13-6-7-19-10-16(13)20-17(14)15(9-11)21-24(22,23)12-4-2-1-3-5-12/h1-10,20-21H |
|  | beta Carboline 28 | 100 | 0 | 0 | 0 | 1 | 0 | 0 | c12c(ccnc1)c(cc(Cl)cc3NC(=O)c4c(OC)cccc4)c3[nH]2 | InChI=1/C19H14ClN3O2/c1-25-17-5-3-2-4-13(17)19(24)23-15-9-11(20)8-14-12-6-7-21-10-16(12)22-18(14)15/h2-10,22H,1H3,(H,23,24) |
|  | beta Carboline 29 | 100 | 0 | 0 | 0 | 1 | 0 | 0 | c12c(ccnc1)c(cc(Cl)cc3NC(=O)c4cc(OC)ccc4)c3[nH]2 | InChI=1/C19H14ClN3O2/c1-25-13-4-2-3-11(7-13)19(24)23-16-9-12(20)8-15-14-5-6-21-10-17(14)22-18(15)16/h2-10,22H,1H3,(H,23,24) |
|  | beta-Carboline 13 | 100 | 0 | 0 | 0 | 1 | 0 | 0 | c12c(ccnc1)c(cc(Cl)c(OCC3CCCCC3)c4Cl)c4[nH]2 | InChI=1/C18H18Cl2N2O/c19-14-8-13-12-6-7-21-9-15(12)22-17(13)16(20)18(14)23-10-11-4-2-1-3-5-11/h6-9,11,22H,1-5,10H2 |
|  | beta Carboline 25 | 100 | 0 | 0 | 0 | 1 | 0 | 0 | c12c(ccnc1)c(cc(Cl)cc3NC(=O)c4ncccc4)c3[nH]2 | InChI=1/C17H11ClN4O/c18-10-7-12-11-4-6-19-9-15(11)21-16(12)14(8-10)22-17(23)13-3-1-2-5-20-13/h1-9,21H,(H,22,23) |
|  | beta Carboline 24 | 100 | 0 | 0 | 0 | 1 | 0 | 0 | c12c(ccnc1)c(cc(Cl)cc3NC(=O)Cc4ccccn4)c3[nH]2 | InChI=1/C18H13ClN4O/c19-11-7-14-13-4-6-20-10-16(13)23-18(14)15(8-11)22-17(24)9-12-3-1-2-5-21-12/h1-8,10,23H,9H2,(H,22,24) |
|  | beta-Carboline 11 | 100 | 0 | 0 | 0 | 1 | 0 | 0 | c12c(ccnc1)c(cc(Cl)c(OCC)c3Cl)c3[nH]2 | InChI=1/C13H10Cl2N2O/c1-2-18-13-9(14)5-8-7-3-4-16-6-10(7)17-12(8)11(13)15/h3-6,17H,2H2,1H3 |
|  | o-phenanthroline | 106 | 1 | 0 | 0 | 0 | 0 | 0 | n1cccc(ccc2c3nccc2)c13 | InChI=1/C12H8N2/c1-3-9-5-6-10-4-2-8-14-12(10)11(9)13-7-1/h1-8H |
|  | 9AA | 106 | 1 | 0 | 0 | 0 | 0 | 0 | c12c(nc3c(cccc3)c1N)cccc2 | InChI=1/C13H10N2/c14-13-9-5-1-3-7-11(9)15-12-8-4-2-6-10(12)13/h1-8H,(H2,14,15) |
|  | glutathione | 107 | 0 | 0 | 0 | 0 | 1 | 0 | C([C@H](N)C(O)=O)CC(N[C@H](C(NCC(O)=O)=O)CSSC[C@H](NC(CC[C@H](N)C(O)=O)=O)C(NCC(O)=O)=O)=O | InChI=1/C20H32N6O12S2/c21-9(19(35)36)1-3-13(27)25-11(17(33)23-5-15(29)30)7-39-40-8-12(18(34)24-6-16(31)32)26-14(28)4-2-10(22)20(37)38/h9-12H,1-8,21-22H2,(H,23,33)(H,24,34)(H,25,27)(H,26,28)(H,29,30)(H,31,32)(H,35,36)(H,37,38)/t9-,10-,11-,12-/m0/s1 |
|  | S-nitrosogluthathione | 107 | 0 | 0 | 0 | 0 | 1 | 0 | S(C[C@H](NC(CC[C@H](N)C(O)=O)=O)C(NCC(O)=O)=O)N=O | InChI=1/C10H16N4O7S/c11-5(10(19)20)1-2-7(15)13-6(4-22-14-21)9(18)12-3-8(16)17/h5-6H,1-4,11H2,(H,12,18)(H,13,15)(H,16,17)(H,19,20)/t5-,6-/m0/s1 |
|  | phenethyl isothiocyanate | 110 | 0 | 0 | 0 | 0 | 1 | 0 | S=C=NCCc1ccccc1 | InChI=1/C9H9NS/c11-8-10-7-6-9-4-2-1-3-5-9/h1-5H,6-7H2 |
|  | Methaamphetamine | 110 | 0 | 1 | 0 | 0 | 0 | 0 | c1(ccccc1)C[C@H](C)NC | InChI=1/C10H15N/c1-9(11-2)8-10-6-4-3-5-7-10/h3-7,9,11H,8H2,1-2H3/t9-/m0/s1 |
|  | Doxorubicin | 112 | 1 | 0 | 0 | 0 | 0 | 0 | COc1c(C(=O)c(c(O)c([C@@H](O[C@H]2O[C@@H](C)[C@@H](O)[C@@H](N)C2)C[C@@](C(CO)=O)(O)C3)c3c4O)c4C5=O)c5ccc1 | InChI=1/C27H29NO11/c1-10-22(31)13(28)6-17(38-10)39-15-8-27(36,16(30)9-29)7-12-19(15)26(35)21-20(24(12)33)23(32)11-4-3-5-14(37-2)18(11)25(21)34/h3-5,10,13,15,17,22,29,31,33,35-36H,6-9,28H2,1-2H3/t10-,13-,15-,17+,22+,27-/m0/s1 |
|  | Polipodine A (unknow steroechemistry on 2-OHs of on 6 membered ring) | 112 | 0 | 0 | 0 | 0 | 1 | 0 | CC(CC[C@H]([C@@](C1[C@@](C)(CC[C@H]([C@@](C)(C[C@H](O)[C@H](O)C2)[C@@H]2C3=O)C4=C3)[C@@]4(O)CC1)(O)C)O)(O)C | InChI=1/C27H44O7/c1-23(2,32)9-8-22(31)26(5,33)21-7-11-27(34)16-12-18(28)17-13-19(29)20(30)14-24(17,3)15(16)6-10-25(21,27)4/h12,15,17,19-22,29-34H,6-11,13-14H2,1-5H3/t15-,17-,19+,20-,21?,22+,24+,25+,26+,27+/m0/s1 |
|  | Compound 3b | 114 | 0 | 0 | 0 | 1 | 0 | 0 | c1(C#N)c(N)nc(c2cccc(C)c2O)cc1[C@@H]3CCCNC3 | InChI=1/C18H20N4O/c1-11-4-2-6-13(17(11)23)16-8-14(12-5-3-7-21-10-12)15(9-19)18(20)22-16/h2,4,6,8,12,21,23H,3,5,7,10H2,1H3,(H2,20,22)/t12-/m1/s1 |
|  | Compound 3e | 114 | 0 | 0 | 0 | 1 | 0 | 0 | c1(C#N)c(N)nc(c2c(C)cccc2O)cc1[C@@H]3CCCNC3 | InChI=1/C18H20N4O/c1-11-4-2-6-16(23)17(11)15-8-13(12-5-3-7-21-10-12)14(9-19)18(20)22-15/h2,4,6,8,12,21,23H,3,5,7,10H2,1H3,(H2,20,22)/t12-/m1/s1 |
|  | Compound 3a | 114 | 0 | 0 | 0 | 1 | 0 | 0 | c1(C#N)c(N)nc(c2ccccc2O)cc1[C@@H]3CCCNC3 | InChI=1/C17H18N4O/c18-9-14-13(11-4-3-7-20-10-11)8-15(21-17(14)19)12-5-1-2-6-16(12)22/h1-2,5-6,8,11,20,22H,3-4,7,10H2,(H2,19,21)/t11-/m1/s1 |
|  | Compound 4a | 114 | 0 | 0 | 0 | 1 | 0 | 0 | c1(C#N)c(N)nc(c2c(O)cccc2)cc1C3CCNCC3 | InChI=1/C17H18N4O/c18-10-14-13(11-5-7-20-8-6-11)9-15(21-17(14)19)12-3-1-2-4-16(12)22/h1-4,9,11,20,22H,5-8H2,(H2,19,21) |
|  | Compound 3c | 114 | 0 | 0 | 0 | 1 | 0 | 0 | c1(C#N)c(N)nc(c2cccc(OC)c2O)cc1[C@@H]3CCCNC3 | InChI=1/C18H20N4O2/c1-24-16-6-2-5-12(17(16)23)15-8-13(11-4-3-7-21-10-11)14(9-19)18(20)22-15/h2,5-6,8,11,21,23H,3-4,7,10H2,1H3,(H2,20,22)/t11-/m1/s1 |
|  | Compound 3d | 114 | 0 | 0 | 0 | 1 | 0 | 0 | c1(C#N)c(N)nc(c2ccc(OC)cc2O)cc1[C@@H]3CCCNC3 | InChI=1/C18H20N4O2/c1-24-12-4-5-13(17(23)7-12)16-8-14(11-3-2-6-21-10-11)15(9-19)18(20)22-16/h4-5,7-8,11,21,23H,2-3,6,10H2,1H3,(H2,20,22)/t11-/m1/s1 |
|  | Compound 3g | 114 | 0 | 0 | 0 | 1 | 0 | 0 | c1(C#N)c(N)nc(c2c(O)cccc2O)cc1[C@@H]3CCCNC3 | InChI=1/C17H18N4O2/c18-8-12-11(10-3-2-6-20-9-10)7-13(21-17(12)19)16-14(22)4-1-5-15(16)23/h1,4-5,7,10,20,22-23H,2-3,6,9H2,(H2,19,21)/t10-/m1/s1 |
|  | Deoxyspergualin | 119 | 0 | 0 | 0 | 0 | 0 | 1 | O=C([C@H](NC(CCCCCC\N=C(\N)/N)=O)O)NCCCCNCCCN | InChI=1/C17H37N7O3/c18-9-7-11-21-10-5-6-12-22-15(26)16(27)24-14(25)8-3-1-2-4-13-23-17(19)20/h16,21,27H,1-13,18H2,(H,22,26)(H,24,25)(H4,19,20,23)/t16-/m1/s1 |
|  | Glucosamine sulphate | 119 | 0 | 0 | 0 | 0 | 1 | 0 | O[C@@H]1[C@H](N)[C@H](O)O[C@H](CO)[C@H]1O.OS(=O)(=O)O | InChI=1/C6H13NO5.H2O4S/c7-3-5(10)4(9)2(1-8)12-6(3)11;1-5(2,3)4/h2-6,8-11H,1,7H2;(H2,1,2,3,4)/t2-,3+,4-,5-,6-;/m1./s1 |
|  | S-ibuprofen | 121 | 1 | 0 | 0 | 0 | 0 | 0 | CC(Cc1ccc([C@@H](C(=O)O)C)cc1)C | InChI=1/C13H18O2/c1-9(2)8-11-4-6-12(7-5-11)10(3)13(14)15/h4-7,9-10H,8H2,1-3H3,(H,14,15)/t10-/m0/s1 |
|  | R-ibuprofen | 121 | 0 | 0 | 0 | 0 | 0 | 1 | CC(Cc1ccc([C@H](C(=O)O)C)cc1)C | InChI=1/C13H18O2/c1-9(2)8-11-4-6-12(7-5-11)10(3)13(14)15/h4-7,9-10H,8H2,1-3H3,(H,14,15)/t10-/m1/s1 |
|  | Compound 1 | 122 | 0 | 0 | 0 | 1 | 0 | 0 | c1(ccccc1O)c2cc(c3cccc(NC(=O)[C@@H]4CCC(=O)O4)c3)c(C#N)c(N)n2 | InChI=1/C23H18N4O4/c24-12-17-16(11-18(27-22(17)25)15-6-1-2-7-19(15)28)13-4-3-5-14(10-13)26-23(30)20-8-9-21(29)31-20/h1-7,10-11,20,28H,8-9H2,(H2,25,27)(H,26,30)/t20-/m0/s1 |
|  | 3-Cyano-2-Aminopyridines | 122 | 0 | 0 | 0 | 1 | 0 | 0 | c1(ccccc1O)c2cc(c3cccc(NC(=O)CN4CCCCC4)c3)c(C#N)c(N)n2 | InChI=1/C25H25N5O2/c26-15-21-20(14-22(29-25(21)27)19-9-2-3-10-23(19)31)17-7-6-8-18(13-17)28-24(32)16-30-11-4-1-5-12-30/h2-3,6-10,13-14,31H,1,4-5,11-12,16H2,(H2,27,29)(H,28,32) |
|  | Compound 16 | 122 | 0 | 0 | 0 | 1 | 0 | 0 | c1(ccccc1O)c2cc(c3cccc(NC(=O)CN4CCCCC4)c3)c(C#N)c(N)n2 | InChI=1/C25H25N5O2/c26-15-21-20(14-22(29-25(21)27)19-9-2-3-10-23(19)31)17-7-6-8-18(13-17)28-24(32)16-30-11-4-1-5-12-30/h2-3,6-10,13-14,31H,1,4-5,11-12,16H2,(H2,27,29)(H,28,32) |
|  | Compound 21 | 122 | 0 | 0 | 0 | 1 | 0 | 0 | c1(ccccc1O)c2cc(c3cccc(NC(=O)COC)c3)c(C#N)c(N)n2 | InChI=1/C21H18N4O3/c1-28-12-20(27)24-14-6-4-5-13(9-14)16-10-18(25-21(23)17(16)11-22)15-7-2-3-8-19(15)26/h2-10,26H,12H2,1H3,(H2,23,25)(H,24,27) |
|  | Compound 23 | 122 | 0 | 0 | 0 | 1 | 0 | 0 | c1(ccccc1O)c2cc(c3cccc(NC(=O)CCCC([O-])=O)c3)c(C#N)c(N)n2.[Na] | InChI=1/C23H20N4O4.Na.H/c24-13-18-17(12-19(27-23(18)25)16-7-1-2-8-20(16)28)14-5-3-6-15(11-14)26-21(29)9-4-10-22(30)31;;/h1-3,5-8,11-12,28H,4,9-10H2,(H2,25,27)(H,26,29)(H,30,31);;/p-1 |
|  | Compound 22 | 122 | 0 | 0 | 0 | 1 | 0 | 0 | c1(ccccc1O)c2cc(c3ccc(NC(=O)CCCC([O-])=O)cc3)c(C#N)c(N)n2.[Na] | InChI=1/C23H20N4O4.Na.H/c24-13-18-17(12-19(27-23(18)25)16-4-1-2-5-20(16)28)14-8-10-15(11-9-14)26-21(29)6-3-7-22(30)31;;/h1-2,4-5,8-12,28H,3,6-7H2,(H2,25,27)(H,26,29)(H,30,31);;/p-1 |
|  | Compound 3r | 122 | 0 | 0 | 0 | 1 | 0 | 0 | c1(C#N)c(N)nc(c2c(OCc3ccccc3)cccc2O)cc1[C@@H]4CCCNC4 | InChI=1/C24H24N4O2/c25-13-19-18(17-8-5-11-27-14-17)12-20(28-24(19)26)23-21(29)9-4-10-22(23)30-15-16-6-2-1-3-7-16/h1-4,6-7,9-10,12,17,27,29H,5,8,11,14-15H2,(H2,26,28)/t17-/m1/s1 |
|  | 5,7-dihydroxy-4-methylcoumarin | 130 | 1 | 0 | 0 | 0 | 0 | 0 | c12c(C(=CC(O1)=O)C)c(O)cc(O)c2 | InChI=1/C10H8O4/c1-5-2-9(13)14-8-4-6(11)3-7(12)10(5)8/h2-4,11-12H,1H3 |
|  | Phenobarbital | 130 | 0 | 1 | 0 | 0 | 0 | 0 | O=C1C(CC)(c2ccccc2)C(=O)NC(=O)N1 | InChI=1/C12H12N2O3/c1-2-12(8-6-4-3-5-7-8)9(15)13-11(17)14-10(12)16/h3-7H,2H2,1H3,(H2,13,14,15,16,17) |
