# Supplementary material for: Predicting the points of interaction of small molecules in the NF-κB pathway
Source: BMC Syst Biol. 2011 Feb 22;5:32. doi: 10.1186/1752-0509-5-32 (PMC3050742; doi:10.1186/1752-0509-5-32)
Supplement: Additional file 3 — Clusters of Compounds Shown in Figure 6. [file 1752-0509-5-32-S3.ZIP › Additional Files 3/Clustering_including_compounds_with_unknown_interactions.htm]

|  |  |  |  |  |  |  |  |  |  |  |
| --- | --- | --- | --- | --- | --- | --- | --- | --- | --- | --- |
| Molecule | Name | Cluster | DNA\_interaction | ROS\_interaction | activates\_IkB\_phosphorylation\_degradation | inhibits\_IKK\_activation | inhibits\_IkB\_degradation\_phosphorylation | inhibits\_translocation | Smiles | InChI |
|  | ginsenoside Rg3 | 1 | 0 | 0 | 0 | 0 | 0 | 0 | C\C(=C\CC[C@@]([C@H]1C([C@@H](C[C@@H]2[C@@]3(C)CC[C@@H](C(C)(C)[C@@H](O[C@@H]4[C@H](O)[C@@H](O)[C@@H](O[C@@H]5[C@H](CO)O[C@@H](O)[C@@H](O)[C@H]5O)O[C@H]4CO)CC6)[C@@]26C)O)[C@@]3(C)CC1)(O)C)\C | InChI=1/C42H72O13/c1-21(2)10-9-14-42(8,51)22-11-16-41(7)29(22)23(45)18-27-39(5)15-13-28(38(3,4)26(39)12-17-40(27,41)6)54-34-25(20-44)53-37(33(49)31(34)47)55-35-24(19-43)52-36(50)32(48)30(35)46/h10,22-37,43-51H,9,11-20H2,1-8H3/t22-,23-,24+,25+,26+,27+,28+,29?,30-,31-,32+,33-,34+,35-,36-,37-,39+,40-,41-,42+/m1/s1 |
|  | Glycyrrhizin | 1 | 0 | 0 | 0 | 0 | 0 | 0 | O=C([C@@H]1[C@H](O)[C@H](O)[C@@H](O)[C@H](O[C@H]2[C@H](O[C@H]3CC[C@](C)([C@H](C(=O)C=C([C@H]4[C@](CC5)(C)CC[C@](C)(C(O)=O)C4)[C@@]56C)[C@@]6(C)CC7)[C@@H]7C3(C)C)O[C@H](C(O)=O)[C@@H](O)[C@@H]2O)O1)O | InChI=1/C42H62O16/c1-37(2)21-8-11-42(7)31(20(43)16-18-19-17-39(4,36(53)54)13-12-38(19,3)14-15-41(18,42)6)40(21,5)10-9-22(37)55-35-30(26(47)25(46)29(57-35)33(51)52)58-34-27(48)23(44)24(45)28(56-34)32(49)50/h16,19,21-31,34-35,44-48H,8-15,17H2,1-7H3,(H,49,50)(H,51,52)(H,53,54)/t19-,21-,22-,23-,24+,25-,26-,27+,28-,29-,30+,31-,34-,35+,38+,39-,40-,41+,42+/m0/s1 |
|  | Asiaticoside | 1 | 0 | 0 | 0 | 0 | 0 | 0 | C[C@H]1[C@@H](C)[C@H](C(=CC[C@@H]2[C@@]3(C)CC[C@H]([C@@](CO)(C)[C@@H](O)[C@H](O)C4)[C@@]24C)[C@@]3(C)CC5)[C@@]5(C(O[C@@H]6O[C@@H](CO[C@@H]7C[C@@H](CO)[C@@H](O[C@H]8O[C@@H](C)[C@@H](O)[C@@H](O)[C@H]8O)[C@@H](O)[C@H]7O)[C@@H](O)[C@@H](O)[C@H]6O)=O)CC1 | InChI=1/C49H80O18/c1-21-10-13-49(15-14-47(6)25(31(49)22(21)2)8-9-30-45(4)17-26(52)41(61)46(5,20-51)29(45)11-12-48(30,47)7)44(62)67-43-39(60)36(57)34(55)28(65-43)19-63-27-16-24(18-50)40(37(58)33(27)54)66-42-38(59)35(56)32(53)23(3)64-42/h8,21-24,26-43,50-61H,9-20H2,1-7H3/t21-,22-,23+,24+,26-,27-,28+,29+,30+,31-,32-,33+,34-,35-,36-,37+,38-,39-,40-,41+,42-,43+,45+,46+,47-,48-,49-/m1/s1 |
|  | acteoside | 1 | 0 | 0 | 0 | 0 | 1 | 0 | C[C@H]1[C@@H](O)[C@H](O)[C@H](O)[C@@H](O[C@H]2[C@@H](OC(\C=C/c3cc(O)c(O)cc3)=O)[C@H](CO)O[C@@H](OCCc4cc(O)c(O)cc4)[C@@H]2O)O1 | InChI=1/C29H36O15/c1-13-22(36)23(37)24(38)29(41-13)44-27-25(39)28(40-9-8-15-3-6-17(32)19(34)11-15)42-20(12-30)26(27)43-21(35)7-4-14-2-5-16(31)18(33)10-14/h2-7,10-11,13,20,22-34,36-39H,8-9,12H2,1H3/b7-4-/t13-,20-,22+,23-,24-,25+,26-,27+,28+,29+/m0/s1 |
|  | leucosceptoside A | 1 | 0 | 0 | 0 | 0 | 1 | 0 | C[C@H]1[C@@H](O)[C@H](O)[C@H](O)[C@@H](O[C@H]2[C@@H](OC(\C=C/c3cc(OC)c(O)cc3)=O)[C@H](CO)O[C@@H](OCCc4cc(O)c(O)cc4)[C@@H]2O)O1 | InChI=1/C30H38O15/c1-14-23(36)24(37)25(38)30(42-14)45-28-26(39)29(41-10-9-16-3-6-17(32)19(34)11-16)43-21(13-31)27(28)44-22(35)8-5-15-4-7-18(33)20(12-15)40-2/h3-8,11-12,14,21,23-34,36-39H,9-10,13H2,1-2H3/b8-5-/t14-,21-,23+,24-,25-,26+,27-,28+,29+,30+/m0/s1 |
|  | acteoside isomer | 1 | 0 | 0 | 0 | 0 | 1 | 0 | C[C@H]1[C@@H](O)[C@H](O)[C@H](O)[C@@H](O[C@H]2[C@@H](CO)[C@@H](OC(\C=C/c3cc(O)c(O)cc3)=O)O[C@@H](OCCc4cc(O)c(O)cc4)[C@@H]2O)O1 | InChI=1/C29H36O15/c1-13-22(36)23(37)24(38)29(41-13)43-26-16(12-30)27(42-21(35)7-4-14-2-5-17(31)19(33)10-14)44-28(25(26)39)40-9-8-15-3-6-18(32)20(34)11-15/h2-7,10-11,13,16,22-34,36-39H,8-9,12H2,1H3/b7-4-/t13-,16+,22+,23-,24-,25+,26-,27-,28+,29+/m0/s1 |
|  | martynoside | 1 | 0 | 0 | 0 | 0 | 1 | 0 | C[C@H]1[C@@H](O)[C@H](O)[C@H](O)[C@@H](O[C@H]2[C@@H](OC(\C=C/c3cc(OC)c(O)cc3)=O)[C@H](CO)O[C@@H](OCCc4cc(O)c(OC)cc4)[C@@H]2O)O1 | InChI=1/C31H40O15/c1-15-24(36)25(37)26(38)31(43-15)46-29-27(39)30(42-11-10-17-5-8-20(40-2)19(34)12-17)44-22(14-32)28(29)45-23(35)9-6-16-4-7-18(33)21(13-16)41-3/h4-9,12-13,15,22,24-34,36-39H,10-11,14H2,1-3H3/b9-6-/t15-,22-,24+,25-,26-,27+,28-,29+,30+,31+/m0/s1 |
|  | isomartynoside | 1 | 0 | 0 | 0 | 0 | 1 | 0 | C[C@H]1[C@@H](O)[C@H](O)[C@H](O)[C@@H](O[C@H]2[C@@H](CO)[C@@H](OC(\C=C/c3cc(O)c(OC)cc3)=O)O[C@@H](OCCc4cc(O)c(OC)cc4)[C@@H]2O)O1 | InChI=1/C31H40O15/c1-15-24(36)25(37)26(38)31(43-15)45-28-18(14-32)29(44-23(35)9-6-16-4-7-21(40-2)19(33)12-16)46-30(27(28)39)42-11-10-17-5-8-22(41-3)20(34)13-17/h4-9,12-13,15,18,24-34,36-39H,10-11,14H2,1-3H3/b9-6-/t15-,18+,24+,25-,26-,27+,28-,29-,30+,31+/m0/s1 |
|  | 8b | 2 | 0 | 0 | 0 | 0 | 0 | 0 | C(=C\c1ccccc1)/C2=CCN=C2 | InChI=1/C12H11N/c1-2-4-11(5-3-1)6-7-12-8-9-13-10-12/h1-8,10H,9H2/b7-6+ |
|  | 8c | 2 | 0 | 0 | 0 | 0 | 0 | 0 | C(=C\c1ccccc1)/C2=NCC=C2 | InChI=1/C12H11N/c1-2-5-11(6-3-1)8-9-12-7-4-10-13-12/h1-9H,10H2/b9-8+ |
|  | 8d | 2 | 0 | 0 | 0 | 0 | 0 | 0 | C(=C\c1ccccc1)/C2=[S+]CC=C2 | InChI=1/C12H11S/c1-2-5-11(6-3-1)8-9-12-7-4-10-13-12/h1-9H,10H2/q+1/b9-8+ |
|  | 8j | 2 | 0 | 0 | 0 | 0 | 0 | 0 | C(=C\c1ccc(OC)cc1)/C2=NCC=C2 | InChI=1/C13H13NO/c1-15-13-8-5-11(6-9-13)4-7-12-3-2-10-14-12/h2-9H,10H2,1H3/b7-4+ |
|  | Phenyl-N-tert-butylnitrone | 2 | 0 | 1 | 0 | 0 | 0 | 0 | [O-]\[N+](\C(C)(C)C)=C/c1ccccc1 | InChI=1/C11H15NO/c1-11(2,3)12(13)9-10-7-5-4-6-8-10/h4-9H,1-3H3/b12-9- |
|  | 8i | 2 | 0 | 0 | 0 | 0 | 0 | 0 | C(=C\c1ccc(OC)cc1)/C2=CCC=[S+]2 | InChI=1/C13H13OS/c1-14-12-7-4-11(5-8-12)6-9-13-3-2-10-15-13/h3-10H,2H2,1H3/q+1/b9-6+ |
|  | 8a | 2 | 0 | 0 | 0 | 0 | 0 | 0 | C(=C\c1ccccc1)/c2c[nH]cc2 | InChI=1/C12H11N/c1-2-4-11(5-3-1)6-7-12-8-9-13-10-12/h1-10,13H/b7-6+ |
|  | indomethacin | 3 | 0 | 0 | 0 | 0 | 1 | 0 | Clc1ccc(C(n2c(C)c(CC(O)=O)c(cc(OC)cc3)c23)=O)cc1 | InChI=1/C19H16ClNO4/c1-11-15(10-18(22)23)16-9-14(25-2)7-8-17(16)21(11)19(24)12-3-5-13(20)6-4-12/h3-9H,10H2,1-2H3,(H,22,23) |
|  | Haloperidol | 3 | 0 | 0 | 0 | 0 | 0 | 0 | Fc1ccc(C(CCCN2CCC(c3ccc(Cl)cc3)(O)CC2)=O)cc1 | InChI=1/C21H23ClFNO2/c22-18-7-5-17(6-8-18)21(26)11-14-24(15-12-21)13-1-2-20(25)16-3-9-19(23)10-4-16/h3-10,26H,1-2,11-15H2 |
|  | Anandamide | 4 | 0 | 0 | 0 | 1 | 0 | 0 | O=C(CCC\C=C/C\C=C/C\C=C/C\C=C/CCCCC)NCCO | InChI=1/C22H37NO2/c1-2-3-4-5-6-7-8-9-10-11-12-13-14-15-16-17-18-19-22(25)23-20-21-24/h6-7,9-10,12-13,15-16,24H,2-5,8,11,14,17-21H2,1H3,(H,23,25)/b7-6-,10-9-,13-12-,16-15- |
|  | Arachidonic acid | 4 | 0 | 0 | 0 | 0 | 0 | 0 | CCCCC\C=C/C\C=C/C\C=C/C\C=C/CCCC(O)=O | InChI=1/C20H32O2/c1-2-3-4-5-6-7-8-9-10-11-12-13-14-15-16-17-18-19-20(21)22/h6-7,9-10,12-13,15-16H,2-5,8,11,14,17-19H2,1H3,(H,21,22)/b7-6-,10-9-,13-12-,16-15- |
|  | linoleic acid isomer | 4 | 0 | 0 | 0 | 0 | 0 | 0 | CCCCCC\C=C\C=C/CCCCCCCC(O)=O | InChI=1/C18H32O2/c1-2-3-4-5-6-7-8-9-10-11-12-13-14-15-16-17-18(19)20/h7-10H,2-6,11-17H2,1H3,(H,19,20)/b8-7+,10-9- |
|  | Compound 4c | 5 | 0 | 0 | 0 | 1 | 0 | 0 | c1(C#N)c(N)nc(c2c(O)cccc2OCC)cc1C3CCNCC3 | InChI=1/C19H22N4O2/c1-2-25-17-5-3-4-16(24)18(17)15-10-13(12-6-8-22-9-7-12)14(11-20)19(21)23-15/h3-5,10,12,22,24H,2,6-9H2,1H3,(H2,21,23) |
|  | Compound 3h | 5 | 0 | 0 | 0 | 1 | 0 | 0 | c1(C#N)c(N)nc(c2c(OC)cccc2O)cc1[C@@H]3CCCNC3 | InChI=1/C18H20N4O2/c1-24-16-6-2-5-15(23)17(16)14-8-12(11-4-3-7-21-10-11)13(9-19)18(20)22-14/h2,5-6,8,11,21,23H,3-4,7,10H2,1H3,(H2,20,22)/t11-/m1/s1 |
|  | Compound 3m | 5 | 0 | 0 | 0 | 1 | 0 | 0 | c1(C#N)c(N)nc(c2c(OC(C)C)cccc2O)cc1[C@@H]3CCCNC3 | InChI=1/C20H24N4O2/c1-12(2)26-18-7-3-6-17(25)19(18)16-9-14(13-5-4-8-23-11-13)15(10-21)20(22)24-16/h3,6-7,9,12-13,23,25H,4-5,8,11H2,1-2H3,(H2,22,24)/t13-/m1/s1 |
|  | Compound 3j | 5 | 0 | 0 | 0 | 1 | 0 | 0 | c1(C#N)c(N)nc(c2c(OCCC)cccc2O)cc1[C@@H]3CCCNC3 | InChI=1/C20H24N4O2/c1-2-9-26-18-7-3-6-17(25)19(18)16-10-14(13-5-4-8-23-12-13)15(11-21)20(22)24-16/h3,6-7,10,13,23,25H,2,4-5,8-9,12H2,1H3,(H2,22,24)/t13-/m1/s1 |
|  | Compound 4b | 5 | 0 | 0 | 0 | 1 | 0 | 0 | c1(C#N)c(N)nc(c2c(O)cccc2O)cc1C3CCNCC3 | InChI=1/C17H18N4O2/c18-9-12-11(10-4-6-20-7-5-10)8-13(21-17(12)19)16-14(22)2-1-3-15(16)23/h1-3,8,10,20,22-23H,4-7H2,(H2,19,21) |
|  | Compound 4k | 5 | 0 | 0 | 0 | 1 | 0 | 0 | c1(C#N)c(N)nc(c2c(O)cccc2OC3CCC3)cc1C4CCNCC4 | InChI=1/C21H24N4O2/c22-12-16-15(13-7-9-24-10-8-13)11-17(25-21(16)23)20-18(26)5-2-6-19(20)27-14-3-1-4-14/h2,5-6,11,13-14,24,26H,1,3-4,7-10H2,(H2,23,25) |
|  | Compound 3n | 5 | 0 | 0 | 0 | 1 | 0 | 0 | c1(C#N)c(N)nc(c2c(OCC(C)C)cccc2O)cc1[C@@H]3CCCNC3 | InChI=1/C21H26N4O2/c1-13(2)12-27-19-7-3-6-18(26)20(19)17-9-15(14-5-4-8-24-11-14)16(10-22)21(23)25-17/h3,6-7,9,13-14,24,26H,4-5,8,11-12H2,1-2H3,(H2,23,25)/t14-/m1/s1 |
|  | Compound 4e | 5 | 0 | 0 | 0 | 1 | 0 | 0 | c1(C#N)c(N)nc(c2c(O)cccc2OCCCC)cc1C3CCNCC3 | InChI=1/C21H26N4O2/c1-2-3-11-27-19-6-4-5-18(26)20(19)17-12-15(14-7-9-24-10-8-14)16(13-22)21(23)25-17/h4-6,12,14,24,26H,2-3,7-11H2,1H3,(H2,23,25) |
|  | Compound 3o | 5 | 0 | 0 | 0 | 1 | 0 | 0 | c1(C#N)c(N)nc(c2c(OCC3CC3)cccc2O)cc1[C@@H]4CCCNC4 | InChI=1/C21H24N4O2/c22-10-16-15(14-3-2-8-24-11-14)9-17(25-21(16)23)20-18(26)4-1-5-19(20)27-12-13-6-7-13/h1,4-5,9,13-14,24,26H,2-3,6-8,11-12H2,(H2,23,25)/t14-/m1/s1 |
|  | Compound 4 | 8 | 1 | 0 | 0 | 0 | 0 | 0 | C=C1[C@@H]([C@@H](C[C@@]([C@H]2[C@@H]3C(C)=CC2=O)(C)O)OC(=O)C=C)[C@@H]3OC1=O | InChI=1/C18H20O6/c1-5-12(20)23-11-7-18(4,22)15-10(19)6-8(2)13(15)16-14(11)9(3)17(21)24-16/h5-6,11,13-16,22H,1,3,7H2,2,4H3/t11-,13+,14+,15-,16-,18-/m1/s1 |
|  | Compound 3 | 8 | 1 | 0 | 0 | 0 | 0 | 0 | C=C1[C@@H]([C@@H](C[C@]([C@H]2[C@@H]3C(C)=CC2=O)(C)O)OC(=O)C=C)[C@@H]3OC1=O | InChI=1/C18H20O6/c1-5-12(20)23-11-7-18(4,22)15-10(19)6-8(2)13(15)16-14(11)9(3)17(21)24-16/h5-6,11,13-16,22H,1,3,7H2,2,4H3/t11-,13+,14+,15-,16-,18+/m1/s1 |
|  | Compound 1 | 8 | 1 | 0 | 0 | 0 | 0 | 0 | C=C1[C@@H]([C@@H](CC(=C2[C@@H]3C(C)=CC2=O)C)OC(=O)C=C)[C@@H]3OC1=O | InChI=1/C18H18O5/c1-5-13(20)22-12-7-9(3)14-11(19)6-8(2)15(14)17-16(12)10(4)18(21)23-17/h5-6,12,15-17H,1,4,7H2,2-3H3/t12-,15+,16+,17-/m1/s1 |
|  | 2-active | 9 | 0 | 0 | 0 | 0 | 1 | 0 | c1(ccc(Cl)cc1)S(c2nn(nnn3)c3cc2)=O | InChI=1/C10H6ClN5OS/c11-7-1-3-8(4-2-7)18(17)10-6-5-9-12-14-15-16(9)13-10/h1-6H |
|  | Ro106-9920 | 9 | 0 | 0 | 0 | 0 | 1 | 0 | c1(ccccc1)S(c2nn(nnn3)c3cc2)=O | InChI=1/C10H7N5OS/c16-17(8-4-2-1-3-5-8)10-7-6-9-11-13-14-15(9)12-10/h1-7H |
|  | 1-active | 9 | 0 | 0 | 0 | 0 | 1 | 0 | c1(ccccc1)S(c2nn(nnn3)c3cc2)(=O)=O | InChI=1/C10H7N5O2S/c16-18(17,8-4-2-1-3-5-8)10-7-6-9-11-13-14-15(9)12-10/h1-7H |
|  | oxidopamine | 10 | 0 | 0 | 0 | 0 | 0 | 0 | c1c(O)c(O)cc(O)c1CCN | InChI=1/C8H11NO3/c9-2-1-5-3-7(11)8(12)4-6(5)10/h3-4,10-12H,1-2,9H2 |
|  | isoproterenol | 10 | 0 | 0 | 0 | 0 | 1 | 0 | Oc1c(O)cc([C@H](CNC(C)C)O)cc1 | InChI=1/C11H17NO3/c1-7(2)12-6-11(15)8-3-4-9(13)10(14)5-8/h3-5,7,11-15H,6H2,1-2H3/t11-/m0/s1 |
|  | Gallic Acid | 10 | 1 | 0 | 0 | 0 | 0 | 0 | c1cc(O)c(O)c(O)c1C(O)=O | InChI=1/C7H6O5/c8-4-2-1-3(7(11)12)5(9)6(4)10/h1-2,8-10H,(H,11,12) |
|  | 1,24-dihydroxy-22-ene-24-cyclopropyl-vitamin D | 11 | 0 | 0 | 1 | 0 | 0 | 0 | C[C@@H]([C@@H]1[C@]2(C)[C@H](\C(=C\C=C(\C(=C)[C@@H](O)C[C@@H]3O)/C3)\CCC2)CC1)\C=C(/CC4CC4)\O | InChI=1/C27H40O3/c1-17(13-22(28)14-19-6-7-19)24-10-11-25-20(5-4-12-27(24,25)3)8-9-21-15-23(29)16-26(30)18(21)2/h8-9,13,17,19,23-26,28-30H,2,4-7,10-12,14-16H2,1,3H3/b20-8+,21-9+,22-13+/t17-,23-,24-,25+,26+,27-/m1/s1 |
|  | 1,25-dihydroxyvitamin D3 | 11 | 0 | 0 | 1 | 0 | 0 | 0 | C[C@@H]([C@@H]1[C@]2(C)[C@H](\C(=C\C=C(\C(=C)[C@@H](O)C[C@@H]3O)/C3)\CCC2)CC1)CCCC(O)(C)C | InChI=1/C27H44O3/c1-18(8-6-14-26(3,4)30)23-12-13-24-20(9-7-15-27(23,24)5)10-11-21-16-22(28)17-25(29)19(21)2/h10-11,18,22-25,28-30H,2,6-9,12-17H2,1,3-5H3/b20-10+,21-11+/t18-,22-,23-,24+,25+,27-/m1/s1 |
|  | 1a,25-dihydroxylumisterol | 11 | 0 | 0 | 1 | 0 | 0 | 0 | C[C@@H]([C@@H]1[C@@](C)(C[C@H]([C@H]2C(=C3)[C@@H](O)C[C@H](O)C2)C(=C3)C4)[C@H]4CC1)CCCC(C)(C)O | InChI=1/C26H42O3/c1-16(6-5-11-25(2,3)29)23-10-8-18-12-17-7-9-20-21(13-19(27)14-24(20)28)22(17)15-26(18,23)4/h7,9,16,18-19,21-24,27-29H,5-6,8,10-15H2,1-4H3/t16-,18+,19-,21-,22+,23-,24+,26+/m1/s1 |
|  | PA-3 (Tylophoridicine C) | 12 | 0 | 0 | 0 | 0 | 0 | 0 | c12c(C[N@+]3([C@@H](CCC3)[C@@H]1O)[O-])c(cc(OC)c(O)c4)c4c(cc(OC)cc5)c25 | InChI=1/C22H23NO5/c1-27-12-5-6-13-14(8-12)15-9-19(24)20(28-2)10-16(15)17-11-23(26)7-3-4-18(23)22(25)21(13)17/h5-6,8-10,18,22,24-25H,3-4,7,11H2,1-2H3/t18-,22-,23+/m0/s1 |
|  | PA-7 (Tylophorinidine) | 12 | 0 | 0 | 0 | 0 | 0 | 0 | c12c(C[N@+]3([C@@H](CCC3)[C@@H]1O)[O-])c(cc(OC)c(O)c4)c4c(cc(OC)cc5)c25 | InChI=1/C22H23NO5/c1-27-12-5-6-13-14(8-12)15-9-19(24)20(28-2)10-16(15)17-11-23(26)7-3-4-18(23)22(25)21(13)17/h5-6,8-10,18,22,24-25H,3-4,7,11H2,1-2H3/t18-,22-,23+/m0/s1 |
|  | PA-6 (Tylophorinine) | 12 | 0 | 0 | 0 | 0 | 0 | 0 | c12c(C[N@+]3([C@@H](CCC3)[C@@H]1O)[O-])c(cc(OC)c(OC)c4)c4c(cc(OC)cc5)c25 | InChI=1/C23H25NO5/c1-27-13-6-7-14-15(9-13)16-10-20(28-2)21(29-3)11-17(16)18-12-24(26)8-4-5-19(24)23(25)22(14)18/h6-7,9-11,19,23,25H,4-5,8,12H2,1-3H3/t19-,23-,24+/m0/s1 |
|  | PA-4 (Tylophoridicine F) | 12 | 0 | 0 | 0 | 0 | 0 | 0 | c12c(C[N@+]3([C@@H](CCC3)[C@@H]1O)[O-])c(cc(OC)c(OC)c4)c4c(cc(OC)cc5)c25 | InChI=1/C23H25NO5/c1-27-13-6-7-14-15(9-13)16-10-20(28-2)21(29-3)11-17(16)18-12-24(26)8-4-5-19(24)23(25)22(14)18/h6-7,9-11,19,23,25H,4-5,8,12H2,1-3H3/t19-,23-,24+/m0/s1 |
|  | PA-2 (Tylophoridicine E) | 12 | 0 | 0 | 0 | 0 | 0 | 0 | c12c(CN3[C@@H](CCC3)[C@@H]1O)c(cc(OC)c(OC)c4)c4c(cc(O)cc5)c25 | InChI=1/C22H23NO4/c1-26-19-9-15-14-8-12(24)5-6-13(14)21-17(16(15)10-20(19)27-2)11-23-7-3-4-18(23)22(21)25/h5-6,8-10,18,22,24-25H,3-4,7,11H2,1-2H3/t18-,22-/m0/s1 |
|  | DCB-3506 | 12 | 0 | 0 | 0 | 0 | 0 | 0 | c12c(CN3[C@@H](CCC3)[C@@H]1O)c(cc(OC)c(OC)c4)c4c(cc(O)c(OC)c5)c25 | InChI=1/C23H25NO5/c1-27-19-10-15-12(7-18(19)25)13-8-20(28-2)21(29-3)9-14(13)16-11-24-6-4-5-17(24)23(26)22(15)16/h7-10,17,23,25-26H,4-6,11H2,1-3H3/t17-,23-/m0/s1 |
|  | PA-5 [(R)-(+)-Deoxytylophorinidine] | 12 | 0 | 0 | 0 | 0 | 0 | 0 | c12c(C[N@+]3([C@H](CCC3)C1)[O-])c(cc(OC)c(OC)c4)c4c(cc(OC)cc5)c25 | InChI=1/C23H25NO4/c1-26-15-6-7-16-17-9-14-5-4-8-24(14,25)13-21(17)20-12-23(28-3)22(27-2)11-19(20)18(16)10-15/h6-7,10-12,14H,4-5,8-9,13H2,1-3H3/t14-,24-/m1/s1 |
|  | DCB-3503 | 12 | 0 | 0 | 0 | 0 | 0 | 0 | c12c(CN3[C@@H](CCC3)[C@@H]1O)c(cc(OC)c(OC)c4)c4c(cc(OC)c(OC)c5)c25 | InChI=1/C24H27NO5/c1-27-19-8-13-14-9-20(28-2)22(30-4)11-16(14)23-17(15(13)10-21(19)29-3)12-25-7-5-6-18(25)24(23)26/h8-11,18,24,26H,5-7,12H2,1-4H3/t18-,24-/m0/s1 |
|  | DCB-3501 | 12 | 0 | 0 | 0 | 0 | 0 | 0 | c12c(CN3[C@@H](CCC3)[C@H]1O)c(cc(OC)c(OC)c4)c4c(cc(OC)c(OC)c5)c25 | InChI=1/C24H27NO5/c1-27-19-8-13-14-9-20(28-2)22(30-4)11-16(14)23-17(15(13)10-21(19)29-3)12-25-7-5-6-18(25)24(23)26/h8-11,18,24,26H,5-7,12H2,1-4H3/t18-,24+/m0/s1 |
|  | Compound 13 | 13 | 0 | 0 | 0 | 1 | 0 | 0 | c1(ccccc1O)c2cc(c3ccc(N4CCCC4)c(NC(=O)CCN5CCCCC5)c3)c(C#N)c(N)n2 | InChI=1/C30H34N6O2/c31-20-24-23(19-25(34-30(24)32)22-8-2-3-9-28(22)37)21-10-11-27(36-15-6-7-16-36)26(18-21)33-29(38)12-17-35-13-4-1-5-14-35/h2-3,8-11,18-19,37H,1,4-7,12-17H2,(H2,32,34)(H,33,38) |
|  | Compound 43 | 13 | 0 | 0 | 0 | 1 | 0 | 0 | c1(ccccc1O)c2cc(c3ccc(N4CCCCC4)c(NC(=O)CCN5CCCCC5)c3)c(C#N)c(N)n2 | InChI=1/C31H36N6O2/c32-21-25-24(20-26(35-31(25)33)23-9-3-4-10-29(23)38)22-11-12-28(37-16-7-2-8-17-37)27(19-22)34-30(39)13-18-36-14-5-1-6-15-36/h3-4,9-12,19-20,38H,1-2,5-8,13-18H2,(H2,33,35)(H,34,39) |
|  | Compound 12 | 13 | 0 | 0 | 0 | 1 | 0 | 0 | c1(ccccc1O)c2cc(c3ccc(N(C)C)c(NC(=O)CCN4CCCCC4)c3)c(C#N)c(N)n2 | InChI=1/C28H32N6O2/c1-33(2)25-11-10-19(16-24(25)31-27(36)12-15-34-13-6-3-7-14-34)21-17-23(32-28(30)22(21)18-29)20-8-4-5-9-26(20)35/h4-5,8-11,16-17,35H,3,6-7,12-15H2,1-2H3,(H2,30,32)(H,31,36) |
|  | Compound 27 | 13 | 0 | 0 | 0 | 1 | 0 | 0 | c1(ccccc1O)c2cc(c3ccc(NC(CCN4CCCCC4)=O)cc3)c(C#N)c(N)n2 | InChI=1/C26H27N5O2/c27-17-22-21(16-23(30-26(22)28)20-6-2-3-7-24(20)32)18-8-10-19(11-9-18)29-25(33)12-15-31-13-4-1-5-14-31/h2-3,6-11,16,32H,1,4-5,12-15H2,(H2,28,30)(H,29,33) |
|  | Compound 26 | 13 | 0 | 0 | 0 | 1 | 0 | 0 | c1(ccccc1O)c2cc(c3cccc(NC(=O)CCN4CCCCC4)c3)c(C#N)c(N)n2 | InChI=1/C26H27N5O2/c27-17-22-21(16-23(30-26(22)28)20-9-2-3-10-24(20)32)18-7-6-8-19(15-18)29-25(33)11-14-31-12-4-1-5-13-31/h2-3,6-10,15-16,32H,1,4-5,11-14H2,(H2,28,30)(H,29,33) |
|  | Compound 42 | 13 | 0 | 0 | 0 | 1 | 0 | 0 | c1(ccccc1O)c2cc(c3cccc(NC(=O)CCN4CCCCC4)c3)c(C#N)c(NC(=O)C)n2 | InChI=1/C28H29N5O3/c1-19(34)30-28-24(18-29)23(17-25(32-28)22-10-3-4-11-26(22)35)20-8-7-9-21(16-20)31-27(36)12-15-33-13-5-2-6-14-33/h3-4,7-11,16-17,35H,2,5-6,12-15H2,1H3,(H,31,36)(H,30,32,34) |
|  | Compound 30 | 13 | 0 | 0 | 0 | 1 | 0 | 0 | c1(ccccc1O)c2cc(c3ccc(C(=O)NCCN4CCCCC4)cc3)c(C#N)c(N)n2 | InChI=1/C26H27N5O2/c27-17-22-21(16-23(30-25(22)28)20-6-2-3-7-24(20)32)18-8-10-19(11-9-18)26(33)29-12-15-31-13-4-1-5-14-31/h2-3,6-11,16,32H,1,4-5,12-15H2,(H2,28,30)(H,29,33) |
|  | Compound 29 | 13 | 0 | 0 | 0 | 1 | 0 | 0 | c1(ccccc1O)c2cc(c3cccc(C(=O)NCCN4CCCCC4)c3)c(C#N)c(N)n2 | InChI=1/C26H27N5O2/c27-17-22-21(16-23(30-25(22)28)20-9-2-3-10-24(20)32)18-7-6-8-19(15-18)26(33)29-11-14-31-12-4-1-5-13-31/h2-3,6-10,15-16,32H,1,4-5,11-14H2,(H2,28,30)(H,29,33) |
|  | Compound 28 | 13 | 0 | 0 | 0 | 1 | 0 | 0 | c1(ccccc1O)c2cc(c3ccccc3C(=O)NCCN4CCCCC4)c(C#N)c(N)n2 | InChI=1/C26H27N5O2/c27-17-22-21(16-23(30-25(22)28)20-10-4-5-11-24(20)32)18-8-2-3-9-19(18)26(33)29-12-15-31-13-6-1-7-14-31/h2-5,8-11,16,32H,1,6-7,12-15H2,(H2,28,30)(H,29,33) |
|  | Raloxifene | 13 | 0 | 0 | 0 | 0 | 0 | 0 | O=C(c1c(ccc(O)c2)c2sc1c3ccc(O)cc3)c4ccc(OCCN5CCCCC5)cc4 | InChI=1/C28H27NO4S/c30-21-8-4-20(5-9-21)28-26(24-13-10-22(31)18-25(24)34-28)27(32)19-6-11-23(12-7-19)33-17-16-29-14-2-1-3-15-29/h4-13,18,30-31H,1-3,14-17H2 |
|  | Pyrazolo[4,3-c]quinoline derivative | 13 | 0 | 0 | 0 | 1 | 0 | 0 | c12c(n(c3cc(OCO4)c4cc3)nc1C(=O)N)c5c(ccc(NC(=O)c6c(Cl)nccc6)c5)N(C(=O)C)C2 | InChI=1/C26H19ClN6O5/c1-13(34)32-11-18-22(25(28)35)31-33(15-5-7-20-21(10-15)38-12-37-20)23(18)17-9-14(4-6-19(17)32)30-26(36)16-3-2-8-29-24(16)27/h2-10H,11-12H2,1H3,(H2,28,35)(H,30,36) |
|  | 6q | 14 | 0 | 0 | 0 | 0 | 0 | 0 | c1(cc(OC)c(\C=C\c2ccc(OC)cc2)cc1OC)OC | InChI=1/C18H20O4/c1-19-15-9-6-13(7-10-15)5-8-14-11-17(21-3)18(22-4)12-16(14)20-2/h5-12H,1-4H3/b8-5+ |
|  | 6l | 14 | 0 | 0 | 0 | 0 | 0 | 0 | c1(c(OC)cc(\C=C\c2ccc(OC)cc2)cc1OC)OC | InChI=1/C18H20O4/c1-19-15-9-7-13(8-10-15)5-6-14-11-16(20-2)18(22-4)17(12-14)21-3/h5-12H,1-4H3/b6-5+ |
|  | 6u | 14 | 0 | 0 | 0 | 0 | 0 | 0 | c1(cc(OC)c(\C=C\c2ccc(OC)cc2)cc1)OC | InChI=1/C17H18O3/c1-18-15-9-5-13(6-10-15)4-7-14-8-11-16(19-2)12-17(14)20-3/h4-12H,1-3H3/b7-4+ |
|  | 6y | 14 | 0 | 0 | 0 | 0 | 0 | 0 | c1cc(OC)c(\C=C\c2ccc(OC)cc2)cc1OC | InChI=1/C17H18O3/c1-18-15-8-5-13(6-9-15)4-7-14-12-16(19-2)10-11-17(14)20-3/h4-12H,1-3H3/b7-4+ |
|  | 6s | 14 | 0 | 0 | 0 | 0 | 0 | 0 | c1(c(OC)c(OC)c(\C=C\c2ccc(OC)cc2)cc1)OC | InChI=1/C18H20O4/c1-19-15-10-6-13(7-11-15)5-8-14-9-12-16(20-2)18(22-4)17(14)21-3/h5-12H,1-4H3/b8-5+ |
|  | 6a | 14 | 0 | 0 | 0 | 0 | 0 | 0 | c1(c(OC)cc(\C=C\c2ccc(OC)cc2)cc1)OC | InChI=1/C17H18O3/c1-18-15-9-6-13(7-10-15)4-5-14-8-11-16(19-2)17(12-14)20-3/h4-12H,1-3H3/b5-4+ |
|  | 6f | 14 | 0 | 0 | 0 | 0 | 0 | 0 | c1c(OC)cc(\C=C\c2ccc(OC)cc2)cc1OC | InChI=1/C17H18O3/c1-18-15-8-6-13(7-9-15)4-5-14-10-16(19-2)12-17(11-14)20-3/h4-12H,1-3H3/b5-4+ |
|  | 6o | 14 | 0 | 0 | 0 | 0 | 0 | 0 | c1c(OC)c(OC)c(\C=C\c2ccc(OC)cc2)cc1 | InChI=1/C17H18O3/c1-18-15-11-8-13(9-12-15)7-10-14-5-4-6-16(19-2)17(14)20-3/h4-12H,1-3H3/b10-7+ |
|  | 6g | 14 | 0 | 0 | 0 | 0 | 0 | 0 | c1cc(OC)c(\C=C\c2ccc(OC)cc2)c(OC)c1 | InChI=1/C17H18O3/c1-18-14-10-7-13(8-11-14)9-12-15-16(19-2)5-4-6-17(15)20-3/h4-12H,1-3H3/b12-9+ |
|  | 4ff | 14 | 0 | 0 | 0 | 0 | 0 | 0 | c1(c(OC)cc(\C=C\c2ccccc2)cc1OC)OC | InChI=1/C17H18O3/c1-18-15-11-14(12-16(19-2)17(15)20-3)10-9-13-7-5-4-6-8-13/h4-12H,1-3H3/b10-9+ |
|  | 6v | 14 | 0 | 0 | 0 | 0 | 0 | 0 | c1(c(OC)c(CC)c(\C=C\c2ccc(OC)cc2)cc1)OC | InChI=1/C19H22O3/c1-5-17-15(10-13-18(21-3)19(17)22-4)9-6-14-7-11-16(20-2)12-8-14/h6-13H,5H2,1-4H3/b9-6+ |
|  | 4gg | 14 | 0 | 0 | 0 | 0 | 0 | 0 | c1(c(OC)c(OC)c(\C=C\c2ccccc2)cc1)OC | InChI=1/C17H18O3/c1-18-15-12-11-14(16(19-2)17(15)20-3)10-9-13-7-5-4-6-8-13/h4-12H,1-3H3/b10-9+ |
|  | 4u | 14 | 0 | 0 | 0 | 0 | 0 | 0 | c1(cc(OC)c(\C=C\c2ccccc2)cc1)OC | InChI=1/C16H16O2/c1-17-15-11-10-14(16(12-15)18-2)9-8-13-6-4-3-5-7-13/h3-12H,1-2H3/b9-8+ |
|  | N,N'-[4-(dimethylamino)-2,6-pyridinylidenedimethyl] bis (S,S) (2'-tritylperoxyhistidine) | 15 | 1 | 0 | 0 | 0 | 0 | 0 | c1c(CN[C@@H](C(OO)=O)CC2=NCN=C2C(c3ccccc3)(c4ccccc4)c5ccccc5)nc(CN[C@H](C(OO)=O)Cc6c[nH]c(C(c7ccccc7)(c8ccccc8)c9ccccc9)n6)cc1N(C)C | InChI=1/C59H56N8O6/c1-67(2)50-33-47(37-60-52(55(68)72-70)35-49-39-62-57(66-49)59(44-27-15-6-16-28-44,45-29-17-7-18-30-45)46-31-19-8-20-32-46)65-48(34-50)38-61-53(56(69)73-71)36-51-54(64-40-63-51)58(41-21-9-3-10-22-41,42-23-11-4-12-24-42)43-25-13-5-14-26-43/h3-34,39,52-53,60-61,70-71H,35-38,40H2,1-2H3,(H,62,66)/t52-,53+/m0/s1 |
|  | N,N'-[4-(dimethylamino)-2,6-pyridinylidenedimethyl] bis (R,S) (2'-tritylperoxyhistidine) | 15 | 1 | 0 | 0 | 0 | 0 | 0 | c1c(CN[C@H](C(OO)=O)CC2=NCN=C2C(c3ccccc3)(c4ccccc4)c5ccccc5)nc(CN[C@H](C(OO)=O)Cc6c[nH]c(C(c7ccccc7)(c8ccccc8)c9ccccc9)n6)cc1N(C)C | InChI=1/C59H56N8O6/c1-67(2)50-33-47(37-60-52(55(68)72-70)35-49-39-62-57(66-49)59(44-27-15-6-16-28-44,45-29-17-7-18-30-45)46-31-19-8-20-32-46)65-48(34-50)38-61-53(56(69)73-71)36-51-54(64-40-63-51)58(41-21-9-3-10-22-41,42-23-11-4-12-24-42)43-25-13-5-14-26-43/h3-34,39,52-53,60-61,70-71H,35-38,40H2,1-2H3,(H,62,66)/t52-,53-/m0/s1 |
|  | N,N'-[4-(dimethylamino)-2,6-pyridinylidenedimethyl] bis (S,S) (2'-tritylhistidine)-dimethylester | 15 | 1 | 0 | 0 | 0 | 0 | 0 | c1c(CN[C@H](C(OC)=O)CC2=NCN=C2C(c3ccccc3)(c4ccccc4)c5ccccc5)nc(CN[C@@H](C(OC)=O)Cc6c[nH]c(C(c7ccccc7)(c8ccccc8)c9ccccc9)n6)cc1N(C)C | InChI=1/C61H60N8O4/c1-69(2)52-35-49(39-62-54(57(70)72-3)37-51-41-64-59(68-51)61(46-29-17-8-18-30-46,47-31-19-9-20-32-47)48-33-21-10-22-34-48)67-50(36-52)40-63-55(58(71)73-4)38-53-56(66-42-65-53)60(43-23-11-5-12-24-43,44-25-13-6-14-26-44)45-27-15-7-16-28-45/h5-36,41,54-55,62-63H,37-40,42H2,1-4H3,(H,64,68)/t54-,55+/m1/s1 |
|  | N,N'-[4-(dimethylamino)-2,6-pyridinylidenedimethyl] bis (S,S) (histidine)-dimethylester, | 15 | 0 | 0 | 0 | 0 | 0 | 0 | c1c(CN[C@@H](C(O)=O)CC2=NCN=C2C(c3ccccc3)(c4ccccc4)c5ccccc5)nc(CN[C@@H](C(O)=O)Cc6c[nH]c(C(c7ccccc7)(c8ccccc8)c9ccccc9)n6)cc1N(C)C | InChI=1/C59H56N8O4/c1-67(2)50-33-47(37-60-52(55(68)69)35-49-39-62-57(66-49)59(44-27-15-6-16-28-44,45-29-17-7-18-30-45)46-31-19-8-20-32-46)65-48(34-50)38-61-53(56(70)71)36-51-54(64-40-63-51)58(41-21-9-3-10-22-41,42-23-11-4-12-24-42)43-25-13-5-14-26-43/h3-34,39,52-53,60-61H,35-38,40H2,1-2H3,(H,62,66)(H,68,69)(H,70,71)/t52-,53-/m1/s1 |
|  | N,N'-[4-(dimethylamino)-2,6-pyridinylidenedimethyl] bis (R,S) (histidine)-dimethylester, | 15 | 0 | 0 | 0 | 0 | 0 | 0 | c1c(CN[C@@H](C(O)=O)CC2=NCN=C2C(c3ccccc3)(c4ccccc4)c5ccccc5)nc(CN[C@H](C(O)=O)Cc6c[nH]c(C(c7ccccc7)(c8ccccc8)c9ccccc9)n6)cc1N(C)C | InChI=1/C59H56N8O4/c1-67(2)50-33-47(37-60-52(55(68)69)35-49-39-62-57(66-49)59(44-27-15-6-16-28-44,45-29-17-7-18-30-45)46-31-19-8-20-32-46)65-48(34-50)38-61-53(56(70)71)36-51-54(64-40-63-51)58(41-21-9-3-10-22-41,42-23-11-4-12-24-42)43-25-13-5-14-26-43/h3-34,39,52-53,60-61H,35-38,40H2,1-2H3,(H,62,66)(H,68,69)(H,70,71)/t52-,53+/m0/s1 |
|  | thiophenecarboxamide\_baxter\_16 | 20 | 0 | 0 | 0 | 1 | 0 | 0 | COc1c(c2sc(C(=O)N)c(NC(=O)N)c2)cccc1 | InChI=1/C13H13N3O3S/c1-19-9-5-3-2-4-7(9)10-6-8(16-13(15)18)11(20-10)12(14)17/h2-6H,1H3,(H2,14,17)(H3,15,16,18) |
|  | thiophenecarboxamide\_baxter\_18 | 20 | 0 | 0 | 0 | 1 | 0 | 0 | COc1ccc(c2sc(C(=O)N)c(NC(=O)N)c2)cc1 | InChI=1/C13H13N3O3S/c1-19-8-4-2-7(3-5-8)10-6-9(16-13(15)18)11(20-10)12(14)17/h2-6H,1H3,(H2,14,17)(H3,15,16,18) |
|  | thiophenecarboxamide\_baxter\_4 | 20 | 0 | 0 | 0 | 1 | 0 | 0 | NC(Nc1c(C(=O)N)sc(c2ccccc2)c1)=O | InChI=1/C12H11N3O2S/c13-11(16)10-8(15-12(14)17)6-9(18-10)7-4-2-1-3-5-7/h1-6H,(H2,13,16)(H3,14,15,17) |
|  | thiophenecarboxamide\_baxter\_19 | 20 | 0 | 0 | 0 | 1 | 0 | 0 | NC(Nc1c(C(=O)N)sc(c2c(Cl)cccc2)c1)=O | InChI=1/C12H10ClN3O2S/c13-7-4-2-1-3-6(7)9-5-8(16-12(15)18)10(19-9)11(14)17/h1-5H,(H2,14,17)(H3,15,16,18) |
|  | thiophenecarboxamide\_baxter\_8 | 20 | 0 | 0 | 0 | 1 | 0 | 0 | CNC(Nc1c(C(=O)N)sc(c2ccccc2)c1)=O | InChI=1/C13H13N3O2S/c1-15-13(18)16-9-7-10(19-11(9)12(14)17)8-5-3-2-4-6-8/h2-7H,1H3,(H2,14,17)(H2,15,16,18) |
|  | thiophenecarboxamide\_baxter\_21 | 20 | 0 | 0 | 0 | 1 | 0 | 0 | NC(Nc1c(C(=O)N)sc(c2ccc(O)cc2)c1)=O | InChI=1/C12H11N3O3S/c13-11(17)10-8(15-12(14)18)5-9(19-10)6-1-3-7(16)4-2-6/h1-5,16H,(H2,13,17)(H3,14,15,18) |
|  | thiophenecarboxamide\_baxter\_13 | 20 | 0 | 0 | 0 | 1 | 0 | 0 | NC(Nc1c(C(=O)N)cc(c2ccccc2)s1)=O | InChI=1/C12H11N3O2S/c13-10(16)8-6-9(7-4-2-1-3-5-7)18-11(8)15-12(14)17/h1-6H,(H2,13,16)(H3,14,15,17) |
|  | thiophenecarboxamide\_baxter\_15 | 20 | 0 | 0 | 0 | 1 | 0 | 0 | NC(Nc1c(C(=O)N)nc(c2ccccc2)cn1)=O | InChI=1/C12H11N5O2/c13-10(18)9-11(17-12(14)19)15-6-8(16-9)7-4-2-1-3-5-7/h1-6H,(H2,13,18)(H3,14,15,17,19) |
|  | thiophenecarboxamide\_baxter\_14 | 20 | 0 | 0 | 0 | 1 | 0 | 0 | NC(Nc1c(C(=O)N)[nH]c(c2ccc(F)cc2)n1)=O | InChI=1/C11H10FN5O2/c12-6-3-1-5(2-4-6)9-15-7(8(13)18)10(16-9)17-11(14)19/h1-4H,(H2,13,18)(H,15,16)(H3,14,17,19) |
|  | beta Carboline 21 | 21 | 0 | 0 | 0 | 1 | 0 | 0 | c12c(ccnc1)c(cc(Cl)cc3NC(=O)C)c3[nH]2 | InChI=1/C13H10ClN3O/c1-7(18)16-11-5-8(14)4-10-9-2-3-15-6-12(9)17-13(10)11/h2-6,17H,1H3,(H,16,18) |
|  | beta Carboline 33 | 21 | 0 | 0 | 0 | 1 | 0 | 0 | c12c(ccnc1)c(cc(Cl)cc3NC(=O)OC)c3[nH]2 | InChI=1/C13H10ClN3O2/c1-19-13(18)17-10-5-7(14)4-9-8-2-3-15-6-11(8)16-12(9)10/h2-6,16H,1H3,(H,17,18) |
|  | beta Carboline 18 | 21 | 0 | 0 | 0 | 1 | 0 | 0 | c12c(ccnc1)c(cc(Cl)cc3NC)c3[nH]2 | InChI=1/C12H10ClN3/c1-14-10-5-7(13)4-9-8-2-3-15-6-11(8)16-12(9)10/h2-6,14,16H,1H3 |
|  | beta Carboline 31 | 21 | 0 | 0 | 0 | 1 | 0 | 0 | c12c(ccnc1)c(cc(Cl)cc3NS(=O)(=O)C)c3[nH]2 | InChI=1/C12H10ClN3O2S/c1-19(17,18)16-10-5-7(13)4-9-8-2-3-14-6-11(8)15-12(9)10/h2-6,15-16H,1H3 |
|  | beta Carboline 16 | 21 | 0 | 0 | 0 | 1 | 0 | 0 | c12c(ccnc1)c(cc(Cl)cc3[N+]([O-])=O)c3[nH]2 | InChI=1/C11H6ClN3O2/c12-6-3-8-7-1-2-13-5-9(7)14-11(8)10(4-6)15(16)17/h1-5,14H |
|  | beta Carboline 19 | 21 | 0 | 0 | 0 | 1 | 0 | 0 | c12c(ccnc1)c(cc(Cl)cc3N(C)C)c3[nH]2 | InChI=1/C13H12ClN3/c1-17(2)12-6-8(14)5-10-9-3-4-15-7-11(9)16-13(10)12/h3-7,16H,1-2H3 |
|  | beta Carboline 27 | 21 | 0 | 0 | 0 | 1 | 0 | 0 | c12c(ccnc1)c(cc(Cl)cc3NC(=O)c4ccncc4)c3[nH]2 | InChI=1/C17H11ClN4O/c18-11-7-13-12-3-6-20-9-15(12)21-16(13)14(8-11)22-17(23)10-1-4-19-5-2-10/h1-9,21H,(H,22,23) |
|  | beta-Carboline 9 | 21 | 0 | 0 | 0 | 1 | 0 | 0 | c12c(ccnc1)c(cc(Cl)cc3Cl)c3[nH]2 | InChI=1/C11H6Cl2N2/c12-6-3-8-7-1-2-14-5-10(7)15-11(8)9(13)4-6/h1-5,15H |
|  | beta Carboline 15 | 21 | 0 | 0 | 0 | 1 | 0 | 0 | c12c(ccnc1)c(cc(Cl)c(O)c3Cl)c3[nH]2 | InChI=1/C11H6Cl2N2O/c12-7-3-6-5-1-2-14-4-8(5)15-10(6)9(13)11(7)16/h1-4,15-16H |
|  | beta Carboline 26 | 21 | 0 | 0 | 0 | 1 | 0 | 0 | c12c(ccnc1)c(cc(Cl)cc3NC(=O)c4cnccc4)c3[nH]2 | InChI=1/C17H11ClN4O/c18-11-6-13-12-3-5-20-9-15(12)21-16(13)14(7-11)22-17(23)10-2-1-4-19-8-10/h1-9,21H,(H,22,23) |
|  | PS-1145 | 21 | 0 | 0 | 0 | 1 | 0 | 0 | c12c(c3c(cncc3)[nH]1)cc(Cl)cc2NC(=O)c4cccnc4 | InChI=1/C17H11ClN4O/c18-11-6-13-12-3-5-20-9-15(12)21-16(13)14(7-11)22-17(23)10-2-1-4-19-8-10/h1-9,21H,(H,22,23) |
|  | beta Carboline 22 | 21 | 0 | 0 | 0 | 1 | 0 | 0 | c12c(ccnc1)c(cc(Cl)cc3NC(=O)CCCO)c3[nH]2 | InChI=1/C15H14ClN3O2/c16-9-6-11-10-3-4-17-8-13(10)19-15(11)12(7-9)18-14(21)2-1-5-20/h3-4,6-8,19-20H,1-2,5H2,(H,18,21) |
|  | beta-Carboline 6 | 21 | 0 | 0 | 0 | 1 | 0 | 0 | c12c(ccnc1)c(cc(Cl)cc3)c3[nH]2 | InChI=1/C11H7ClN2/c12-7-1-2-10-9(5-7)8-3-4-13-6-11(8)14-10/h1-6,14H |
|  | beta-Carboline 8 | 21 | 0 | 0 | 0 | 1 | 0 | 0 | c12c(ccnc1)c(cc(C#N)cc3)c3[nH]2 | InChI=1/C12H7N3/c13-6-8-1-2-11-10(5-8)9-3-4-14-7-12(9)15-11/h1-5,7,15H |
|  | beta-Carboline 3 | 21 | 0 | 0 | 0 | 1 | 0 | 0 | c12c(ccnc1)c(cc(OC)cc3)c3[nH]2 | InChI=1/C12H10N2O/c1-15-8-2-3-11-10(6-8)9-4-5-13-7-12(9)14-11/h2-7,14H,1H3 |
|  | 5-bromo-6-methoxy-b-carboline | 21 | 0 | 0 | 0 | 1 | 0 | 0 | c12c(ccnc1)c(c(Br)c(O)cc3)c3[nH]2 | InChI=1/C11H7BrN2O/c12-11-9(15)2-1-7-10(11)6-3-4-13-5-8(6)14-7/h1-5,14-15H |
|  | beta-Carboline 5 | 21 | 0 | 0 | 0 | 1 | 0 | 0 | c12c(ccnc1)c(cc(Br)cc3)c3[nH]2 | InChI=1/C11H7BrN2/c12-7-1-2-10-9(5-7)8-3-4-13-6-11(8)14-10/h1-6,14H |
|  | beta-Carboline 2 | 21 | 0 | 0 | 0 | 1 | 0 | 0 | c12c(ccnc1)c(cccc3)c3[nH]2 | InChI=1/C11H8N2/c1-2-4-10-8(3-1)9-5-6-12-7-11(9)13-10/h1-7,13H |
|  | beta-Carboline 7 | 21 | 0 | 0 | 0 | 1 | 0 | 0 | c12c(ccnc1)c(cc(C(F)(F)F)cc3)c3[nH]2 | InChI=1/C12H7F3N2/c13-12(14,15)7-1-2-10-9(5-7)8-3-4-16-6-11(8)17-10/h1-6,17H |
|  | beta Carboline 35 | 21 | 0 | 0 | 0 | 1 | 0 | 0 | c12c(ccnc1)c(cc(Cl)cc3NC(O)=N(CCOC4)C4)c3[nH]2 | InChI=1/C16H16ClN4O2/c17-10-7-12-11-1-2-18-9-14(11)19-15(12)13(8-10)20-16(22)21-3-5-23-6-4-21/h1-2,7-9,19-20,22H,3-6H2 |
|  | Benzoimidazole carboxamide | 23 | 0 | 0 | 0 | 1 | 0 | 0 | c1nccc(c2[nH]c(ccc(C(=O)N[C@@H](C(=O)N)Cc3ccccc3)c4)c4n2)c1 | InChI=1/C22H19N5O2/c23-20(28)19(12-14-4-2-1-3-5-14)27-22(29)16-6-7-17-18(13-16)26-21(25-17)15-8-10-24-11-9-15/h1-11,13,19H,12H2,(H2,23,28)(H,25,26)(H,27,29)/t19-/m1/s1 |
|  | Indolecarboxamide derivative | 23 | 0 | 0 | 0 | 1 | 0 | 0 | c1nccc(c2[nH]c(ccc(C(=O)N[C@@H](C(=O)N)CSc3ccccc3)c4)c4c2)c1 | InChI=1/C23H20N4O2S/c24-22(28)21(14-30-18-4-2-1-3-5-18)27-23(29)16-6-7-19-17(12-16)13-20(26-19)15-8-10-25-11-9-15/h1-13,21,26H,14H2,(H2,24,28)(H,27,29)/t21-/m1/s1 |
|  | 6cc | 25 | 0 | 0 | 0 | 0 | 0 | 0 | c1c(O)c(O)c(\C=C\c2ccc(OC)cc2)cc1 | InChI=1/C15H14O3/c1-18-13-9-6-11(7-10-13)5-8-12-3-2-4-14(16)15(12)17/h2-10,16-17H,1H3/b8-5+ |
|  | 6x | 25 | 0 | 0 | 0 | 0 | 0 | 0 | c1(c(O)cc(\C=C\c2ccc(OC)cc2)cc1)O | InChI=1/C15H14O3/c1-18-13-7-4-11(5-8-13)2-3-12-6-9-14(16)15(17)10-12/h2-10,16-17H,1H3/b3-2+ |
|  | 4ee | 25 | 0 | 0 | 0 | 0 | 0 | 0 | Oc1ccc(\C=C\c2ccccc2)cc1O | InChI=1/C14H12O2/c15-13-9-8-12(10-14(13)16)7-6-11-4-2-1-3-5-11/h1-10,15-16H/b7-6+ |
|  | 4q | 25 | 0 | 0 | 0 | 0 | 0 | 0 | c1(c(OC)cc(\C=C\c2ccccc2)cc1)O | InChI=1/C15H14O2/c1-17-15-11-13(9-10-14(15)16)8-7-12-5-3-2-4-6-12/h2-11,16H,1H3/b8-7+ |
|  | 4s | 25 | 0 | 0 | 0 | 0 | 0 | 0 | Oc1ccc(\C=C\c2ccccc2)cc1OC | InChI=1/C15H14O2/c1-17-15-11-13(9-10-14(15)16)8-7-12-5-3-2-4-6-12/h2-11,16H,1H3/b8-7+ |
|  | Resveratrol | 25 | 0 | 0 | 0 | 0 | 1 | 0 | C(=C\c1ccc(O)cc1)/c2cc(O)cc(O)c2 | InChI=1/C14H12O3/c15-12-5-3-10(4-6-12)1-2-11-7-13(16)9-14(17)8-11/h1-9,15-17H/b2-1+ |
|  | Compound 18 | 26 | 0 | 0 | 0 | 1 | 0 | 0 | c1(ccccc1O)c2cc(c3ccccc3C([O-])=O)c(C#N)c(N)n2.[Na] | InChI=1/C19H13N3O3.Na.H/c20-10-15-14(11-5-1-2-6-12(11)19(24)25)9-16(22-18(15)21)13-7-3-4-8-17(13)23;;/h1-9,23H,(H2,21,22)(H,24,25);;/p-1 |
|  | Compound 19 | 26 | 0 | 0 | 0 | 1 | 0 | 0 | c1(ccccc1O)c2cc(c3cccc(C([O-])=O)c3)c(C#N)c(N)n2.[Na] | InChI=1/C19H13N3O3.Na.H/c20-10-15-14(11-4-3-5-12(8-11)19(24)25)9-16(22-18(15)21)13-6-1-2-7-17(13)23;;/h1-9,23H,(H2,21,22)(H,24,25);;/p-1 |
|  | Compound 20 | 26 | 0 | 0 | 0 | 1 | 0 | 0 | c1(ccccc1O)c2cc(c3ccc(C([O-])=O)cc3)c(C#N)c(N)n2.[Na] | InChI=1/C19H13N3O3.Na.H/c20-10-15-14(11-5-7-12(8-6-11)19(24)25)9-16(22-18(15)21)13-3-1-2-4-17(13)23;;/h1-9,23H,(H2,21,22)(H,24,25);;/p-1 |
|  | Compound 2 | 26 | 0 | 0 | 0 | 1 | 0 | 0 | c1(C#N)c(N)nc(c2ccccc2O)cc1[C@@H](Cc3ccccc3)N | InChI=1/C20H18N4O/c21-12-16-15(17(22)10-13-6-2-1-3-7-13)11-18(24-20(16)23)14-8-4-5-9-19(14)25/h1-9,11,17,25H,10,22H2,(H2,23,24)/t17-/m1/s1 |
|  | Dimethyl sulfoxide | 28 | 0 | 0 | 0 | 0 | 0 | 0 | CS(=O)C | InChI=1/C2H6OS/c1-4(2)3/h1-2H3 |
|  | hydrogen peroxide | 28 | 0 | 1 | 0 | 0 | 0 | 0 | OO | InChI=1/H2O2/c1-2/h1-2H |
|  | Pyrithione | 28 | 0 | 0 | 0 | 0 | 0 | 0 | S=C1N(O)C=CC=C1 | InChI=1/C5H5NOS/c7-6-4-2-1-3-5(6)8/h1-4,7H |
|  | Mevastatin | 29 | 0 | 0 | 0 | 0 | 0 | 0 | [C@H]1(CC[C@@H]2[C@@H](C)C=CC([C@@H]2[C@@H](OC(=O)[C@@H](C)CC)C3)=CC3)C[C@@H](O)CC(=O)O1 | InChI=1/C23H34O5/c1-4-14(2)23(26)28-20-7-5-6-16-9-8-15(3)19(22(16)20)11-10-18-12-17(24)13-21(25)27-18/h6,8-9,14-15,17-20,22,24H,4-5,7,10-13H2,1-3H3/t14-,15-,17+,18-,19+,20-,22-/m0/s1 |
|  | lovastatin | 29 | 0 | 0 | 0 | 0 | 0 | 0 | [C@H]1(CC[C@@H]2[C@@H](C)C=CC([C@@H]2[C@@H](OC(=O)[C@@H](C)CC)C3)=C[C@@H]3C)C[C@@H](O)CC(=O)O1 | InChI=1/C24H36O5/c1-5-15(3)24(27)29-21-11-14(2)10-17-7-6-16(4)20(23(17)21)9-8-19-12-18(25)13-22(26)28-19/h6-7,10,14-16,18-21,23,25H,5,8-9,11-13H2,1-4H3/t14-,15-,16-,18+,19-,20+,21-,23-/m0/s1 |
|  | Simvastatin | 29 | 0 | 0 | 0 | 0 | 0 | 0 | [C@H]1(CC[C@@H]2[C@@H](C)C=CC([C@@H]2[C@@H](OC(=O)C(C)(C)CC)C3)=C[C@@H]3C)C[C@@H](O)CC(=O)O1 | InChI=1/C25H38O5/c1-6-25(4,5)24(28)30-21-12-15(2)11-17-8-7-16(3)20(23(17)21)10-9-19-13-18(26)14-22(27)29-19/h7-8,11,15-16,18-21,23,26H,6,9-10,12-14H2,1-5H3/t15-,16-,18+,19-,20+,21-,23-/m0/s1 |
|  | Wedelolactone | 30 | 0 | 0 | 0 | 1 | 0 | 0 | c12c([C@H]3[C@@H](c(cc(O)c(O)c4)c4O3)C(O1)=O)c(O)cc(O)c2 | InChI=1/C15H10O7/c16-5-1-9(19)13-11(2-5)22-15(20)12-6-3-7(17)8(18)4-10(6)21-14(12)13/h1-4,12,14,16-19H/t12-,14-/m1/s1 |
|  | quercetin | 30 | 0 | 0 | 0 | 1 | 0 | 0 | O=C1C(O)=C(c2cc(O)c(O)cc2)Oc(cc(cc3O)O)c13 | InChI=1/C15H10O7/c16-7-4-10(19)12-11(5-7)22-15(14(21)13(12)20)6-1-2-8(17)9(18)3-6/h1-5,16-19,21H |
|  | alpha Tocopherol | 34 | 0 | 0 | 0 | 0 | 0 | 0 | c1(C(O)=O)c(C)c(CC[C@@](CCC[C@@H](CCC[C@H](CCCC(C)C)C)C)(C)O2)c2c(C)c1C | InChI=1/C30H50O3/c1-20(2)12-9-13-21(3)14-10-15-22(4)16-11-18-30(8)19-17-26-25(7)27(29(31)32)23(5)24(6)28(26)33-30/h20-22H,9-19H2,1-8H3,(H,31,32)/t21-,22+,30-/m0/s1 |
|  | vitamin E | 34 | 0 | 1 | 0 | 0 | 0 | 0 | C(C[C@](Oc(c(C)c1C)c2c(C)c1O)(C)CC2)C[C@@H](CCC[C@@H](CCCC(C)C)C)C | InChI=1/C29H50O2/c1-20(2)12-9-13-21(3)14-10-15-22(4)16-11-18-29(8)19-17-26-25(7)27(30)23(5)24(6)28(26)31-29/h20-22,30H,9-19H2,1-8H3/t21-,22-,29-/m1/s1 |
|  | EPC-k1 | 34 | 0 | 0 | 0 | 0 | 0 | 0 | Cc1c(O[C@](CCC[C@H](CCC[C@H](CCCC(C)C)C)C)(C)CC2)c2c(C)c(OP(OC3=C(C(OC3=O)[C@H](CO)O)O)([O-])=O)c1.[K+] | InChI=1/C34H55O10P.K/c1-21(2)11-8-12-22(3)13-9-14-23(4)15-10-17-34(7)18-16-26-25(6)28(19-24(5)30(26)42-34)43-45(39,40)44-32-29(37)31(27(36)20-35)41-33(32)38;/h19,21-23,27,31,35-37H,8-18,20H2,1-7H3,(H,39,40);/q;+1/p-1/t22-,23-,27-,31?,34-;/m0./s1 |
|  | Carnosol | 36 | 0 | 0 | 0 | 0 | 1 | 0 | O=C([C@]1([C@H](C2)C(C)(C)CCC1)c(c(O)c(O)c(C(C)C)c3)c34)O[C@H]24 | InChI=1/C20H26O4/c1-10(2)11-8-12-13-9-14-19(3,4)6-5-7-20(14,18(23)24-13)15(12)17(22)16(11)21/h8,10,13-14,21-22H,5-7,9H2,1-4H3/t13-,14-,20-/m1/s1 |
|  | Carnosic acid | 36 | 0 | 0 | 0 | 0 | 0 | 0 | CC(c1c(O)c(O)c([C@](C(O)=O)(CCCC2(C)C)[C@H]2CC3)c3c1)C | InChI=1/C20H28O4/c1-11(2)13-10-12-6-7-14-19(3,4)8-5-9-20(14,18(23)24)15(12)17(22)16(13)21/h10-11,14,21-22H,5-9H2,1-4H3,(H,23,24)/t14-,20-/m0/s1 |
|  | Epoxyquinol A | 38 | 0 | 0 | 0 | 0 | 0 | 0 | [C@]1([C@H](O[C@H]2C)C([C@H](O)[C@@H](O3)[C@@H]3C4=O)=C45)([C@H]([C@H]25)[C@@H](C)O6)C([C@H](O)[C@@H](O7)[C@@H]7C1=O)=C6 | InChI=1/C20H20O8/c1-4-7-8-9(13(23)16-15(27-16)12(8)22)19(26-4)20-6(3-25-5(2)10(7)20)11(21)14-17(28-14)18(20)24/h3-5,7,10-11,13-17,19,21,23H,1-2H3/t4-,5+,7+,10-,11-,13-,14+,15-,16+,17+,19+,20+/m0/s1 |
|  | quinone dimer (9) | 38 | 0 | 0 | 0 | 0 | 0 | 0 | [C@]1([C@H](O[C@H]2C)C(C(=O)[C@@H](O3)[C@@H]3C4=O)=C45)([C@H]([C@H]25)[C@@H](C)O6)C(C(=O)[C@@H](O7)[C@@H]7C1=O)=C6 | InChI=1/C20H16O8/c1-4-7-8-9(13(23)16-15(27-16)12(8)22)19(26-4)20-6(3-25-5(2)10(7)20)11(21)14-17(28-14)18(20)24/h3-5,7,10,14-17,19H,1-2H3/t4-,5+,7+,10-,14+,15-,16+,17+,19+,20+/m0/s1 |
|  | fern-9(11)ene | 39 | 0 | 0 | 0 | 0 | 1 | 0 | CC([C@@H]1[C@](CC[C@]2([C@@]3(C)CC=C([C@@](C)(CCCC4(C)C)[C@@H]4CC5)[C@@H]25)C)(C)[C@@H]3CC1)C | InChI=1/C30H50/c1-20(2)21-10-13-25-28(21,6)18-19-29(7)23-11-12-24-26(3,4)15-9-16-27(24,5)22(23)14-17-30(25,29)8/h14,20-21,23-25H,9-13,15-19H2,1-8H3/t21-,23+,24-,25+,27-,28-,29-,30+/m1/s1 |
|  | dryocrassol | 39 | 0 | 0 | 0 | 0 | 1 | 0 | C([C@H]([C@@H]1[C@]2(C)C(=C(CC[C@H]([C@@](C)(CCCC3(C)C)[C@@H]3CC4)[C@@]45C)[C@@]5(C)CC2)CC1)C)O | InChI=1/C30H50O/c1-20(19-31)21-9-10-22-23-11-12-25-28(5)15-8-14-26(2,3)24(28)13-16-30(25,7)29(23,6)18-17-27(21,22)4/h20-21,24-25,31H,8-19H2,1-7H3/t20-,21-,24-,25-,27-,28+,29-,30-/m1/s1 |
|  | CAPE analogue 2 | 42 | 1 | 0 | 0 | 0 | 0 | 0 | c12c(cc(c(c1)OC)OC)cc(\C=C\C(=O)OCCc3ccccc3)cc2 | InChI=1/C23H22O4/c1-25-21-15-19-10-8-18(14-20(19)16-22(21)26-2)9-11-23(24)27-13-12-17-6-4-3-5-7-17/h3-11,14-16H,12-13H2,1-2H3/b11-9+ |
|  | 30d9 | 42 | 0 | 0 | 0 | 0 | 0 | 0 | c12c(nc3c(cccc3)c1N[C@@H](C)CCCN(CC)CC)cccc2 | InChI=1/C22H29N3/c1-4-25(5-2)16-10-11-17(3)23-22-18-12-6-8-14-20(18)24-21-15-9-7-13-19(21)22/h6-9,12-15,17H,4-5,10-11,16H2,1-3H3,(H,23,24)/t17-/m0/s1 |
|  | 8e | 43 | 0 | 0 | 0 | 0 | 0 | 0 | C(=C\c1ccccc1)/c2ccc(cccc3)c3c2 | InChI=1/C18H14/c1-2-6-15(7-3-1)10-11-16-12-13-17-8-4-5-9-18(17)14-16/h1-14H/b11-10+ |
|  | 8g | 43 | 0 | 0 | 0 | 0 | 0 | 0 | C(=C\c1ccc(OC)cc1)/c2cc(cccc3)c3cc2 | InChI=1/C19H16O/c1-20-19-12-9-15(10-13-19)6-7-16-8-11-17-4-2-3-5-18(17)14-16/h2-14H,1H3/b7-6+ |
|  | 8f | 43 | 0 | 0 | 0 | 0 | 0 | 0 | C(=C\c1ccccc1)/c2cccc(cccc3)c23 | InChI=1/C18H14/c1-2-7-15(8-3-1)13-14-17-11-6-10-16-9-4-5-12-18(16)17/h1-14H/b14-13+ |
|  | 8h | 43 | 0 | 0 | 0 | 0 | 0 | 0 | C(=C\c1ccc(OC)cc1)/c2c(cccc3)c3ccc2 | InChI=1/C19H16O/c1-20-18-13-10-15(11-14-18)9-12-17-7-4-6-16-5-2-3-8-19(16)17/h2-14H,1H3/b12-9+ |
|  | 4h | 43 | 0 | 0 | 0 | 0 | 0 | 0 | c1c(Cl)cc(\C=C\c2ccccc2)cc1 | InChI=1/C14H11Cl/c15-14-8-4-7-13(11-14)10-9-12-5-2-1-3-6-12/h1-11H/b10-9+ |
|  | 4p | 43 | 0 | 0 | 0 | 0 | 0 | 0 | c1cc(F)c(\C=C\c2ccccc2)cc1 | InChI=1/C14H11F/c15-14-9-5-4-8-13(14)11-10-12-6-2-1-3-7-12/h1-11H/b11-10+ |
|  | 4c | 43 | 0 | 0 | 0 | 0 | 0 | 0 | c1(ccc(\C=C\c2ccccc2)cc1)Cl | InChI=1/C14H11Cl/c15-14-10-8-13(9-11-14)7-6-12-4-2-1-3-5-12/h1-11H/b7-6+ |
|  | 4i | 43 | 0 | 0 | 0 | 0 | 0 | 0 | c1c(C)cc(\C=C\c2ccccc2)cc1 | InChI=1/C15H14/c1-13-6-5-9-15(12-13)11-10-14-7-3-2-4-8-14/h2-12H,1H3/b11-10+ |
|  | 4d | 43 | 0 | 0 | 0 | 0 | 0 | 0 | c1(ccc(\C=C\c2ccccc2)cc1)C | InChI=1/C15H14/c1-13-7-9-15(10-8-13)12-11-14-5-3-2-4-6-14/h2-12H,1H3/b12-11+ |
|  | 4n | 43 | 0 | 0 | 0 | 0 | 0 | 0 | c1c(F)cc(\C=C\c2ccccc2)cc1 | InChI=1/C14H11F/c15-14-8-4-7-13(11-14)10-9-12-5-2-1-3-6-12/h1-11H/b10-9+ |
|  | 4m | 43 | 0 | 0 | 0 | 0 | 0 | 0 | c1(ccc(\C=C\c2ccccc2)cc1)F | InChI=1/C14H11F/c15-14-10-8-13(9-11-14)7-6-12-4-2-1-3-5-12/h1-11H/b7-6+ |
|  | 4w | 43 | 0 | 0 | 0 | 0 | 0 | 0 | c1c(CF)cc(\C=C\c2ccccc2)cc1 | InChI=1/C15H13F/c16-12-15-8-4-7-14(11-15)10-9-13-5-2-1-3-6-13/h1-11H,12H2/b10-9+ |
|  | 4aa | 43 | 0 | 0 | 0 | 0 | 0 | 0 | c1(cc(C)c(\C=C\c2ccccc2)c(C)c1)C | InChI=1/C17H18/c1-13-11-14(2)17(15(3)12-13)10-9-16-7-5-4-6-8-16/h4-12H,1-3H3/b10-9+ |
|  | 4v | 43 | 0 | 0 | 0 | 0 | 0 | 0 | c1cc(C)c(\C=C\c2ccccc2)cc1 | InChI=1/C15H14/c1-13-7-5-6-10-15(13)12-11-14-8-3-2-4-9-14/h2-12H,1H3/b12-11+ |
|  | 4x | 43 | 0 | 0 | 0 | 0 | 0 | 0 | c1(ccc(\C=C\c2ccccc2)cc1)CF | InChI=1/C15H13F/c16-12-15-10-8-14(9-11-15)7-6-13-4-2-1-3-5-13/h1-11H,12H2/b7-6+ |
|  | 4t | 43 | 0 | 0 | 0 | 0 | 0 | 0 | c1c(O)cc(\C=C\c2ccccc2)cc1 | InChI=1/C14H12O/c15-14-8-4-7-13(11-14)10-9-12-5-2-1-3-6-12/h1-11,15H/b10-9+ |
|  | 4e | 43 | 0 | 0 | 0 | 0 | 0 | 0 | c1(C#N)ccc(\C=C\c2ccccc2)cc1 | InChI=1/C15H11N/c16-12-15-10-8-14(9-11-15)7-6-13-4-2-1-3-5-13/h1-11H/b7-6+ |
|  | 4l | 43 | 0 | 0 | 0 | 0 | 0 | 0 | c1(ccc(\C=C\c2ccccc2)cc1)O | InChI=1/C14H12O/c15-14-10-8-13(9-11-14)7-6-12-4-2-1-3-5-12/h1-11,15H/b7-6+ |
|  | E-Capsiate | 49 | 0 | 0 | 0 | 0 | 0 | 0 | c1c(OC)c(O)ccc1COC(=O)CCCC\C=C\C(C)C | InChI=1/C18H26O4/c1-14(2)8-6-4-5-7-9-18(20)22-13-15-10-11-16(19)17(12-15)21-3/h6,8,10-12,14,19H,4-5,7,9,13H2,1-3H3/b8-6+ |
|  | Z-Capsiate | 49 | 0 | 0 | 0 | 0 | 0 | 0 | c1c(OC)c(O)ccc1COC(=O)CCCC\C=C/C(C)C | InChI=1/C18H26O4/c1-14(2)8-6-4-5-7-9-18(20)22-13-15-10-11-16(19)17(12-15)21-3/h6,8,10-12,14,19H,4-5,7,9,13H2,1-3H3/b8-6- |
|  | Dihydrocapsiate | 49 | 0 | 0 | 0 | 0 | 0 | 0 | c1c(OC)c(O)ccc1COC(=O)CCCCCCC(C)C | InChI=1/C18H28O4/c1-14(2)8-6-4-5-7-9-18(20)22-13-15-10-11-16(19)17(12-15)21-3/h10-12,14,19H,4-9,13H2,1-3H3 |
|  | Compound 24 | 51 | 0 | 0 | 0 | 1 | 0 | 0 | c1(ccccc1O)c2cc(c3cccc(NC(=O)[C@H](CCC(O)=O)N)c3)c(C#N)c(N)n2 | InChI=1/C23H21N5O4/c24-12-17-16(11-19(28-22(17)26)15-6-1-2-7-20(15)29)13-4-3-5-14(10-13)27-23(32)18(25)8-9-21(30)31/h1-7,10-11,18,29H,8-9,25H2,(H2,26,28)(H,27,32)(H,30,31)/t18-/m0/s1 |
|  | Compound 25 | 51 | 0 | 0 | 0 | 1 | 0 | 0 | c1(ccccc1O)c2cc(c3cccc(NC(=O)C[C@@H](N)CC(O)=O)c3)c(C#N)c(N)n2 | InChI=1/C23H21N5O4/c24-12-18-17(11-19(28-23(18)26)16-6-1-2-7-20(16)29)13-4-3-5-15(8-13)27-21(30)9-14(25)10-22(31)32/h1-8,11,14,29H,9-10,25H2,(H2,26,28)(H,27,30)(H,31,32)/t14-/m1/s1 |
|  | Compound 6 | 51 | 0 | 0 | 0 | 1 | 0 | 0 | c1(ccccc1O)c2cc(c3cccc(NC(=O)[C@@H](O)CCC([O-])=O)c3)c(C#N)c(N)n2.[Na+] | InChI=1/C23H20N4O5.Na/c24-12-17-16(11-18(27-22(17)25)15-6-1-2-7-19(15)28)13-4-3-5-14(10-13)26-23(32)20(29)8-9-21(30)31;/h1-7,10-11,20,28-29H,8-9H2,(H2,25,27)(H,26,32)(H,30,31);/q;+1/p-1/t20-;/m0./s1 |
|  | Compound 36 | 51 | 0 | 0 | 0 | 1 | 0 | 0 | c1(cc(OC)ccc1O)c2cc(c3cccc(NC(=O)[C@@H](O)CCC([O-])=O)c3)c(C#N)c(N)n2.[Na+] | InChI=1/C24H22N4O6.Na/c1-34-15-5-6-20(29)17(10-15)19-11-16(18(12-25)23(26)28-19)13-3-2-4-14(9-13)27-24(33)21(30)7-8-22(31)32;/h2-6,9-11,21,29-30H,7-8H2,1H3,(H2,26,28)(H,27,33)(H,31,32);/q;+1/p-1/t21-;/m0./s1 |
|  | Compound 38 | 51 | 0 | 0 | 0 | 1 | 0 | 0 | c1(c(OC)cccc1O)c2cc(c3cccc(NC(=O)[C@@H](O)CCC([O-])=O)c3)c(C#N)c(N)n2.[Na+] | InChI=1/C24H22N4O6.Na/c1-34-20-7-3-6-18(29)22(20)17-11-15(16(12-25)23(26)28-17)13-4-2-5-14(10-13)27-24(33)19(30)8-9-21(31)32;/h2-7,10-11,19,29-30H,8-9H2,1H3,(H2,26,28)(H,27,33)(H,31,32);/q;+1/p-1/t19-;/m0./s1 |
|  | Compound 35 | 51 | 0 | 0 | 0 | 1 | 0 | 0 | c1(cc(Cl)ccc1O)c2cc(c3cccc(NC(=O)[C@@H](O)CCC([O-])=O)c3)c(C#N)c(N)n2.[Na+] | InChI=1/C23H19ClN4O5.Na/c24-13-4-5-19(29)16(9-13)18-10-15(17(11-25)22(26)28-18)12-2-1-3-14(8-12)27-23(33)20(30)6-7-21(31)32;/h1-5,8-10,20,29-30H,6-7H2,(H2,26,28)(H,27,33)(H,31,32);/q;+1/p-1/t20-;/m0./s1 |
|  | Hypericin | 57 | 0 | 0 | 0 | 0 | 0 | 0 | O=C1c(c(O)cc2O)c(c23)c(c(c(c4c(C)cc5O)c5C6=O)c(c6c(O)cc7O)c37)c(c4c(C)cc8O)c18 | InChI=1/C30H16O8/c1-7-3-9(31)19-23-15(7)16-8(2)4-10(32)20-24(16)28-26-18(12(34)6-14(36)22(26)30(20)38)17-11(33)5-13(35)21(29(19)37)25(17)27(23)28/h3-6,31-36H,1-2H3 |
|  | selligueain | 57 | 0 | 0 | 0 | 0 | 1 | 0 | Oc1ccc([C@H]2Oc(c([C@H]3[C@@H](O)[C@@]4(c5ccc(O)cc5)Oc(cc(O)cc6O)c36)c4cc7)c7[C@H](c8c(O[C@H](c9ccc(O)cc9)[C@H](O)C%10)c%10c(O)cc8O)C2)cc1 | InChI=1/C45H36O12/c46-23-7-1-20(2-8-23)35-18-28(37-33(52)19-31(50)29-17-34(53)41(56-43(29)37)21-3-9-24(47)10-4-21)27-13-14-30-38(42(27)55-35)40-39-32(51)15-26(49)16-36(39)57-45(30,44(40)54)22-5-11-25(48)12-6-22/h1-16,19,28,34-35,40-41,44,46-54H,17-18H2/t28-,34-,35+,40-,41-,44-,45+/m1/s1 |
|  | Compound 3l | 60 | 0 | 0 | 0 | 1 | 0 | 0 | c1(C#N)c(N)nc(c2c(OCCCCCCC)cccc2O)cc1[C@@H]3CCCNC3 | InChI=1/C24H32N4O2/c1-2-3-4-5-6-13-30-22-11-7-10-21(29)23(22)20-14-18(17-9-8-12-27-16-17)19(15-25)24(26)28-20/h7,10-11,14,17,27,29H,2-6,8-9,12-13,16H2,1H3,(H2,26,28)/t17-/m1/s1 |
|  | Compound 4h | 60 | 0 | 0 | 0 | 1 | 0 | 0 | c1(C#N)c(N)nc(c2c(O)cccc2OCCCCCCC)cc1C3CCNCC3 | InChI=1/C24H32N4O2/c1-2-3-4-5-6-14-30-22-9-7-8-21(29)23(22)20-15-18(17-10-12-27-13-11-17)19(16-25)24(26)28-20/h7-9,15,17,27,29H,2-6,10-14H2,1H3,(H2,26,28) |
|  | Compound 4g | 60 | 0 | 0 | 0 | 1 | 0 | 0 | c1(C#N)c(N)nc(c2c(O)cccc2OCCCCCC)cc1C3CCNCC3 | InChI=1/C23H30N4O2/c1-2-3-4-5-13-29-21-8-6-7-20(28)22(21)19-14-17(16-9-11-26-12-10-16)18(15-24)23(25)27-19/h6-8,14,16,26,28H,2-5,9-13H2,1H3,(H2,25,27) |
|  | Compound 3k | 60 | 0 | 0 | 0 | 1 | 0 | 0 | c1(C#N)c(N)nc(c2c(OCCCCC)cccc2O)cc1[C@@H]3CCCNC3 | InChI=1/C22H28N4O2/c1-2-3-4-11-28-20-9-5-8-19(27)21(20)18-12-16(15-7-6-10-25-14-15)17(13-23)22(24)26-18/h5,8-9,12,15,25,27H,2-4,6-7,10-11,14H2,1H3,(H2,24,26)/t15-/m1/s1 |
|  | Compound 4f | 60 | 0 | 0 | 0 | 1 | 0 | 0 | c1(C#N)c(N)nc(c2c(O)cccc2OCCCCC)cc1C3CCNCC3 | InChI=1/C22H28N4O2/c1-2-3-4-12-28-20-7-5-6-19(27)21(20)18-13-16(15-8-10-25-11-9-15)17(14-23)22(24)26-18/h5-7,13,15,25,27H,2-4,8-12H2,1H3,(H2,24,26) |
|  | Compound 3q | 60 | 0 | 0 | 0 | 1 | 0 | 0 | c1(C#N)c(N)nc(c2c(OCC3CCCCC3)cccc2O)cc1[C@@H]4CCCNC4 | InChI=1/C24H30N4O2/c25-13-19-18(17-8-5-11-27-14-17)12-20(28-24(19)26)23-21(29)9-4-10-22(23)30-15-16-6-2-1-3-7-16/h4,9-10,12,16-17,27,29H,1-3,5-8,11,14-15H2,(H2,26,28)/t17-/m1/s1 |
|  | Erbstatin | 62 | 0 | 0 | 0 | 0 | 1 | 0 | c1(cc(O)ccc1O)\C=C\C(OC)=O | InChI=1/C10H10O4/c1-14-10(13)5-2-7-6-8(11)3-4-9(7)12/h2-6,11-12H,1H3/b5-2+ |
|  | Aspirin | 62 | 0 | 0 | 0 | 0 | 1 | 0 | CC(Oc1c(C(O)=O)cccc1)=O | InChI=1/C9H8O4/c1-6(10)13-8-5-3-2-4-7(8)9(11)12/h2-5H,1H3,(H,11,12) |
|  | Dimethyl maleate | 62 | 0 | 0 | 0 | 0 | 0 | 0 | O=C(\C=C/C(OC)=O)OC | InChI=1/C6H8O4/c1-9-5(7)3-4-6(8)10-2/h3-4H,1-2H3/b4-3- |
|  | 194 | 63 | 1 | 0 | 0 | 0 | 0 | 0 | [C@@H]1(\C(=C\C=C=CC[C@@H]([NH3+])CC)\C(=O)C=C1)C\C=C\CCCC(O)=O | InChI=1/C20H27NO3/c1-2-17(21)11-7-5-8-12-18-16(14-15-19(18)22)10-6-3-4-9-13-20(23)24/h3,6-8,12,14-17H,2,4,9-11,13,21H2,1H3,(H,23,24)/p+1/b6-3+,18-12-/t5?,16-,17+/m1/s1 |
|  | 189 | 63 | 1 | 0 | 0 | 0 | 0 | 0 | [C@@H]1(\C(=C/C=C/CC[C@@H](O)CC)\C(=O)C=C1)C\C=C\CCCC(O)=O | InChI=1/C20H28O4/c1-2-17(21)11-7-5-8-12-18-16(14-15-19(18)22)10-6-3-4-9-13-20(23)24/h3,5-6,8,12,14-17,21H,2,4,7,9-11,13H2,1H3,(H,23,24)/b6-3+,8-5+,18-12+/t16-,17+/m1/s1 |
|  | 190 | 63 | 1 | 0 | 0 | 0 | 0 | 0 | [C@H]1(C\C=C\CCCC(O)=O)\C(=C/C=C/CCC[C@@H](C(N)=O)C)\C(=O)C=C1 | InChI=1/C21H29NO4/c1-16(21(22)26)10-6-2-4-8-12-18-17(14-15-19(18)23)11-7-3-5-9-13-20(24)25/h3-4,7-8,12,14-17H,2,5-6,9-11,13H2,1H3,(H2,22,26)(H,24,25)/b7-3+,8-4+,18-12+/t16-,17+/m0/s1 |
|  | 193 | 63 | 1 | 0 | 0 | 0 | 0 | 0 | [C@H]1(C\C=C\CCCC(O)=O)\C(=C/C=C/CCCCCN)\C(=O)C=C1 | InChI=1/C20H29NO3/c21-16-10-6-2-1-3-8-12-18-17(14-15-19(18)22)11-7-4-5-9-13-20(23)24/h3-4,7-8,12,14-15,17H,1-2,5-6,9-11,13,16,21H2,(H,23,24)/b7-4+,8-3+,18-12+/t17-/m1/s1 |
|  | 199 | 63 | 1 | 0 | 0 | 0 | 0 | 0 | [C@@H]1(\C(=C\C=C=C[C@@H](C)[C@@H]([NH3+])CC)\C(=O)C=C1S)C\C=C\CCCC(O)=O | InChI=1/C21H29NO3S/c1-3-18(22)15(2)10-8-9-11-16-17(20(26)14-19(16)23)12-6-4-5-7-13-21(24)25/h4,6,9-11,14-15,17-18,26H,3,5,7,12-13,22H2,1-2H3,(H,24,25)/p+1/b6-4+,16-11-/t8?,15-,17+,18+/m1/s1 |
|  | 184 | 63 | 1 | 0 | 0 | 0 | 0 | 0 | [C@@H]1(\C(=C/C=C/CC[C@@H](F)CC)\C(=O)C=C1)C\C=C\CCCC(O)=O | InChI=1/C20H27FO3/c1-2-17(21)11-7-5-8-12-18-16(14-15-19(18)22)10-6-3-4-9-13-20(23)24/h3,5-6,8,12,14-17H,2,4,7,9-11,13H2,1H3,(H,23,24)/b6-3+,8-5+,18-12+/t16-,17+/m1/s1 |
|  | 182 | 63 | 1 | 0 | 0 | 0 | 0 | 0 | [C@@H]1(\C(=C/C=C/CC[C@@H](Cl)CC)\C(=O)C=C1)C\C=C\CCCC(O)=O | InChI=1/C20H27ClO3/c1-2-17(21)11-7-5-8-12-18-16(14-15-19(18)22)10-6-3-4-9-13-20(23)24/h3,5-6,8,12,14-17H,2,4,7,9-11,13H2,1H3,(H,23,24)/b6-3+,8-5+,18-12+/t16-,17+/m1/s1 |
|  | 192 | 63 | 1 | 0 | 0 | 0 | 0 | 0 | [C@@H]1(\C(=C/C=C/CCCCC)\C(=O)C=C1)C\C=C\CCCC(O)=O | InChI=1/C20H28O3/c1-2-3-4-5-6-10-13-18-17(15-16-19(18)21)12-9-7-8-11-14-20(22)23/h6-7,9-10,13,15-17H,2-5,8,11-12,14H2,1H3,(H,22,23)/b9-7+,10-6+,18-13+/t17-/m1/s1 |
|  | Parthenolide | 64 | 0 | 0 | 0 | 0 | 1 | 0 | C=C1[C@@H](CC\C(=C/CC[C@@]2([C@@H]3O2)C)\C)[C@@H]3OC1=O | InChI=1/C15H20O3/c1-9-5-4-8-15(3)13(18-15)12-11(7-6-9)10(2)14(16)17-12/h5,11-13H,2,4,6-8H2,1,3H3/b9-5-/t11-,12+,13-,15-/m1/s1 |
|  | Isohelenin | 64 | 0 | 0 | 0 | 0 | 1 | 0 | C=C1[C@H](C[C@@H](C(=C)C(=O)O2)[C@@H]2C3)[C@]3(C)CCC1 | InChI=1/C15H20O2/c1-9-5-4-6-15(3)8-13-11(7-12(9)15)10(2)14(16)17-13/h11-13H,1-2,4-8H2,3H3/t11-,12-,13-,15+/m0/s1 |
|  | Pimobendan | 68 | 0 | 0 | 0 | 0 | 0 | 0 | O[C@@H]([C@H](CO)[N+](C)(C)C)\C=C\CCC | InChI=1/C11H24NO2/c1-5-6-7-8-11(14)10(9-13)12(2,3)4/h7-8,10-11,13-14H,5-6,9H2,1-4H3/q+1/b8-7+/t10-,11+/m0/s1 |
|  | N,N,N-Trimethylsphingosine | 68 | 0 | 1 | 0 | 0 | 0 | 0 | O[C@@H]([C@H](CO)[N+](C)(C)C)\C=C\CCC | InChI=1/C11H24NO2/c1-5-6-7-8-11(14)10(9-13)12(2,3)4/h7-8,10-11,13-14H,5-6,9H2,1-4H3/q+1/b8-7+/t10-,11+/m0/s1 |
|  | DCB-3502 | 69 | 0 | 0 | 0 | 0 | 0 | 0 | C1(=C[C@H](CCC2)N2C3)[C@H]3c(cc(OC)c(OC)c4)c4c(cc(OC)c(OC)c5)c15 | InChI=1/C24H27NO4/c1-26-21-9-16-15-8-14-6-5-7-25(14)13-20(15)19-12-24(29-4)23(28-3)11-18(19)17(16)10-22(21)27-2/h8-12,14,20H,5-7,13H2,1-4H3/t14-,20-/m0/s1 |
|  | DCB-3500 | 69 | 0 | 0 | 0 | 0 | 0 | 0 | c12c(CN3[C@@H](CCC3)C1)c(cc(OC)c(OC)c4)c4c(cc(OC)c(OC)c5)c25 | InChI=1/C24H27NO4/c1-26-21-9-16-15-8-14-6-5-7-25(14)13-20(15)19-12-24(29-4)23(28-3)11-18(19)17(16)10-22(21)27-2/h9-12,14H,5-8,13H2,1-4H3/t14-/m0/s1 |
|  | Emodin | 70 | 0 | 0 | 0 | 0 | 1 | 0 | Cc1cc(C(=O)c(cc(O)cc2O)c2C3=O)c3c(O)c1 | InChI=1/C15H10O5/c1-6-2-8-12(10(17)3-6)15(20)13-9(14(8)19)4-7(16)5-11(13)18/h2-5,16-18H,1H3 |
|  | Apigenin | 70 | 0 | 0 | 0 | 0 | 0 | 0 | O=C1c(c(O)cc(O)c2)c2OC(c3ccc(O)cc3)=C1 | InChI=1/C15H10O5/c16-9-3-1-8(2-4-9)13-7-12(19)15-11(18)5-10(17)6-14(15)20-13/h1-7,16-18H |
|  | Baicalein | 70 | 0 | 0 | 0 | 0 | 0 | 1 | O=C1C=C(c2ccccc2)Oc(cc(O)c(O)c3O)c13 | InChI=1/C15H10O5/c16-9-6-11(8-4-2-1-3-5-8)20-12-7-10(17)14(18)15(19)13(9)12/h1-7,17-19H |
|  | hesperetin | 70 | 0 | 0 | 0 | 0 | 1 | 0 | O=C1C[C@@H](c2cc(O)c(OC)cc2)Oc(cc(cc3O)O)c13 | InChI=1/C16H14O6/c1-21-13-3-2-8(4-10(13)18)14-7-12(20)16-11(19)5-9(17)6-15(16)22-14/h2-6,14,17-19H,7H2,1H3/t14-/m0/s1 |
|  | 3,4,3',5'-tetrahydroxy-trans-stilbene | 70 | 0 | 0 | 0 | 0 | 1 | 0 | c1(cc(O)cc(O)c1)\C=C\c2cc(O)cc(O)c2 | InChI=1/C14H12O4/c15-11-3-9(4-12(16)7-11)1-2-10-5-13(17)8-14(18)6-10/h1-8,15-18H/b2-1+ |
|  | Lacidipine | 71 | 0 | 0 | 0 | 0 | 0 | 0 | CCOC(C1=C(NC(=C(C1c2c(\C=C/C(OC(C)(C)C)=O)cccc2)C(OCC)=O)C)C)=O | InChI=1/C26H33NO6/c1-8-31-24(29)21-16(3)27-17(4)22(25(30)32-9-2)23(21)19-13-11-10-12-18(19)14-15-20(28)33-26(5,6)7/h10-15,23,27H,8-9H2,1-7H3/b15-14- |
|  | nifedipine | 71 | 0 | 0 | 0 | 0 | 0 | 0 | O=C(C1=C(C)NC(C)=C(C1c2c([N+](=O)[O-])cccc2)C(OC)=O)OC | InChI=1/C17H18N2O6/c1-9-13(16(20)24-3)15(14(10(2)18-9)17(21)25-4)11-7-5-6-8-12(11)19(22)23/h5-8,15,18H,1-4H3 |
|  | beta Carboline 14 | 72 | 0 | 0 | 0 | 1 | 0 | 0 | c12c(ccnc1)c(cc(Cl)c(OC(=O)N3CCOCC3)c4Cl)c4[nH]2 | InChI=1/C16H13Cl2N3O3/c17-11-7-10-9-1-2-19-8-12(9)20-14(10)13(18)15(11)24-16(22)21-3-5-23-6-4-21/h1-2,7-8,20H,3-6H2 |
|  | Anilino-pyrimidine | 72 | 0 | 0 | 0 | 1 | 0 | 0 | c1c(c2nc(Nc3ccc(C(=O)N4CCOCC4)cc3)ncc2)ccc(Cl)c1 | InChI=1/C21H19ClN4O2/c22-17-5-1-15(2-6-17)19-9-10-23-21(25-19)24-18-7-3-16(4-8-18)20(27)26-11-13-28-14-12-26/h1-10H,11-14H2,(H,23,24,25) |
|  | Nicotinamide | 74 | 0 | 0 | 0 | 0 | 0 | 0 | O=C(c1cnccc1)N | InChI=1/C6H6N2O/c7-6(9)5-2-1-3-8-4-5/h1-4H,(H2,7,9) |
|  | 3-aminobenzamide | 74 | 0 | 0 | 0 | 0 | 0 | 0 | O=C(N)c1cccc(N)c1 | InChI=1/C7H8N2O/c8-6-3-1-2-5(4-6)7(9)10/h1-4H,8H2,(H2,9,10) |
|  | Sodium salicylate | 74 | 0 | 0 | 0 | 0 | 1 | 0 | [Na+].O=C(c1c(O)cccc1)[O-] | InChI=1/C7H6O3.Na/c8-6-4-2-1-3-5(6)7(9)10;/h1-4,8H,(H,9,10);/q;+1/p-1 |
|  | nicotine | 74 | 0 | 0 | 0 | 0 | 1 | 0 | n1cccc([C@@H]2CCCN2C)c1 | InChI=1/C10H14N2/c1-12-7-3-5-10(12)9-4-2-6-11-8-9/h2,4,6,8,10H,3,5,7H2,1H3/t10-/m0/s1 |
|  | thiophenecarboxamide\_baxter\_2 | 74 | 0 | 0 | 0 | 1 | 0 | 0 | NC(Nc1c(C(=O)N)scc1)=O | InChI=1/C6H7N3O2S/c7-5(10)4-3(1-2-12-4)9-6(8)11/h1-2H,(H2,7,10)(H3,8,9,11) |
|  | Amrinone | 74 | 0 | 0 | 0 | 0 | 0 | 0 | O=C1NC=C(c2ccncc2)C=C1N | InChI=1/C10H9N3O/c11-9-5-8(6-13-10(9)14)7-1-3-12-4-2-7/h1-6H,11H2,(H,13,14) |
|  | Hydroquinone | 74 | 0 | 0 | 0 | 0 | 0 | 0 | O=C1C=C(O)C(=O)C=C1 | InChI=1/C6H4O3/c7-4-1-2-5(8)6(9)3-4/h1-3,9H |
|  | Milrinone | 75 | 0 | 0 | 0 | 0 | 0 | 0 | O=C1C(=O)C(C#N)=C(c2ccncc2)C=C1C | InChI=1/C13H8N2O2/c1-8-6-10(9-2-4-15-5-3-9)11(7-14)13(17)12(8)16/h2-6H,1H3 |
|  | menadion | 75 | 0 | 1 | 0 | 0 | 0 | 0 | CC1=CC(c2c(C1=O)cccc2)=O | InChI=1/C11H8O2/c1-7-6-10(12)8-4-2-3-5-9(8)11(7)13/h2-6H,1H3 |
|  | CAPE | 76 | 1 | 0 | 0 | 0 | 0 | 0 | Oc1c(O)cc(\C=C\C(=O)OCCc2ccccc2)cc1 | InChI=1/C17H16O4/c18-15-8-6-14(12-16(15)19)7-9-17(20)21-11-10-13-4-2-1-3-5-13/h1-9,12,18-19H,10-11H2/b9-7+ |
|  | CAPE analogue 1 | 76 | 1 | 0 | 0 | 0 | 0 | 0 | c1cc(O)c(\C=C\C(=O)OCCc2ccccc2)cc1O | InChI=1/C17H16O4/c18-15-7-8-16(19)14(12-15)6-9-17(20)21-11-10-13-4-2-1-3-5-13/h1-9,12,18-19H,10-11H2/b9-6+ |
|  | Clarithromycin | 79 | 0 | 0 | 0 | 0 | 0 | 0 | O=C1[C@@H](C)[C@@H](O[C@H]2C[C@](C)(OC)[C@H](O)[C@H](C)O2)[C@H](C)C(O[C@H]3[C@@H](O)[C@H](N(C)C)C[C@@H](C)O3)[C@](C)(OC)C[C@@H](C)C(=O)[C@H](C)[C@@H](O)[C@](C)(O)[C@@H](CC)O1 | InChI=1/C38H69NO13/c1-15-26-38(10,45)31(42)21(4)28(40)19(2)17-37(9,47-14)33(52-35-29(41)25(39(11)12)16-20(3)48-35)22(5)30(23(6)34(44)50-26)51-27-18-36(8,46-13)32(43)24(7)49-27/h19-27,29-33,35,41-43,45H,15-18H2,1-14H3/t19-,20-,21+,22+,23+,24+,25-,26-,27+,29+,30+,31-,32-,33?,35+,36+,37-,38-/m1/s1 |
|  | FK-506 | 79 | 0 | 0 | 0 | 0 | 0 | 0 | O=C1[C@]([C@@H](C[C@H]2OC)C)(O)O[C@@H]2[C@@H](OC)C[C@@H](C)C\C(\C)=C/[C@@H](CC=C)C(=O)C[C@H](O)[C@@H](C)[C@@H](\C(\C)=C\[C@H]3C[C@@H](OC)[C@H](O)CC3)OC(=O)[C@@H](CCCC4)N4C1=O | InChI=1/C44H69NO12/c1-10-13-31-19-25(2)18-26(3)20-37(54-8)40-38(55-9)22-28(5)44(52,57-40)41(49)42(50)45-17-12-11-14-32(45)43(51)56-39(29(6)34(47)24-35(31)48)27(4)21-30-15-16-33(46)36(23-30)53-7/h10,19,21,26,28-34,36-40,46-47,52H,1,11-18,20,22-24H2,2-9H3/b25-19-,27-21+/t26-,28+,29+,30-,31+,32+,33+,34-,36+,37-,38+,39+,40+,44+/m0/s1 |
|  | TP301 | 81 | 0 | 0 | 0 | 1 | 0 | 0 | [C@@]12([C@H](CC[C@@H]3C1=CC(=O)[C@H]([C@H](CC(C)(C)C[C@H]4C(=O)NCCCCCCCNC(=O)CCCC[C@@H]5S[C@H](NC(=O)N6)[C@H]6C5)[C@H]4CC7)[C@H]37)C(C)(C)C(=O)C(C#N)=C2)C | InChI=1/C46H67N5O5S/c1-44(2)24-32-29(15-16-31-30-17-18-37-45(3,4)40(54)27(26-47)23-46(37,5)34(30)22-36(52)39(31)32)33(25-44)41(55)49-20-12-8-6-7-11-19-48-38(53)14-10-9-13-28-21-35-42(57-28)51-43(56)50-35/h22-23,28-33,35,37,39,42H,6-21,24-25H2,1-5H3,(H,48,53)(H,49,55)(H2,50,51,56)/t28-,29+,30-,31-,32+,33+,35+,37+,39-,42-,46+/m0/s1 |
|  | TP304 | 81 | 0 | 0 | 0 | 1 | 0 | 0 | [C@@]12([C@H](CC[C@@H]3C1=CC(=O)[C@H]([C@H](CC(C)(C)C[C@H]4C(=O)OC)[C@H]4CC5)[C@H]35)[C@](C)(COC(=O)CCCCCNC(=O)CCCC[C@@H]6S[C@H](NC(=O)N7)[C@H]7C6)C(=O)C(C#N)=C2)C | InChI=1/C46H64N4O8S/c1-44(2)22-31-28(32(23-44)42(55)57-5)14-15-30-29-16-17-36-45(3,33(29)20-35(51)39(30)31)21-26(24-47)40(54)46(36,4)25-58-38(53)13-7-6-10-18-48-37(52)12-9-8-11-27-19-34-41(59-27)50-43(56)49-34/h20-21,27-32,34,36,39,41H,6-19,22-23,25H2,1-5H3,(H,48,52)(H2,49,50,56)/t27-,28+,29-,30-,31+,32+,34+,36-,39-,41-,45+,46-/m0/s1 |
|  | ROCAGLAMIDE 11 | 82 | 0 | 0 | 0 | 0 | 0 | 0 | c1(c([C@@]([C@H](O)C[C@H]2c3ccccc3)(O)[C@@]2(c4ccc(OC)cc4)O5)c5cc(OC)c1)OC | InChI=1/C26H26O6/c1-29-18-11-9-17(10-12-18)26-20(16-7-5-4-6-8-16)15-23(27)25(26,28)24-21(31-3)13-19(30-2)14-22(24)32-26/h4-14,20,23,27-28H,15H2,1-3H3/t20-,23+,25+,26-/m0/s1 |
|  | ROCAGLAMIDE 4 | 82 | 0 | 0 | 0 | 0 | 0 | 0 | c1(c([C@@]([C@H](O)[C@H](C(=O)N(C)C)[C@H]2c3ccccc3)(O)[C@@]2(c4ccc(OC)cc4)O5)c5cc(OC)c1)OC | InChI=1/C29H31NO7/c1-30(2)27(32)23-24(17-9-7-6-8-10-17)29(18-11-13-19(34-3)14-12-18)28(33,26(23)31)25-21(36-5)15-20(35-4)16-22(25)37-29/h6-16,23-24,26,31,33H,1-5H3/t23-,24-,26-,28+,29+/m1/s1 |
|  | N-alpha-p-Tosyl-L-lysine chloromethyl ketone | 84 | 0 | 0 | 0 | 0 | 1 | 0 | O=S(c1ccc(C)cc1)(N[C@H](C(=O)O)CCCCN)=O | InChI=1/C13H20N2O4S/c1-10-5-7-11(8-6-10)20(18,19)15-12(13(16)17)4-2-3-9-14/h5-8,12,15H,2-4,9,14H2,1H3,(H,16,17)/t12-/m0/s1 |
|  | L-buthionine-(S,R)-sulfoximine | 84 | 0 | 0 | 0 | 0 | 1 | 0 | CCCC[S@](CC[C@H](C(O)=O)N)(=O)=N | InChI=1/C8H18N2O3S/c1-2-3-5-14(10,13)6-4-7(9)8(11)12/h7,10H,2-6,9H2,1H3,(H,11,12)/t7-,14-/m1/s1 |
|  | sulfasalazine | 85 | 0 | 0 | 0 | 1 | 0 | 0 | O=S(c1ccc(N\N=C(\C=C(C(O)=O)C2=O)/C=C2)cc1)(Nc3ccccn3)=O | InChI=1/C18H14N4O5S/c23-16-9-6-13(11-15(16)18(24)25)21-20-12-4-7-14(8-5-12)28(26,27)22-17-3-1-2-10-19-17/h1-11,20H,(H,19,22)(H,24,25)/b21-13+ |
|  | T-614 | 85 | 0 | 0 | 0 | 0 | 0 | 0 | S(C)(Nc1cc2c(C(C(=CO2)NC=O)=O)cc1Oc3ccccc3)(=O)=O | InChI=1/C17H14N2O6S/c1-26(22,23)19-13-8-15-12(17(21)14(9-24-15)18-10-20)7-16(13)25-11-5-3-2-4-6-11/h2-10,19H,1H3,(H,18,20) |
|  | BAY 11-7085 | 86 | 0 | 0 | 0 | 0 | 1 | 0 | c1(ccc(S(\C=C/C#N)(=O)=O)cc1)C(C)(C)C | InChI=1/C13H15NO2S/c1-13(2,3)11-5-7-12(8-6-11)17(15,16)10-4-9-14/h4-8,10H,1-3H3/b10-4- |
|  | BAY 11-7082 | 86 | 0 | 0 | 0 | 0 | 1 | 0 | c1(ccc(S(\C=C/C#N)(=O)=O)cc1)C | InChI=1/C10H9NO2S/c1-9-3-5-10(6-4-9)14(12,13)8-2-7-11/h2-6,8H,1H3/b8-2- |
|  | SC-514 | 91 | 0 | 0 | 0 | 1 | 0 | 0 | s1c(C(N)=O)c(cc1c(c2)ccs2)N | InChI=1/C9H8N2OS2/c10-6-3-7(5-1-2-13-4-5)14-8(6)9(11)12/h1-4H,10H2,(H2,11,12) |
|  | thiophenecarboxamide\_baxter\_1 | 91 | 0 | 0 | 0 | 1 | 0 | 0 | NC(c1c(N)cc(c2cscc2)s1)=O | InChI=1/C9H8N2OS2/c10-6-3-7(5-1-2-13-4-5)14-8(6)9(11)12/h1-4H,10H2,(H2,11,12) |
|  | thiophenecarboxamide\_baxter\_5 | 91 | 0 | 0 | 0 | 1 | 0 | 0 | NC(c1c(N)cc(c2ccccc2)s1)=O | InChI=1/C11H10N2OS/c12-8-6-9(15-10(8)11(13)14)7-4-2-1-3-5-7/h1-6H,12H2,(H2,13,14) |
|  | thiophenecarboxamide\_baxter\_3 | 91 | 0 | 0 | 0 | 1 | 0 | 0 | NC(Nc1c(C(=O)N)sc(c2cscc2)c1)=O | InChI=1/C10H9N3O2S2/c11-9(14)8-6(13-10(12)15)3-7(17-8)5-1-2-16-4-5/h1-4H,(H2,11,14)(H3,12,13,15) |
|  | DHM3EQ | 92 | 0 | 0 | 0 | 0 | 0 | 0 | C1=C(NC(=O)c2ccccc2O)C(=O)[C@H](O3)[C@H]3[C@@H]1O | InChI=1/C13H11NO5/c15-8-4-2-1-3-6(8)13(18)14-7-5-9(16)11-12(19-11)10(7)17/h1-5,9,11-12,15-16H,(H,14,18)/t9-,11-,12+/m1/s1 |
|  | dehydroxymethylepoxyquinomicin | 92 | 0 | 0 | 0 | 0 | 0 | 1 | C1=C(NC(=O)c2c(O)cccc2)[C@H](O)[C@@H](O3)[C@@H]3C1=O | InChI=1/C13H11NO5/c15-8-4-2-1-3-6(8)13(18)14-7-5-9(16)11-12(19-11)10(7)17/h1-5,10-12,15,17H,(H,14,18)/t10-,11-,12+/m0/s1 |
|  | 200 | 96 | 1 | 0 | 0 | 0 | 0 | 0 | [C@H]1(C\C=C\CCCC(O)=O)\C(=C(\C)/C=C/CCCCCC(O)=O)\C(=O)C=C1[S-] | InChI=1/C22H30O5S/c1-16(11-7-3-2-4-9-13-20(24)25)22-17(19(28)15-18(22)23)12-8-5-6-10-14-21(26)27/h5,7-8,11,15,17,28H,2-4,6,9-10,12-14H2,1H3,(H,24,25)(H,26,27)/p-1/b8-5+,11-7+,22-16+/t17-/m1/s1 |
|  | 203 | 96 | 1 | 0 | 0 | 0 | 0 | 0 | [C@H]1(C\C=C\CCCC(O)=O)\C(=C/C=C/CCCCCC(O)=O)\C(=O)C=C1[S-] | InChI=1/C21H28O5S/c22-18-15-19(27)17(12-8-5-6-10-14-21(25)26)16(18)11-7-3-1-2-4-9-13-20(23)24/h3,5,7-8,11,15,17,27H,1-2,4,6,9-10,12-14H2,(H,23,24)(H,25,26)/p-1/b7-3+,8-5+,16-11+/t17-/m0/s1 |
|  | 188 | 96 | 1 | 0 | 0 | 0 | 0 | 0 | [C@H]1(C\C=C\CCCC(O)=O)\C(=C(\C)/C=C/CCCC[C@@](C)(CCC(O)=O)O)\C(=O)C=C1 | InChI=1/C25H36O6/c1-19(11-7-5-6-10-17-25(2,31)18-16-23(29)30)24-20(14-15-21(24)26)12-8-3-4-9-13-22(27)28/h3,7-8,11,14-15,20,31H,4-6,9-10,12-13,16-18H2,1-2H3,(H,27,28)(H,29,30)/b8-3+,11-7+,24-19+/t20-,25+/m1/s1 |
|  | 185 | 96 | 1 | 0 | 0 | 0 | 0 | 0 | [C@H]1(C\C=C\CCCC(O)=O)\C(=C\C=C\CCCCC)\C(=O)C=C1C(O)=O | InChI=1/C21H28O5/c1-2-3-4-5-6-10-13-17-16(18(21(25)26)15-19(17)22)12-9-7-8-11-14-20(23)24/h6-7,9-10,13,15-16H,2-5,8,11-12,14H2,1H3,(H,23,24)(H,25,26)/b9-7+,10-6+,17-13-/t16-/m0/s1 |
|  | 187 | 96 | 1 | 0 | 0 | 0 | 0 | 0 | [C@H]1(C\C=C\CCCC(O)=O)\C(=C(\C)/C=C/CCCC[C@@H](C)CCC(O)=O)\C(=O)C=C1 | InChI=1/C25H36O5/c1-19(15-18-24(29)30)11-7-3-4-8-12-20(2)25-21(16-17-22(25)26)13-9-5-6-10-14-23(27)28/h5,8-9,12,16-17,19,21H,3-4,6-7,10-11,13-15,18H2,1-2H3,(H,27,28)(H,29,30)/b9-5+,12-8+,25-20+/t19-,21-/m1/s1 |
|  | 202 | 96 | 1 | 0 | 0 | 0 | 0 | 0 | [C@H]1(C\C=C\CCCC(O)=O)\C(=C\C=C\CCCCC)\C(=O)C=C1[S-] | InChI=1/C20H28O3S/c1-2-3-4-5-6-9-12-16-17(19(24)15-18(16)21)13-10-7-8-11-14-20(22)23/h6-7,9-10,12,15,17,24H,2-5,8,11,13-14H2,1H3,(H,22,23)/p-1/b9-6+,10-7+,16-12-/t17-/m0/s1 |
|  | 183 | 96 | 1 | 0 | 0 | 0 | 0 | 0 | [C@H]1(C\C=C\CCCC(O)=O)\C(=C/C=C/CCC[C@@H](C)F)\C(=O)C=C1O | InChI=1/C20H27FO4/c1-15(21)10-6-2-3-7-11-16-17(19(23)14-18(16)22)12-8-4-5-9-13-20(24)25/h3-4,7-8,11,14-15,17,23H,2,5-6,9-10,12-13H2,1H3,(H,24,25)/b7-3+,8-4+,16-11+/t15-,17+/m1/s1 |
|  | 198 | 96 | 1 | 0 | 0 | 0 | 0 | 0 | [C@H]1(C\C=C\CCCC(O)=O)\C(=C\C=C\CCCCC)\C(=O)C=C1S | InChI=1/C20H28O3S/c1-2-3-4-5-6-9-12-16-17(19(24)15-18(16)21)13-10-7-8-11-14-20(22)23/h6-7,9-10,12,15,17,24H,2-5,8,11,13-14H2,1H3,(H,22,23)/b9-6+,10-7+,16-12-/t17-/m0/s1 |
|  | 196 | 96 | 1 | 0 | 0 | 0 | 0 | 0 | [C@H]1(C\C=C\CCCC(O)=O)\C(=C/C=C/CCC[C@@H](C)F)\C(=O)C=C1S | InChI=1/C20H27FO3S/c1-15(21)10-6-2-3-7-11-16-17(19(25)14-18(16)22)12-8-4-5-9-13-20(23)24/h3-4,7-8,11,14-15,17,25H,2,5-6,9-10,12-13H2,1H3,(H,23,24)/b7-3+,8-4+,16-11+/t15-,17+/m1/s1 |
|  | 181 | 96 | 1 | 0 | 0 | 0 | 0 | 0 | [C@H]1(C\C=C\CCCC(O)=O)\C(=C\C=C\CCCCC)\C(=O)C=C1Br | InChI=1/C20H27BrO3/c1-2-3-4-5-6-10-13-17-16(18(21)15-19(17)22)12-9-7-8-11-14-20(23)24/h6-7,9-10,13,15-16H,2-5,8,11-12,14H2,1H3,(H,23,24)/b9-7+,10-6+,17-13-/t16-/m0/s1 |
|  | 197 | 96 | 1 | 0 | 0 | 0 | 0 | 0 | [C@H]1(C\C=C\CCCC(O)=O)\C(=C/C=C/CCC[C@@H](C)O)\C(=O)C=C1S | InChI=1/C20H28O4S/c1-15(21)10-6-2-3-7-11-16-17(19(25)14-18(16)22)12-8-4-5-9-13-20(23)24/h3-4,7-8,11,14-15,17,21,25H,2,5-6,9-10,12-13H2,1H3,(H,23,24)/b7-3+,8-4+,16-11+/t15-,17+/m1/s1 |
|  | 195 | 96 | 1 | 0 | 0 | 0 | 0 | 0 | [C@H]1(C\C=C\CCCC(O)=O)\C(=C/C=C/CCC[C@@H](C)O)\C(=O)C=C1O | InChI=1/C20H28O5/c1-15(21)10-6-2-3-7-11-16-17(19(23)14-18(16)22)12-8-4-5-9-13-20(24)25/h3-4,7-8,11,14-15,17,21,23H,2,5-6,9-10,12-13H2,1H3,(H,24,25)/b7-3+,8-4+,16-11+/t15-,17+/m1/s1 |
|  | 191 | 96 | 1 | 0 | 0 | 0 | 0 | 0 | [C@H]1(C\C=C\CCCC(O)=O)\C(=C/C=C/CCC[C@@H]([N+]([O-])=O)C)\C(=O)C=C1 | InChI=1/C20H27NO5/c1-16(21(25)26)10-6-2-4-8-12-18-17(14-15-19(18)22)11-7-3-5-9-13-20(23)24/h3-4,7-8,12,14-17H,2,5-6,9-11,13H2,1H3,(H,23,24)/b7-3+,8-4+,18-12+/t16-,17+/m0/s1 |
|  | 1-hydroxy-1-hydroperoxynonane | 97 | 1 | 0 | 0 | 0 | 0 | 0 | CCCCCCCC[C@H](OO)O | InChI=1/C9H20O3/c1-2-3-4-5-6-7-8-9(10)12-11/h9-11H,2-8H2,1H3/t9-/m0/s1 |
|  | 4-hydroxynonenal | 97 | 0 | 0 | 0 | 0 | 1 | 0 | CCCCC[C@H](\C=C\C=O)O | InChI=1/C9H16O2/c1-2-3-4-6-9(11)7-5-8-10/h5,7-9,11H,2-4,6H2,1H3/b7-5+/t9-/m1/s1 |
|  | ATM | 99 | 0 | 0 | 0 | 0 | 0 | 0 | C([O-])(=O)[C@@H](S[Au])CC(O)=O.[Na+] | InChI=1/C4H6O4S.Au.Na/c5-3(6)1-2(9)4(7)8;;/h2,9H,1H2,(H,5,6)(H,7,8);;/q;2\*+1/p-2/t2-;;/m0../s1 |
|  | N-acetyl-L-cysteine (NAC) | 99 | 0 | 0 | 0 | 0 | 0 | 1 | O=C([C@H](CS)NC(C)=O)O | InChI=1/C5H9NO3S/c1-3(7)6-4(2-10)5(8)9/h4,10H,2H2,1H3,(H,6,7)(H,8,9)/t4-/m0/s1 |
|  | S-ALLYL CYSTEINE | 99 | 0 | 1 | 0 | 0 | 0 | 0 | O=C([C@H](CS)N)O | InChI=1/C3H7NO2S/c4-2(1-7)3(5)6/h2,7H,1,4H2,(H,5,6)/t2-/m0/s1 |
|  | beta Carboline 20 | 100 | 0 | 0 | 0 | 1 | 0 | 0 | c12c(ccnc1)c(cc(Cl)cc3N(C)c4ccccc4)c3[nH]2 | InChI=1/C18H14ClN3/c1-22(13-5-3-2-4-6-13)17-10-12(19)9-15-14-7-8-20-11-16(14)21-18(15)17/h2-11,21H,1H3 |
|  | beta Carboline 23 | 100 | 0 | 0 | 0 | 1 | 0 | 0 | c12c(ccnc1)c(cc(Cl)cc3NC(=O)c4ccccc4)c3[nH]2 | InChI=1/C18H12ClN3O/c19-12-8-14-13-6-7-20-10-16(13)21-17(14)15(9-12)22-18(23)11-4-2-1-3-5-11/h1-10,21H,(H,22,23) |
|  | beta Carboline 32 | 100 | 0 | 0 | 0 | 1 | 0 | 0 | c12c(ccnc1)c(cc(Cl)cc3NS(=O)(=O)c4ccccc4)c3[nH]2 | InChI=1/C17H12ClN3O2S/c18-11-8-14-13-6-7-19-10-16(13)20-17(14)15(9-11)21-24(22,23)12-4-2-1-3-5-12/h1-10,20-21H |
|  | beta Carboline 28 | 100 | 0 | 0 | 0 | 1 | 0 | 0 | c12c(ccnc1)c(cc(Cl)cc3NC(=O)c4c(OC)cccc4)c3[nH]2 | InChI=1/C19H14ClN3O2/c1-25-17-5-3-2-4-13(17)19(24)23-15-9-11(20)8-14-12-6-7-21-10-16(12)22-18(14)15/h2-10,22H,1H3,(H,23,24) |
|  | beta Carboline 29 | 100 | 0 | 0 | 0 | 1 | 0 | 0 | c12c(ccnc1)c(cc(Cl)cc3NC(=O)c4cc(OC)ccc4)c3[nH]2 | InChI=1/C19H14ClN3O2/c1-25-13-4-2-3-11(7-13)19(24)23-16-9-12(20)8-15-14-5-6-21-10-17(14)22-18(15)16/h2-10,22H,1H3,(H,23,24) |
|  | beta-Carboline 13 | 100 | 0 | 0 | 0 | 1 | 0 | 0 | c12c(ccnc1)c(cc(Cl)c(OCC3CCCCC3)c4Cl)c4[nH]2 | InChI=1/C18H18Cl2N2O/c19-14-8-13-12-6-7-21-9-15(12)22-17(13)16(20)18(14)23-10-11-4-2-1-3-5-11/h6-9,11,22H,1-5,10H2 |
|  | beta Carboline 25 | 100 | 0 | 0 | 0 | 1 | 0 | 0 | c12c(ccnc1)c(cc(Cl)cc3NC(=O)c4ncccc4)c3[nH]2 | InChI=1/C17H11ClN4O/c18-10-7-12-11-4-6-19-9-15(11)21-16(12)14(8-10)22-17(23)13-3-1-2-5-20-13/h1-9,21H,(H,22,23) |
|  | beta Carboline 24 | 100 | 0 | 0 | 0 | 1 | 0 | 0 | c12c(ccnc1)c(cc(Cl)cc3NC(=O)Cc4ccccn4)c3[nH]2 | InChI=1/C18H13ClN4O/c19-11-7-14-13-4-6-20-10-16(13)23-18(14)15(8-11)22-17(24)9-12-3-1-2-5-21-12/h1-8,10,23H,9H2,(H,22,24) |
|  | beta-Carboline 11 | 100 | 0 | 0 | 0 | 1 | 0 | 0 | c12c(ccnc1)c(cc(Cl)c(OCC)c3Cl)c3[nH]2 | InChI=1/C13H10Cl2N2O/c1-2-18-13-9(14)5-8-7-3-4-16-6-10(7)17-12(8)11(13)15/h3-6,17H,2H2,1H3 |
|  | o-phenanthroline | 106 | 1 | 0 | 0 | 0 | 0 | 0 | n1cccc(ccc2c3nccc2)c13 | InChI=1/C12H8N2/c1-3-9-5-6-10-4-2-8-14-12(10)11(9)13-7-1/h1-8H |
|  | 9AA | 106 | 1 | 0 | 0 | 0 | 0 | 0 | c12c(nc3c(cccc3)c1N)cccc2 | InChI=1/C13H10N2/c14-13-9-5-1-3-7-11(9)15-12-8-4-2-6-10(12)13/h1-8H,(H2,14,15) |
|  | glutathione | 107 | 0 | 0 | 0 | 0 | 1 | 0 | C([C@H](N)C(O)=O)CC(N[C@H](C(NCC(O)=O)=O)CSSC[C@H](NC(CC[C@H](N)C(O)=O)=O)C(NCC(O)=O)=O)=O | InChI=1/C20H32N6O12S2/c21-9(19(35)36)1-3-13(27)25-11(17(33)23-5-15(29)30)7-39-40-8-12(18(34)24-6-16(31)32)26-14(28)4-2-10(22)20(37)38/h9-12H,1-8,21-22H2,(H,23,33)(H,24,34)(H,25,27)(H,26,28)(H,29,30)(H,31,32)(H,35,36)(H,37,38)/t9-,10-,11-,12-/m0/s1 |
|  | S-nitrosogluthathione | 107 | 0 | 0 | 0 | 0 | 1 | 0 | S(C[C@H](NC(CC[C@H](N)C(O)=O)=O)C(NCC(O)=O)=O)N=O | InChI=1/C10H16N4O7S/c11-5(10(19)20)1-2-7(15)13-6(4-22-14-21)9(18)12-3-8(16)17/h5-6H,1-4,11H2,(H,12,18)(H,13,15)(H,16,17)(H,19,20)/t5-,6-/m0/s1 |
|  | 6ee | 109 | 0 | 0 | 0 | 0 | 0 | 0 | c1cc(Cl)c(\C=C\c2ccc(OC)cc2)c(Cl)c1 | InChI=1/C15H12Cl2O/c1-18-12-8-5-11(6-9-12)7-10-13-14(16)3-2-4-15(13)17/h2-10H,1H3/b10-7+ |
|  | 6j | 109 | 0 | 0 | 0 | 0 | 0 | 0 | c1cc(Cl)c(\C=C\c2ccc(OC)cc2)cc1 | InChI=1/C15H13ClO/c1-17-14-10-7-12(8-11-14)6-9-13-4-2-3-5-15(13)16/h2-11H,1H3/b9-6+ |
|  | 6c | 109 | 0 | 0 | 0 | 0 | 0 | 0 | c1(ccc(\C=C\c2ccc(OC)cc2)cc1)Cl | InChI=1/C15H13ClO/c1-17-15-10-6-13(7-11-15)3-2-12-4-8-14(16)9-5-12/h2-11H,1H3/b3-2+ |
|  | 6h | 109 | 0 | 0 | 0 | 0 | 0 | 0 | c1c(Cl)cc(\C=C\c2ccc(OC)cc2)cc1 | InChI=1/C15H13ClO/c1-17-15-9-7-12(8-10-15)5-6-13-3-2-4-14(16)11-13/h2-11H,1H3/b6-5+ |
|  | 4dd | 109 | 0 | 0 | 0 | 0 | 0 | 0 | c1cc(Cl)c(\C=C\c2ccccc2)c(Cl)c1 | InChI=1/C14H10Cl2/c15-13-7-4-8-14(16)12(13)10-9-11-5-2-1-3-6-11/h1-10H/b10-9+ |
|  | 6w | 109 | 0 | 0 | 0 | 0 | 0 | 0 | c1c(Br)cc(\C=C\c2ccc(OC)cc2)cc1 | InChI=1/C15H13BrO/c1-17-15-9-7-12(8-10-15)5-6-13-3-2-4-14(16)11-13/h2-11H,1H3/b6-5+ |
|  | 6t | 109 | 0 | 0 | 0 | 0 | 0 | 0 | c1(ccc(\C=C\c2ccc(OC)cc2)cc1)Br | InChI=1/C15H13BrO/c1-17-15-10-6-13(7-11-15)3-2-12-4-8-14(16)9-5-12/h2-11H,1H3/b3-2+ |
|  | 6aa | 109 | 0 | 0 | 0 | 0 | 0 | 0 | c1(cc(C)c(\C=C\c2ccc(OC)cc2)c(C)c1)C | InChI=1/C18H20O/c1-13-11-14(2)18(15(3)12-13)10-7-16-5-8-17(19-4)9-6-16/h5-12H,1-4H3/b10-7+ |
|  | 4b | 109 | 0 | 0 | 0 | 0 | 0 | 0 | c1(ccc(\C=C\c2ccccc2)cc1)OC | InChI=1/C15H14O/c1-16-15-11-9-14(10-12-15)8-7-13-5-3-2-4-6-13/h2-12H,1H3/b8-7+ |
|  | 6b | 109 | 0 | 0 | 0 | 0 | 0 | 0 | c1(ccc(\C=C\c2ccc(OC)cc2)cc1)OC | InChI=1/C16H16O2/c1-17-15-9-5-13(6-10-15)3-4-14-7-11-16(18-2)12-8-14/h3-12H,1-2H3/b4-3+ |
|  | 6d | 109 | 0 | 0 | 0 | 0 | 0 | 0 | c1(ccc(\C=C\c2ccc(OC)cc2)cc1)C | InChI=1/C16H16O/c1-13-3-5-14(6-4-13)7-8-15-9-11-16(17-2)12-10-15/h3-12H,1-2H3/b8-7+ |
|  | 6r | 109 | 0 | 0 | 0 | 0 | 0 | 0 | c1c(OC)cc(\C=C\c2ccc(OC)cc2)cc1 | InChI=1/C16H16O2/c1-17-15-10-8-13(9-11-15)6-7-14-4-3-5-16(12-14)18-2/h3-12H,1-2H3/b7-6+ |
|  | 6i | 109 | 0 | 0 | 0 | 0 | 0 | 0 | c1c(C)cc(\C=C\c2ccc(OC)cc2)cc1 | InChI=1/C16H16O/c1-13-4-3-5-15(12-13)7-6-14-8-10-16(17-2)11-9-14/h3-12H,1-2H3/b7-6+ |
|  | 6m | 109 | 0 | 0 | 0 | 0 | 0 | 0 | c1(ccc(\C=C\c2ccc(OC)cc2)cc1)F | InChI=1/C15H13FO/c1-17-15-10-6-13(7-11-15)3-2-12-4-8-14(16)9-5-12/h2-11H,1H3/b3-2+ |
|  | 6dd | 109 | 0 | 0 | 0 | 0 | 0 | 0 | c1(ccc(\C=C\c2ccc(OC)cc2)cc1)N(C)C | InChI=1/C17H19NO/c1-18(2)16-10-6-14(7-11-16)4-5-15-8-12-17(19-3)13-9-15/h4-13H,1-3H3/b5-4+ |
|  | 6z | 109 | 0 | 0 | 0 | 0 | 0 | 0 | c1cc(C(F)(F)F)c(\C=C\c2ccc(OC)cc2)cc1 | InChI=1/C16H13F3O/c1-20-14-10-7-12(8-11-14)6-9-13-4-2-3-5-15(13)16(17,18)19/h2-11H,1H3/b9-6+ |
|  | 6n | 109 | 0 | 0 | 0 | 0 | 0 | 0 | c1c(F)cc(\C=C\c2ccc(OC)cc2)cc1 | InChI=1/C15H13FO/c1-17-15-9-7-12(8-10-15)5-6-13-3-2-4-14(16)11-13/h2-11H,1H3/b6-5+ |
|  | 6p | 109 | 0 | 0 | 0 | 0 | 0 | 0 | c1cc(F)c(\C=C\c2ccc(OC)cc2)cc1 | InChI=1/C15H13FO/c1-17-14-10-7-12(8-11-14)6-9-13-4-2-3-5-15(13)16/h2-11H,1H3/b9-6+ |
|  | 4r | 109 | 0 | 0 | 0 | 0 | 0 | 0 | c1c(OC)cc(\C=C\c2ccccc2)cc1 | InChI=1/C15H14O/c1-16-15-9-5-8-14(12-15)11-10-13-6-3-2-4-7-13/h2-12H,1H3/b11-10+ |
|  | 6e | 109 | 0 | 0 | 0 | 0 | 0 | 0 | c1(C#N)ccc(\C=C\c2ccc(OC)cc2)cc1 | InChI=1/C16H13NO/c1-18-16-10-8-14(9-11-16)3-2-13-4-6-15(12-17)7-5-13/h2-11H,1H3/b3-2+ |
|  | 6k | 109 | 0 | 0 | 0 | 0 | 0 | 0 | c1(ccc(\C=C\c2ccc(OC)cc2)cc1)OCC | InChI=1/C17H18O2/c1-3-19-17-12-8-15(9-13-17)5-4-14-6-10-16(18-2)11-7-14/h4-13H,3H2,1-2H3/b5-4+ |
|  | 6bb | 109 | 0 | 0 | 0 | 0 | 0 | 0 | c1cc(OC)c(\C=C\c2ccc(OC)cc2)cc1 | InChI=1/C16H16O2/c1-17-15-11-8-13(9-12-15)7-10-14-5-3-4-6-16(14)18-2/h3-12H,1-2H3/b10-7+ |
|  | 4k | 109 | 0 | 0 | 0 | 0 | 0 | 0 | c1(ccc(\C=C\c2ccccc2)cc1)OCC | InChI=1/C16H16O/c1-2-17-16-12-10-15(11-13-16)9-8-14-6-4-3-5-7-14/h3-13H,2H2,1H3/b9-8+ |
|  | 4y | 109 | 0 | 0 | 0 | 0 | 0 | 0 | c1cc(OC)c(\C=C\c2ccccc2)cc1OC | InChI=1/C16H16O2/c1-17-15-10-11-16(18-2)14(12-15)9-8-13-6-4-3-5-7-13/h3-12H,1-2H3/b9-8+ |
|  | 4g | 109 | 0 | 0 | 0 | 0 | 0 | 0 | c1cc(OC)c(\C=C\c2ccccc2)c(OC)c1 | InChI=1/C16H16O2/c1-17-15-9-6-10-16(18-2)14(15)12-11-13-7-4-3-5-8-13/h3-12H,1-2H3/b12-11+ |
|  | 4f | 109 | 0 | 0 | 0 | 0 | 0 | 0 | c1c(OC)cc(\C=C\c2ccccc2)cc1OC | InChI=1/C16H16O2/c1-17-15-10-14(11-16(12-15)18-2)9-8-13-6-4-3-5-7-13/h3-12H,1-2H3/b9-8+ |
|  | 4j | 109 | 0 | 0 | 0 | 0 | 0 | 0 | c1cc(Cl)c(\C=C\c2ccccc2)cc1 | InChI=1/C14H11Cl/c15-14-9-5-4-8-13(14)11-10-12-6-2-1-3-7-12/h1-11H/b11-10+ |
|  | 4a | 109 | 0 | 0 | 0 | 0 | 0 | 0 | c1(c(OC)cc(\C=C\c2ccccc2)cc1)OC | InChI=1/C16H16O2/c1-17-15-11-10-14(12-16(15)18-2)9-8-13-6-4-3-5-7-13/h3-12H,1-2H3/b9-8+ |
|  | 4cc | 109 | 0 | 0 | 0 | 0 | 0 | 0 | c1(ccc(\C=C\c2ccccc2)cc1)N(C)C | InChI=1/C16H17N/c1-17(2)16-12-10-15(11-13-16)9-8-14-6-4-3-5-7-14/h3-13H,1-2H3/b9-8+ |
|  | 4o | 109 | 0 | 0 | 0 | 0 | 0 | 0 | c1c(OC)c(OC)c(\C=C\c2ccccc2)cc1 | InChI=1/C16H16O2/c1-17-15-10-6-9-14(16(15)18-2)12-11-13-7-4-3-5-8-13/h3-12H,1-2H3/b12-11+ |
|  | 4bb | 109 | 0 | 0 | 0 | 0 | 0 | 0 | c1cc(OC)c(\C=C\c2ccccc2)cc1 | InChI=1/C15H14O/c1-16-15-10-6-5-9-14(15)12-11-13-7-3-2-4-8-13/h2-12H,1H3/b12-11+ |
|  | 4z | 109 | 0 | 0 | 0 | 0 | 0 | 0 | c1cc(C(F)(F)F)c(\C=C\c2ccccc2)cc1 | InChI=1/C15H11F3/c16-15(17,18)14-9-5-4-8-13(14)11-10-12-6-2-1-3-7-12/h1-11H/b11-10+ |
|  | phenethyl isothiocyanate | 110 | 0 | 0 | 0 | 0 | 1 | 0 | S=C=NCCc1ccccc1 | InChI=1/C9H9NS/c11-8-10-7-6-9-4-2-1-3-5-9/h1-5H,6-7H2 |
|  | Methaamphetamine | 110 | 0 | 1 | 0 | 0 | 0 | 0 | c1(ccccc1)C[C@H](C)NC | InChI=1/C10H15N/c1-9(11-2)8-10-6-4-3-5-7-10/h3-7,9,11H,8H2,1-2H3/t9-/m0/s1 |
|  | quinone monomer (7) | 111 | 0 | 0 | 0 | 0 | 0 | 0 | C\C=C/C1=C(C([C@@H]2[C@@H](C1=O)O2)=O)CO | InChI=1/C10H10O4/c1-2-3-5-6(4-11)8(13)10-9(14-10)7(5)12/h2-3,9-11H,4H2,1H3/b3-2-/t9-,10-/m1/s1 |
|  | quinol monomer (3) | 111 | 0 | 0 | 0 | 0 | 0 | 0 | C\C=C/C1=C([C@@H]([C@@H]2[C@@H](C1=O)O2)O)CO | InChI=1/C10H12O4/c1-2-3-5-6(4-11)8(13)10-9(14-10)7(5)12/h2-3,8-11,13H,4H2,1H3/b3-2-/t8-,9+,10+/m0/s1 |
|  | Panepoxydone | 111 | 0 | 0 | 0 | 0 | 1 | 0 | C\C(\C)=C/[C@H](O)C1=C[C@@H](O)[C@H]2[C@H](O2)C1=O | InChI=1/C11H14O4/c1-5(2)3-7(12)6-4-8(13)10-11(15-10)9(6)14/h3-4,7-8,10-13H,1-2H3/t7-,8+,10-,11+/m0/s1 |
|  | Baicalin | 112 | 0 | 0 | 0 | 0 | 0 | 0 | O=C1C=C(c2ccccc2)Oc(cc(O[C@@H]3[C@H](O)[C@@H](O)[C@@H](O)[C@@H](C(O)=O)O3)c(O)c4O)c14 | InChI=1/C21H18O11/c22-9-6-10(8-4-2-1-3-5-8)30-11-7-12(14(23)15(24)13(9)11)31-21-18(27)16(25)17(26)19(32-21)20(28)29/h1-7,16-19,21,23-27H,(H,28,29)/t16-,17+,18+,19-,21-/m0/s1 |
|  | Doxorubicin | 112 | 1 | 0 | 0 | 0 | 0 | 0 | COc1c(C(=O)c(c(O)c([C@@H](O[C@H]2O[C@@H](C)[C@@H](O)[C@@H](N)C2)C[C@@](C(CO)=O)(O)C3)c3c4O)c4C5=O)c5ccc1 | InChI=1/C27H29NO11/c1-10-22(31)13(28)6-17(38-10)39-15-8-27(36,16(30)9-29)7-12-19(15)26(35)21-20(24(12)33)23(32)11-4-3-5-14(37-2)18(11)25(21)34/h3-5,10,13,15,17,22,29,31,33,35-36H,6-9,28H2,1-2H3/t10-,13-,15-,17+,22+,27-/m0/s1 |
|  | Polipodine A (unknow steroechemistry on 2-OHs of on 6 membered ring) | 112 | 0 | 0 | 0 | 0 | 1 | 0 | CC(CC[C@H]([C@@](C1[C@@](C)(CC[C@H]([C@@](C)(C[C@H](O)[C@H](O)C2)[C@@H]2C3=O)C4=C3)[C@@]4(O)CC1)(O)C)O)(O)C | InChI=1/C27H44O7/c1-23(2,32)9-8-22(31)26(5,33)21-7-11-27(34)16-12-18(28)17-13-19(29)20(30)14-24(17,3)15(16)6-10-25(21,27)4/h12,15,17,19-22,29-34H,6-11,13-14H2,1-5H3/t15-,17-,19+,20-,21?,22+,24+,25+,26+,27+/m0/s1 |
|  | Compound 3b | 114 | 0 | 0 | 0 | 1 | 0 | 0 | c1(C#N)c(N)nc(c2cccc(C)c2O)cc1[C@@H]3CCCNC3 | InChI=1/C18H20N4O/c1-11-4-2-6-13(17(11)23)16-8-14(12-5-3-7-21-10-12)15(9-19)18(20)22-16/h2,4,6,8,12,21,23H,3,5,7,10H2,1H3,(H2,20,22)/t12-/m1/s1 |
|  | Compound 3e | 114 | 0 | 0 | 0 | 1 | 0 | 0 | c1(C#N)c(N)nc(c2c(C)cccc2O)cc1[C@@H]3CCCNC3 | InChI=1/C18H20N4O/c1-11-4-2-6-16(23)17(11)15-8-13(12-5-3-7-21-10-12)14(9-19)18(20)22-15/h2,4,6,8,12,21,23H,3,5,7,10H2,1H3,(H2,20,22)/t12-/m1/s1 |
|  | Compound 3a | 114 | 0 | 0 | 0 | 1 | 0 | 0 | c1(C#N)c(N)nc(c2ccccc2O)cc1[C@@H]3CCCNC3 | InChI=1/C17H18N4O/c18-9-14-13(11-4-3-7-20-10-11)8-15(21-17(14)19)12-5-1-2-6-16(12)22/h1-2,5-6,8,11,20,22H,3-4,7,10H2,(H2,19,21)/t11-/m1/s1 |
|  | Compound 4a | 114 | 0 | 0 | 0 | 1 | 0 | 0 | c1(C#N)c(N)nc(c2c(O)cccc2)cc1C3CCNCC3 | InChI=1/C17H18N4O/c18-10-14-13(11-5-7-20-8-6-11)9-15(21-17(14)19)12-3-1-2-4-16(12)22/h1-4,9,11,20,22H,5-8H2,(H2,19,21) |
|  | Compound 3c | 114 | 0 | 0 | 0 | 1 | 0 | 0 | c1(C#N)c(N)nc(c2cccc(OC)c2O)cc1[C@@H]3CCCNC3 | InChI=1/C18H20N4O2/c1-24-16-6-2-5-12(17(16)23)15-8-13(11-4-3-7-21-10-11)14(9-19)18(20)22-15/h2,5-6,8,11,21,23H,3-4,7,10H2,1H3,(H2,20,22)/t11-/m1/s1 |
|  | Compound 3d | 114 | 0 | 0 | 0 | 1 | 0 | 0 | c1(C#N)c(N)nc(c2ccc(OC)cc2O)cc1[C@@H]3CCCNC3 | InChI=1/C18H20N4O2/c1-24-12-4-5-13(17(23)7-12)16-8-14(11-3-2-6-21-10-11)15(9-19)18(20)22-16/h4-5,7-8,11,21,23H,2-3,6,10H2,1H3,(H2,20,22)/t11-/m1/s1 |
|  | Compound 3g | 114 | 0 | 0 | 0 | 1 | 0 | 0 | c1(C#N)c(N)nc(c2c(O)cccc2O)cc1[C@@H]3CCCNC3 | InChI=1/C17H18N4O2/c18-8-12-11(10-3-2-6-20-9-10)7-13(21-17(12)19)16-14(22)4-1-5-15(16)23/h1,4-5,7,10,20,22-23H,2-3,6,9H2,(H2,19,21)/t10-/m1/s1 |
|  | ROCAGLAMIDE 8 | 115 | 0 | 0 | 0 | 0 | 0 | 0 | c1(c([C@@]([C@H](O)[C@H](C(=O)NCCCCO)[C@H]2c3ccccc3)(O)[C@@]2(c4ccc(OC)cc4)O5)c5cc(OC)c1)OC | InChI=1/C31H35NO8/c1-37-21-13-11-20(12-14-21)31-26(19-9-5-4-6-10-19)25(29(35)32-15-7-8-16-33)28(34)30(31,36)27-23(39-3)17-22(38-2)18-24(27)40-31/h4-6,9-14,17-18,25-26,28,33-34,36H,7-8,15-16H2,1-3H3,(H,32,35)/t25-,26-,28-,30+,31+/m1/s1 |
|  | ROCAGLAMIDE 3 | 115 | 0 | 0 | 0 | 0 | 0 | 0 | c1(c([C@@]([C@H](O)[C@H](C(=O)NC)[C@H]2c3ccccc3)(O)[C@@]2(c4ccc(OC)cc4)O5)c5cc(OC)c1)OC | InChI=1/C28H29NO7/c1-29-26(31)22-23(16-8-6-5-7-9-16)28(17-10-12-18(33-2)13-11-17)27(32,25(22)30)24-20(35-4)14-19(34-3)15-21(24)36-28/h5-15,22-23,25,30,32H,1-4H3,(H,29,31)/t22-,23-,25-,27+,28+/m1/s1 |
|  | ROCAGLAMIDE 1 | 115 | 0 | 0 | 0 | 0 | 0 | 0 | c1(c([C@@]([C@H](O)[C@H](C(=O)N)[C@H]2c3ccccc3)(O)[C@@]2(c4ccc(OC)cc4)O5)c5cc(OC)c1)OC | InChI=1/C27H27NO7/c1-32-17-11-9-16(10-12-17)27-22(15-7-5-4-6-8-15)21(25(28)30)24(29)26(27,31)23-19(34-3)13-18(33-2)14-20(23)35-27/h4-14,21-22,24,29,31H,1-3H3,(H2,28,30)/t21-,22-,24-,26+,27+/m1/s1 |
|  | ROCAGLAMIDE 2 | 115 | 0 | 0 | 0 | 0 | 0 | 0 | c1(c([C@@]([C@H](O)[C@H](C(=O)N)[C@H]2c3ccccc3)(O)[C@@]2(c4ccc(OC)c(O)c4)O5)c5cc(OC)c1)OC | InChI=1/C27H27NO8/c1-33-16-12-19(35-3)23-20(13-16)36-27(15-9-10-18(34-2)17(29)11-15)22(14-7-5-4-6-8-14)21(25(28)31)24(30)26(23,27)32/h4-13,21-22,24,29-30,32H,1-3H3,(H2,28,31)/t21-,22-,24-,26+,27+/m1/s1 |
|  | ROCAGLAMIDE 9 | 115 | 0 | 0 | 0 | 0 | 0 | 0 | c1(c([C@@]([C@H](O)[C@H](C(=O)OC)[C@H]2c3ccccc3)(O)[C@@]2(c4ccc(OC)cc4)O5)c5cc(OC)c1)OC | InChI=1/C28H28O8/c1-32-18-12-10-17(11-13-18)28-23(16-8-6-5-7-9-16)22(26(30)35-4)25(29)27(28,31)24-20(34-3)14-19(33-2)15-21(24)36-28/h5-15,22-23,25,29,31H,1-4H3/t22-,23-,25-,27+,28+/m1/s1 |
|  | ROCAGLAMIDE 5 | 115 | 0 | 0 | 0 | 0 | 0 | 0 | c1(c([C@@]([C@H](O)[C@H](C(=O)N(C)C)[C@H]2c3ccccc3)(O)[C@@]2(c4ccc(OC)c(O)c4)O5)c5cc(OC)c1)OC | InChI=1/C29H31NO8/c1-30(2)27(33)23-24(16-9-7-6-8-10-16)29(17-11-12-20(36-4)19(31)13-17)28(34,26(23)32)25-21(37-5)14-18(35-3)15-22(25)38-29/h6-15,23-24,26,31-32,34H,1-5H3/t23-,24-,26-,28+,29+/m1/s1 |
|  | ROCAGLAMIDE 7 | 115 | 0 | 0 | 0 | 0 | 0 | 0 | c1(c([C@@]([C@H](OC(=O)C)[C@H](C(=O)N(C)C)[C@H]2c3ccccc3)(O)[C@@]2(c4ccc(OC)c(O)c4)O5)c5cc(OC)c1)OC | InChI=1/C31H33NO9/c1-17(33)40-28-25(29(35)32(2)3)26(18-10-8-7-9-11-18)31(19-12-13-22(38-5)21(34)14-19)30(28,36)27-23(39-6)15-20(37-4)16-24(27)41-31/h7-16,25-26,28,34,36H,1-6H3/t25-,26-,28-,30+,31+/m1/s1 |
|  | ROCAGLAMIDE 14 | 115 | 0 | 0 | 0 | 0 | 0 | 0 | c1(c([C@@]([C@H](O)[C@H](C(=O)OC)[C@H]2c3ccccc3)(O)[C@@]2(c4ccc(OCO5)c5c4)O6)c6cc(OC)c1)OC | InChI=1/C28H26O9/c1-32-17-12-20(33-2)24-21(13-17)37-28(16-9-10-18-19(11-16)36-14-35-18)23(15-7-5-4-6-8-15)22(26(30)34-3)25(29)27(24,28)31/h4-13,22-23,25,29,31H,14H2,1-3H3/t22-,23-,25-,27+,28+/m1/s1 |
|  | ROCAGLAMIDE 15 | 115 | 0 | 0 | 0 | 0 | 0 | 0 | c1(c([C@@]([C@H](O)[C@H](C(=O)OC)[C@H]2c3ccccc3)(O)[C@@]2(c4ccc(O)cc4)O5)c5cc(OCO6)c16)OC | InChI=1/C27H24O9/c1-32-23-21-17(12-18-22(23)35-13-34-18)36-27(15-8-10-16(28)11-9-15)20(14-6-4-3-5-7-14)19(25(30)33-2)24(29)26(21,27)31/h3-12,19-20,24,28-29,31H,13H2,1-2H3/t19-,20-,24-,26+,27+/m1/s1 |
|  | CAPE analogue 7 | 117 | 1 | 0 | 0 | 0 | 0 | 0 | C(\C=C\c1cc(c(cc1)O)O)(=O)NCCc2cc(O)c(O)c(O)c2 | InChI=1/C17H17NO6/c19-12-3-1-10(7-13(12)20)2-4-16(23)18-6-5-11-8-14(21)17(24)15(22)9-11/h1-4,7-9,19-22,24H,5-6H2,(H,18,23)/b4-2+ |
|  | N-(p-coumaroyl) serotonin | 117 | 0 | 0 | 0 | 0 | 0 | 0 | c12c([nH]cc1CCNC(=O)\C=C/c3ccc(O)cc3)ccc(O)c2 | InChI=1/C19H18N2O3/c22-15-4-1-13(2-5-15)3-8-19(24)20-10-9-14-12-21-18-7-6-16(23)11-17(14)18/h1-8,11-12,21-23H,9-10H2,(H,20,24)/b8-3- |
|  | Deoxyspergualin | 119 | 0 | 0 | 0 | 0 | 0 | 1 | O=C([C@H](NC(CCCCCC\N=C(\N)/N)=O)O)NCCCCNCCCN | InChI=1/C17H37N7O3/c18-9-7-11-21-10-5-6-12-22-15(26)16(27)24-14(25)8-3-1-2-4-13-23-17(19)20/h16,21,27H,1-13,18H2,(H,22,26)(H,24,25)(H4,19,20,23)/t16-/m1/s1 |
|  | Glucosamine sulphate | 119 | 0 | 0 | 0 | 0 | 1 | 0 | O[C@@H]1[C@H](N)[C@H](O)O[C@H](CO)[C@H]1O.OS(=O)(=O)O | InChI=1/C6H13NO5.H2O4S/c7-3-5(10)4(9)2(1-8)12-6(3)11;1-5(2,3)4/h2-6,8-11H,1,7H2;(H2,1,2,3,4)/t2-,3+,4-,5-,6-;/m1./s1 |
|  | S-ibuprofen | 121 | 1 | 0 | 0 | 0 | 0 | 0 | CC(Cc1ccc([C@@H](C(=O)O)C)cc1)C | InChI=1/C13H18O2/c1-9(2)8-11-4-6-12(7-5-11)10(3)13(14)15/h4-7,9-10H,8H2,1-3H3,(H,14,15)/t10-/m0/s1 |
|  | R-ibuprofen | 121 | 0 | 0 | 0 | 0 | 0 | 1 | CC(Cc1ccc([C@H](C(=O)O)C)cc1)C | InChI=1/C13H18O2/c1-9(2)8-11-4-6-12(7-5-11)10(3)13(14)15/h4-7,9-10H,8H2,1-3H3,(H,14,15)/t10-/m1/s1 |
|  | Compound 1 | 122 | 0 | 0 | 0 | 1 | 0 | 0 | c1(ccccc1O)c2cc(c3cccc(NC(=O)[C@@H]4CCC(=O)O4)c3)c(C#N)c(N)n2 | InChI=1/C23H18N4O4/c24-12-17-16(11-18(27-22(17)25)15-6-1-2-7-19(15)28)13-4-3-5-14(10-13)26-23(30)20-8-9-21(29)31-20/h1-7,10-11,20,28H,8-9H2,(H2,25,27)(H,26,30)/t20-/m0/s1 |
|  | 3-Cyano-2-Aminopyridines | 122 | 0 | 0 | 0 | 1 | 0 | 0 | c1(ccccc1O)c2cc(c3cccc(NC(=O)CN4CCCCC4)c3)c(C#N)c(N)n2 | InChI=1/C25H25N5O2/c26-15-21-20(14-22(29-25(21)27)19-9-2-3-10-23(19)31)17-7-6-8-18(13-17)28-24(32)16-30-11-4-1-5-12-30/h2-3,6-10,13-14,31H,1,4-5,11-12,16H2,(H2,27,29)(H,28,32) |
|  | Compound 16 | 122 | 0 | 0 | 0 | 1 | 0 | 0 | c1(ccccc1O)c2cc(c3cccc(NC(=O)CN4CCCCC4)c3)c(C#N)c(N)n2 | InChI=1/C25H25N5O2/c26-15-21-20(14-22(29-25(21)27)19-9-2-3-10-23(19)31)17-7-6-8-18(13-17)28-24(32)16-30-11-4-1-5-12-30/h2-3,6-10,13-14,31H,1,4-5,11-12,16H2,(H2,27,29)(H,28,32) |
|  | Compound 21 | 122 | 0 | 0 | 0 | 1 | 0 | 0 | c1(ccccc1O)c2cc(c3cccc(NC(=O)COC)c3)c(C#N)c(N)n2 | InChI=1/C21H18N4O3/c1-28-12-20(27)24-14-6-4-5-13(9-14)16-10-18(25-21(23)17(16)11-22)15-7-2-3-8-19(15)26/h2-10,26H,12H2,1H3,(H2,23,25)(H,24,27) |
|  | Compound 23 | 122 | 0 | 0 | 0 | 1 | 0 | 0 | c1(ccccc1O)c2cc(c3cccc(NC(=O)CCCC([O-])=O)c3)c(C#N)c(N)n2.[Na] | InChI=1/C23H20N4O4.Na.H/c24-13-18-17(12-19(27-23(18)25)16-7-1-2-8-20(16)28)14-5-3-6-15(11-14)26-21(29)9-4-10-22(30)31;;/h1-3,5-8,11-12,28H,4,9-10H2,(H2,25,27)(H,26,29)(H,30,31);;/p-1 |
|  | Compound 22 | 122 | 0 | 0 | 0 | 1 | 0 | 0 | c1(ccccc1O)c2cc(c3ccc(NC(=O)CCCC([O-])=O)cc3)c(C#N)c(N)n2.[Na] | InChI=1/C23H20N4O4.Na.H/c24-13-18-17(12-19(27-23(18)25)16-4-1-2-5-20(16)28)14-8-10-15(11-9-14)26-21(29)6-3-7-22(30)31;;/h1-2,4-5,8-12,28H,3,6-7H2,(H2,25,27)(H,26,29)(H,30,31);;/p-1 |
|  | Compound 3r | 122 | 0 | 0 | 0 | 1 | 0 | 0 | c1(C#N)c(N)nc(c2c(OCc3ccccc3)cccc2O)cc1[C@@H]4CCCNC4 | InChI=1/C24H24N4O2/c25-13-19-18(17-8-5-11-27-14-17)12-20(28-24(19)26)23-21(29)9-4-10-22(23)30-15-16-6-2-1-3-7-16/h1-4,6-7,9-10,12,17,27,29H,5,8,11,14-15H2,(H2,26,28)/t17-/m1/s1 |
|  | 5,7-dihydroxy-4-methylcoumarin | 130 | 1 | 0 | 0 | 0 | 0 | 0 | c12c(C(=CC(O1)=O)C)c(O)cc(O)c2 | InChI=1/C10H8O4/c1-5-2-9(13)14-8-4-6(11)3-7(12)10(5)8/h2-4,11-12H,1H3 |
|  | Phenobarbital | 130 | 0 | 1 | 0 | 0 | 0 | 0 | O=C1C(CC)(c2ccccc2)C(=O)NC(=O)N1 | InChI=1/C12H12N2O3/c1-2-12(8-6-4-3-5-7-8)9(15)13-11(17)14-10(12)16/h3-7H,2H2,1H3,(H2,13,14,15,16,17) |
|  | diarylheptanoid HMP | 134 | 0 | 0 | 0 | 0 | 0 | 0 | c1(ccccc1)CCC(=O)\C=C\CCc2cc(OC)c(O)cc2 | InChI=1/C20H22O3/c1-23-20-15-17(12-14-19(20)22)9-5-6-10-18(21)13-11-16-7-3-2-4-8-16/h2-4,6-8,10,12,14-15,22H,5,9,11,13H2,1H3/b10-6+ |
|  | Yakuchinone A | 134 | 0 | 0 | 0 | 0 | 0 | 0 | c1c(CCC(=O)CCCCc2ccccc2)cc(OC)c(O)c1 | InChI=1/C20H24O3/c1-23-20-15-17(12-14-19(20)22)11-13-18(21)10-6-5-9-16-7-3-2-4-8-16/h2-4,7-8,12,14-15,22H,5-6,9-11,13H2,1H3 |
|  | Yakuchinone B | 134 | 0 | 0 | 0 | 0 | 0 | 0 | c1c(\C=C\C(=O)CCCCc2ccccc2)cc(OC)c(O)c1 | InChI=1/C20H22O3/c1-23-20-15-17(12-14-19(20)22)11-13-18(21)10-6-5-9-16-7-3-2-4-8-16/h2-4,7-8,11-15,22H,5-6,9-10H2,1H3/b13-11+ |
|  | Part of BSASM mixture | 135 | 1 | 0 | 0 | 0 | 1 | 0 | O=C(c1cc(O)c(O)c(O)c1)O[C@H]2[C@H](c3cc(O)c(O)c(O)c3)Oc(cc(cc4O)O)c4C2 | InChI=1/C22H18O11/c23-10-5-12(24)11-7-18(33-22(31)9-3-15(27)20(30)16(28)4-9)21(32-17(11)6-10)8-1-13(25)19(29)14(26)2-8/h1-6,18,21,23-30H,7H2/t18-,21+/m1/s1 |
|  | 1,2,3,4,6-penta-O-galloyl-beta-D-glucose | 135 | 0 | 0 | 0 | 0 | 0 | 0 | O=C(c1cc(O)c(O)c(O)c1)O[C@H]2[C@@H](OC(c3cc(O)c(O)c(O)c3)=O)[C@@H](OC(c4cc(O)c(O)c(O)c4)=O)[C@H](OC(c5cc(O)c(O)c(O)c5)=O)[C@@H](COC(c6cc(O)c(O)c(O)c6)=O)O2 | InChI=1/C41H32O26/c42-17-1-12(2-18(43)28(17)52)36(57)62-11-27-33(64-37(58)13-3-19(44)29(53)20(45)4-13)34(65-38(59)14-5-21(46)30(54)22(47)6-14)35(66-39(60)15-7-23(48)31(55)24(49)8-15)41(63-27)67-40(61)16-9-25(50)32(56)26(51)10-16/h1-10,27,33-35,41-56H,11H2/t27-,33-,34+,35+,41+/m1/s1 |
