# Supplementary figures and images for: Predicting the points of interaction of small molecules in the NF-κB pathway (part 6 of 6)
Source: BMC Syst Biol. 2011 Feb 22;5:32. doi: 10.1186/1752-0509-5-32 (PMC3050742; doi:10.1186/1752-0509-5-32)

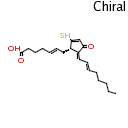

Supplement: Additional file 3 — Clusters of Compounds Shown in Figure 6. [file 1752-0509-5-32-S3.ZIP › Additional Files 3/Clustering_excluding_compounds_with_unknown_interactions_files/image5422.png]

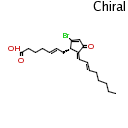

Supplement: Additional file 3 — Clusters of Compounds Shown in Figure 6. [file 1752-0509-5-32-S3.ZIP › Additional Files 3/Clustering_excluding_compounds_with_unknown_interactions_files/image5424.png]

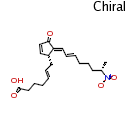

Supplement: Additional file 3 — Clusters of Compounds Shown in Figure 6. [file 1752-0509-5-32-S3.ZIP › Additional Files 3/Clustering_excluding_compounds_with_unknown_interactions_files/image5427.png]

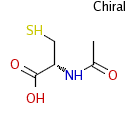

Supplement: Additional file 3 — Clusters of Compounds Shown in Figure 6. [file 1752-0509-5-32-S3.ZIP › Additional Files 3/Clustering_excluding_compounds_with_unknown_interactions_files/image5430.png]

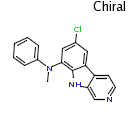

Supplement: Additional file 3 — Clusters of Compounds Shown in Figure 6. [file 1752-0509-5-32-S3.ZIP › Additional Files 3/Clustering_excluding_compounds_with_unknown_interactions_files/image5432.png]

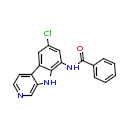

Supplement: Additional file 3 — Clusters of Compounds Shown in Figure 6. [file 1752-0509-5-32-S3.ZIP › Additional Files 3/Clustering_excluding_compounds_with_unknown_interactions_files/image5433.png]

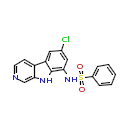

Supplement: Additional file 3 — Clusters of Compounds Shown in Figure 6. [file 1752-0509-5-32-S3.ZIP › Additional Files 3/Clustering_excluding_compounds_with_unknown_interactions_files/image5434.png]

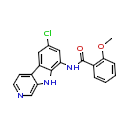

Supplement: Additional file 3 — Clusters of Compounds Shown in Figure 6. [file 1752-0509-5-32-S3.ZIP › Additional Files 3/Clustering_excluding_compounds_with_unknown_interactions_files/image5435.png]

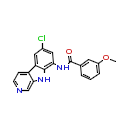

Supplement: Additional file 3 — Clusters of Compounds Shown in Figure 6. [file 1752-0509-5-32-S3.ZIP › Additional Files 3/Clustering_excluding_compounds_with_unknown_interactions_files/image5436.png]

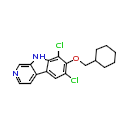

Supplement: Additional file 3 — Clusters of Compounds Shown in Figure 6. [file 1752-0509-5-32-S3.ZIP › Additional Files 3/Clustering_excluding_compounds_with_unknown_interactions_files/image5437.png]

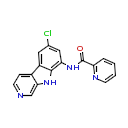

Supplement: Additional file 3 — Clusters of Compounds Shown in Figure 6. [file 1752-0509-5-32-S3.ZIP › Additional Files 3/Clustering_excluding_compounds_with_unknown_interactions_files/image5438.png]

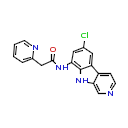

Supplement: Additional file 3 — Clusters of Compounds Shown in Figure 6. [file 1752-0509-5-32-S3.ZIP › Additional Files 3/Clustering_excluding_compounds_with_unknown_interactions_files/image5439.png]

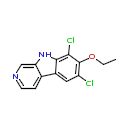

Supplement: Additional file 3 — Clusters of Compounds Shown in Figure 6. [file 1752-0509-5-32-S3.ZIP › Additional Files 3/Clustering_excluding_compounds_with_unknown_interactions_files/image5440.png]

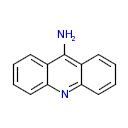

Supplement: Additional file 3 — Clusters of Compounds Shown in Figure 6. [file 1752-0509-5-32-S3.ZIP › Additional Files 3/Clustering_excluding_compounds_with_unknown_interactions_files/image5442.png]

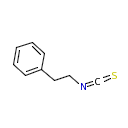

Supplement: Additional file 3 — Clusters of Compounds Shown in Figure 6. [file 1752-0509-5-32-S3.ZIP › Additional Files 3/Clustering_excluding_compounds_with_unknown_interactions_files/image5445.png]

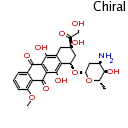

Supplement: Additional file 3 — Clusters of Compounds Shown in Figure 6. [file 1752-0509-5-32-S3.ZIP › Additional Files 3/Clustering_excluding_compounds_with_unknown_interactions_files/image5447.png]

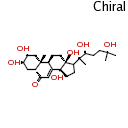

Supplement: Additional file 3 — Clusters of Compounds Shown in Figure 6. [file 1752-0509-5-32-S3.ZIP › Additional Files 3/Clustering_excluding_compounds_with_unknown_interactions_files/image5448.png]

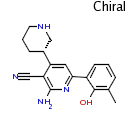

Supplement: Additional file 3 — Clusters of Compounds Shown in Figure 6. [file 1752-0509-5-32-S3.ZIP › Additional Files 3/Clustering_excluding_compounds_with_unknown_interactions_files/image5449.png]

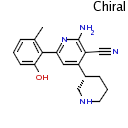

Supplement: Additional file 3 — Clusters of Compounds Shown in Figure 6. [file 1752-0509-5-32-S3.ZIP › Additional Files 3/Clustering_excluding_compounds_with_unknown_interactions_files/image5450.png]

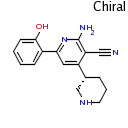

Supplement: Additional file 3 — Clusters of Compounds Shown in Figure 6. [file 1752-0509-5-32-S3.ZIP › Additional Files 3/Clustering_excluding_compounds_with_unknown_interactions_files/image5451.png]

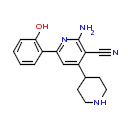

Supplement: Additional file 3 — Clusters of Compounds Shown in Figure 6. [file 1752-0509-5-32-S3.ZIP › Additional Files 3/Clustering_excluding_compounds_with_unknown_interactions_files/image5452.png]

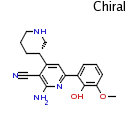

Supplement: Additional file 3 — Clusters of Compounds Shown in Figure 6. [file 1752-0509-5-32-S3.ZIP › Additional Files 3/Clustering_excluding_compounds_with_unknown_interactions_files/image5453.png]

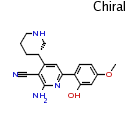

Supplement: Additional file 3 — Clusters of Compounds Shown in Figure 6. [file 1752-0509-5-32-S3.ZIP › Additional Files 3/Clustering_excluding_compounds_with_unknown_interactions_files/image5454.png]

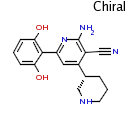

Supplement: Additional file 3 — Clusters of Compounds Shown in Figure 6. [file 1752-0509-5-32-S3.ZIP › Additional Files 3/Clustering_excluding_compounds_with_unknown_interactions_files/image5455.png]

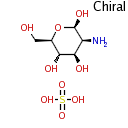

Supplement: Additional file 3 — Clusters of Compounds Shown in Figure 6. [file 1752-0509-5-32-S3.ZIP › Additional Files 3/Clustering_excluding_compounds_with_unknown_interactions_files/image5457.png]

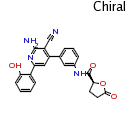

Supplement: Additional file 3 — Clusters of Compounds Shown in Figure 6. [file 1752-0509-5-32-S3.ZIP › Additional Files 3/Clustering_excluding_compounds_with_unknown_interactions_files/image5460.png]

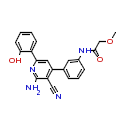

Supplement: Additional file 3 — Clusters of Compounds Shown in Figure 6. [file 1752-0509-5-32-S3.ZIP › Additional Files 3/Clustering_excluding_compounds_with_unknown_interactions_files/image5463.png]

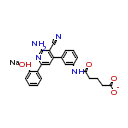

Supplement: Additional file 3 — Clusters of Compounds Shown in Figure 6. [file 1752-0509-5-32-S3.ZIP › Additional Files 3/Clustering_excluding_compounds_with_unknown_interactions_files/image5464.png]

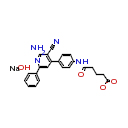

Supplement: Additional file 3 — Clusters of Compounds Shown in Figure 6. [file 1752-0509-5-32-S3.ZIP › Additional Files 3/Clustering_excluding_compounds_with_unknown_interactions_files/image5465.png]

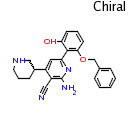

Supplement: Additional file 3 — Clusters of Compounds Shown in Figure 6. [file 1752-0509-5-32-S3.ZIP › Additional Files 3/Clustering_excluding_compounds_with_unknown_interactions_files/image5466.png]

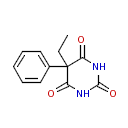

Supplement: Additional file 3 — Clusters of Compounds Shown in Figure 6. [file 1752-0509-5-32-S3.ZIP › Additional Files 3/Clustering_excluding_compounds_with_unknown_interactions_files/image5468.png]

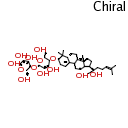

Supplement: Additional file 3 — Clusters of Compounds Shown in Figure 6. [file 1752-0509-5-32-S3.ZIP › Additional Files 3/Clustering_including_compounds_with_unknown_interactions_files/image49642.png]

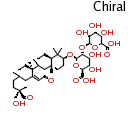

Supplement: Additional file 3 — Clusters of Compounds Shown in Figure 6. [file 1752-0509-5-32-S3.ZIP › Additional Files 3/Clustering_including_compounds_with_unknown_interactions_files/image49643.png]

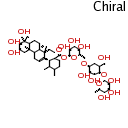

Supplement: Additional file 3 — Clusters of Compounds Shown in Figure 6. [file 1752-0509-5-32-S3.ZIP › Additional Files 3/Clustering_including_compounds_with_unknown_interactions_files/image49644.png]

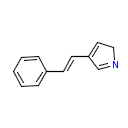

Supplement: Additional file 3 — Clusters of Compounds Shown in Figure 6. [file 1752-0509-5-32-S3.ZIP › Additional Files 3/Clustering_including_compounds_with_unknown_interactions_files/image49650.png]

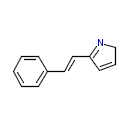

Supplement: Additional file 3 — Clusters of Compounds Shown in Figure 6. [file 1752-0509-5-32-S3.ZIP › Additional Files 3/Clustering_including_compounds_with_unknown_interactions_files/image49651.png]

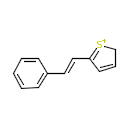

Supplement: Additional file 3 — Clusters of Compounds Shown in Figure 6. [file 1752-0509-5-32-S3.ZIP › Additional Files 3/Clustering_including_compounds_with_unknown_interactions_files/image49652.png]

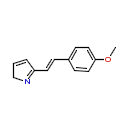

Supplement: Additional file 3 — Clusters of Compounds Shown in Figure 6. [file 1752-0509-5-32-S3.ZIP › Additional Files 3/Clustering_including_compounds_with_unknown_interactions_files/image49653.png]

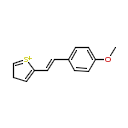

Supplement: Additional file 3 — Clusters of Compounds Shown in Figure 6. [file 1752-0509-5-32-S3.ZIP › Additional Files 3/Clustering_including_compounds_with_unknown_interactions_files/image49655.png]

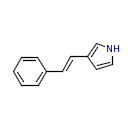

Supplement: Additional file 3 — Clusters of Compounds Shown in Figure 6. [file 1752-0509-5-32-S3.ZIP › Additional Files 3/Clustering_including_compounds_with_unknown_interactions_files/image49656.png]

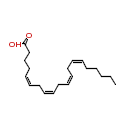

Supplement: Additional file 3 — Clusters of Compounds Shown in Figure 6. [file 1752-0509-5-32-S3.ZIP › Additional Files 3/Clustering_including_compounds_with_unknown_interactions_files/image49660.png]

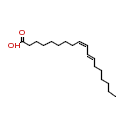

Supplement: Additional file 3 — Clusters of Compounds Shown in Figure 6. [file 1752-0509-5-32-S3.ZIP › Additional Files 3/Clustering_including_compounds_with_unknown_interactions_files/image49661.png]
