# Supplementary figures and images for: Predicting the points of interaction of small molecules in the NF-κB pathway (part 5 of 6)
Source: BMC Syst Biol. 2011 Feb 22;5:32. doi: 10.1186/1752-0509-5-32 (PMC3050742; doi:10.1186/1752-0509-5-32)

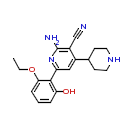

Supplement: Additional file 3 — Clusters of Compounds Shown in Figure 6. [file 1752-0509-5-32-S3.ZIP › Additional Files 3/Clustering_excluding_compounds_with_unknown_interactions_files/image5298.png]

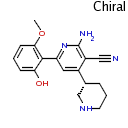

Supplement: Additional file 3 — Clusters of Compounds Shown in Figure 6. [file 1752-0509-5-32-S3.ZIP › Additional Files 3/Clustering_excluding_compounds_with_unknown_interactions_files/image5299.png]

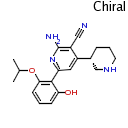

Supplement: Additional file 3 — Clusters of Compounds Shown in Figure 6. [file 1752-0509-5-32-S3.ZIP › Additional Files 3/Clustering_excluding_compounds_with_unknown_interactions_files/image5300.png]

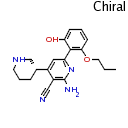

Supplement: Additional file 3 — Clusters of Compounds Shown in Figure 6. [file 1752-0509-5-32-S3.ZIP › Additional Files 3/Clustering_excluding_compounds_with_unknown_interactions_files/image5301.png]

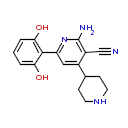

Supplement: Additional file 3 — Clusters of Compounds Shown in Figure 6. [file 1752-0509-5-32-S3.ZIP › Additional Files 3/Clustering_excluding_compounds_with_unknown_interactions_files/image5302.png]

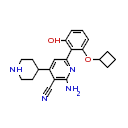

Supplement: Additional file 3 — Clusters of Compounds Shown in Figure 6. [file 1752-0509-5-32-S3.ZIP › Additional Files 3/Clustering_excluding_compounds_with_unknown_interactions_files/image5303.png]

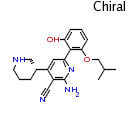

Supplement: Additional file 3 — Clusters of Compounds Shown in Figure 6. [file 1752-0509-5-32-S3.ZIP › Additional Files 3/Clustering_excluding_compounds_with_unknown_interactions_files/image5304.png]

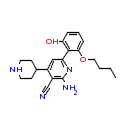

Supplement: Additional file 3 — Clusters of Compounds Shown in Figure 6. [file 1752-0509-5-32-S3.ZIP › Additional Files 3/Clustering_excluding_compounds_with_unknown_interactions_files/image5305.png]

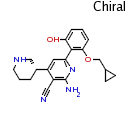

Supplement: Additional file 3 — Clusters of Compounds Shown in Figure 6. [file 1752-0509-5-32-S3.ZIP › Additional Files 3/Clustering_excluding_compounds_with_unknown_interactions_files/image5306.png]

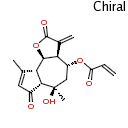

Supplement: Additional file 3 — Clusters of Compounds Shown in Figure 6. [file 1752-0509-5-32-S3.ZIP › Additional Files 3/Clustering_excluding_compounds_with_unknown_interactions_files/image5307.png]

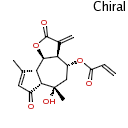

Supplement: Additional file 3 — Clusters of Compounds Shown in Figure 6. [file 1752-0509-5-32-S3.ZIP › Additional Files 3/Clustering_excluding_compounds_with_unknown_interactions_files/image5308.png]

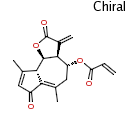

Supplement: Additional file 3 — Clusters of Compounds Shown in Figure 6. [file 1752-0509-5-32-S3.ZIP › Additional Files 3/Clustering_excluding_compounds_with_unknown_interactions_files/image5309.png]

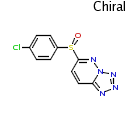

Supplement: Additional file 3 — Clusters of Compounds Shown in Figure 6. [file 1752-0509-5-32-S3.ZIP › Additional Files 3/Clustering_excluding_compounds_with_unknown_interactions_files/image5310.png]

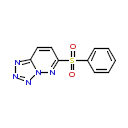

Supplement: Additional file 3 — Clusters of Compounds Shown in Figure 6. [file 1752-0509-5-32-S3.ZIP › Additional Files 3/Clustering_excluding_compounds_with_unknown_interactions_files/image5312.png]

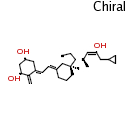

Supplement: Additional file 3 — Clusters of Compounds Shown in Figure 6. [file 1752-0509-5-32-S3.ZIP › Additional Files 3/Clustering_excluding_compounds_with_unknown_interactions_files/image5315.png]

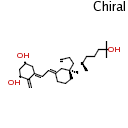

Supplement: Additional file 3 — Clusters of Compounds Shown in Figure 6. [file 1752-0509-5-32-S3.ZIP › Additional Files 3/Clustering_excluding_compounds_with_unknown_interactions_files/image5316.png]

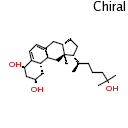

Supplement: Additional file 3 — Clusters of Compounds Shown in Figure 6. [file 1752-0509-5-32-S3.ZIP › Additional Files 3/Clustering_excluding_compounds_with_unknown_interactions_files/image5317.png]

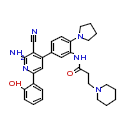

Supplement: Additional file 3 — Clusters of Compounds Shown in Figure 6. [file 1752-0509-5-32-S3.ZIP › Additional Files 3/Clustering_excluding_compounds_with_unknown_interactions_files/image5318.png]

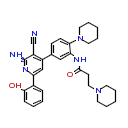

Supplement: Additional file 3 — Clusters of Compounds Shown in Figure 6. [file 1752-0509-5-32-S3.ZIP › Additional Files 3/Clustering_excluding_compounds_with_unknown_interactions_files/image5319.png]

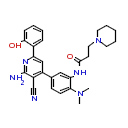

Supplement: Additional file 3 — Clusters of Compounds Shown in Figure 6. [file 1752-0509-5-32-S3.ZIP › Additional Files 3/Clustering_excluding_compounds_with_unknown_interactions_files/image5320.png]

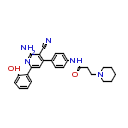

Supplement: Additional file 3 — Clusters of Compounds Shown in Figure 6. [file 1752-0509-5-32-S3.ZIP › Additional Files 3/Clustering_excluding_compounds_with_unknown_interactions_files/image5321.png]

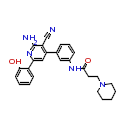

Supplement: Additional file 3 — Clusters of Compounds Shown in Figure 6. [file 1752-0509-5-32-S3.ZIP › Additional Files 3/Clustering_excluding_compounds_with_unknown_interactions_files/image5322.png]

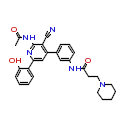

Supplement: Additional file 3 — Clusters of Compounds Shown in Figure 6. [file 1752-0509-5-32-S3.ZIP › Additional Files 3/Clustering_excluding_compounds_with_unknown_interactions_files/image5323.png]

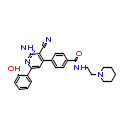

Supplement: Additional file 3 — Clusters of Compounds Shown in Figure 6. [file 1752-0509-5-32-S3.ZIP › Additional Files 3/Clustering_excluding_compounds_with_unknown_interactions_files/image5324.png]

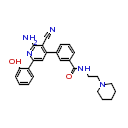

Supplement: Additional file 3 — Clusters of Compounds Shown in Figure 6. [file 1752-0509-5-32-S3.ZIP › Additional Files 3/Clustering_excluding_compounds_with_unknown_interactions_files/image5325.png]

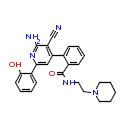

Supplement: Additional file 3 — Clusters of Compounds Shown in Figure 6. [file 1752-0509-5-32-S3.ZIP › Additional Files 3/Clustering_excluding_compounds_with_unknown_interactions_files/image5326.png]

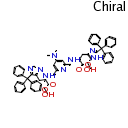

Supplement: Additional file 3 — Clusters of Compounds Shown in Figure 6. [file 1752-0509-5-32-S3.ZIP › Additional Files 3/Clustering_excluding_compounds_with_unknown_interactions_files/image5328.png]

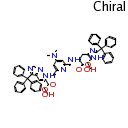

Supplement: Additional file 3 — Clusters of Compounds Shown in Figure 6. [file 1752-0509-5-32-S3.ZIP › Additional Files 3/Clustering_excluding_compounds_with_unknown_interactions_files/image5329.png]

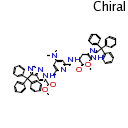

Supplement: Additional file 3 — Clusters of Compounds Shown in Figure 6. [file 1752-0509-5-32-S3.ZIP › Additional Files 3/Clustering_excluding_compounds_with_unknown_interactions_files/image5330.png]

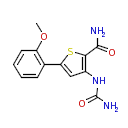

Supplement: Additional file 3 — Clusters of Compounds Shown in Figure 6. [file 1752-0509-5-32-S3.ZIP › Additional Files 3/Clustering_excluding_compounds_with_unknown_interactions_files/image5331.png]

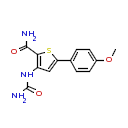

Supplement: Additional file 3 — Clusters of Compounds Shown in Figure 6. [file 1752-0509-5-32-S3.ZIP › Additional Files 3/Clustering_excluding_compounds_with_unknown_interactions_files/image5332.png]

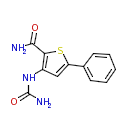

Supplement: Additional file 3 — Clusters of Compounds Shown in Figure 6. [file 1752-0509-5-32-S3.ZIP › Additional Files 3/Clustering_excluding_compounds_with_unknown_interactions_files/image5333.png]

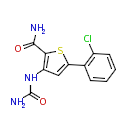

Supplement: Additional file 3 — Clusters of Compounds Shown in Figure 6. [file 1752-0509-5-32-S3.ZIP › Additional Files 3/Clustering_excluding_compounds_with_unknown_interactions_files/image5334.png]

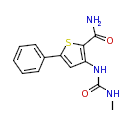

Supplement: Additional file 3 — Clusters of Compounds Shown in Figure 6. [file 1752-0509-5-32-S3.ZIP › Additional Files 3/Clustering_excluding_compounds_with_unknown_interactions_files/image5335.png]

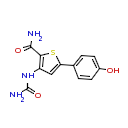

Supplement: Additional file 3 — Clusters of Compounds Shown in Figure 6. [file 1752-0509-5-32-S3.ZIP › Additional Files 3/Clustering_excluding_compounds_with_unknown_interactions_files/image5336.png]

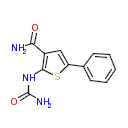

Supplement: Additional file 3 — Clusters of Compounds Shown in Figure 6. [file 1752-0509-5-32-S3.ZIP › Additional Files 3/Clustering_excluding_compounds_with_unknown_interactions_files/image5337.png]

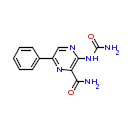

Supplement: Additional file 3 — Clusters of Compounds Shown in Figure 6. [file 1752-0509-5-32-S3.ZIP › Additional Files 3/Clustering_excluding_compounds_with_unknown_interactions_files/image5338.png]

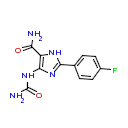

Supplement: Additional file 3 — Clusters of Compounds Shown in Figure 6. [file 1752-0509-5-32-S3.ZIP › Additional Files 3/Clustering_excluding_compounds_with_unknown_interactions_files/image5339.png]

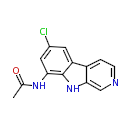

Supplement: Additional file 3 — Clusters of Compounds Shown in Figure 6. [file 1752-0509-5-32-S3.ZIP › Additional Files 3/Clustering_excluding_compounds_with_unknown_interactions_files/image5340.png]

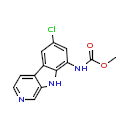

Supplement: Additional file 3 — Clusters of Compounds Shown in Figure 6. [file 1752-0509-5-32-S3.ZIP › Additional Files 3/Clustering_excluding_compounds_with_unknown_interactions_files/image5341.png]

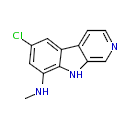

Supplement: Additional file 3 — Clusters of Compounds Shown in Figure 6. [file 1752-0509-5-32-S3.ZIP › Additional Files 3/Clustering_excluding_compounds_with_unknown_interactions_files/image5342.png]

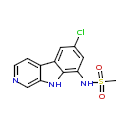

Supplement: Additional file 3 — Clusters of Compounds Shown in Figure 6. [file 1752-0509-5-32-S3.ZIP › Additional Files 3/Clustering_excluding_compounds_with_unknown_interactions_files/image5343.png]

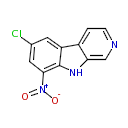

Supplement: Additional file 3 — Clusters of Compounds Shown in Figure 6. [file 1752-0509-5-32-S3.ZIP › Additional Files 3/Clustering_excluding_compounds_with_unknown_interactions_files/image5344.png]

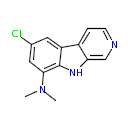

Supplement: Additional file 3 — Clusters of Compounds Shown in Figure 6. [file 1752-0509-5-32-S3.ZIP › Additional Files 3/Clustering_excluding_compounds_with_unknown_interactions_files/image5345.png]

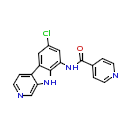

Supplement: Additional file 3 — Clusters of Compounds Shown in Figure 6. [file 1752-0509-5-32-S3.ZIP › Additional Files 3/Clustering_excluding_compounds_with_unknown_interactions_files/image5346.png]

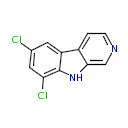

Supplement: Additional file 3 — Clusters of Compounds Shown in Figure 6. [file 1752-0509-5-32-S3.ZIP › Additional Files 3/Clustering_excluding_compounds_with_unknown_interactions_files/image5347.png]

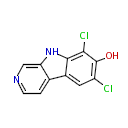

Supplement: Additional file 3 — Clusters of Compounds Shown in Figure 6. [file 1752-0509-5-32-S3.ZIP › Additional Files 3/Clustering_excluding_compounds_with_unknown_interactions_files/image5348.png]

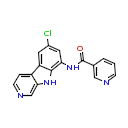

Supplement: Additional file 3 — Clusters of Compounds Shown in Figure 6. [file 1752-0509-5-32-S3.ZIP › Additional Files 3/Clustering_excluding_compounds_with_unknown_interactions_files/image5349.png]

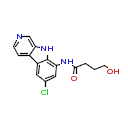

Supplement: Additional file 3 — Clusters of Compounds Shown in Figure 6. [file 1752-0509-5-32-S3.ZIP › Additional Files 3/Clustering_excluding_compounds_with_unknown_interactions_files/image5351.png]

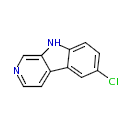

Supplement: Additional file 3 — Clusters of Compounds Shown in Figure 6. [file 1752-0509-5-32-S3.ZIP › Additional Files 3/Clustering_excluding_compounds_with_unknown_interactions_files/image5352.png]

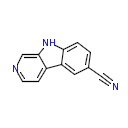

Supplement: Additional file 3 — Clusters of Compounds Shown in Figure 6. [file 1752-0509-5-32-S3.ZIP › Additional Files 3/Clustering_excluding_compounds_with_unknown_interactions_files/image5353.png]

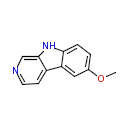

Supplement: Additional file 3 — Clusters of Compounds Shown in Figure 6. [file 1752-0509-5-32-S3.ZIP › Additional Files 3/Clustering_excluding_compounds_with_unknown_interactions_files/image5354.png]

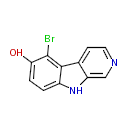

Supplement: Additional file 3 — Clusters of Compounds Shown in Figure 6. [file 1752-0509-5-32-S3.ZIP › Additional Files 3/Clustering_excluding_compounds_with_unknown_interactions_files/image5355.png]

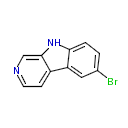

Supplement: Additional file 3 — Clusters of Compounds Shown in Figure 6. [file 1752-0509-5-32-S3.ZIP › Additional Files 3/Clustering_excluding_compounds_with_unknown_interactions_files/image5356.png]

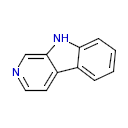

Supplement: Additional file 3 — Clusters of Compounds Shown in Figure 6. [file 1752-0509-5-32-S3.ZIP › Additional Files 3/Clustering_excluding_compounds_with_unknown_interactions_files/image5357.png]

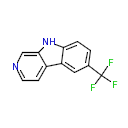

Supplement: Additional file 3 — Clusters of Compounds Shown in Figure 6. [file 1752-0509-5-32-S3.ZIP › Additional Files 3/Clustering_excluding_compounds_with_unknown_interactions_files/image5358.png]

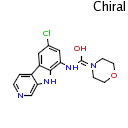

Supplement: Additional file 3 — Clusters of Compounds Shown in Figure 6. [file 1752-0509-5-32-S3.ZIP › Additional Files 3/Clustering_excluding_compounds_with_unknown_interactions_files/image5359.png]

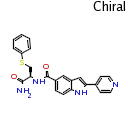

Supplement: Additional file 3 — Clusters of Compounds Shown in Figure 6. [file 1752-0509-5-32-S3.ZIP › Additional Files 3/Clustering_excluding_compounds_with_unknown_interactions_files/image5361.png]

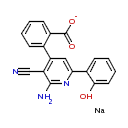

Supplement: Additional file 3 — Clusters of Compounds Shown in Figure 6. [file 1752-0509-5-32-S3.ZIP › Additional Files 3/Clustering_excluding_compounds_with_unknown_interactions_files/image5362.png]

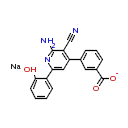

Supplement: Additional file 3 — Clusters of Compounds Shown in Figure 6. [file 1752-0509-5-32-S3.ZIP › Additional Files 3/Clustering_excluding_compounds_with_unknown_interactions_files/image5363.png]

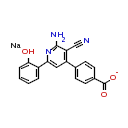

Supplement: Additional file 3 — Clusters of Compounds Shown in Figure 6. [file 1752-0509-5-32-S3.ZIP › Additional Files 3/Clustering_excluding_compounds_with_unknown_interactions_files/image5364.png]

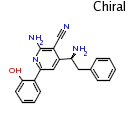

Supplement: Additional file 3 — Clusters of Compounds Shown in Figure 6. [file 1752-0509-5-32-S3.ZIP › Additional Files 3/Clustering_excluding_compounds_with_unknown_interactions_files/image5365.png]

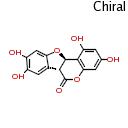

Supplement: Additional file 3 — Clusters of Compounds Shown in Figure 6. [file 1752-0509-5-32-S3.ZIP › Additional Files 3/Clustering_excluding_compounds_with_unknown_interactions_files/image5366.png]

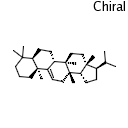

Supplement: Additional file 3 — Clusters of Compounds Shown in Figure 6. [file 1752-0509-5-32-S3.ZIP › Additional Files 3/Clustering_excluding_compounds_with_unknown_interactions_files/image5368.png]

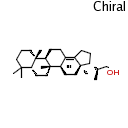

Supplement: Additional file 3 — Clusters of Compounds Shown in Figure 6. [file 1752-0509-5-32-S3.ZIP › Additional Files 3/Clustering_excluding_compounds_with_unknown_interactions_files/image5369.png]

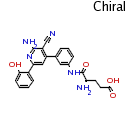

Supplement: Additional file 3 — Clusters of Compounds Shown in Figure 6. [file 1752-0509-5-32-S3.ZIP › Additional Files 3/Clustering_excluding_compounds_with_unknown_interactions_files/image5370.png]

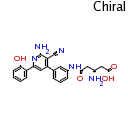

Supplement: Additional file 3 — Clusters of Compounds Shown in Figure 6. [file 1752-0509-5-32-S3.ZIP › Additional Files 3/Clustering_excluding_compounds_with_unknown_interactions_files/image5371.png]

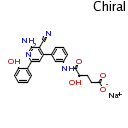

Supplement: Additional file 3 — Clusters of Compounds Shown in Figure 6. [file 1752-0509-5-32-S3.ZIP › Additional Files 3/Clustering_excluding_compounds_with_unknown_interactions_files/image5372.png]

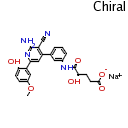

Supplement: Additional file 3 — Clusters of Compounds Shown in Figure 6. [file 1752-0509-5-32-S3.ZIP › Additional Files 3/Clustering_excluding_compounds_with_unknown_interactions_files/image5373.png]

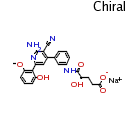

Supplement: Additional file 3 — Clusters of Compounds Shown in Figure 6. [file 1752-0509-5-32-S3.ZIP › Additional Files 3/Clustering_excluding_compounds_with_unknown_interactions_files/image5374.png]

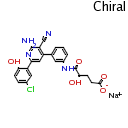

Supplement: Additional file 3 — Clusters of Compounds Shown in Figure 6. [file 1752-0509-5-32-S3.ZIP › Additional Files 3/Clustering_excluding_compounds_with_unknown_interactions_files/image5375.png]

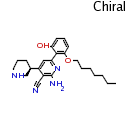

Supplement: Additional file 3 — Clusters of Compounds Shown in Figure 6. [file 1752-0509-5-32-S3.ZIP › Additional Files 3/Clustering_excluding_compounds_with_unknown_interactions_files/image5376.png]

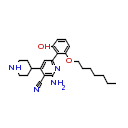

Supplement: Additional file 3 — Clusters of Compounds Shown in Figure 6. [file 1752-0509-5-32-S3.ZIP › Additional Files 3/Clustering_excluding_compounds_with_unknown_interactions_files/image5377.png]

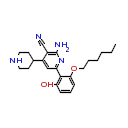

Supplement: Additional file 3 — Clusters of Compounds Shown in Figure 6. [file 1752-0509-5-32-S3.ZIP › Additional Files 3/Clustering_excluding_compounds_with_unknown_interactions_files/image5378.png]

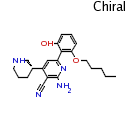

Supplement: Additional file 3 — Clusters of Compounds Shown in Figure 6. [file 1752-0509-5-32-S3.ZIP › Additional Files 3/Clustering_excluding_compounds_with_unknown_interactions_files/image5379.png]

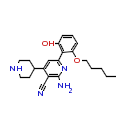

Supplement: Additional file 3 — Clusters of Compounds Shown in Figure 6. [file 1752-0509-5-32-S3.ZIP › Additional Files 3/Clustering_excluding_compounds_with_unknown_interactions_files/image5380.png]

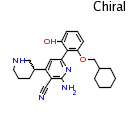

Supplement: Additional file 3 — Clusters of Compounds Shown in Figure 6. [file 1752-0509-5-32-S3.ZIP › Additional Files 3/Clustering_excluding_compounds_with_unknown_interactions_files/image5381.png]

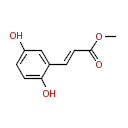

Supplement: Additional file 3 — Clusters of Compounds Shown in Figure 6. [file 1752-0509-5-32-S3.ZIP › Additional Files 3/Clustering_excluding_compounds_with_unknown_interactions_files/image5382.png]

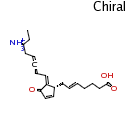

Supplement: Additional file 3 — Clusters of Compounds Shown in Figure 6. [file 1752-0509-5-32-S3.ZIP › Additional Files 3/Clustering_excluding_compounds_with_unknown_interactions_files/image5384.png]

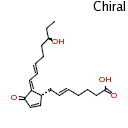

Supplement: Additional file 3 — Clusters of Compounds Shown in Figure 6. [file 1752-0509-5-32-S3.ZIP › Additional Files 3/Clustering_excluding_compounds_with_unknown_interactions_files/image5385.png]

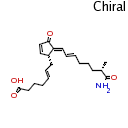

Supplement: Additional file 3 — Clusters of Compounds Shown in Figure 6. [file 1752-0509-5-32-S3.ZIP › Additional Files 3/Clustering_excluding_compounds_with_unknown_interactions_files/image5386.png]

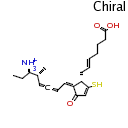

Supplement: Additional file 3 — Clusters of Compounds Shown in Figure 6. [file 1752-0509-5-32-S3.ZIP › Additional Files 3/Clustering_excluding_compounds_with_unknown_interactions_files/image5388.png]

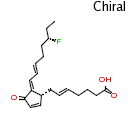

Supplement: Additional file 3 — Clusters of Compounds Shown in Figure 6. [file 1752-0509-5-32-S3.ZIP › Additional Files 3/Clustering_excluding_compounds_with_unknown_interactions_files/image5389.png]

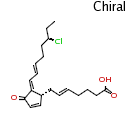

Supplement: Additional file 3 — Clusters of Compounds Shown in Figure 6. [file 1752-0509-5-32-S3.ZIP › Additional Files 3/Clustering_excluding_compounds_with_unknown_interactions_files/image5390.png]

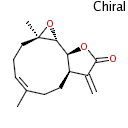

Supplement: Additional file 3 — Clusters of Compounds Shown in Figure 6. [file 1752-0509-5-32-S3.ZIP › Additional Files 3/Clustering_excluding_compounds_with_unknown_interactions_files/image5392.png]

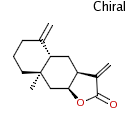

Supplement: Additional file 3 — Clusters of Compounds Shown in Figure 6. [file 1752-0509-5-32-S3.ZIP › Additional Files 3/Clustering_excluding_compounds_with_unknown_interactions_files/image5393.png]

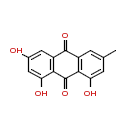

Supplement: Additional file 3 — Clusters of Compounds Shown in Figure 6. [file 1752-0509-5-32-S3.ZIP › Additional Files 3/Clustering_excluding_compounds_with_unknown_interactions_files/image5394.png]

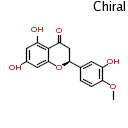

Supplement: Additional file 3 — Clusters of Compounds Shown in Figure 6. [file 1752-0509-5-32-S3.ZIP › Additional Files 3/Clustering_excluding_compounds_with_unknown_interactions_files/image5396.png]

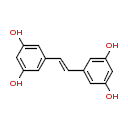

Supplement: Additional file 3 — Clusters of Compounds Shown in Figure 6. [file 1752-0509-5-32-S3.ZIP › Additional Files 3/Clustering_excluding_compounds_with_unknown_interactions_files/image5397.png]

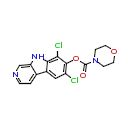

Supplement: Additional file 3 — Clusters of Compounds Shown in Figure 6. [file 1752-0509-5-32-S3.ZIP › Additional Files 3/Clustering_excluding_compounds_with_unknown_interactions_files/image5398.png]

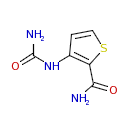

Supplement: Additional file 3 — Clusters of Compounds Shown in Figure 6. [file 1752-0509-5-32-S3.ZIP › Additional Files 3/Clustering_excluding_compounds_with_unknown_interactions_files/image5402.png]

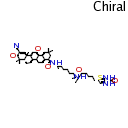

Supplement: Additional file 3 — Clusters of Compounds Shown in Figure 6. [file 1752-0509-5-32-S3.ZIP › Additional Files 3/Clustering_excluding_compounds_with_unknown_interactions_files/image5405.png]

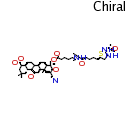

Supplement: Additional file 3 — Clusters of Compounds Shown in Figure 6. [file 1752-0509-5-32-S3.ZIP › Additional Files 3/Clustering_excluding_compounds_with_unknown_interactions_files/image5406.png]

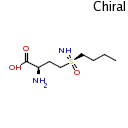

Supplement: Additional file 3 — Clusters of Compounds Shown in Figure 6. [file 1752-0509-5-32-S3.ZIP › Additional Files 3/Clustering_excluding_compounds_with_unknown_interactions_files/image5408.png]

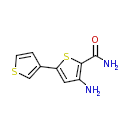

Supplement: Additional file 3 — Clusters of Compounds Shown in Figure 6. [file 1752-0509-5-32-S3.ZIP › Additional Files 3/Clustering_excluding_compounds_with_unknown_interactions_files/image5412.png]

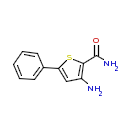

Supplement: Additional file 3 — Clusters of Compounds Shown in Figure 6. [file 1752-0509-5-32-S3.ZIP › Additional Files 3/Clustering_excluding_compounds_with_unknown_interactions_files/image5413.png]

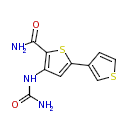

Supplement: Additional file 3 — Clusters of Compounds Shown in Figure 6. [file 1752-0509-5-32-S3.ZIP › Additional Files 3/Clustering_excluding_compounds_with_unknown_interactions_files/image5414.png]

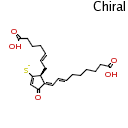

Supplement: Additional file 3 — Clusters of Compounds Shown in Figure 6. [file 1752-0509-5-32-S3.ZIP › Additional Files 3/Clustering_excluding_compounds_with_unknown_interactions_files/image5416.png]

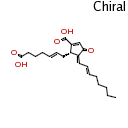

Supplement: Additional file 3 — Clusters of Compounds Shown in Figure 6. [file 1752-0509-5-32-S3.ZIP › Additional Files 3/Clustering_excluding_compounds_with_unknown_interactions_files/image5418.png]

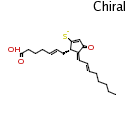

Supplement: Additional file 3 — Clusters of Compounds Shown in Figure 6. [file 1752-0509-5-32-S3.ZIP › Additional Files 3/Clustering_excluding_compounds_with_unknown_interactions_files/image5420.png]
