# Supplementary material for: Predicting the points of interaction of small molecules in the NF-κB pathway
Source: BMC Syst Biol. 2011 Feb 22;5:32. doi: 10.1186/1752-0509-5-32 (PMC3050742; doi:10.1186/1752-0509-5-32)
Supplement: Additional file 1 — List of Compounds that interact with NF-kB. [file 1752-0509-5-32-S1.ZIP › Additional Files 1/Compounds_with_more_than_one_interaction.htm]

|  |  |  |  |  |  |  |  |  |
| --- | --- | --- | --- | --- | --- | --- | --- | --- |
| Molecule | Name | Ref | inhibits\_IkB\_degradation\_phosphorylation | inhibits\_IKK\_activation | inhibits\_translocation | activates\_IkB\_phosphorylation\_degradation | DNA\_interaction | ROS\_interaction |
|  | Losartan | Chen (2002) J. Cardio. Pharmacol. (39) 332-339 | 1 | 0 | 0 | 0 | 0 | 1 |
|  | pyrrolidine dithiocarbamate PDTC Pyrrolidine dithiocarbamic acid | Schreck et al. (1992) J Exp Med (175) 1181-1194 Bessho et al (1994) Biochem Pharma (48) 1883-1889 Ferran et al. (1995) Biochem. Biophys. Res. Comm. (214) 212-223 Cuzzocrea et al (2002) Bre. J. Pharmacol. (135) 496-510 | 1 | 0 | 0 | 0 | 0 | 1 |
|  | Part of BSASM mixture (-)-epigallocatechin gallate (EGCG) Epigallocatechin gallate (-)-Epigallocatechin 3-O-gallate (-)-Epigallocatechin gallate (-)-Epigallocatechin gallate (85% (-)-epigallocatechin gallate, 10% (-)-epigallocatechin, 5% (-)- epicatechin galla te) (-)-Epigallocatechol gallate (2R,3R)-5,7-dihydroxy-2-(3,4,5-trihydroxyphenyl)-3,4-dihydro-2H-chromen-3-yl 3,4,5-trihydroxybenzoate 3,4,5-Trihydroxybenzoic acid (2R-cis)-3,4-dihydro-5,7-dihydroxy-2-(3,4,5-trihydroxyphenyl)-2H-1-benzopyran-3-yl est er 989-51-5[RN] Benzoic acid, 3,4,5-trihydroxy-, (2R,3R)-3,4-dihydro-5,7-dihydroxy-2-(3,4,5-trihydroxyphenyl)-2H-1-benzopyran-3-yl esterBenzoic acid, 3,4,5-trihydroxy-, (2R,3R)-3,4-dihydro-5,7-dihydroxy-2-(3,4,5-trihydroxyphenyl)-2H-1-benzopyran- 3-yl ester EGCG Green tea extract NOCAS[RN] (-)-Epigallocatechin gallat (-)-Epigallocatechin-3-o-gallate (-)-Epigallocatehin gallate Epigallocatcchin Gallate Epigallocate Epigallocatechin 3-gallate Epigallocic acid Galloyl-L-epigallocatechol Tea catechin (-)-EGCG (-)-epigallocatechin 3-gallate 11-cis-Retinaldehyde 19-norepiandrosterone 2-Methylbutyryl CoA 2-Methylbutyrylcarnitine 3b,12b-Dihydroxy-5b-cholanoic acid 3b,7a-Dihydroxy-cholenoic acid sulfate 6,7-Dimethyltetrahydropterin AIDS-000674 AIDS000674 Benzoquinoneacetic acid Biliverdin IX g C09731 CCRIS 3729 CHEBI:4806 Chenodeoxycholic acid 3-sulfate DivK1c\_006373 Epigallocatechin-3-gallate Epigallocatechol, 3-gallate, (-)- Glycochenodeoxycholic acid 3-glucuronide KBio1\_001317 KBio2\_000796 KBio2\_003364 KBio2\_005932 KBio3\_001128 KBioGR\_002002 KBioSS\_000796 Malonyl-CoA semialdehyde N-Carboxyethyl-gamma-aminobutyric acid R-Methylmalonyl-CoA SDCCGMLS-0066550.P001 SPBio\_000035 SpecPlus\_000277 Spectrum\_000316 Spectrum2\_000168 Spectrum3\_000244 Spectrum4\_001541 Taurocholic acid 3-sulfate Teavigo Ursodeoxycholic acid 3-sulfate | Yang et al. (1998) J. Nutr. (128) 2334-2340 Jeong et al. (2004) Pharm. Res. (21) 661-670 Lee et al. (2005) J. Ethnopharm. (96) 211-219 | 1 | 0 | 0 | 0 | 1 | 0 |
|  | plumbagin Plumbagine Plumbaein Plumbagone 2-Methyljuglone 5-Hydroxy-2-methyl-1,4-naphthoquinone BSPBio\_002546 P7262\_SIGMA Plumbagin from Plumbago indica SPECTRUM1505129 | Sandur et al (2006) J. Bio. Chem. (281) 17023-17033 | 0 | 1 | 0 | 0 | 1 | 0 |
